# Prebiotic Synthesis of *N-*Formylaminonitriles and Derivatives in Formamide

**DOI:** 10.1021/jacs.2c13306

**Published:** 2023-05-05

**Authors:** Nicholas J. Green, David A. Russell, Sasha H. Tanner, John D. Sutherland

**Affiliations:** † MRC Laboratory of Molecular Biology, Francis Crick Avenue, Cambridge Biomedical Campus, Cambridge CB2 0QH, U.K.; ‡ Department of Chemistry, University of Otago, Dunedin 9054, New Zealand

## Abstract

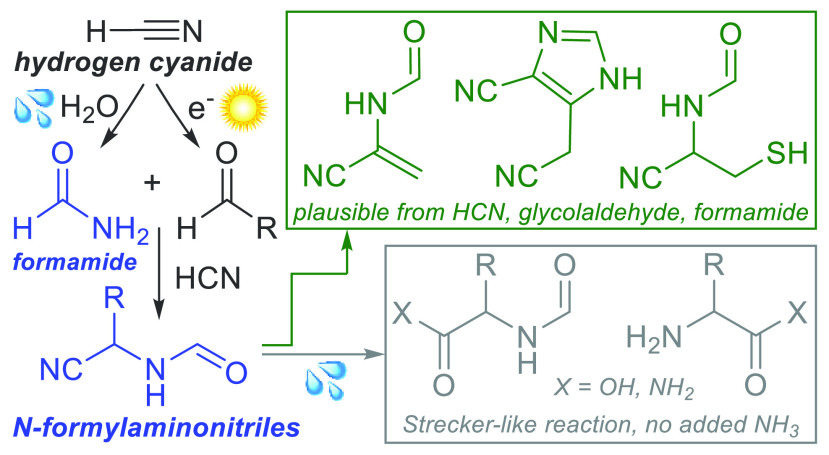

Amino acids and their derivatives were probably instrumental in the transition of prebiotic chemistry to early biology. Accordingly, amino acid formation under prebiotic conditions has been intensively investigated. Unsurprisingly, most of these studies have taken place with water as the solvent. Herein, we describe an investigation into the formation and subsequent reactions of aminonitriles and their formylated derivatives in formamide. We find that *N-*formylaminonitriles form readily from aldehydes and cyanide in formamide, even in the absence of added ammonia, suggesting a potentially prebiotic source of amino acid derivatives. Alkaline processing of *N-*formylaminonitriles proceeds with hydration at the nitrile group faster than deformylation, protecting aminonitrile derivatives from reversion of the Strecker condensation equilibrium during hydration/hydrolysis and furnishing mixtures of N-formylated and unformylated amino acid derivatives. Furthermore, the facile synthesis of *N-*formyldehydroalanine nitrile is observed in formamide from glycolaldehyde and cyanide without intervention. Dehydroalanine derivatives have been proposed as important compounds for prebiotic peptide synthesis, and we demonstrate both a synthesis suggesting that they are potentially plausible components of a prebiotic inventory, and reactions showing their utility as abiotic precursors to a range of compounds of prebiological interest.

## Introduction

Amino acids and their derivatives are indispensable to biology and a likely cornerstone of any prebiotic inventory supporting the advent of life.^1^ Chemistries potentially leading to the endogenous production of amino acid derivatives include the Strecker reaction,^2^ Bucherer–Bergs reaction,^3,4^ reductive amination of α-ketoacids,^5,6^ and hydrolytic processing of oligomers of HCN and its derivatives.^7−10^ None of these pathways toward the accumulation of amino acid derivatives is without potential drawbacks in a prebiotic context: the Strecker reaction requires relatively high concentrations of ammonia,^11^ is pH-sensitive,^12,13^ and furnishes varied products after hydration/hydrolysis;^14^ reductive amination under prebiotic conditions also requires high ammonia concentration and pH control and is low-yielding and limited in scope;^15,16^ the Bucherer–Bergs chemistry under an atmosphere of CO_2_ provides relatively refractory hydantoins and carbamoyl amino acids,^13^ and pathways based on the oligomerization of HCN and its derivatives provide complex mixtures.^7,17^ While these aqueous chemistries have been extensively investigated, surprisingly, the synthesis of amino acid derivatives in formamide, a medium commonly invoked in prebiotic chemistry, has not progressed beyond parts per million—yielding thermal and photochemical decompositions of formamide itself.^18−20^ Although the occurrence of primordial terrestrial formamide is debated,^21^ mechanisms for its potential accumulation exist.^22^ Formamide could also be accumulated by the reaction of hydrogen cyanide with hydrosulfide to give thioformamide,^23^ which is hydrolyzed to formamide more rapidly than formamide itself is hydrolyzed to ammonium formate. Given the relationship between HCN, its hydration product (formamide), and aldehydic products of its reduction and homologation,^24,25^ the co-occurrence and reactions of the constituents of aminonitriles (HCN, aldehydes, and ammonia) in formamide-rich media are of potential prebiotic relevance. Also, given a major problem in biomimetic peptide synthesis (diketopiperazine formation^26^) and its solution in bacteria (initiation of peptide synthesis with an *N-*formyl-protected amino acid^27^), we wondered if amino acid derivative synthesis in formamide and subsequent aqueous processing might provide mixtures of formylated and unformylated amino acids, as a potential basis for a similar, prebiotic solution to the problem of diketopiperazine formation. Thus, we set out to explore the formation and reactivity of amino acid building blocks in formamide.

## Results and Discussion

### *N*-Formylaminonitrile Formation

Initially, we investigated the behavior of aminonitriles **1** (AA-CN, AA = amino acid) in formamide. Unsurprisingly, given synthetic formylation procedures,^28,29^ glycine nitrile (Gly-CN **1a**), alanine nitrile (Ala-CN **1b**), and valine nitrile (Val-CN **1c**) were readily *N*-formylated in good yield by heating their acid salts in formamide in an open vessel exposed to air and moisture (Table 1, entries 1–3; Graph S1; and Figures S1–S9). Gly-CN **1a** formylated at an appreciable rate at room temperature (RT), while Ala-CN **1b** and Val-CN **1c** required heating to 80 °C to achieve high conversion after16 h. Formylated aminonitriles (FoAA-CN **2**) FoGly-CN **2a**, FoAla-CN **2b**, and FoVal-CN **2c** were stable when heated to 100 °C in formamide, without any additional products being observed. Experiments employing a ^15^N-labeled aminonitrile demonstrated that the major mechanism of formylation is transamidation between the aminonitrile and formamide, rather than a pathway involving initial reversion of the Strecker condensation (see Table S5 and Figures S145–S147). Serine nitrile (Ser-CN **1d**) was also formylated relatively quickly (Table 1, entries 4), but at temperatures above 50 °C, we observed its disappearance as it was converted to other products. This effect was especially apparent in the presence of MgCl_2_ (5 equiv), with either the free base or HNO_3_ salt of Ser-CN (Table 1, entries 4b and 4c; Figures S10–S13).

**Table 1:**
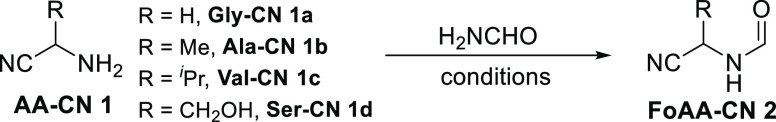
Formylation of α-Aminonitriles (AA-CN) in Formamide

		conversion (%)^a^						
entry	AA-CN	RT,^e^ 1 h	RT, +16 h	50 °C, +4 h	50 °C, +16 h	80 °C, +5 h	80 °C, +16 h	80 °C, +20 h
1a	Gly-CN·HCl	6	67	∼90^b^	nd^b^	nd^b^	∼90^b^	∼90^b^
1b	Gly-CN·HCl^c^	nd	28	77	∼90^b^	∼90^b^	∼90^b^	∼90^b^
2a	Ala-CN·HCl	1	13	20	25	37	57	65
2b	Ala-CN·HCl^c^	nd	6	9	15	20	48	59
3a	Val-CN·HCl	0	9	28	35	47	87	88
3b	Val-CN·HCl^c^	0	6	15	26	38	85	87
4a	Ser-CN·HNO_3_	0	11	31	53	82	77	74
4b	Ser-CN·HNO_3_^c^	6	28	55	51	37	29	20
4c	Ser-CN^c^,^d^	nd	40	53	58	47	31	18

a Measured by integration relative to a ^1^H NMR standard. Conditions applied successively.

b Accurate integration not possible due to signal overlap with the water peak, but only a single product was observed by one-dimensional (1D) and two-dimensional (2D) NMR spectroscopy.

c MgCl_2_ (5 equiv) added.

d Prepared by lyophilizing an aqueous solution of Ser-CN·HNO_3_ at pH 9.2.

e RT = room temperature.

Further investigation of the formylation of Ser-CN **1d** led to the identification of *N-*formyldehydroalanine nitrile (FoDHA-CN, **3**) and (*N*,*N*′-diformyl)-β-aminoalanine nitrile (Fo(β-FoNH)Ala-CN, **2e**) as additional products (Table 2 and Figures S10–S28). Although we observed no intermediate, we postulate that FoDHA-CN **3** is formed via N- and O-formylation of Ser-CN **1d** followed by rapid elimination of formic acid to provide FoDHA-CN **3**. Given the simplicity and likely prebiotic availability of both Ser-CN **1d** and formamide,^22^ the simple heating of a mixture of the two presents an expedient route to a dehydroalanine derivative of notable recent prebiotic interest.^30^ In the presence of MgCl_2_ (5 equiv), we observed yields of up to 18% for the formation of FoDHA-CN **3** just by heating Ser-CN **1d** in formamide (Table 2, entry 3).

**Table 2:**
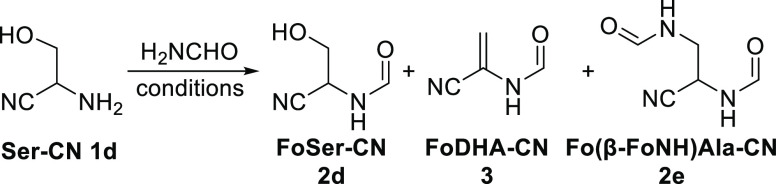
Reaction of Serine Nitrile (Ser-CN **1d**) in Formamide

		conversion (%)^a^											
		20 h, 50 °C			+5 h, 80 °C			+16 h, 80 °C			+20 h, 80 °C		
entry	conditions	**2d**	**3**	**2e**	**2d**	**3**	**2e**	**2d**	**3**	**2e**	**2d**	**3**	**2e**
1	Ser-CN·HNO_3_	53	0	0	82	1	8	77	2	9	74	4	5
2	Ser-CN·HNO_3_^b^	53	0	0	51	9	5	37	12	14	29	14	17
3	Ser-CN^b^,^c^	58	3	0	47	18	0	31	16	22	18	8	19

a Measured by relative integration compared to a ^1^H NMR spectroscopic standard. Conditions applied successively.

b MgCl_2_ added (5 equiv).

c Prepared by lyophilizing an aqueous solution of Ser-CN·HNO_3_ at pH 9.2.

Intrigued by the facile production of FoDHA-CN **3** as a byproduct of Ser-CN **1d** formylation, we wondered if the Strecker condensation itself, which produces aminonitriles such as Ser-CN **1d**, could be carried out in formamide starting from an aldehyde, cyanide, and ammonia. We began by using similar conditions to those used for aminonitrile formation in water, which require an excess of ammonia and alkaline pH to ensure the equilibrium favors aminonitrile formation.^11,12^ Under these conditions, we observed facile cyanohydrin formation in formamide and, upon heating, conversion to aminonitriles **1** and ultimately *N-*formylaminonitriles **2** of Gly, Ala, Val, and Ser (Table 3, entries 1–4; Graphs S2–S5; and Figures S29–S33). When glycolaldehyde **4d**, NaCN (3.0 equiv), and NH_4_Cl (5.0 equiv) were heated in formamide, FoSer-CN **2d** was ultimately produced without any FoDHA-CN **3** being observed, suggesting that these conditions either inhibit the formation of FoDHA-CN **3** or lead to its destruction as it forms. However, FoDHA-CN **3** could be observed, as a minor product, alongside FoSer-CN **2d** and Fo(β-FoNH)Ala-CN **2e** under various other conditions employing ammonium salts (Tables S1 and S2).

**Table 3:**
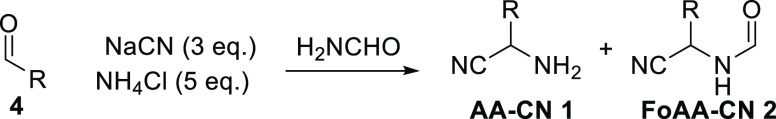
Reaction of Aldehydes and Cyanide in Formamide with Added Ammonia

		conversion^a^ (%), AA-CN (**1**)/FoAA-CN (**2**)			
entry	R	16 h, RT	+16 h, 50 °C	+16 h, 80 °C	+16 h, 80 °C
1	H^b^	0/2	32/7	5/45	0/47
2	H^c^	1/0	25/6	1/60	0/52
3	Me	35/0	58/6	32/34	9/51
4	^*i*^Pr	19/0	46/3	52/20	31/41^d^
5	CH_2_OH	7/0	36/0	35/36	0/31

a Measured by relative integration compared to a ^1^H NMR spectroscopic standard. Conditions applied successively.

b Paraformaldehyde used instead of formaldehyde.

c Glycolonitrile used in place of formaldehyde with 2 additional equivalents of NaCN.

d Yield of FoVal-CN **2c** increases to 59% after an additional 80 h heating at 80 °C.

### *N*-Formylaminonitrile Formation without Added Ammonia

**Scheme 1:**
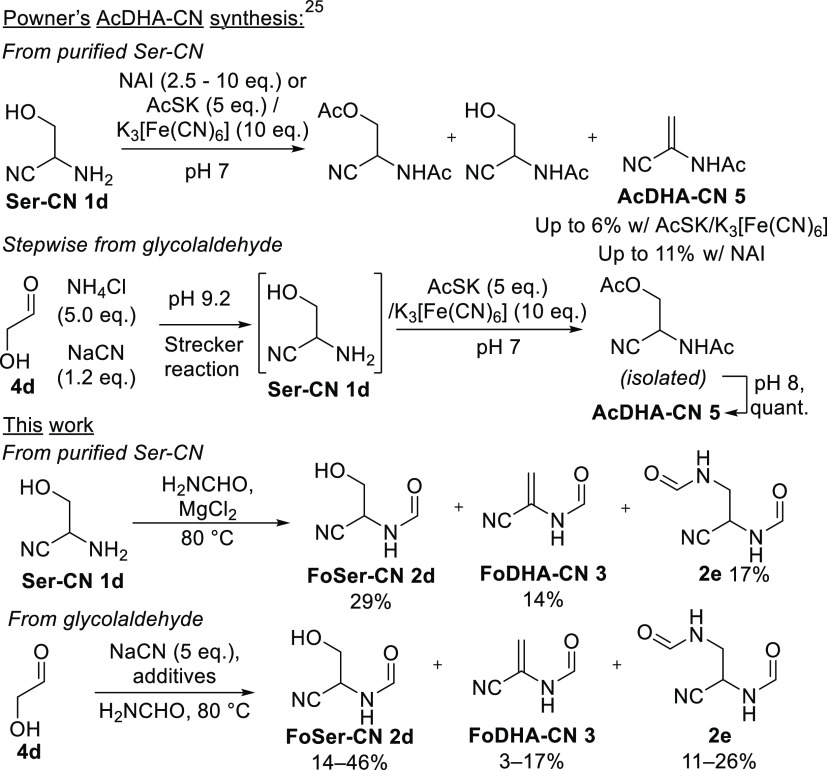
Comparison of Prebiotic Approaches to Dehydroalanine Nitrile Derivatives NAI, *N*-acetylimidazole.

We suspected that FoDHA-CN **3** probably does form alongside FoSer-CN **2d** from the Strecker reaction in formamide, but the presence of excess ammonia might lead to its decomposition. This presents a potential obstacle for the formation of any dehydroalanine derivative, since concentrations of ammonia high enough to promote aminonitrile formation^11^ are likely to lead to reaction with a dehydroalanine as it is formed (see also Table 6) and may explain why reported prebiotically themed syntheses of dehydroalanine derivatives were performed with isolation of intermediates. Eschenmoser et al. had previously synthesized dehydroalanine nitrile from an N-silylated imine precursor,^31^ and Powner et al. synthesized *N-*acetyldehydroalanine nitrile^30^ (AcDHA-CN **5**) by isolating its precursor, Ser-CN **1d**, thereby separating ammonia-requiring aminonitrile formation from alkene formation (Scheme 1). In addition to this apparent incompatibility between FoDHA-CN **3** and ammonia, the prebiotic availability of ammonia in concentrations high enough to promote the Strecker aminonitrile synthesis in useful yields has been questioned, given ammonia’s modest theoretical production rates and its volatility, reactivity, and photochemical instability.^13,32−34^ We thus set out to determine whether added ammonia is required for a Strecker-type reaction in formamide.

We next attempted aminonitrile and *N-*formylaminonitrile synthesis without added ammonia. Strecker aldehydic precursors **4** to Gly, Ala, and Val were cleanly converted to *N-*formylaminonitriles **2** in good yield when combined with equimolar quantities of sodium cyanide and formic acid and heated in formamide (Table 4, entries 1–3; Graph S6; and Figures S34–S36). Formic acid was used to neutralize the cyanide salt and better simulate conditions of partially hydrolyzed primordial formamide. Formamide is thus not only an excellent medium for the production of *N-*formylaminonitriles **2** but also a plausible source of nitrogen that is readily available for incorporation into amino acid derivatives, without requiring exogenous ammonia or pH modulation. In contrast to the reactions in Table 3, which likely proceed via a Strecker-type mechanism involving the condensation of ammonia and an aldehyde to form imines, in the absence of added ammonia, products **2** in Table 4 are likely formed via *N-*formyliminia, although we did not observe their intermediacy by ^1^H NMR.

**Table 4:**
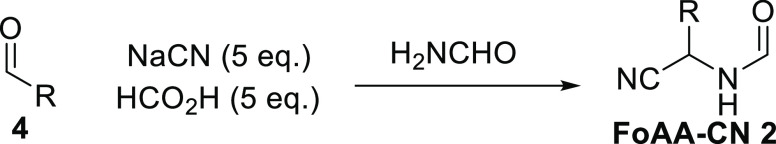
Reaction of Aldehydes and Cyanide in Formamide without Added Ammonia

		conversion^a^ (%)		
entry	R	50 °C, 16 h	+5 h, 80 °C	+16 h, 80 °C
1	H^b^	5	51	79
2	Me	0	31	85
3	^*i*^Pr	10	37	98
4	CH_2_OH	nd	nd	46

a Measured by relative integration compared to a ^1^H NMR spectroscopic standard. Conditions applied successively.

b Glycolonitrile used in place of formaldehyde with 4 additional equivalents of NaCN.

### *N*-Formyldehydroalanine Nitrile from Glycolaldehyde and Cyanide

**Figure 1:**
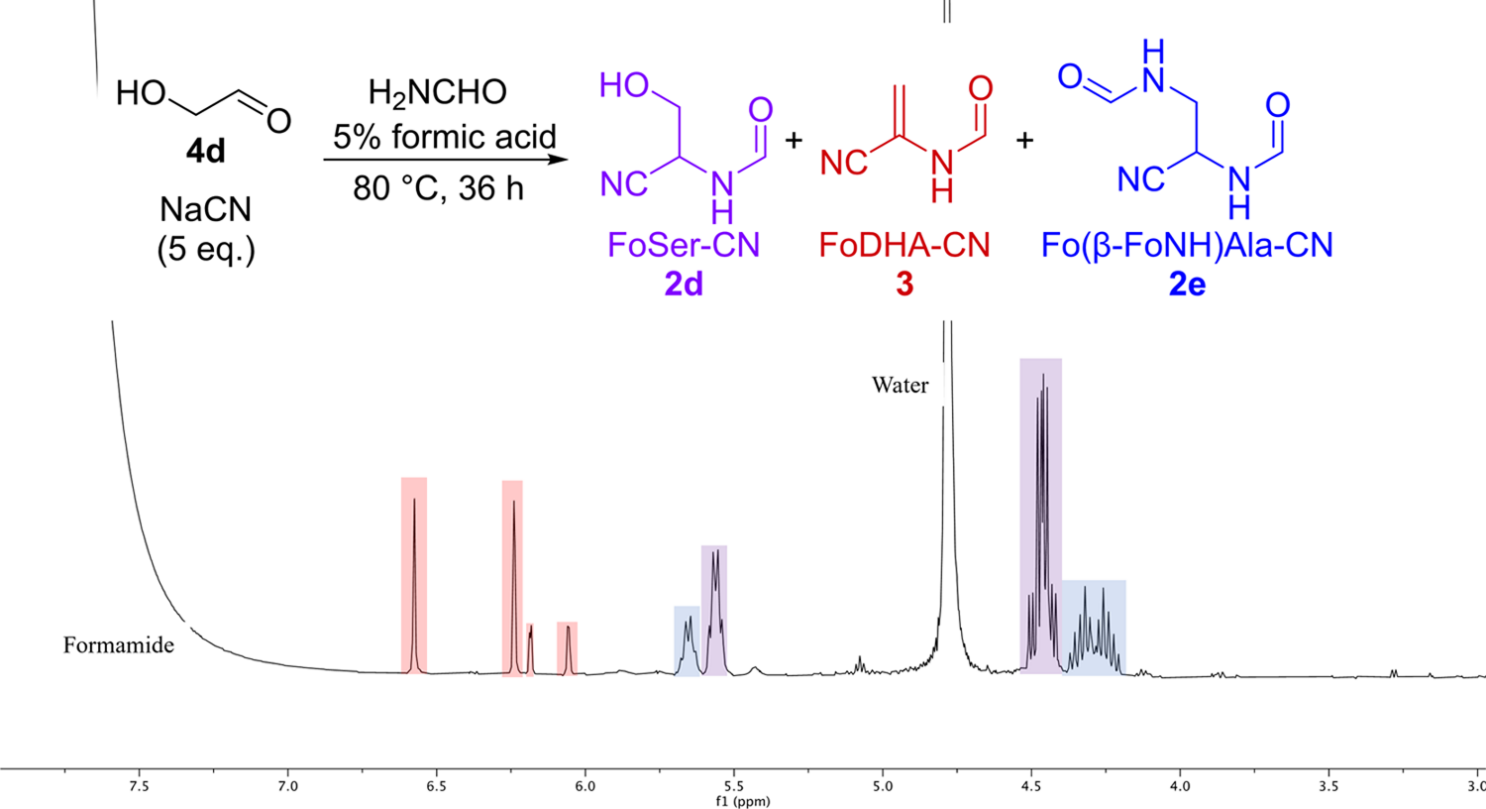
^1^H NMR spectrum showing the formation of major products FoSer-CN **2d**, FoDHA-CN **3**, and Fo(β-FoNH)Ala-CN **2e** from the reaction between NaCN and glycolaldehyde **4d** in formamide/formic acid after heating at 80 °C for 36 h. The two sets of alkenic peaks correspond to the two conformers of FoDHA-CN **2d**, presumably due to restricted rotation about the amide C–N bond.

When we combined glycoladehyde **4d**, NaCN, and formic acid in formamide without ammonia, at 80 °C, we observed the formation of FoSer-CN **2d** (Table 4, entry 4), but to a lesser extent compared to *N*-formylaminonitriles **2a**–**c**, because under these conditions FoSer-CN **2d** is converted to FoDHA-CN **3** (see Table 5 and Figure S37). Further experimentation revealed that FoDHA-CN **3** was observed alongside FoSer-CN **2d** and Fo(β-FoNH)Ala-CN **2e** under the majority of conditions tested in the presence of additives including MgCl_2_, KH_2_PO_4_, NH_4_H_2_PO_4_, and K_3_PO_4_ (Tables 5, S1, and S2). While the yield for FoDHA-CN **3** in some reactions was low, the continuous conversion of these precursors to FoDHA-CN **3** under varied conditions suggests that locales producing FoDHA-CN **3** may not have been uncommon. In some cases, the reaction was remarkably clean. For example, heating a 0.1 M formamide solution of glycolaldehyde **4d** with 5 equiv of NaCN and formic acid at 80 °C for 36 h resulted in the formation of FoDHA-CN in a yield of 16% (Table 5, entry 1). The ^1^H NMR spectrum of this reaction mixture is shown in Figure 1. Additives such as KH_2_PO_4_ or MgCl_2_ did not significantly affect this yield (17 and 9%, respectively, Table 5, entries 2 and 3). A similar reaction using only 1.5 equiv of NaCN, with 5 equiv of MgCl_2_ present, resulted in the formation of FoDHA-CN **3** in a 7% yield (Table 5, entry 4). Thus, we demonstrate that wherever formamide, glycoladehyde **4d**, and cyanide were present, it is reasonable to expect some flux of these feedstocks to FoDHA-CN **3**.

**Table 5:**
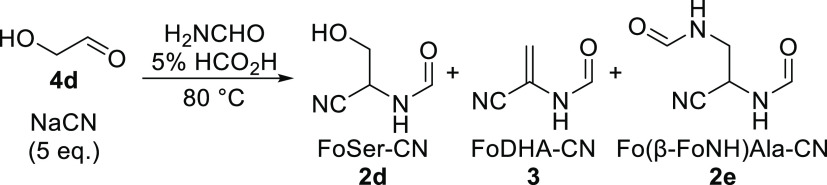
Reaction of Glycolaldehyde **4d** and Cyanide in Formamide without Added Ammonia

		conversion (%)^a^					
		16 h			36 h		
entry	additive	**2d**	**3**	**2e**	**2d**	**3**	**2e**
1	none	46	12	17	36	16	21
2	MgCl_2_ (5 equiv)	37	3	20	34	9	26
3	KH_2_PO_4_ (5 equiv)	35	18	16	23	17	21
4	MgCl_2_ (5 equiv)^b^	26	5	11	14	7	14

a Measured by relative integration compared to a ^1^H NMR spectroscopic standard.

b Reaction performed using 1.5 equiv NaCN, without added formic acid.

Our observation of the facile manner of the formation of FoDHA-CN **3** is interesting because it presents a simple and prebiotically plausible route to dehydroalanine derivatives. Dehydroalanine and its derivatives are important motifs in modern biology,^35,36^ including the biosynthesis of cysteine^37^ and α,β-diaminopropionic acid,^38^ and thus the provision of a similar molecule in a prebiotic context may have been important to allow nascent biology to access a variety of useful building blocks. Eschenmoser et al. had previously used chemically synthesized dehydroalanine nitrile to study its potential utility in prebiotic amino acid and cofactor synthesis.^31^ Moreover, Powner et al. recently showed that the related *N-*acetyldehydroalanine nitrile (AcDHA-CN **5**) is a precursor to cysteine derivatives, which have proven difficult to access under prebiotic conditions.^30^ Such derivatives were shown to be organocatalysts for peptide ligation and both peptidyl amidine and peptide synthesis.^30,39^ While Powner et al. demonstrated routes to AcDHA-CN **5** in water via acetylation and elimination, our observation of the formation of FoDHA-CN **3** by simply heating serine nitrile **1d** or its precursors in formamide, without the use of purified intermediates, synthetic acetylating agents, oxidants, pH switches, or other interventions, significantly bolsters the argument that dehydroalanine nitriles would have been present on the prebiotic earth (Scheme 1). We thus set about accessing FoDHA-CN **3** synthetically and investigating its reactivity with other molecules of prebiotic relevance in water and formamide.

### Preparative *N*-Formyldehydroalanine Nitrile Synthesis

**Scheme 2:**
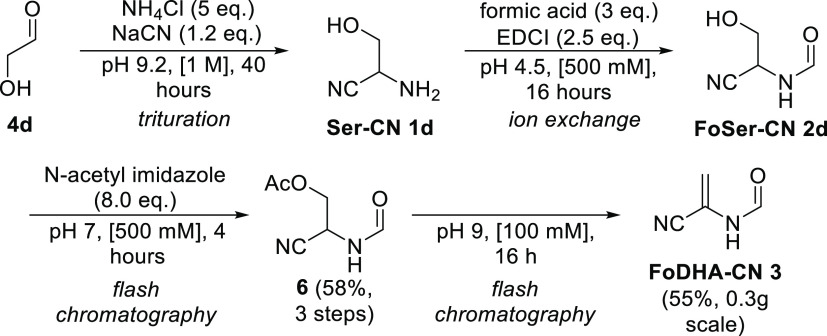
Preparative Route to FoDHA-CN

FoDHA-CN **3** was prepared synthetically from glycolaldehyde **4d** in four steps and a 32% overall yield (Scheme 2 and Figures S38–S45). Serine nitrile **1d** was prepared using the Strecker reaction^2^ and then N-formylated using the condensing agent 1-ethyl-3-(3-dimethylaminopropyl)carbodiimide (EDCI) and formic acid. Removal of the spent condensing agent by ion exchange chromatography (Dowex-Na^+^) preceded O-acetylation by *N-*acetylimidazole, which provided FoSer(Ac)-CN **7** in 50% yield. When attempting to telescope the subsequent elimination step and the acetylation step, at alkaline pH, we found that the remaining imidazole added readily to FoDHA-CN **3** as it was formed, giving **2f** (see Scheme 5). Thus, we purified FoSer(Ac)-CN **6** before performing the elimination. This observation of cross-reactivity in our preparative synthesis underscores the importance of our findings of a single-operation prebiotic synthesis of FoDHA-CN **3** in formamide, which obviates the requirement to purify intermediates.

### Additional Reactions of FoDHA-CN

With FoDHA-CN **3** in hand, we evaluated its reactivity with a variety of molecules that might reasonably be expected to have co-occurred in various primordial settings. FoDHA-CN **3** reacts readily with ammonia in both formamide and water (Table 6 and Figures S72–S83). When FoDHA-CN **3** and ammonium chloride (5 equiv) were mixed in water at pH 9.2, clean addition of ammonia to the alkene was observed to form Fo(β-NH_2_)Ala-CN **2g**, followed by migration of the formyl group to form (β-FoNH)Ala-CN **1e** in 84% yield. In formamide, FoDHA-CN **3** reacted at room temperature with NH_4_OH (5 equiv) to provide Fo(β-NH_2_)Ala-CN **2g** as the major product. A similar reaction in formamide under approximately neutral conditions, with ammonium formate (5 equiv), produced the diformyl ammonia adduct Fo(β-FoNH)Ala-CN **2e** after heating for 36 h at 80 °C. **1e**, **2g**, and **2e** are the formylated nitrile precursors to α,β-diaminopropionic acid, which is a secondary metabolite biosynthesized by the addition of ammonia to dehydroalanine generated by dehydration of serine.^38^

**Table 6:**
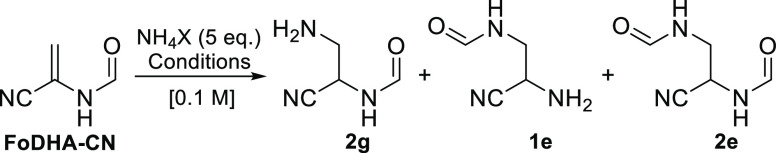
Reactions of Ammonia with FoDHA-CN **3**

		conversion (%)		
entry	conditions	**2g**	**1e**	**2e**
1	NH_4_Cl; H_2_O, pH 9.2, RT, 96 h	3	84	
2	NH_4_OH; H_2_NCHO, RT, 20 h	55	9	2
3	NH_4_O_2_CH; H_2_NCHO, 80 °C, 36 h			77

**Scheme 3:**
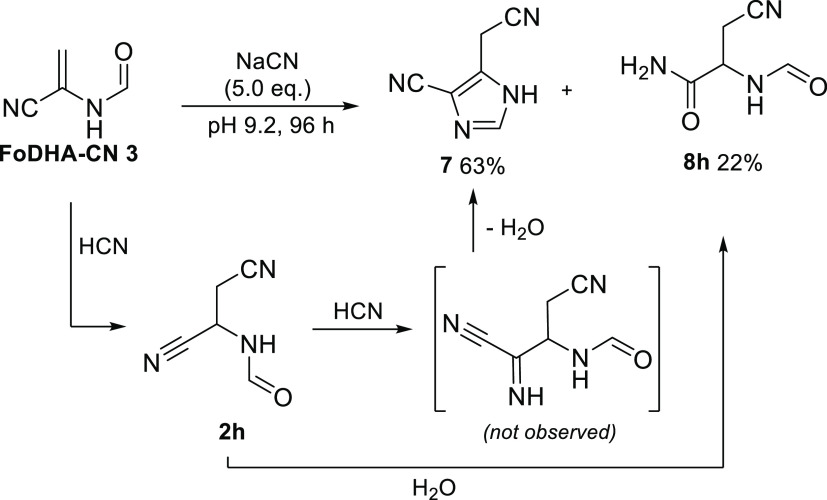
Reactions of FoDHA-CN **3** and Aqueous Cyanide

When FoDHA-CN **3** was reacted with aqueous cyanide, a cascade reaction was observed, resulting in the formation of imidazole **7**(40) in a 63% yield via the formation of three new bonds (Scheme 3 and Figures S84–S102). The hydration product, Fo(β-CN)Ala-NH_2_**8h**, of the cyanide adduct Fo(β-CN)Ala-CN **2h**, also formed, in a 22% yield. Initial addition of cyanide to FoDHA-CN **3** was observed, but adduct **2h** reacted further, undergoing a second addition of cyanide at the (*N-*formylamino)nitrile group, followed by cyclization to form imidazole **7**. This sequence was verified using ^13^C-isotopically labeled cyanide, and the same imidazole could be accessed starting from Fo(β-CN)Ala-CN **2h** and cyanide (Figures S85 and S103). When a single equivalent of NaCN was reacted with FoDHA-CN **3** at pH 9.2, cyanide adduct **2h**, its hydration product **8h**, and imidazole derivative **7** were observed in 39, 14, and 16% yields, respectively. Given the important role of imidazoles in prebiotic chemistry and nonenzymatic nucleic acid replication,^41,42^ the properties of this readily formed heterocycle in related processes are now under investigation.

**Scheme 4:**
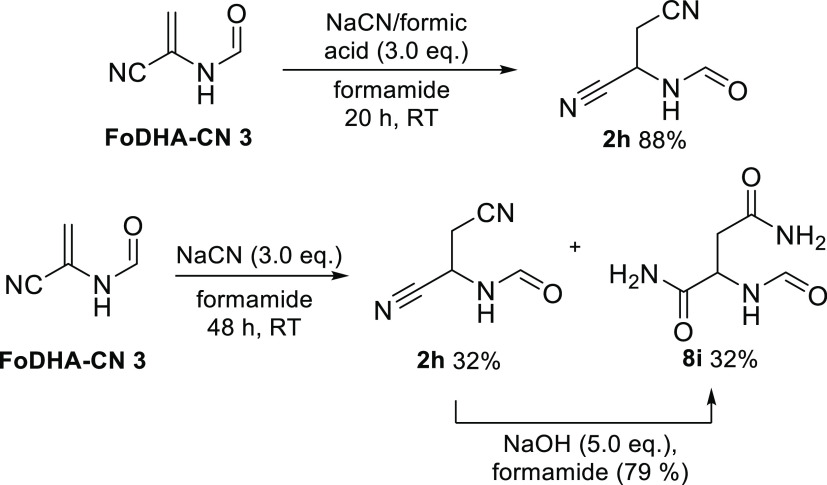
Reactions between FoDHA-CN **3** and Cyanide in Formamide

We also saw a dichotomy of reactivity of FoDHA-CN **3** with cyanide in water versus formamide. In formamide, the addition of an equimolar mixture of NaCN and formic acid (3.0 equiv) at room temperature led to the stable formation of Fo(β-CN)Ala-CN **2h** in an 88% yield (Scheme 4 and Figure S104). Without formic acid to neutralize the NaCN, we observed conversion of the starting material to cyanide adduct **2h**, but upon standing, it was slowly converted to another product, FoAsn-NH_2_**8i** (Figure S105). This *in situ* hydration of both nitrile groups is notable given the less activated nature of the β-nitrile, which is slow to hydrate in aqueous conditions (see below). This result could be recapitulated by reacting Fo(β-CN)Ala-CN **2h** with NaOH in formamide (Scheme 4 and Figure S106).

FoDHA-CN **3** reacted cleanly with the model heterocyclic nucleophile, imidazole, in water at pH 7.5 (87%) and in formamide (86%) (Scheme 5 and Figures S107–S114). The addition product **2f** was the only product observed by ^1^H NMR spectroscopy.

**Scheme 5:**
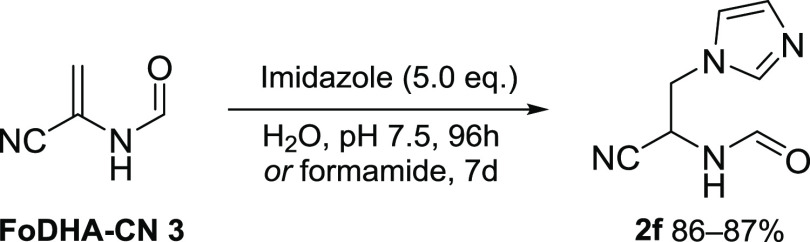
Reactions between Imidazole and FoDHA-CN **3** in Water and Formamide

Finally, given the interest in the potentially prebiotic synthesis of cysteine and its derivatives, we examined the reactivity of FoDHA-CN **3** with hydrosulfide in water and formamide (Table 7 and Figures S115–S140). We found that at neutral pH, a single hydrosulfide molecule was added to two molecules of FoDHA-CN **3**, and H_2_S subsequently the nitrile groups, forming sulfur-bridged dimers **9** and **10** (Table 7, entries 1 and 3). Similar reactivity to provide dithioamide **10** was observed in formamide, where the added NaSH·H_2_O was neutralized with formic acid (Table 7, entry 5). In contrast, under slightly alkaline conditions (pH 9), **9** formed first but was converted to *N-*formylcysteine nitrile **2j** (entry 2, 56%), with subsequent addition of hydrosulfide to the nitrile forming **11**. Similar reactivity was observed in formamide without neutralization of the added NaSH, forming **2j** (Table 7, entry 4, 83%). Thus, we have disclosed a prebiotically plausible synthesis of cysteine precursors from glycolaldehyde **4d** requiring just two straightforward steps in formamide, without the use of oxidants or protecting groups on sulfur.

**Table 7:**
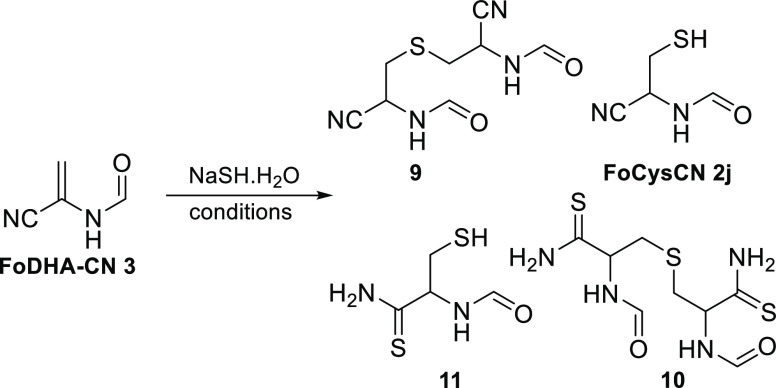
Reactions of FoDHA-CN **3** and Hydrosulfide in Water and Formamide

entry	conditions	outcome
1	1.5 equiv NaSH, pH 7, 1 h	90% **9**
2	1.5 equiv NaSH, pH 9, 2 h	56% **2j** (via **9**), 20% **11**
3	5.0 equiv NaSH, pH 7, 16 h	81% **10**
4	3.0 equiv NaSH, formamide, 6 h	83% **2j**
5	3.0 equiv NaSH + formic acid, formamide, 18 h	61% **10**

These additional reactions were all performed using purified FoDHA-CN **3** to demonstrate model reactivity in formamide and water. Now that the outcome of these reactions is characterized, further studies will investigate which may occur continuously (with all starting materials present from the outset) and which would require mutually exclusive chemistries occurring separately before a geochemically plausible combination of reagents.

### Hydrolysis of *N*-Formylaminonitriles

Having shown the potentially prebiotic synthesis of *N-*formylaminonitriles **2** in formamide, we evaluated the hydrolysis pathways that subsequent aqueous processing might bring about (Tables 8, S3, and S4; Graphs S7 and S8; and Figures S141–S144). We found that alkaline hydrolysis did not proceed via initial deformylation to give aminonitriles. Instead, at pH 10, FoGly-CN **2a** underwent hydration of the nitrile moiety and hydrolysis of the newly formed terminal amide faster than *N-*formyl hydrolysis, furnishing a mixture with FoGly-OH **12a** and FoGly-NH_2_**8a** as major products (32 and 30%, respectively) alongside smaller amounts of Gly-NH_2_**13a** and Gly-OH **14a** (16 and 12%, respectively; Table 8, entry 1). For FoAla-CN **2b**, overlapping ^1^H NMR signals prevented a precise quantitative analysis, but the same general trend in reactivity was observed, i.e., hydration and subsequent hydrolysis of the newly formed terminal amide were faster than formyl hydrolysis. After 11 days, the major products were FoAla-NH_2_**8b** (∼30%), FoAla-OH **12b** (∼30%), and FoAla-CN **2b** (∼15%), with Ala-NH_2_**13b** (∼5%), Ala-CN **1b** (∼2%), and Ala-OH **14b** (∼1%) as minor products (Table 8, entry 2). FoSer-CN **2d** underwent fairly rapid hydration of its nitrile group, providing a mixture of FoSer-NH_2_**8d**, FoSer-OH **12d**, and Ser-NH_2_**13d** as major products (Table 8, entry 4). Since the addition of cyanide to FoDHA-CN **3** provides the dinitrile precursor **2h** to *N-*formylamino acids Asn and Asp (Schemes 3 and 4), we also investigated the hydrolysis of **2h** (Table 7, entry 4). After 11 days at pH 10 and 40 °C, hydration of the α-nitrile and hydrolysis of the newly formed α-amide had occurred faster than both *N-*formyl hydrolysis and β-nitrile hydration, furnishing a mixture of FoAsn*-*OH **12i** and Fo(β-CN)Ala-OH **12h** as major products, alongside Asn*-*OH **14i**. Further hydrolysis proceeds more quickly at the *N-*formyl group than the β-amide moiety, and thus Asp-OH **14k** was observed with only traces of FoAsp-OH **12k**. These results show that when exposed to alkaline conditions, *N-*formylaminonitriles undergo hydration at the α-nitrile position fastest and provide mixtures of *N-*formylamino amides/acids and amino amides/acids en route to the terminal products of hydration and hydrolysis, amino acids. Such mixtures may have been advantageous for prebiotic peptide synthesis, providing N-terminal protection from the major pathway of peptide degradation^26^ (diketopiperazine formation), which is indeed the strategy used for bacterial peptide biosynthesis.^27^ The investigation of hydrolytic processing in the presence of various potential prebiotic catalysts is ongoing.

**Table 8:**
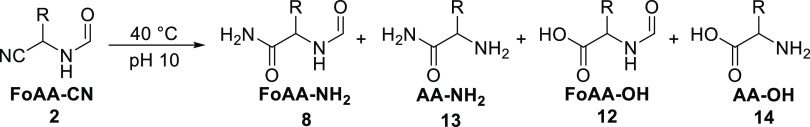
Partial Hydrolysis Outcomes of *N-*Formylaminonitriles

			hydrolysis outcome					
entry	R group (FoAA-CN **2**)	time (days)	FoAA-CN (**2**)	FoAA-NH_2_ (**8**)	FoAA-OH (**12**)	AA-CN (**1**)	AA-NH_2_ (**13**)	AA-OH (**14**)
1	H (FoGly-CN **2a**)	14	1%	30%	32%	2%	16%	12%
2	Me (FoAla-CN **2b**)	11	∼15%	∼30%	∼30%	∼2%	∼5%	∼2%
3	CH_2_OH (FoSer-CN **2d**)	10	3%	41%	22%		23%	
4	CH_2_CN (Fo(β-CN)Ala-CN **2h**)	11	FoAsn-OH **12i** 54% Fo(β-CN)Ala-OH **12h** 25%		FoAsp-OH **12k** trace		Asn-OH **14i** 6% (β-CN)Ala-OH **14h** 3%	

## Conclusions

In summary, we have investigated the formation and subsequent reactions of aminonitriles and *N-*formylaminonitriles in formamide, a medium of prebiotic relevance. We found that *N-*formylaminonitriles are produced by thermally promoted reactions between aldehydes and cyanide in formamide with or without added ammonia. In the case of glycolaldehyde, reaction with cyanide in formamide generated not only FoSer-CN **2d** but also the elimination product FoDHA-CN **2** and the formylated α,β-diaminoacid **3**. This facile synthesis of a dehydroalanine derivative significantly bolsters the prebiotic plausibility of such compounds, which are precursors to cysteine derivatives that have important catalytic properties in the context of prebiotic peptide synthesis. FoDHA-CN was also shown to react with a variety of other compounds of prebiotic interest, generating precursors of Asp and Asn and an imidazole derivative by an unexpected cascade reaction. The contrasting reactivities of FoDHA-CN in water and formamide suggest that prebiotic access to some compounds from dehydroalanine derivatives may have required formamide as a solvent, which follows naturally from the synthesis of FoDHA-CN in formamide. Finally, the alkaline processing of *N-*formylaminonitriles was shown to proceed by hydration of the nitrile group, preventing reversion of the Strecker equilibrium, which would occur upon deformylation,^10,14^ and generating mixtures of N-formylated and unformylated amino amides and acids prior to their complete hydrolysis to amino acids. Thus, *N-*formylaminonitrile formation from cyanide and aldehydes in formamide, and the subsequent reactivity of FoDHA-CN, may have been integral to the provision of chemical building blocks for peptide synthesis and other processes at the origin of life.

## Experimental Section

All experimental details, as well as characterization data and spectra collected on all compounds, are available in the Supporting Information.

## Supporting Information Available

Methods, supporting data, and NMR spectra (PDF)
