# Supplementary material for: Prebiotic Synthesis of N-Formylaminonitriles and Derivatives in Formamide
Source: J Am Chem Soc. 2023 May 5;145(19):10533–41. doi: 10.1021/jacs.2c13306 (PMC10197134; doi:10.1021/jacs.2c13306)
Supplement: Supplementary file 1 — ja2c13306_si_001.pdf [file ja2c13306_si_001.pdf]

Prebiotic Synthesis of *N*-formylaminonitriles and Derivatives in Formamide

Nicholas J. Green[a,b]*, David A. Russell[a], Sasha H. Tanner[b], John D. Sutherland[a]*

[a] MRC Laboratory of Molecular Biology, Francis Crick Avenue, Cambridge Biomedical Campus, Cambridge, CB2 0QH, UK.
[b] Department of Chemistry, University of Otago, Dunedin 9054, New Zealand.
**Correspondence to: johns@mrc-lmb.cam.ac.uk; nick.green@otago.ac.nz*

This PDF file includes:

Materials and Methods
References
Supplementary Figures

Materials and Methods

General Methods

Reagents and deuterated solvents used for reactions were purchased from Sigma-Aldrich or Acros Organics and were used without further purification. A Mettler Toledo SevenEasy pH Meter S20 was used to monitor the pH, and deoxygenation of solution was achieved by sparging anhydrous argon through the solution for 15-20 min. ^{1}H and ^{13}C NMR spectra were acquired using a Bruker Ultrashield 400 Plus operating at 400.1 MHz and 100.6 MHz respectively. Samples consisting of H_2O/D_2O mixtures were analysed using HOD suppression to collect ^{1}H NMR data. Chemical shifts (δ) are shown in ppm. Coupling constants (J) are given in Hertz (Hz) and the notations s, d, m represent the multiplicities singlet, doublet, and multiplet signal. For reactions in water with yields measured by internal standard, an NMR tube insert containing a 0.05% wt solution of 3-(Trimethylsilyl)propionic-2,2,3,3-d_4 acid sodium salt in D_2O was added to the NMR tube. 3-(Trimethylsilyl)propionic-2,2,3,3-d_4 was referenced to 0.015 ppm. For reactions in formamide, an NMR tube insert was added containing a 0.03% (v/v) solution of TMS in $CDCl_3$. TMS was referenced to 0 ppm. Reactions in formamide were conducted in an open vessel exposed to air and moisture, and formamide was not stored in an airtight container – all spectra collected in formamide contain a significant water peak, of the order of 5 mol%. Where reactions were conducted using hydrosulfide salts in water, the water and pHing solutions were purged with argon for ten minutes prior to their use. Mass spectra were recorded with an Agilent Technologies 6130 Quadrupole LC-MS using positive and negative Electron Spray Ionisation (ESI).

Formation of (N-formyl)aminonitriles in formamide

The relevant aminonitrile (0.030 mmol) was dissolved in formamide (0.30 mL, 0.1M). The mixture was then heated in an open NMR tube and the reaction progress monitored by NMR spectroscopy. For reactions with $MgCl_2$ added, 5.0 equiv. of $MgCl_2$ (0.150 mmol, 14.3 mg) was added at the commencement of the reaction.

Formation of serine nitrile free base, SerCN

SerCN was prepared by mixing glycolaldehyde (0.06 mmol, 1.0 eq.), ammonium chloride (0.30 mmol, 5.0 eq.) and sodium cyanide (0.072 mmol, 1.2 eq.) were dissolved in 9:1 H_2O/D_2O (0.6 mL, 1 M) and the pH adjusted to 9.2. After 48 h, the solution was lyophilised, and the residue dissolved in formamide for formylation reactions as above.

Strecker reactions in formamide

The relevant aldehyde (0.040 mmol), sodium cyanide (5.9 mg, 0.12 mmol, 3.0 eq.) and ammonium chloride (10.7 mg, 0.20 mmol, 5.0 eq) were was dissolved in formamide (0.40 mL, 0.1M). The mixture was then heated in an open NMR tube and the reaction progress monitored by NMR spectroscopy.

Reactions of aldehydes, sodium cyanide and formic acid in formamide

The relevant aldehyde (0.040 mmol), sodium cyanide (9.8 mg, 0.20 mmol, 5.0 eq.) and formic acid (9.0 mg, 0.20 mmol, 5.0 eq) were dissolved in formamide (0.40 mL, 0.1M). Various additives were included at this point. The mixture was then heated in an open NMR tube and the reaction progress monitored by NMR spectroscopy.

Preparation of the hydrochloride salts of serine nitrile and valine nitrile, SerCN·HCl and ValCN·HCl

The hydrochloride salts of serine nitrile and valine nitrile were prepared by method of Paventi and Edward[1]. The relevant aldehyde (2.0 mmol, 1.0 eq.) was added to a mixture of 28% ammonium hydroxide solution (275 μL) and tetrahydrofuran (1.2 mL). The mixture was stirred for 10 min before ammonium chloride (107 mg, 2.0 mmol, 1.0 eq.) was added followed by sodium cyanide (98 mg, 2.0 mmol, 1.0 eq.). The biphasic mixture was stirred vigorously for 2 h before sodium sulfate (approx. 4 g) was added followed by diethyl ether (20 mL). The slurry was stirred for 0.5 h. The solution was decanted from the solids, which were washed with diethyl ether (3 x 10 mL) and the combined solution was concentrated to approx. 4 mL under a stream of nitrogen. To precipitate the hydrochloride salt of the amino acid nitrile the solution was cooled to 0 ºC and 2.0 M HCl in diethyl ether (approx. 1 mL) was added dropwise. The resulting precipitate was collected by filtration, washed with diethyl ether, and dried under a gentle stream of nitrogen. Valine nitrile hydrochloride was obtained as a white solid (135 mg, 1.00 mmol, 50% yield). Serine nitrile hydrochloride was obtained as a white solid (66 mg, 0.54 mmol, 27% yield).

Valine nitrile hydrochloride, ValCN·HCl

^{1}H NMR (400 MHz, D_2O): δ 4.36 (d, 1H, *J* = 5.9 Hz, HCCN), 2.25 (m, 1H, $HC(CH_3)_2$), 1.08 (d, 3H, *J* = 7.0 Hz, CH_3), 1.07 (d, 3H, *J* = 7.0 Hz, CH_3).

^{13}C NMR (100 MHz, D_2O): δ 115.2 (CN), 47.6 (CHCN), 29.8 ($CH(CH_3)_2$), 18.0 (CH_3), 16.5 (CH_3).

MS (LCMS ESI+): 99.1

Serine nitrile hydrochloride, SerCN·HCl

^{1}H NMR (D_2O): δ 4.5 (t, 1H, *J* = 4.4 Hz, HCCN), 3.98 (dd, 1H, *J* = 12.4, 4.6 Hz, CH_aH_b), 3.92 (dd, 1H, *J* = 12.4, 4.4 Hz, CH_aH_b).

^{13}C NMR (100 MHz, D_2O): δ 115. 4 (CN), 59.6 (CH_2), 43.5 (CH).

MS (LCMS ESI+): 87.1

Preparation of (N-formyl)serine nitrile, FoSerCN, starting from glycolaldehyde

Glycolaldehyde (0.100 g, 1.67 mmol, 1.0 eq.), ammonium chloride (445 g, 8.4 mmol, 5.0 eq.) and sodium cyanide (0.100 g, 2.0 mmol, 1.2 eq.) were dissolved in 9:1 H_2O/D_2O (3.33 mL, 0.5 M) and the pH adjusted to 9.2. After 40 h stirring, formic acid (0.317 mL, 8.4 mmol, 5.0 eq.) was added and the pH adjusted to 5.0. EDCI (480 mg, 3.09 mmol, 1.5 eq.) was added. After 2h, the mixture was passed through Dowex resin (Na+ form) and the fraction containing FoSerCN lyophilised to provide the target material (136 mg, 1.19 mmol, 71% yield).

(N-formyl)serinenitrile, FoSer-CN

^{1}H NMR (400 MHz, D_2O): δ 8.22 (s, 1H, HCO), 5.03 (t, 1H, *J* = 5.2 Hz, HCCN), 3.96 (dd, 1H, *J* = 11.7, 5.2 Hz, CH_aH_b), 3.91 (dd, 1H, *J* = 11.7, 5.2 Hz, CH_aH_b).

Minor conformer peaks (from restricted formyl C–N rotation) visible: δ 8.18 (s, 1H), 4.90 (t, *J* = 5.5 Hz, 1H).

^{13}C NMR (100 MHz, D_2O) δ 163.9 (CHO), 117.44 (CN), 60.54 (CH_2), 41.60 (CH).

Minor conformer peaks (from restricted formyl C–N rotation) visible: δ 167.2, 117.3, 62.0, and 46.0.

MS (LCMS ESI-): 113.0

In formamide, $CDCl_3$ insert:

^{1}H NMR (400 MHz, $CDCl_3$) δ 8.82 (s, 1H), 5.58 (dd, 1H, *J* = 5.2, 4.9 Hz), 4.50 (dd, 1H, *J* = 11.2, 4.9 Hz), 4.45 (dd, 1H, *J* = 11.4, 5.3 Hz).

^{13}C NMR (100 MHz, $CDCl_3$) δ 166.5 (CHO), 121.7 (CN), 64.4 (CH_2), 45.3 (CH).

Preparation of (N-formyl)amino nitriles

EDCI (38.4 mg, 0.2 mmol, 2.0 eq.) was added to a solution of the amino nitrile hydrochloride (0.1 mmol, 1.0 eq.) and formic acid (18.9 μL, 0.5 mmol, 5.0 eq.) in water (1.0 mL) at pH 5.0. The mixture was allowed to stand at room temperature overnight or until the reaction had gone to completion as determined by ^{1}H NMR spectroscopy. The mixture was then passed through a column of Dowex resin (Na+ form), eluting with water. The fraction containing the desired product was lyophilised, the solid was extracted with ethyl acetate and the combined extracts were concentrated under reduced pressure to give the (N-formyl)amino nitrile as a white solid in quantitative yield.

(N-formyl)glycine nitrile, FoGly-CN

^{1}H NMR (400 MHz, D_2O) δ 8.19 (s, 2H, HCO), 4.26 (s, 1H, H_2CCN, conformer 1) 4.25 (s, 1H, H_2CCN, conformer 2). This is consistent with two equally populated rotamers.

^{13}C NMR (100 MHz, D_2O) δ 164.7 (CHO), 116.9 (CN), 26.3 (CH_2).

In formamide, $CDCl_3$ insert:

^{1}H NMR (400 MHz, $CDCl_3$) δ formyl signal(s) obscured by solvent, 4.86 (s, 1H, H_2CCN, conformer 1), 4.85 (s, 1H, H_2CCN, conformer 2).

^{13}C NMR (100 MHz, $CDCl_3$) δ 166.9 (CHO), 120.3 (CN), 29.0 (CH_2)

(N-formyl)alaninenitrile, FoAla-CN:

^{1}H NMR (400 MHz, D_2O) δ 8.12 (s, 1H, HCO), 4.86 (q, 1H, J = 7.2 Hz, HCCN), 1.59 (d, 3H, J = 7.2 Hz, CH_3).

Minor conformer signals (from restricted formyl C–N rotation) visible: δ 8.16 (s), 1.42 (d, 7.2 Hz)

^{13}C NMR (100MHz, D_2O) δ 163.8 (CHO), 119.7 (CN), 35.1 (CH), 17.3 (CH_3).

Minor conformer signals (from restricted formyl C–N rotation) visible: δ 166.9, 39.5, and 19.5.

MS (LCMS ESI-): 97.1

In formamide, $CDCl_3$ insert:

^{1}H NMR (400 MHz, $CDCl_3$) δ 8.69 (s, 1H, HCO) 5.42 (app quint, 1H, J = 7.1 Hz, HCCN), 2.11 (d, 3H, J = 7.2 Hz, CH_3).

^{13}C NMR (100 MHz, $CDCl_3$) δ 166.0 (CHO), 123.0 (CN), 37.9 (CH), 20.8 (CH_3)

(N-formyl)valine nitrile, FoVal-CN

^{1}H NMR (400 MHz, D_2O) δ 8.09 (s, 1H, HCO), 4.65 (dd, 1H, J = 6.7, 0.9 Hz, HCCN), 2.09 (m, 1H, $HC(CH_3)_2$), 1.01 (d, 3H, J = 6.8 Hz, CH_3), 0.98 (d, 3H, J = 6.8 Hz, CH_3);

Minor conformer signals (from restricted formyl C–N rotation) visible: δ 8.04 (s) and 4.50 (d, J = 6.9 Hz).

^{13}C NMR (100MHz, D_2O) δ 163.4 (CHO), 118.4 (CN), 45.7 (CHCN), 30.6 ($CH(CH_3)_2$), 17.7 (CH_3), 17.5 (CH_3);

Minor conformer signals (from restricted formyl C–N rotation) visible: δ 167.3, 50.3, 32.1, 17.4, and 17.3.

MS (LCMS ESI-): 125.1

In formamide, $CDCl_3$ insert:

^{1}H NMR (400 MHz, $CDCl_3$) δ 8.76 (s, 1H, HCO), 5.23 (t, 1H, J = 7.2 Hz, HCCN), 2.60 (app octet, 1H, J = 6.7 Hz, $HC(CH_3)_2$), 1.55 (d, 3H, J = 6.7 Hz, CH_3), 1.51 (d, 3H, J = 6.8 Hz, CH_3).

^{13}C NMR (100 MHz, $CDCl_3$) δ 166.0 (CHO), 121.5, (CN), 48.5 (CHCN) 33.7 ($CH(CH_3)_2$), 21.3 (CH_3), 20.9 (CH_3)

(N-formyl)aspartic acid dinitrile, Fo(β-CN)Ala-CN

^{1}H NMR (400 MHz, D_2O): δ 8.15 (s, 1H, HCO), 5.26 (t, 1H, J = 6.7 Hz, HCCN), 3.23 (dd, 1H, J = 17.2, 7.1 Hz, CH_aH_bCN), 3.19 (dd, 1H, J = 17.2, 6.4 Hz, CH_aH_bCN).

^{13}C NMR (100MHz, D_2O): δ 163.8 (CHO), 116.4 (CN), 116.3 (CN), 36.3 (CHCN), 21.5 (CH_2CN).

MS (LCMS ESI-): 122.1

In formamide, $CDCl_3$ insert:

^{1}H NMR (400 MHz, $CDCl_3$) δ 8.80 (s, 1H), 5.89 (t, 1H, J = 7.0 Hz, HCCN), 3.87 – 3.76 (m, 2H, CH_aH_bCN and CH_aH_bCN).

^{13}C NMR (100 MHz, $CDCl_3$) δ 163.5 (CHO), 118.4 (CN), 117.5 (CN), 36.8 (CHCN), 21.8 (CH_2CN).

Preparation of (N-formyl)-dehydroalanine nitrile, FoDHA-CN, from glycolaldehyde

Glycolaldehyde (0.600 g, 10 mmol, 1.0 eq.), ammonium chloride (2.65 g, 50 mmol, 5.0 eq.) and sodium cyanide (0.588 g, 12 mmol, 1.2 eq.) were dissolved in 9:1 H_2O/D_2O (10 mL, 1 M) and the pH adjusted to 9.2. After 16 h stirring, the mixture was concentrated under vacuum and triturated with methanol. The mother liquor was concentrated once more and triturated with methanol. The mother liquor was concentrated, then dissolved in 9:1 H_2O/D_2O (20 mL, 0.5 M), cooled with an ice bath, and formic acid (1.14 mL, 30 mmol, 3.0 eq.) added. The pH of the solution was adjusted to 4.5 using 5M NaOH, and then EDCI (2.3 g, 15 mmol, 1.5 eq.) was added. After 4h, the pH was adjusted to 4.5 and an additional portion of EDCI was added (1.5 g, 10 mmol, 1.0 eq.). The reaction was stirred overnight at room temperature then filtered through Dowex resin (Na+ form). The residue was concentrated to 20 mL and the pH adjusted to 7.0 before N-acetyl imidazole (9.0 g, 82 mmol, 8.2 eq.) was added. After 4 h the solution was lyophilized, then the remainder triturated with 1:1 EtOAc/MeOH. The supernatant was

concentrated and then purified by normal flash chromatography (8:1 EtOAc/pentane) to provide (N-formyl)-O-acetyl-serine nitrile, FoSer(Ac)-CN, (0.91 g, 5.8 mmol, 58% yield).

FoSer(Ac)-CN (0.91 g, 5.8 mmol, 1.0 eq.) was dissolved in water (58 mL, 0.1M) and the pH of the solution periodically adjusted to 9 over sixteen hours, until the pH was constant. Consumption of starting material was verified by extracting an aliquot (0.45 mL), adding D_2O (0.05 mL), and obtaining a ^{1}H NMR spectrum. The pH was then adjusted to 7, and the mixture lyophilised. The residue was subjected to reverse phase flash chromatography (0–15 % MeCN in H_2O) to provide FoDHACN (0.31 g, 3.2 mmol, 55% yield).

FoSer(Ac)-CN
^{1}H NMR (400 MHz, D_2O) δ 8.12 (s, 1H, $\underline{H}$CO), 5.21 (t, 1H, *J* = 5.0 Hz, $\underline{H}$CCN), 4.42 (dd, 1H, *J* = 11.5, 5.0 Hz, $C\underline{H_a}H_b$), 4.35 (dd, 1H, *J* = 11.5, 5.0 Hz, $CH_a\underline{H_b}$), 2.11 (s, 3H, $C\underline{H_3}$).
^{13}C NMR (100MHz, D_2O) δ 173.2 ($\underline{C}OCH_3$), 163.8 ($\underline{C}$HO), 116.5 ($\underline{C}$N), 62.5 ($\underline{C}H_2OCOCH_3$), 38.7 ($\underline{C}$HCN), 20.0 ($\underline{C}H_3$).
MS (LCMS ESI): 155.1

FoDHA-CN
^{1}H NMR (400 MHz, D_2O) δ 8.20 (s, 1H, $\underline{H}$CO), 6.06 (d, 1H, *J* = 1.9 Hz, $C\underline{H_a}H_b$), 5.87 (m, 1H, $CH_a\underline{H_b}$),
Minor conformer peaks (from restricted formyl C–N rotation) visible: δ 8.57 (s, 1H), 5.72 (d, 1H, *J* = 2.5 Hz), 5.65 (d, 1H, *J* = 2.5 Hz).
^{13}C NMR (100MHz, D_2O) δ 162.3 ($\underline{C}$HO), 121.5 ($\underline{C}$H), 115.1 ($\underline{C}$N), 113.7 ($\underline{C}$).
Minor conformer peaks (from restricted formyl C–N rotation) visible: δ 164.8, 115.9, 115.7, and 114.0.
MS (LCMS ESI-): 95.0

In formamide, $CDCl_3$ insert:
^{1}H NMR (400 MHz, $CDCl_3$) δ 8.81 (s, 1H, $\underline{H}$CO), 6.60 (d, 1H, *J* = 1.5 Hz, $C\underline{H_a}H_b$), 6.26 (s, 1H, $CH_a\underline{H_b}$);
Minor conformer peaks (from restricted formyl C–N rotation) visible: δ 8.81 (s), 6.19 (d, *J* = 2.2 Hz), 6.07 (d, *J* = 2.2 Hz).
^{13}C NMR (100 MHz, $CDCl_3$) δ 164.3, 122.0 ($\underline{C}$HO), 118.6 ($\underline{C}$N), 117.9 ($\underline{C}H_2$);
Minor conformer peaks (from restricted formyl C–N rotation) visible: δ 166.7, 119.6, 117.4, and 117.1.

Fo(FoNH)Ala-CN
In formamide, $CDCl_3$ insert:
^{1}H NMR (400 MHz, $CDCl_3$) δ 8.80-8.78 (m, 2H, 2 x $\underline{H}$CO) 5.65 (s, 1H, $\underline{H}$CCN), 4.38-4.19 (m, 2H, $C\underline{H_a}H_b$ and $CH_a\underline{H_b}$).
^{13}C NMR (100 MHz, $CDCl_3$) δ 167.1 ($\underline{C}$HO), 166.2 ($\underline{C}$HO), 120.9 ($\underline{C}$N), 42.6 ($\underline{C}$HNHFo), 41.9 ($\underline{C}H_2$NHFo).
MS (LCMS ESI+): 142.0

Preparation of aspartic acid dinitrile free base, (β-CN)Ala-CN:
Aspartic acid dinitrile was prepared by the method of Ksander et al[2]. 28% Ammonium hydroxide solution (0.9 mL) was added to a solution of fumaronitrile (156 mg, 2.0 mmol) in 1,4-dioxane (3.7 mL). The mixture was stirred at room temperature for 24 hours. The almost black mixture was diluted with ethyl acetate and the mixture was added slowly to a column of neutral alumina, eluting with 1:1 dichloromethane/ethyl acetate. The product was obtained as a yellow oil, homogenous by TLC (SiO_2, R_f 0.3, 1:1 CH_2Cl_2/EtOAc) and ^{1}H NMR spectroscopy (120 mg, 1.26 mmol, 63% yield).
^{1}H NMR (D_2O): δ 4.25 (t, 1H, *J* = 6.4 Hz, $C\underline{H}$CN), 3.01 (d, 2H, *J* = 6.4 Hz, $C\underline{H_2}$CN);
^{13}C NMR (D_2O): δ 120.0 ($CH_2\underline{C}N$), 117.2 ($CH\underline{C}N$), 39.8 ($\underline{C}$HCN), 23.8 ($\underline{C}H_2$CN); NMR spectroscopic data collected in CD_3CN were in good agreement with those reported by Ksander et al.

Preparation of (N-formyl)amino amides
EDCI (38.4 mg, 0.2 mmol, 2.0 eq.) was added to a solution of the amino amide hydrochloride (0.1 mmol, 1.0 eq.) and formic acid (18.9 μL, 0.5 mmol, 5.0 eq.) in water (1.0 mL) at pH 5.0. The mixture was allowed to stand at room temperature overnight or until the reaction had gone to completion as determined by ^{1}H NMR spectroscopy. The mixture was then passed through a column of Dowex resin (Na+ form, 200-400 mesh), eluting with water. The fraction containing the desired product was lyophilised, the solid was extracted with ethyl acetate and the combined extracts were concentrated under reduced pressure to give the (N-formyl)amino amide as a white solid in quantitative yield.

(N-formyl)glycinamide, FoGly-NH_2
^{1}H NMR (400 MHz, D_2O): δ 8.12 (s, 1H, $\underline{H}$CO), 3.91 (s, 2H, $C\underline{H_2}CONH_2$);
Minor conformer peaks (from restricted formyl C–N rotation) visible: δ 3.98 (s).
^{13}C NMR (100MHz, D_2O): δ 173.9 ($\underline{C}ONH_2$), 165.0 ($\underline{C}$HO), 40.8 ($\underline{C}H_2CONH_2$); unique signals for the minor conformer (from restricted formyl C–N rotation) peaks visible: δ 168.5 and 44.3.

(N-formyl)alaninamide, FoAla-NH_2
^{1}H NMR (400 MHz, D_2O): δ 8.03 (s, 1H, $\underline{H}$CO), 4.32 (dq, 1H, *J* = 7.3, 1.0 Hz, $\underline{H}CONH_2$), 1.35 (d, 3H, *J* = 7.3 Hz, $C\underline{H_3}$);
Minor conformer peaks (from restricted formyl C–N rotation) visible: δ 7.99 (s), 4.26 (q, *J* = 7.2 Hz), and 1.38 (d, *J* = 7.2 Hz).
^{13}C NMR (100MHz, D_2O): δ 177.6 ($\underline{C}ONH_2$), 164.1 ($\underline{C}$HO), 48.2 ($\underline{C}HCONH_2$), 17.0 ($\underline{C}H_3$);

Minor conformer peaks (from restricted formyl C–N rotation) visible: δ 167.3, 51.5, and 18.2.

(N-formyl)serinamide, FoSer-NH_2
^{1}H NMR (400 MHz, D_2O): δ 8.13 (s, 1H, $\underline{H}CO$), 4.45 (dt, 1H, *J* = 1.0, 4.8 Hz, $\underline{H}CCH_2$), 3.85 (dd, 1H, *J* = 11.7, 5.2 Hz, $C\underline{H}_aH_bOH$), 3.80 (dd, 1H, *J* = 11.7 4.5 Hz, $CH_a\underline{H}_bOH$);
Minor conformer peaks (from restricted formyl C–N rotation) visible: δ 4.29 (dd, *J* = 6.1, 4.6 Hz).
^{13}C NMR (100MHz, D_2O): δ 174.2 ($\underline{C}ONH_2$), 164.5 ($\underline{C}HO$), 61.3 ($\underline{C}H_2OH$), 53.9 ($\underline{C}HCONH_2$).

(N-formyl)-β-cyanoalaninamide, Fo(β-CN)Ala-NH_2
^{1}H NMR (400 MHz, D_2O) δ 8.24 (s, 1H), 4.89 (ddd, 1H, *J* = 7.4, 5.6, 0.8 Hz), 3.13 (dd, 1H, *J* = 17.3, 5.6 Hz), 3.06 (dd, 1H, *J* = 17.2, 7.3 Hz).
^{13}C NMR (100MHz, D_2O) δ 172.6 ($\underline{C}ONH_2$), 164.3 ($\underline{C}HO$), 118.0 ($\underline{C}N$), 48.0 ($\underline{C}H$), 20.2 ($\underline{C}H_2$).
MS (LCMS ESI+): 142.0

(N-formyl)-asparaginamide, FoAsnNH_2
^{1}H NMR (400 MHz, D_2O): δ 8.08 (s, 1H, $\underline{H}CO$), 2.78 (dd, 1H, *J* = 15.8, 5.5 Hz, $C\underline{H}_aH_bCONH_2$), 2.71 (dd, 1H, *J* = 15.8, 7.7 Hz, $CH_a\underline{H}_bCONH_2$); the $\underline{H}CCONH_2$ signal for the major conformer (δ 4.72, assigned by HSQC) is obscured by the HOD peak;
Minor conformer peaks (from restricted formyl C–N rotation) visible: δ 7.99 (s), 4.53 (dd, *J* = 15.8, 4.9 Hz), and 2.68 (dd, *J* = 15.8, 8.8 Hz).
^{13}C NMR (100MHz, D_2O): δ 175.0 ($\underline{C}ONH_2$), 174.6 ($\underline{C}ONH_2$), 164.2 ($\underline{C}HO$), 48.9 ($\underline{C}HCONH_2$), 36.6 ($\underline{C}H_2CONH_2$);
Minor conformer peaks (from restricted formyl C–N rotation) visible: δ 167.5, 52.8, and 37.6.

In formamide, $CDCl_3$ insert:
^{1}H NMR (400 MHz, $CDCl_3$) δ 8.72 (s, 1H), 5.41 (dd, 1H, *J* = 8.9, 5.5 Hz), 3.62 (dd, 1H, *J* = 17.3, 9.0 Hz), 3.24 (dd, 1H, *J* = 17.3, 5.5 Hz).
^{13}C NMR (100 MHz, $CDCl_3$) δ 182.9 (β-$\underline{C}ONH_2$), 180.5 ($\underline{C}ONH_2$), 164.0 ($\underline{C}HO$), 50.6 ($\underline{C}HCONH_2$), 37.3 ($\underline{C}H_2CONH_2$).
LCMS: ESI+ 160.1, ESI- 158.1

<u>Preparation of (N-formyl)amino acids</u>:
(N-formyl)amino acids were prepared by the method of Sheehan and Yang[3]. Acetic anhydride (350 μL) was added to a solution of the relevant amino acid (1.0 mmol) in formic acid (1.0 mL) at 60 ^{O}C. The mixture was then stirred at room temperature for 1 hour before water (4 mL) was added and the mixture allowed to evaporate overnight at room temperature in a crystallising dish. The crystalline residue was washed with diethyl ether (3 x 4 mL) and the solid dried in air to afford the pure (N-formyl)amino acid product. The (N-formyl)serine obtained by this method was, however, contaminated with acetylated material and pure (N-formyl)serine, in the form of small white crystals, was obtained only after two recrystallisations from ethyl acetate (56 mg, 0.49 mmol, 49% yield).

(N-formyl)serine, FoSer-OH
^{1}H NMR (400 MHz, D_2O): δ 8.38 (br s, 1H, $N\underline{H}$), 8.11 (s, 1H, $\underline{H}CO$), 4.54 (m, 1H, $\underline{H}CCO_2H$), 3.92 (dd, 1H, *J* = 11.7, 4.8 Hz, $C\underline{H}_aH_bOH$), 3.83 (dd, 1H, *J* = 11.7, 3.8 Hz, $CH_a\underline{H}_bOH$);
Minor conformer peaks (from restricted formyl C–N rotation) visible: δ 4.32 (m).
^{13}C NMR (100MHz, D_2O): δ 173.4 ($\underline{C}O_2H$), 164.3 ($\underline{C}HO$), 61.3 ($\underline{C}H_2OH$), 53.7 ($\underline{C}HCO_2H$); Minor conformer peaks visible: *δ* 173.6, 167.7, 62.4, and 57.6.

(N-formyl)-β-cyanoalanine
^{1}H NMR (400 MHz, D_2O): δ 8.69 (br s, 1H, $N\underline{H}$), 8.13 (s, 1H, $\underline{H}CO$), 4.77 (m, 1H, partially obscured by the HOD peak, $\underline{H}CCO_2H$), 3.08 (dd, 1H, *J* = 17.2, 5.5 Hz, $C\underline{H}_aH_bCN$), 3.00 (dd, 1H, *J* = 17.3, 7.2 Hz, $CH_a\underline{H}_bCN$).
^{13}C NMR (100MHz, D_2O): δ 172.2 ($\underline{C}O_2H$), 164.2 ($\underline{C}HO$), 118.2 ($\underline{C}N$), 48.0 ($\underline{C}HCO_2H$), 20.3 ($\underline{C}H_2CN$).

(N-formyl)asparagine, FoAsn-OH
^{1}H NMR (400 MHz, D_2O): δ 8.38 (br s, 1H, $N\underline{H}$), 8.06 (s, 1H, $\underline{H}CO$), 2.81 (app d, 2H, *J* = 6.0 Hz, $C\underline{H}_2CONH_2$); the $\underline{H}CCO_2H$ signal for the major conformer (from restricted formyl C–N rotation) (δ 4.74, assigned by HSQC) is obscured by the HOD peak;
Minor conformer peaks (from restricted formyl C–N rotation) visible: δ 7.99 (s) and 4.52 (m).
^{13}C NMR (100MHz, D_2O): δ 174.9 ($\underline{C}ONH_2$), 174.3 ($\underline{C}O_2H$), 164.0 ($\underline{C}HO$), 48.4 ($\underline{C}HCO_2H$), 36.6 ($\underline{C}H_2CONH_2$);
Minor conformer peaks visible: δ 167.5, 52.6, and 37.8.

(N-formyl)aspartic acid, FoAsp-OH
^{1}H NMR (400 MHz, D_2O): δ 8.46 (br s, 1H, $N\underline{H}$), 8.06 (s, 1H, $\underline{H}CO$), 4.77 (m, 1H, partially obscured by the HOD peak, $\underline{H}CO_2H$), 2.94 (dd, 1H, *J* = 17.4, 6.2 Hz, $C\underline{H}_aH_bCO_2H$), 2.88 (dd, 1H, *J* = 17.4, 5.0 Hz, $CH_a\underline{H}_bCO_2H$);
Minor conformer peaks (from restricted formyl C–N rotation) visible: δ 7.99 (s) and 4.58 (m).
^{13}C NMR (D_2O): δ 174.5 ($\underline{C}O_2H$), 174.1 ($\underline{C}O_2H$), 164.0 ($\underline{C}HO$), 47.9 ($\underline{C}H$), 35.9 ($\underline{C}H_2$);

Minor conformer peaks (from restricted formyl C–N rotation) visible: δ 167.8, 52.2, and 37.0.

Preparation of β-cyanoalanine methyl ester

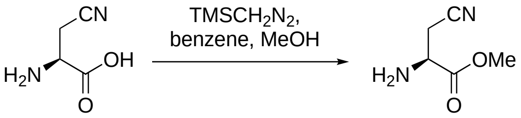

β-Cyanoalanine methyl ester was prepared by adapting the method of Chan et al[4]. 2M TMS-diazomethane in ether (1.5 mL, 3.0 mmol, 8.5 eq.) was added dropwise to a stirred suspension of β-cyanoalanine (40 mg, 0.35 mmol, 1.0 eq.) in benzene (5.6 mL) and methanol (1.6 mL). The mixture was stirred overnight at room temperature and then acetic acid (0.1 mL) was added dropwise and the reaction stirred for two hours. The mixture was concentrated and then triturated with ethyl acetate, which precipitated remaining starting material. The mother liquor was purified by flash chromatography (20:79:1 pentane/ethyl acetate/triethylamine) to provide β-cyanoalanine methyl ester (32 mg, 0.25 mmol, 71 %).
^{1}H NMR (400 MHz, methanol-d_4) δ 3.80 (dd, 1H, *J* = 6.4, 5.8 Hz, HCO_2CH_3), 3.77 (s, 3H, CH_3), 2.85 (dd, 1H, *J* = 17.0, 6.0 Hz, CH_aH_bCN), 2.79 (dd, 1H, *J* = 17.0, 6.0 Hz, CH_aH_bCN);
^{13}C NMR (100 MHz, methanol-d_4) δ 172.5 (CO_2CH_3), 117.0 (CN), 51.0 (CH_3), 50.2 ($CHCO_2CH_3$), 21.6 (CH_2CN).

Preparation of β-cyanoalaninamide

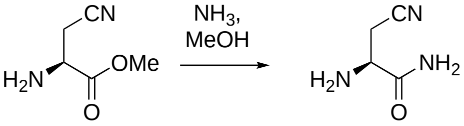

β-Cyanoalanine methyl ester (16 mg, 0.12 mmol, 1.0 eq.) was dissolved in MeOH (1 mL) and to this stirred solution was added, dropwise, 7M methanolic ammonia solution (1.0 mL). The mixture was stirred for 16 hours, then concentrated under vacuum, redissolved in MeOH, and concentrated under vacuum again to yield β-cyanoalaninamide (13 mg, 0.12 mmol, quant.).
^{1}H NMR (methanol-d_4) δ 3.63 (dd, 1H, *J* = 6.6, 5.6 Hz, $HCONH_2$), 2.82 (dd, 1H, *J* = 16.8, 5.6 Hz, CH_aH_bCN), 2.73 (dd, 1H, *J* = 16.8, 6.6 Hz, CH_aH_bCN);
^{13}C NMR (methanol-d_4) δ 175.7 ($CONH_2$), 117.2 (CN), 51.0 ($CHCONH_2$), 22.7 (CH_2CN).

Reactions of FoDHA-CN with Nucleophiles

Reactions of FoDHA-CN and Ammonia

In water, ammonium chloride
FoDHA-CN (10 mg, 0.10 mmol, 1.0 eq.) and NH_4Cl (28 mg, 0.52 mmol, 5.0 eq.) were dissolved in H_2O (1 mL, 0.1 M). The pH was adjusted using 5M NaOH to 9.2. The reaction was left at room temperature in an NMR tube and monitored by ^{1}H NMR spectroscopy using an internal standard.

(β-FoNH)Ala-CN
^{1}H NMR (400 MHz, D_2O) δ 8.11 (s, 1H), 4.02 (t, 1H, *J* = 6.4 Hz), 3.52 (d, 2H, *J* = 6.4 Hz).
^{13}C NMR (100MHz, D_2O): δ 165.0 (CHO), 120.9 (CN), 42.9 (CHNHFo), 41.0 (CH_2NH_2).
MS (LCMS-ESI+): 114.1

Fo(β-NH$_2$)Ala-CN
^{1}H NMR (400 MHz, D_2O) δ 8.10 (s, 1H), 4.78 (obscured by water signal, visible by 2D NMR, 1H), 3.05 (dd, 1H, *J* = 13.5, 6.2 Hz), 3.00 (dd, 1H, *J* = 13.5, 6.9 Hz).
^{13}C NMR (100MHz, D_2O): δ 164.2 (CHO), 118.0 (CN), 42.3 (CH_2NH_2), 42.2 (CHNHFo)
MS (LCMS ESI+): 137.1, 114.1

In formamide, ammonium hydroxide
FoDHA-CN (3 mg, 0.026 mmol, 1.0 eq.) was dissolved in formamide (0.30 mL) and concentrated ammonium hydroxide added (0.13 mmol, 5.0 eq. 0.021 mL). The reaction was left open in an NMR tube at room temperature and monitored by ^{1}H NMR spectroscopy using an internal standard.

Fo(β-NH$_2$)Ala-CN
In formamide, $CDCl_3$ insert:
^{1}H NMR (400 MHz, $CDCl_3$) δ 8.78 (s, 1H), 5.41 (t, 1H, 6.4 Hz), 3.61 (d, 2H, 6.3 Hz)
^{13}C NMR (100 MHz, $CDCl_3$) δ 166.2 (CHO) 121.5 (CN), 46.0 (CH_2NH_2) 45.4 (CHNHFo)
MS (LCMS ESI+): 137.1, 114.1

(β-FoNH)Ala-CN
In formamide, $CDCl_3$ insert:

^{1}H NMR (400 MHz, $CDCl_3$) δ 8.77 (s, 1H), 4.59 (t, 1H, 6.5 Hz), 4.15-4.08 (m, 2H)
^{13}C NMR (100 MHz, $CDCl_3$) δ 166.8 (CHO), 124.7 (CN) 46.28 (CHNHFo) 44.33 (CH_2NH_2)
MS (LCMS ESI+): 137.1, 114.1

In formamide, ammonium formate
Fo-DHA-CN (3 mg, 0.026 mmol, 1.0 eq.) was dissolved in formamide (0.30 mL) and ammonium formate was added (8.2 mg, 0.13 mmol, 5.0 eq.). The reaction was left in an open NMR tube and heated to 50°C for 16 h, then 80 °C for 36h, and monitored by ^{1}H NMR spectroscopy using an internal standard.

Fo(β-FoNH)Ala-CN
As reported above in formamide.

Reactions of FoDHA-CN and Cyanide

In water, with sodium cyanide

FoDHA-CN (10 mg, 0.104 mmol, 1.0 eq.) and sodium cyanide (26 mg, 0.520 mmol, 5.0 eq.) were dissolved in H_2O (1.0 mL, 0.1 M). The pH was adjusted using 5M HCl to 9.2. The reaction was left at room temperature in an NMR tube and monitored by ^{1}H NMR spectroscopy using an internal standard.

In formamide, with sodium cyanide and formic acid

FoDHA-CN (2.9 mg, 30 μmol, 1.0 eq.), formic acid (4.14 mg, 90 μmol, 3.0 eq.) and sodium cyanide (4.4 mg, 90 μmol, 3.0 eq.) were dissolved in formamide (0.3 mL, 0.1 M). The reaction was left at room temperature in an open NMR tube and monitored by ^{1}H NMR spectroscopy using an internal standard.

In formamide, with sodium cyanide

FoDHA-CN (2.9 mg, 30 μmol, 1.0 eq.), formic acid (4.14 mg, 90 μmol, 3.0 eq.) and sodium cyanide (4.4 mg, 90 μmol, 3.0 eq.) were dissolved in formamide (0.3 mL, 0.1 M). The reaction was left at room temperature in an open NMR tube and monitored by ^{1}H NMR using an internal standard.

5-(cyanomethyl)-1H-imidazole-4-carbonitrile[5]

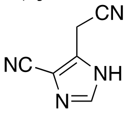

^{1}H NMR (400 MHz, D_2O) δ 7.52 (s, 1H), 4.05 (s, 2H).
^{13}C NMR (100 MHz, D_2O) δ 146.4 (CH), 139.1 (C), 118.4 (CN), 117.7 (CN), 106.7 (C), 16.1 (CH_2).
MS: (LCMS, ESI-): 131.1
Note: we found the chemical shift of peaks to vary with pH and concentration.

Several crude reactions were combined and filtered through a Sep-Pak column before performing preparative reverse phase HPLC, eluting with 0-50% MeCN in water. All fractions were collected and assayed by ^{1}H NMR, as the product was not easily detected by the UV detector. Fractions containing **10** were combined and concentrated to yield the pure material for characterisation in DMSO-d_6.

^{1}H NMR (400 MHz, DMSO-d_6) δ 7.11 (s, 1H), 3.84 (s, 2H).
^{13}C NMR (100 MHz, DMSO-d_6) δ 145.1 (CH), 137.5 (C), 119.0 (CN), 118.4 (CN), 106.4 (C), 16.2 (CH_2).

NMR spectroscopic data were in good agreement with those reported by Rousseau et al.[5]

Fo(β-CN)Ala-CN
As reported above in D_2O and formamide.

Fo(β-CN)Ala-NH_2
As reported above in D_2O.

(N-formyl)-asparaginamide, FoAsn-NH_2
As reported above in D_2O and formamide.

Reactions of FoDHA-CN and Imidazole

In water, with imidazole

FoDHACN (2.9 mg, 30 μmol, 1.0 eq.) and imidazole (10.2 mg, 0.15 mmol, 5.0 eq.) were dissolved in H_2O (0.3 mL, 0.1 M). The pH was adjusted using 5M HCl to 7.5. The reaction was left at room temperature in an NMR tube and monitored by ^1H NMR using an internal standard. After 96 hours, **14** was observed in 87% conversion (26 μmol, 87% yield, measured by internal standard).

^1H NMR (400 MHz, D_2O) δ 8.11 (s, 1H), 7.76 (dd, 1H, *J* = 1.1, 1.1 Hz), 7.23 (dd, 1H, *J* = 1.1, 1.1 Hz), 7.05 (dd, 1H, *J* = 1.1, 1.1 Hz), 5.30 (t, 1H, *J* = 6.4 Hz), 4.54 (dd, 1H, *J* = 14.5, 5.8 Hz), 4.47 (dd, 1H, *J* = 14.4, 7.0 Hz).
Minor conformer (from restricted formyl C–N rotation) peaks visible: δ 7.80 (s), 5.17 (dd, 1H, 5.4, 7.4 Hz), 4.57-4.44 (m)

^{13}C NMR (100MHz, D_2O): δ 163.7 (C̲HO), 138.4 (Im-C̲H), 128.2 (Im-C̲H), 120.5 (Im-C̲H), 116.4 (C̲N), 47.1 (C̲H_2), 40.4 (C̲H).
Minor conformer (from restricted formyl C–N rotation) peaks visible: δ 166.5 (C̲HO), 128.6 (Im-C̲H), 48.6 (C̲H2), 44.8 (C̲H).

MS: (LCMS, ESI+): 165.1

In formamide, with imidazole

FoDHACN (2.4 mg, 25 μmol, 1.0 eq.) and imidazole (8.5 mg, 0.125 mmol, 5.0 eq.) were dissolved in formamide (0.2 mL, 0.1 M). The reaction was left at room temperature in an open NMR tube and monitored by ^1H NMR using an internal standard. After 7 days, product **14** was observed in 86% conversion (22 μmol, 86% yield, measured by internal standard).

In formamide, $CDCl_3$ insert:
^1H NMR (400 MHz, $CDCl_3$) δ 8.75 (s, 1H), 8.31 – 8.29 (m, 1H), 7.77 – 7.75 (m, 1H), 7.60 – 7.58 (m, 1H), 5.89 (dd, 1H, *J* = 6.5 Hz, 6.5 Hz), 5.12 – 4.96 (m, 2H).
Minor conformer (from restricted formyl C–N rotation) peaks visible: δ 5.75 (dd, 1H, *J* = 6.35 Hz, 6.3 Hz).

^{13}C NMR (100 MHz, $CDCl_3$) δ 166.0 (C̲HO), 141.4 (Im-C̲H), 131.8 (Im-C̲H), 123.5 (Im-C̲H), 120.1 (C̲N), 50.0 (C̲H_2), 43.7 (C̲H).
Minor conformer (from restricted formyl C–N rotation) peaks visible: δ 132.1 (Im-C̲H), 123.4 (Im-C̲H), 120.2 (C̲N), 51.7 (C̲H_2), 47.9 (C̲H).

MS: (LCMS, ESI+): 165.1

Reactions of FoDHA-CN and Hydrosulfide

In formamide, with sodium hydrosulfide hydrate and formic acid

Fo-DHA-CN (3 mg, 0.026 mmol, 1.0 eq.) was dissolved in formamide (0.30 mL) and formic acid (3.4 μL, 0.078 mmol, 3.0 eq.) and NaSH.H_2O (60% NaSH by weight, 7.3 mg, 0.078 mmol, 3.0 eq.) were added. The reaction was left in a sealed NMR tube at room temperature and monitored by ^1H NMR spectroscopy using an internal standard.

In formamide, with sodium hydrosulfide hydrate

FoDHA-CN (3 mg, 0.026 mmol, 1.0 eq.) was dissolved in formamide (0.30 mL) NaSH.H_2O (60% NaSH by weight, 7.3 mg, 0.078 mmol, 3.0 eq.) was added. The reaction was left in a sealed NMR tube at room temperature and monitored by ^1H NMR spectroscopy using an internal standard.

In water, with sodium hydrosulfide hydrate, pH 7

FoDHA-CN (4.3 mg, 0.045 mmol, 1.0 eq.) was dissolved in water (0.35 mL, 0.13M). A solution of NaSH (0.45M) was prepared and the pH adjusted to 7.2. 0.10 mL was added to the FoDHA-CN solution and the final reaction pH adjusted to 7.0. The reaction was left at room temperature in an NMR tube with the cap on and monitored by ^1H NMR spectroscopy using an internal standard.

In water, with sodium hydrosulfide hydrate, pH 9, 1.5 eq.

FoDHA-CN (4.3 mg, 0.045 mmol, 1.0 eq.) was dissolved in water (0.35 mL, 0.13M). A solution of NaSH (0.45M) was prepared and the pH adjusted to 9.0. 0.10 mL (1.5 eq.) was added to the FoDHA-CN solution and the final reaction pH adjusted to 7.0. The reaction was left at room temperature in an NMR tube with the cap on and monitored by ^1H NMR spectroscopy using an internal standard.

In water, with sodium hydrosulfide hydrate, pH 9, 5 eq.

FoDHA-CN (4.3 mg, 0.045 mmol, 1.0 eq.) was added to a pH 9, 0.45M solution of NaSH (0.5 mL, 5.0 eq.), and the final pH adjusted to 9.0. The reaction was left at room temperature in an NMR tube with the cap on and monitored by ^{1}H NMR spectroscopy using an internal standard.

Sulfur-bridged di(formylaminonitrile)
^{1}H NMR (400 MHz, D_2O) δ 8.29 (s, 2H), 5.20 (dd, 2H, *J* = 7.1 Hz, 7.1 Hz), 3.42 – 3.19 (m, 4H).
^{13}C NMR (100MHz, D_2O): δ 163.9 (2 x $\underline{C}HO$), 117.9 (2 x $\underline{C}N$), 40.1 (2 x $\underline{C}H$), 33.5 ($\underline{C}H_2$), 33.5 ($\underline{C}H_2$).
The two ($\underline{C}H_2$) peaks are of equal intensity and are attributed to conformers with restricted formyl C–N rotation, although the other peaks are not split.
MS: (LCMS, ESI+): 227.0

FoCys-CN
In formamide, $CDCl_3$ insert:
^{1}H NMR (400 MHz, $CDCl_3$) δ 8.74 (s, 1H) 5.42 (t, 1H, *J* = 6.1 Hz,), 3.56 – 3.45 (m, 2H).
^{13}C NMR (100 MHz, $CDCl_3$) δ 166.0 ($\underline{C}HO$), 123.5 ($\underline{C}N$), 48.2 ($\underline{C}HCN$), 31.5 ($\underline{C}H_2S$)
MS: (LCMS, ESI-): 129.0

^{1}H NMR (400 MHz, D_2O) δ 8.22 (s, 1H), 4.78 (obscured by solvent), 2.98 (d, 2H, *J* = 6.7 Hz).
^{13}C NMR (100 MHz, D_2O) δ 163.8 ($\underline{C}HO$), 119.4 ($\underline{C}N$), 45.1 ($\underline{C}H$), 27.0 ($\underline{C}H_2$).

FoCys-SNH$_2$
^{1}H NMR (400 MHz, D_2O) δ 8.21 (s, 1H), 4.58 (dd, 1H, *J* = 7.4, 5.1 Hz,), 3.01 (dd, *J* = 13.1, 5.0 Hz, 1H), 3.01 (dd, 1H, *J* = 13.1, 7.1 Hz).
^{13}C NMR (100MHz, D_2O): δ 205.6 ($\underline{C}SNH_2$), 164.4 ($\underline{C}HO$), 63.1 ($\underline{C}H$), 30.0 ($\underline{C}H_2$).
MS: (LCMS, ESI-): 163.0

In formamide, $CDCl_3$ insert:
^{1}H NMR (400 MHz, $CDCl_3$) δ 8.78 (s, 1H) 5.22 (t, 1H, *J* = 5.4 Hz), 3.68 – 3.56 (m, 2H).
^{13}C NMR (100 MHz, $CDCl_3$) δ 210.5 ($\underline{C}SNH_2$), 166.5 ($\underline{C}HO$), 65.8 ($\underline{C}H$), 35.5 ($\underline{C}H_2$),

Sulfur-bridged di(formylaminothioamide)
In formamide, $CDCl_3$ insert:
^{1}H NMR (400 MHz, $CDCl_3$) δ 8.77 (s, 2H), 5.55 – 5.48 (m, 2H), 3.78 – 3.70 (m, 2H), 3.65 – 3.54 (m, 2H).
^{13}C NMR (100 MHz, $CDCl_3$) δ 208.5 ($\underline{C}SNH_2$) 166.1 ($\underline{C}HO$), 60.2 ($\underline{C}H$), 60.2 ($\underline{C}H$), 39.7 ($\underline{C}H_2$), 39.6 ($\underline{C}H_2$).
The splitting of some of these ^{13}C peaks, with equal intensity, is attributed to conformers with restricted formyl C–N rotation.

^{1}H NMR (400 MHz, D_2O) δ 8.13 – 7.98 (m, 2H), 4.74 (dd, 2H, *J* = 7.5, 5.6 Hz), 3.12 – 2.90 (m, 4H).
^{13}C NMR (100 MHz, D_2O) δ 205.4 ($\underline{C}SNH_2$), 205.3 ($\underline{C}SNH_2$), 164.4 (2 x $\underline{C}HO$), 57.6 ($\underline{C}H$), 57.53 ($\underline{C}H$), 36.1 (2 x $\underline{C}H_2$).
The splitting of some of these ^{13}C peaks, with equal intensity, is attributed to conformers with restricted formyl C–N rotation.
MS: (LCMS, ESI-): 293.0

Hydrolysis of (N-formyl)aminonitriles

The relevant aminonitrile (0.025 mmol, reaction concentration 50 mM) was dissolved in 0.5 mL of 200 mM borate buffer and the pH adjusted to pH 10. The reaction was transferred to an NMR tube and heated at 40 °C, monitored by ^{1}H NMR spectroscopy using an internal standard.

15N labelled material

(^{15}N)Valine nitrile hydrochloride, (^{15}N)ValCN.HCl

The hydrochloride salt of ^{15}N-valine nitrile ((^{15}N)ValCN.HCl) was prepared based on the procedure for the unlabelled material above, based on the method of Paventi and Edward[1]. $^{15}NH_4Cl$ was used in place of NH_4Cl, and a solution of $^{15}NH_4OH$ was prepared from $^{15}NH_4Cl$ and NaOH, and used in place of NH_4OH.

^{1}H NMR (400 MHz, D_2O): δ 4.50 – 4.35 (m, 1H, HCCN), 2.40 – 2.25 (m, 1H, HC(CH_3)$_2$), 1.25 – 0.99 (m, 6H, 2 x CH_3).
MS (LCMS ESI+): 100.1

(^{15}N)(N-formyl)valine nitrile, (^{15}N)FoVal-CN

Was prepared using formic acid and EDCI as per (N-formyl)valine nitrile, but using (^{15}N)ValCN.HCl as starting material.

^{1}H NMR (400 MHz, D_2O) δ 8.09 (d, 1H, *J* = 15.8 Hz, HCO), 4.65 (m, 1H, HCCN), 2.09 (m, 1H, HC(CH_3)$_2$), 1.01 (d, 3H, *J* = 6.8 Hz, CH_3), 0.98 (d, 3H, *J* = 6.8 Hz, CH_3);
MS (LCMS ESI-): 126.2

^{15}N NMR (51 MHz, formamide, $CDCl_3$ insert) δ 116.4

References:

[1] M. Paventi, J. T. Edward, *Can. J. Chem.* **1987**, *65*, 282–289.

[2] G. Ksander, G. Bold, R. Lattmann, C. Lehmann, T. Früh, Y. -B Xiang, K. Inomata, H. -P Buser, J. Schreiber, E. Zass, et al., *Helv. Chim. Acta* **1987**, *70*, 1115–1172.

[3] J. C. Sheehan, D. D. H. Yang, *J. Am. Chem. Soc.* **1958**, *80*, 1154–1158.

[4] K. S. L. Chan, H. Y. Fu, J. Q. Yu, *J. Am. Chem. Soc.* **2015**, *137*, 2042–2046.

[5] R. J. Rousseau, J. A. May, R. K. Robins, L. B. Townsend, *J. Heterocycl. Chem.* **1974**, *11*, 233–235.

Formylation reactions

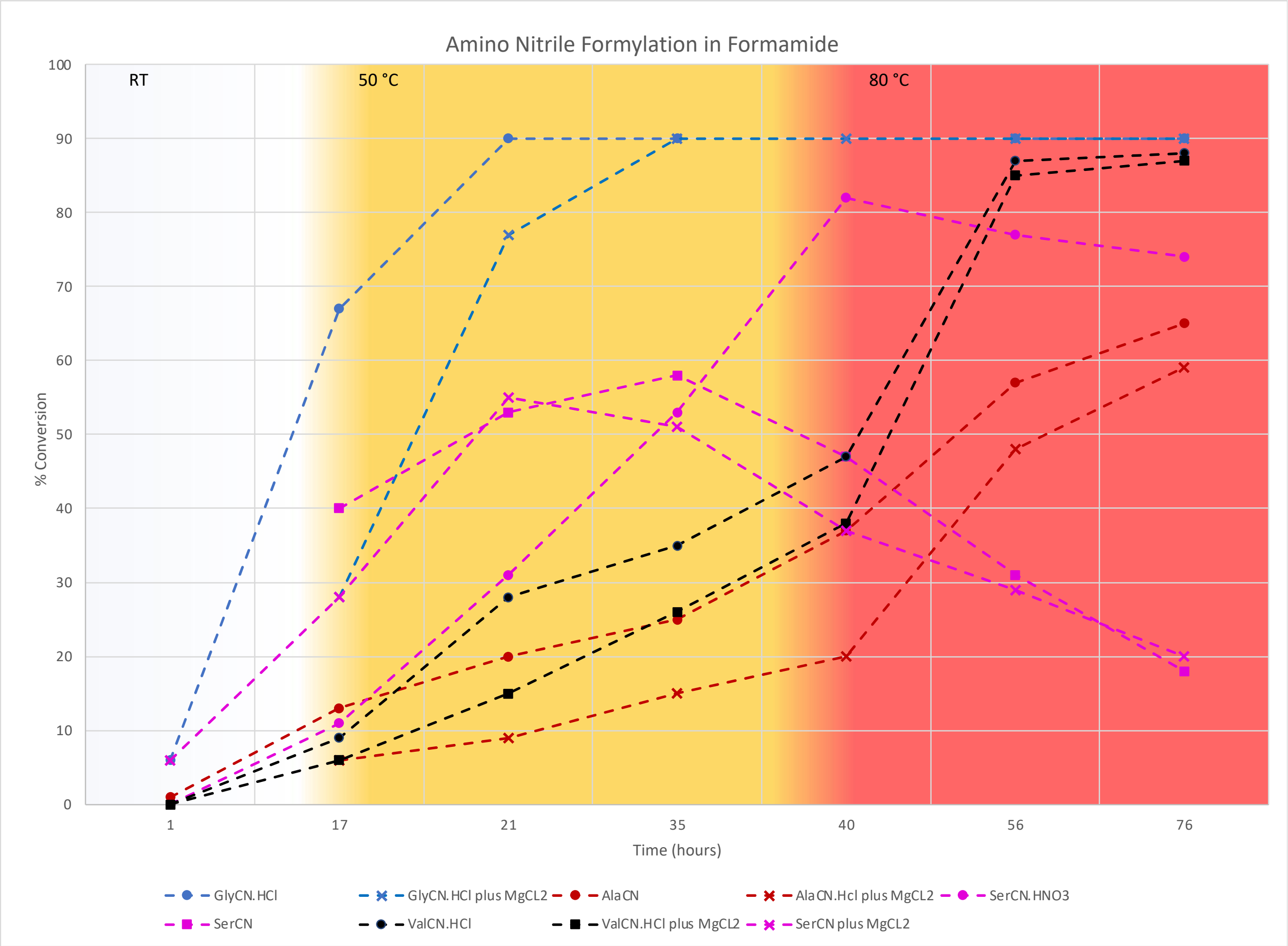

Graph S1. Formylation of aminonitriles in formamide.

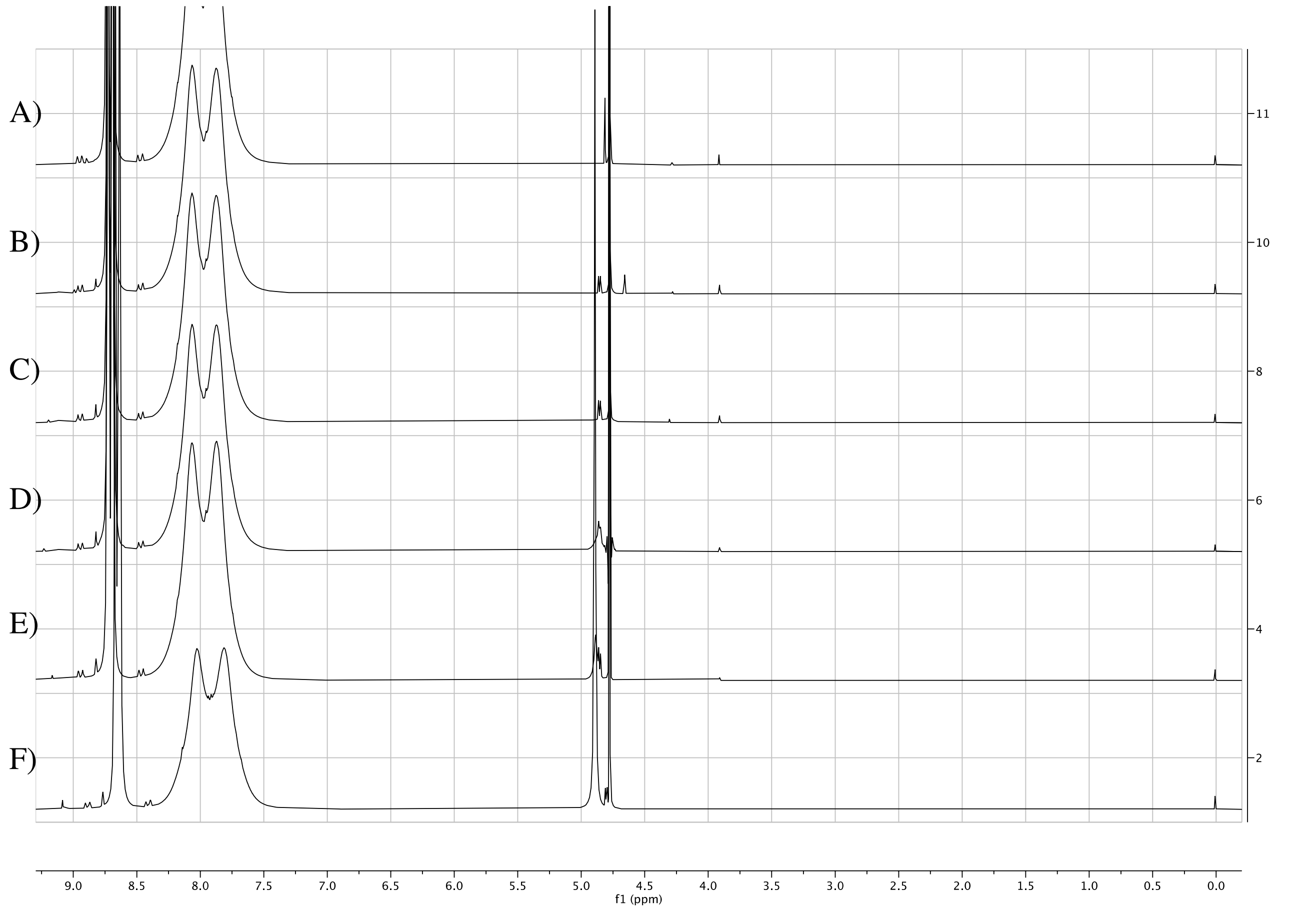

Fig S1. Stack showing conversion of Gly-CN to FoGly-CN. A) 1h RT; B) +16 h RT; C) +4h 50°C; D) 16h 50°C; E) +5h, 80°C; F) +16h, 80°C.

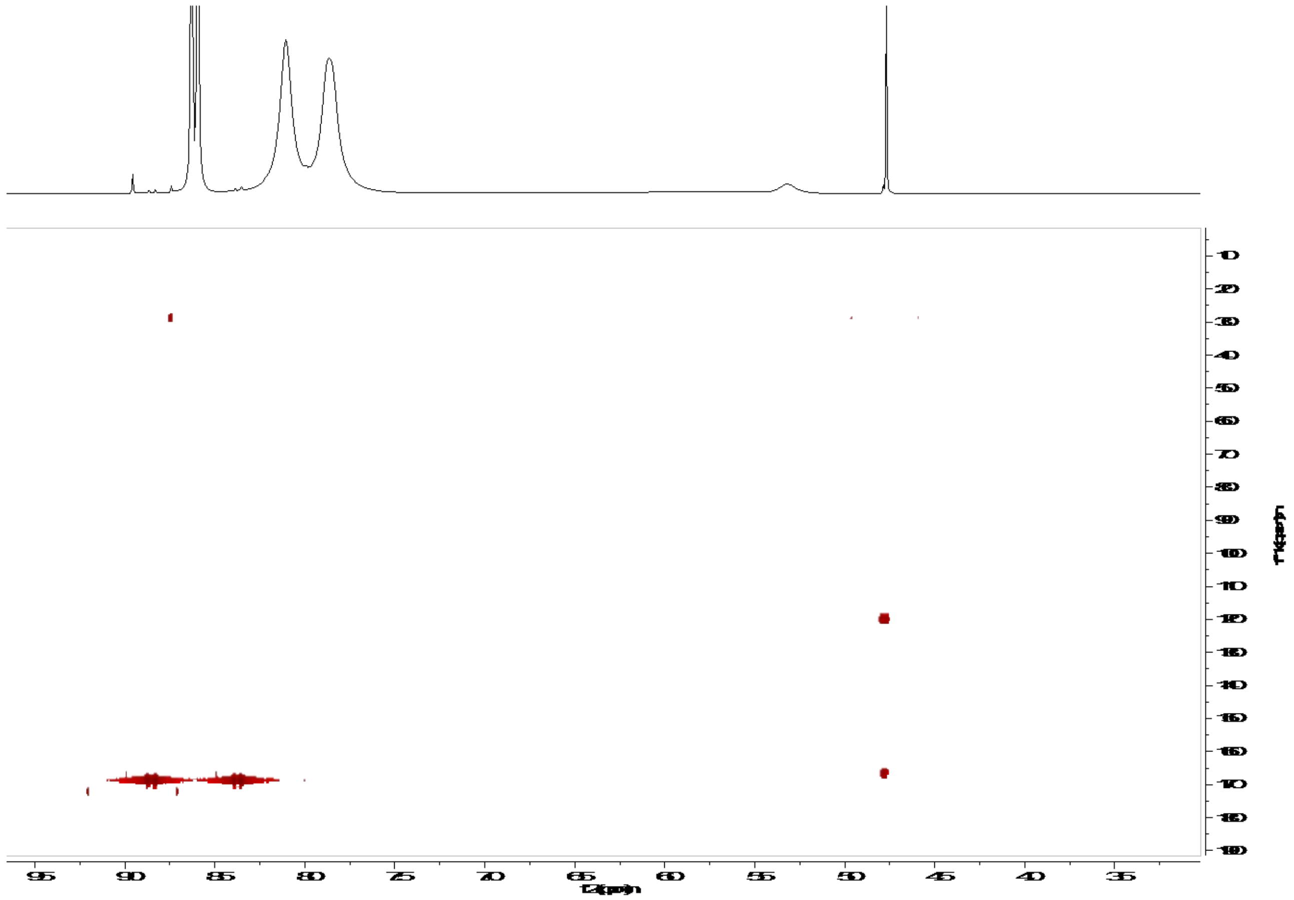

Fig S2. HMBC spectrum showing a single product (FoGly-CN) after 2h heating at 100°C.

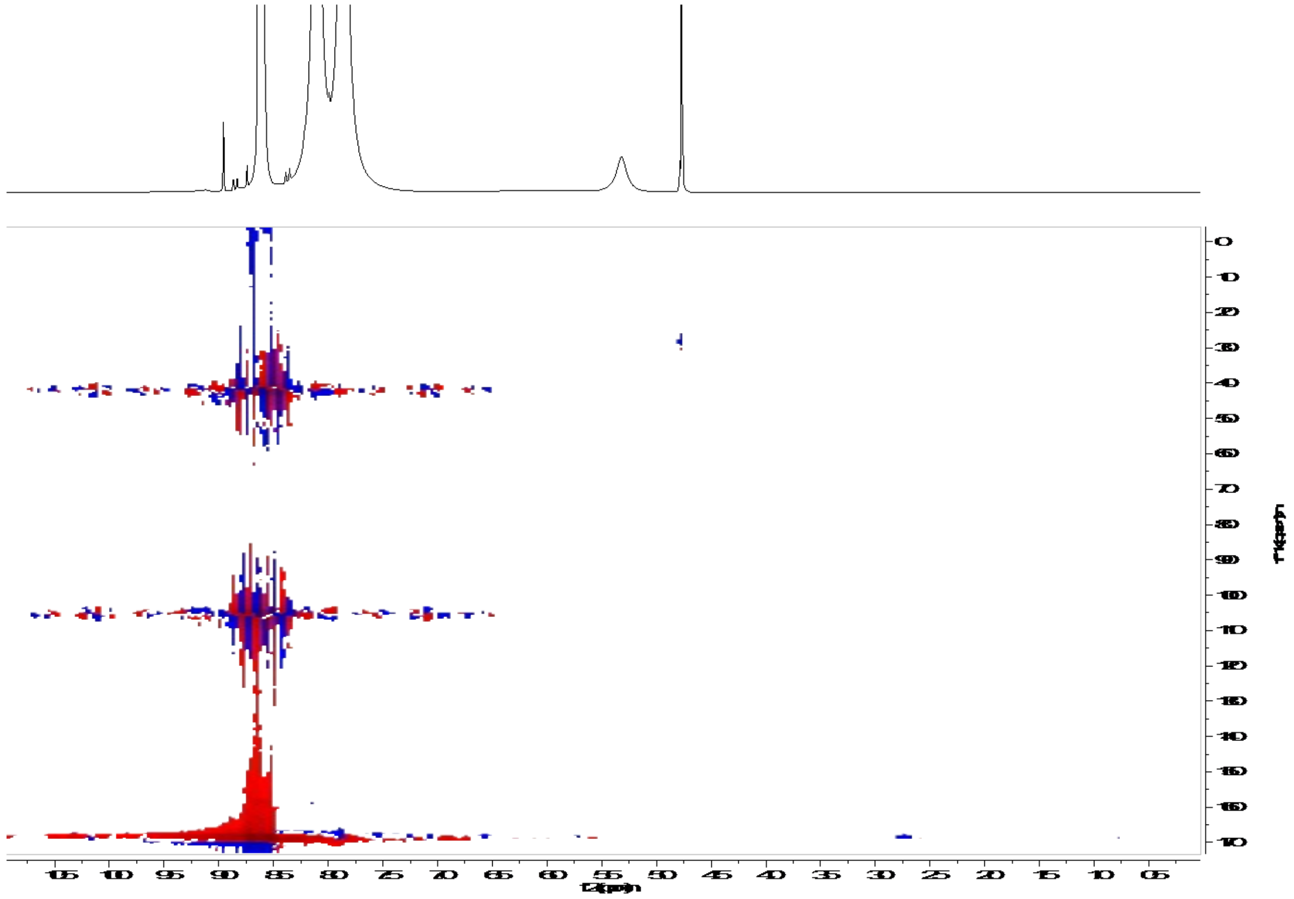

Fig S3. HSQC spectrum showing a single product (FoGly-CN) after 2h heating at 100°C.

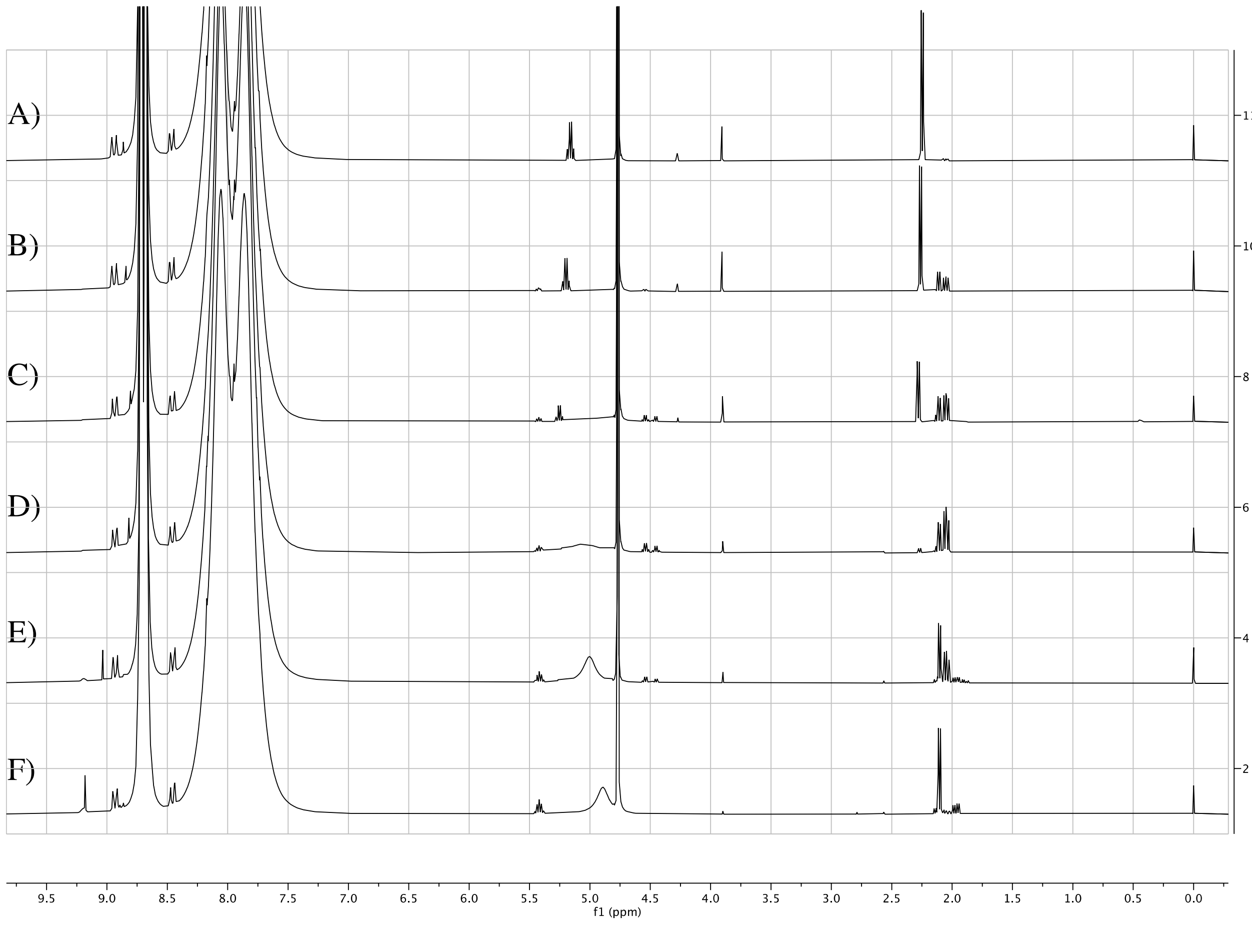

Fig S4. Stack showing conversion of Ala-CN to FoAla-CN. A) 1h RT; B) +16h RT; C) +4h 50°C; D) +16h 50°C; E) +5h, 80°C; F) +16h, 80°C.

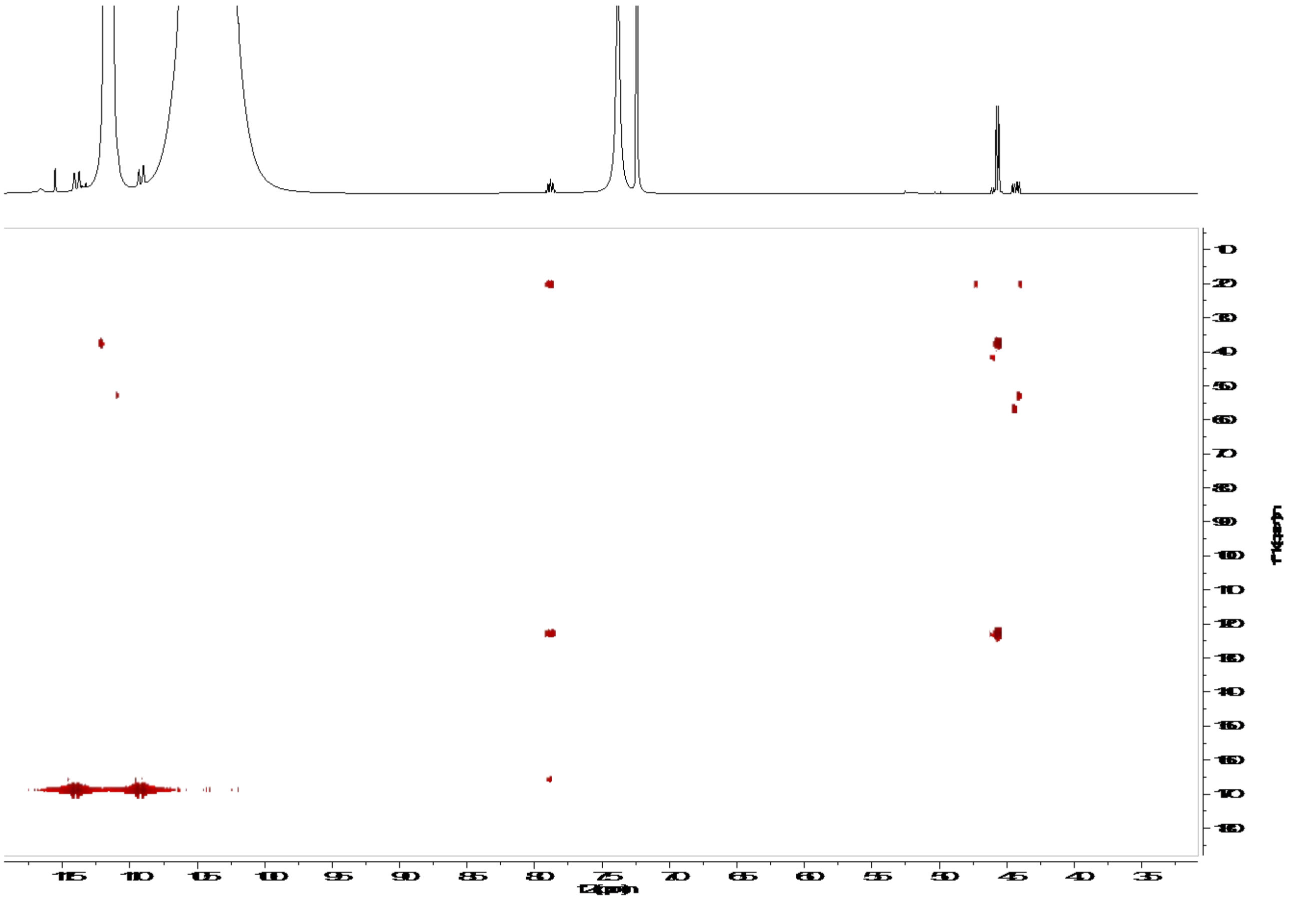

Fig S5. HMBC spectrum showing major product FoAlaCN after 2h heating at 100°C.

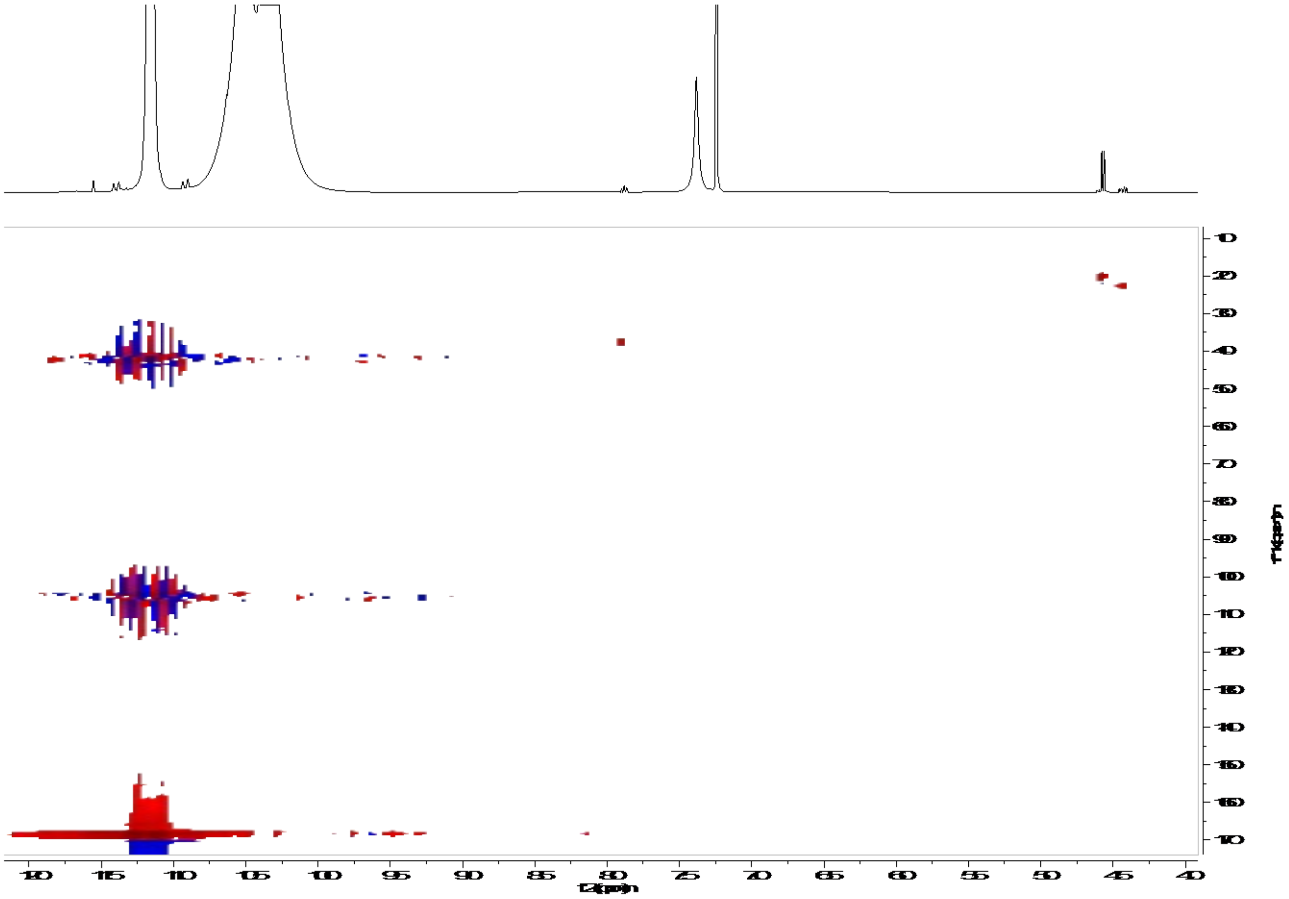

Fig S6. HMBC spectrum showing major product FoAlaCN after 2h heating at 100°C.

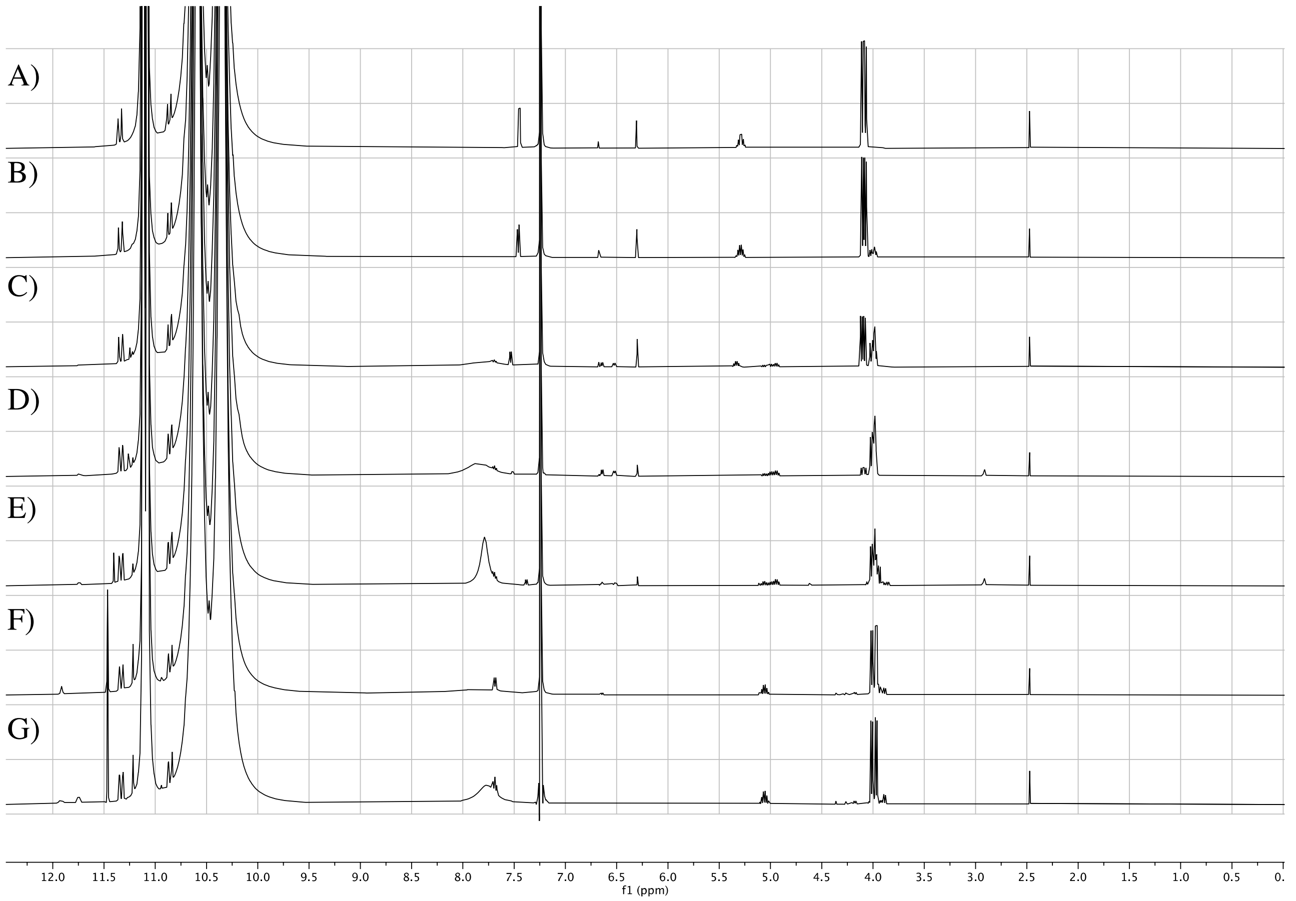

Fig S7. Stack of ^{1}H NMR spectra showing conversion of ValCN to FoValCN. A) 1h RT; B) +16 h RT; C) +4h 50°C; D) +16h 50°C; E) + 80°C 5h; F) +80°C 16h; G) +20h, 80°C.

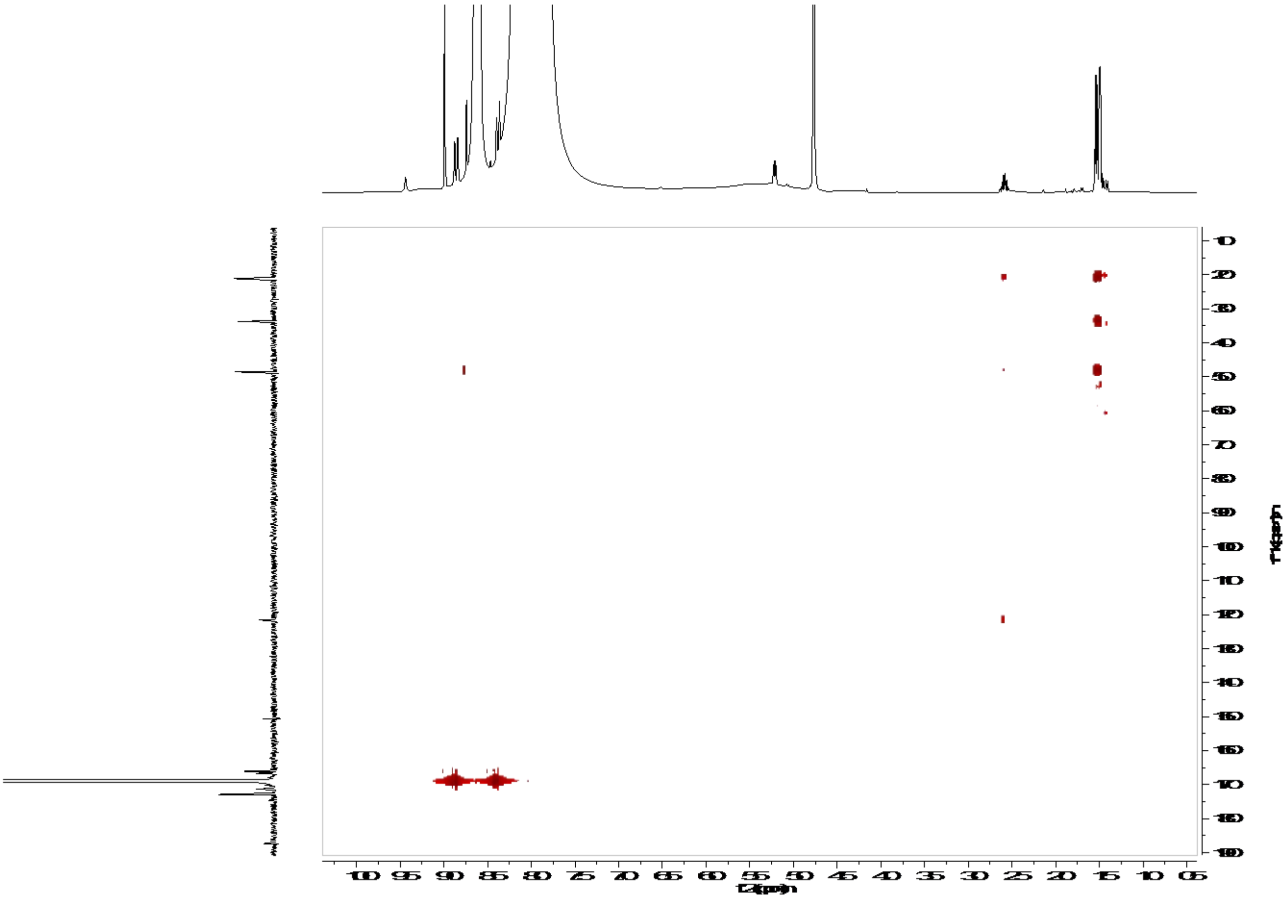

Fig S8. HMBC spectrum showing major product FoValCN after 16h heating at 80°C.

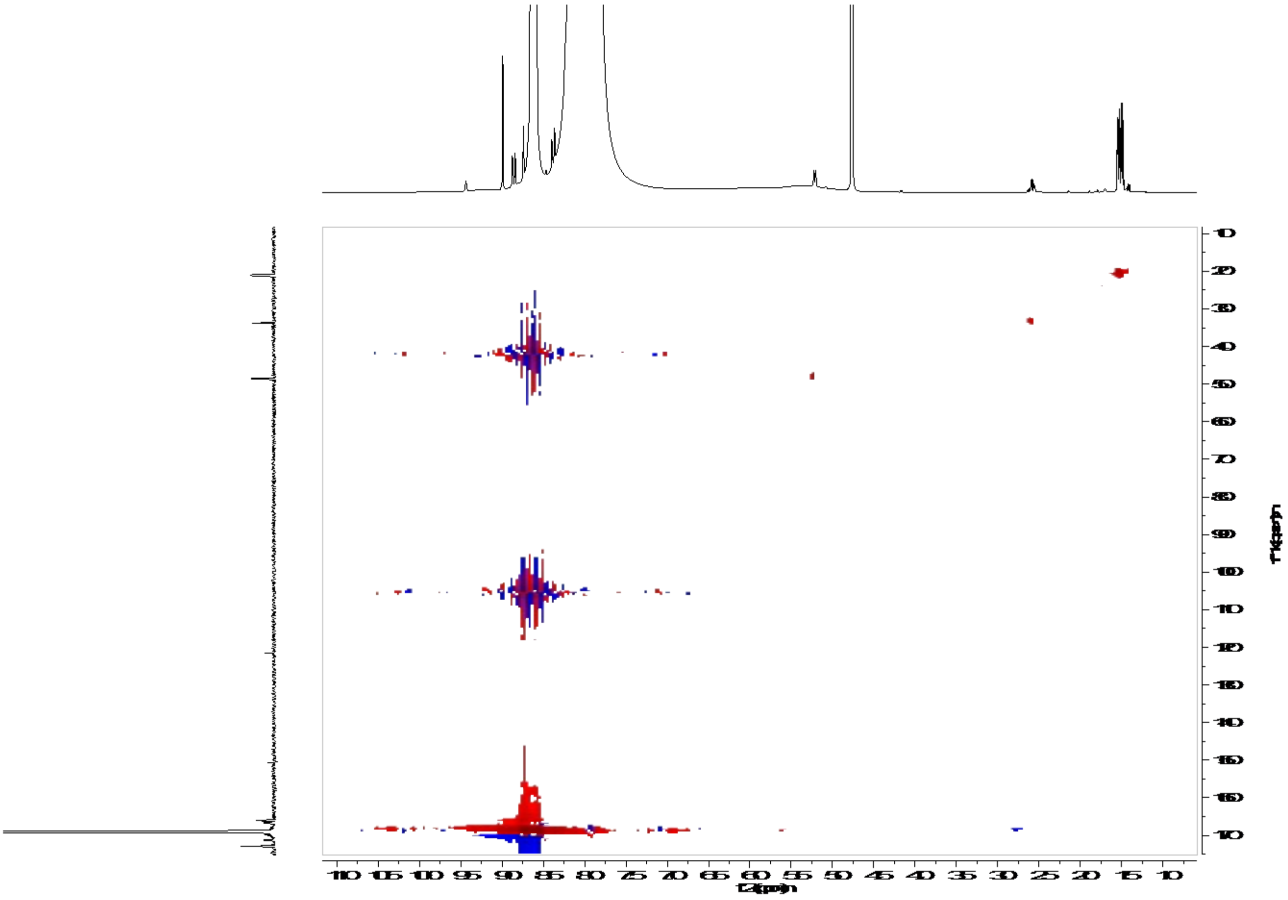

Fig S9. HSQC spectrum showing major product FoValCN after 16h heating at 80°C.

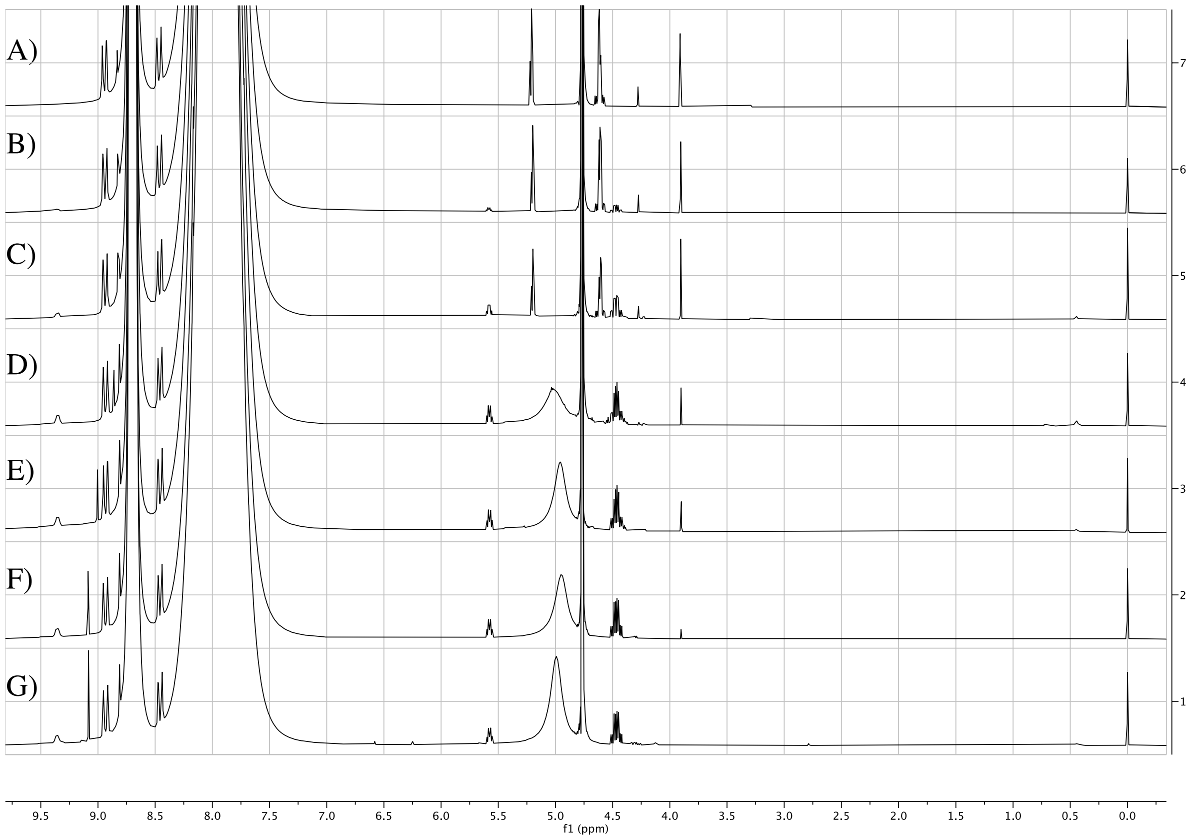

Fig S10. Stack of ^{1}H NMR spectra showing conversion of SerCN to FoSerCN and FoDHA-CN. A) 1h RT; B) +16 h RT; C) +4h 50°C; D) +16h 50°C; E) +5h 80°C; F) +16h 80°C; G) +20h 80°C.

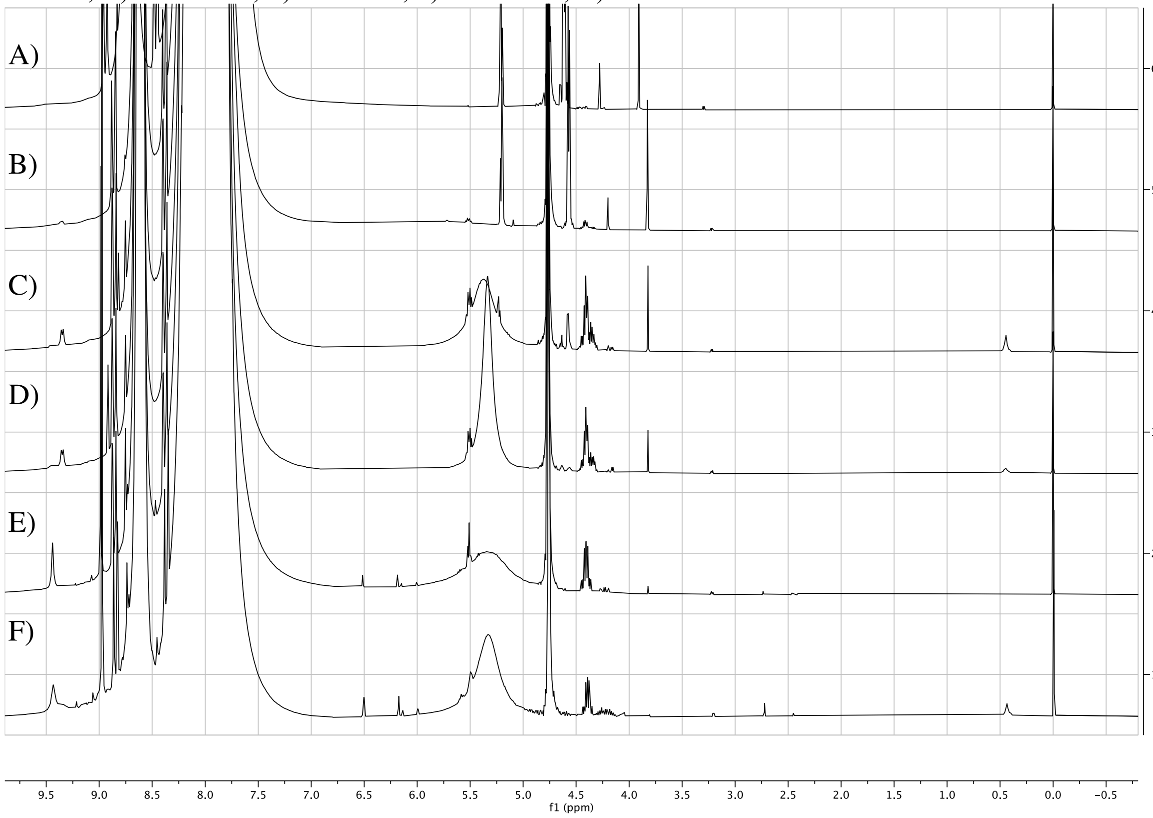

Fig S11 Stack of ^{1}H NMR spectra showing conversion of SerCN.HNO_3 with $MgCl_2$ and formic acid in formamide to FoSerCN and FoDHA-CN. The increased amount of FoDHA-CN compared to the reaction without $MgCl_2$ is apparent. A) 1h RT; B) +16 h RT; C) +20h 50°C; D) +5h 80°C; E) +16h 80°C; F) +20h 80°C.

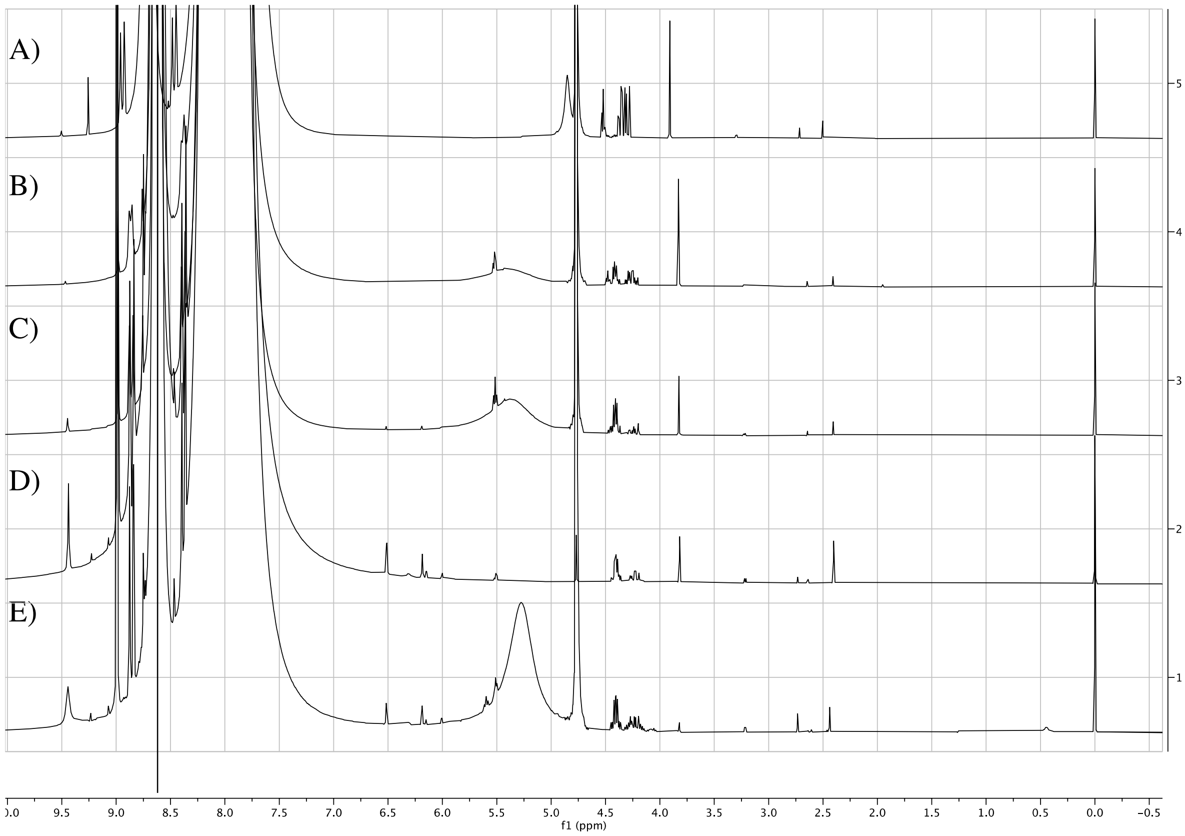

Fig S12. Stack of ^{1}H NMR spectra showing conversion of SerCN (free base) with $MgCl_2$ (5eq.) to FoSerCN and FoDHA-CN. The increased amount of FoDHA-CN compared to the reaction of the HNO_3 salt is apparent. A) 1h RT; B) +16 h RT; C) 20h 50°C; D) + 80°C 5h; E) +80°C 16h; F) +20h 80°C.

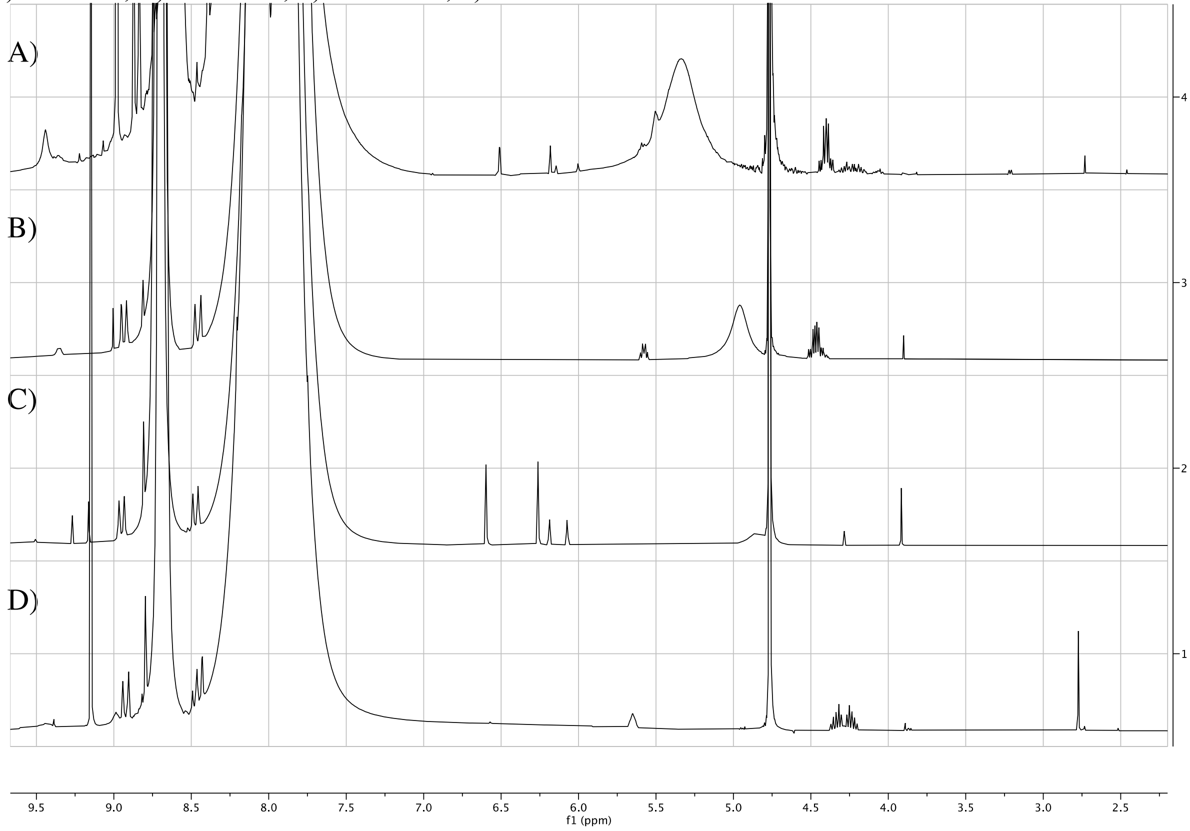

Fig S13. Stack of ^{1}H NMR spectra showing conversion of $SerCN.HNO_3$ with $MgCl_2$ and formic acid in formamide to FoSerCN and FoDHA-CN, with standards. A) reaction after 36 h 80 °C; B) FoSer-CN standard in formamide; C) FoDHA-CN standard in formamide; D) Fo-FoNH-Ala-CN standard in formamide.

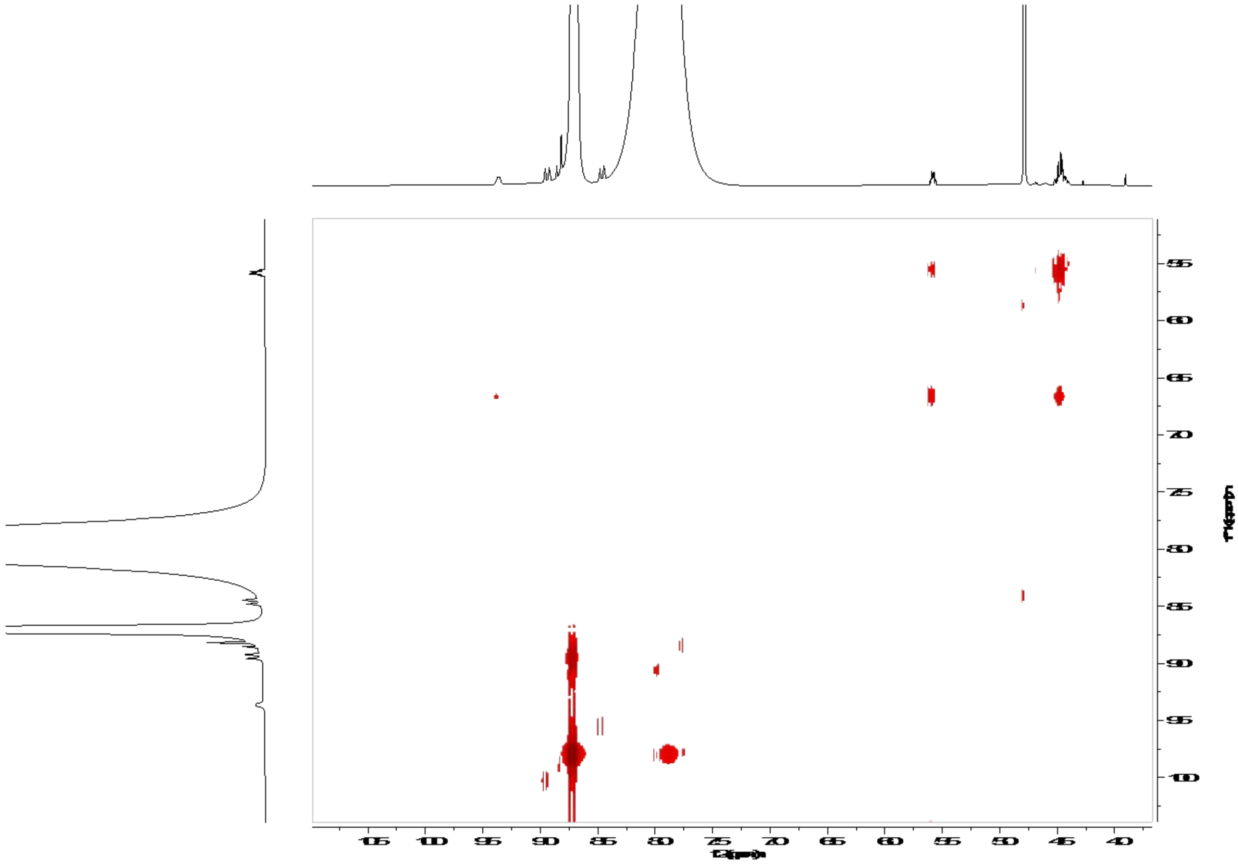

Fig S14. COSY spectrum of FoSer-CN in formamide.

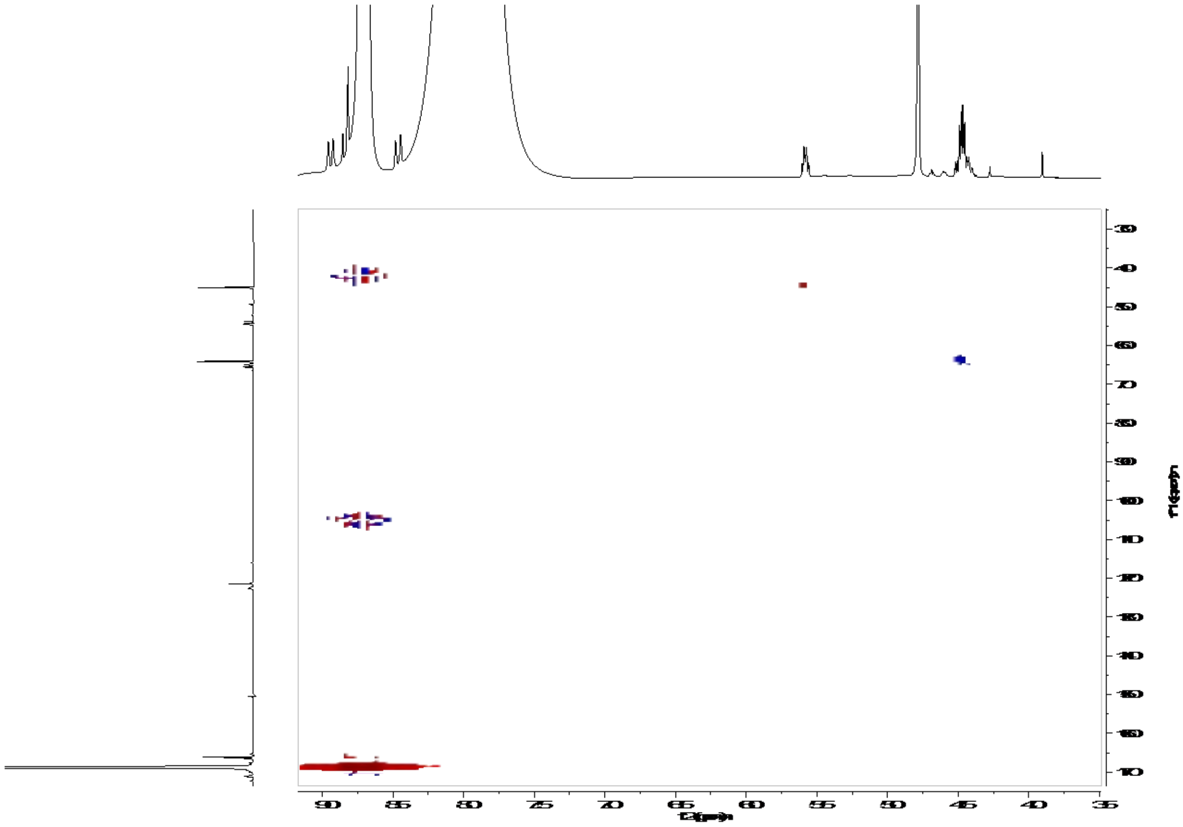

Fig S15. HSQC spectrum of FoSer-CN in formamide.

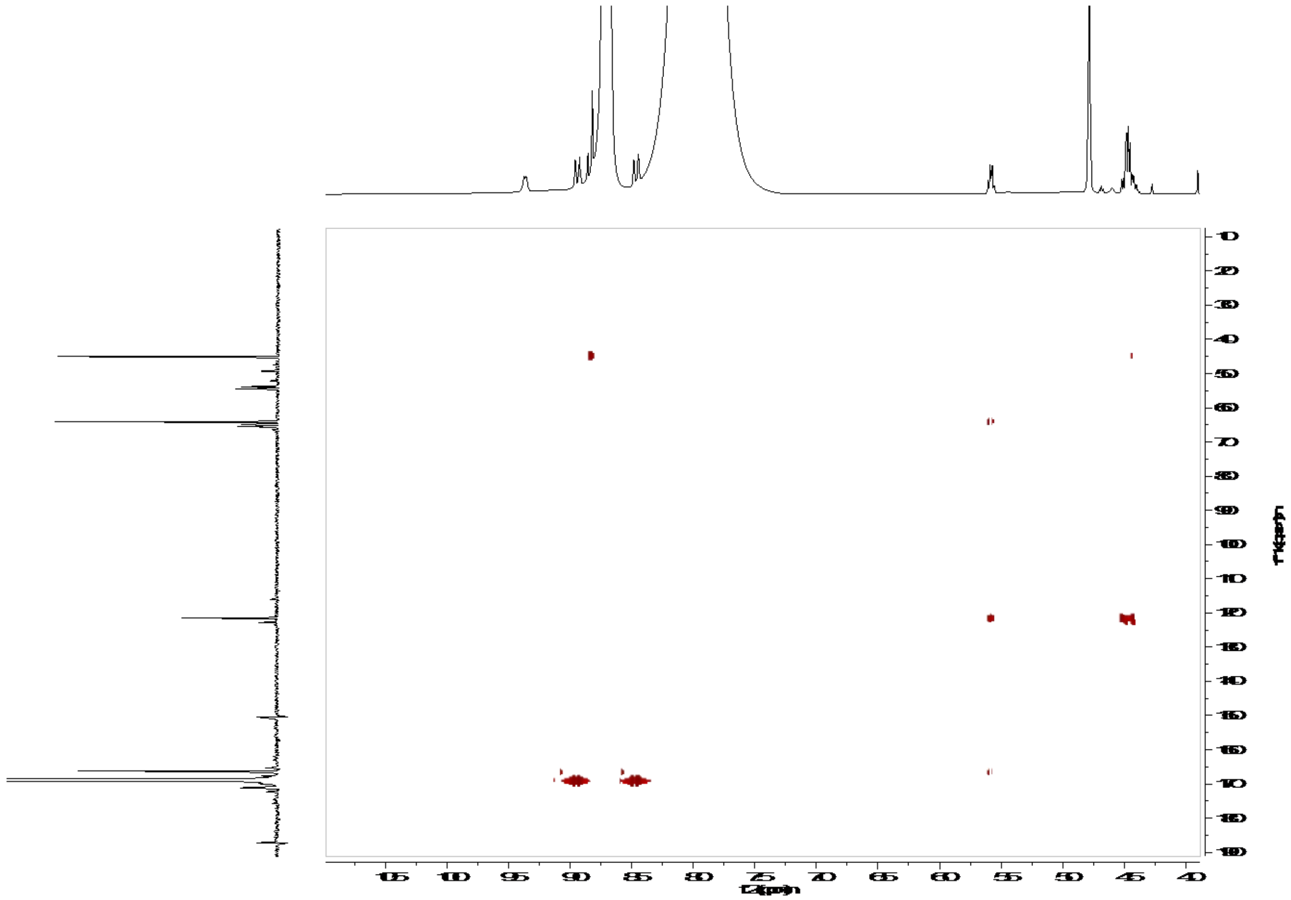

Fig S16. HMBC spectrum of FoSer-CN in formamide.

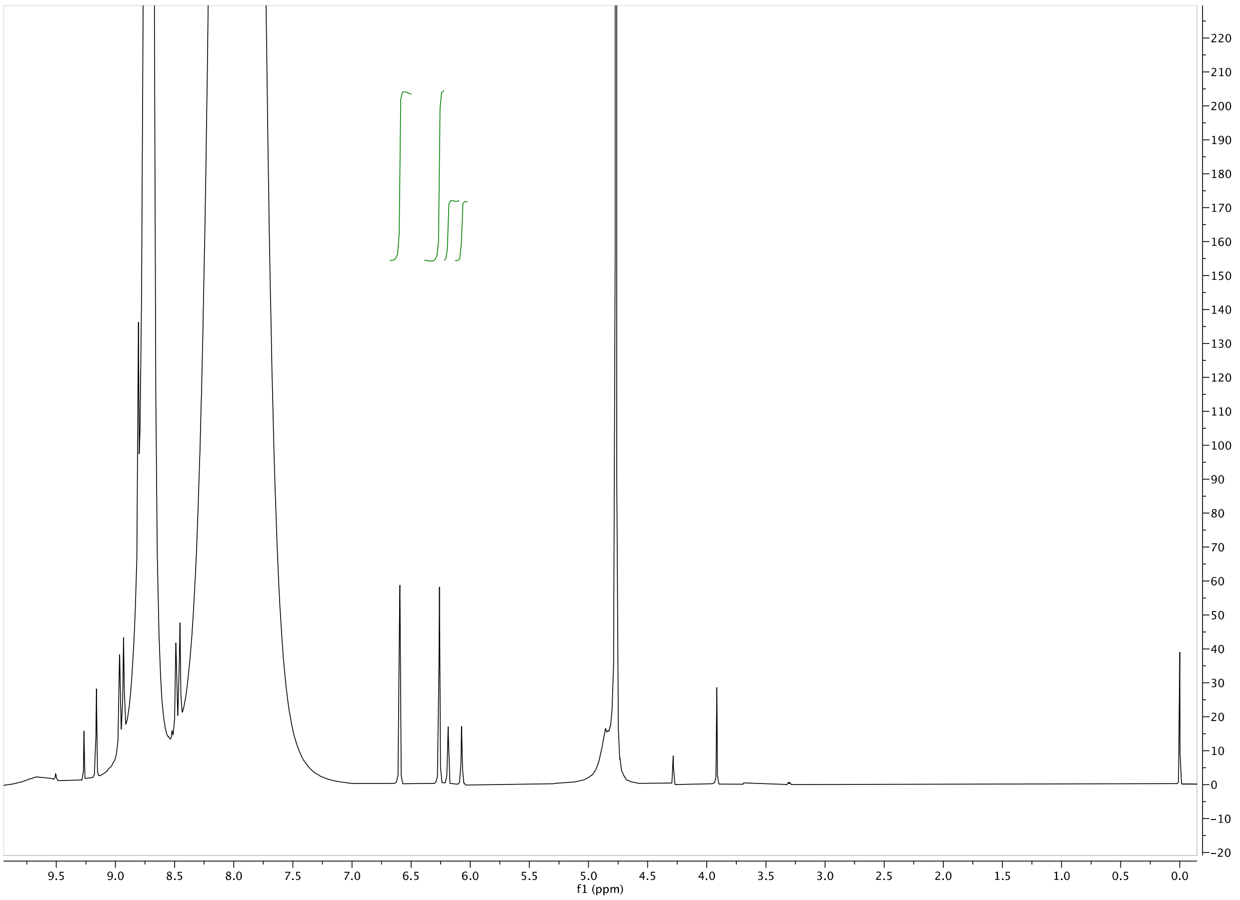

Fig S17. ^{1}H NMR spectrum of FoDHA-CN in formamide.

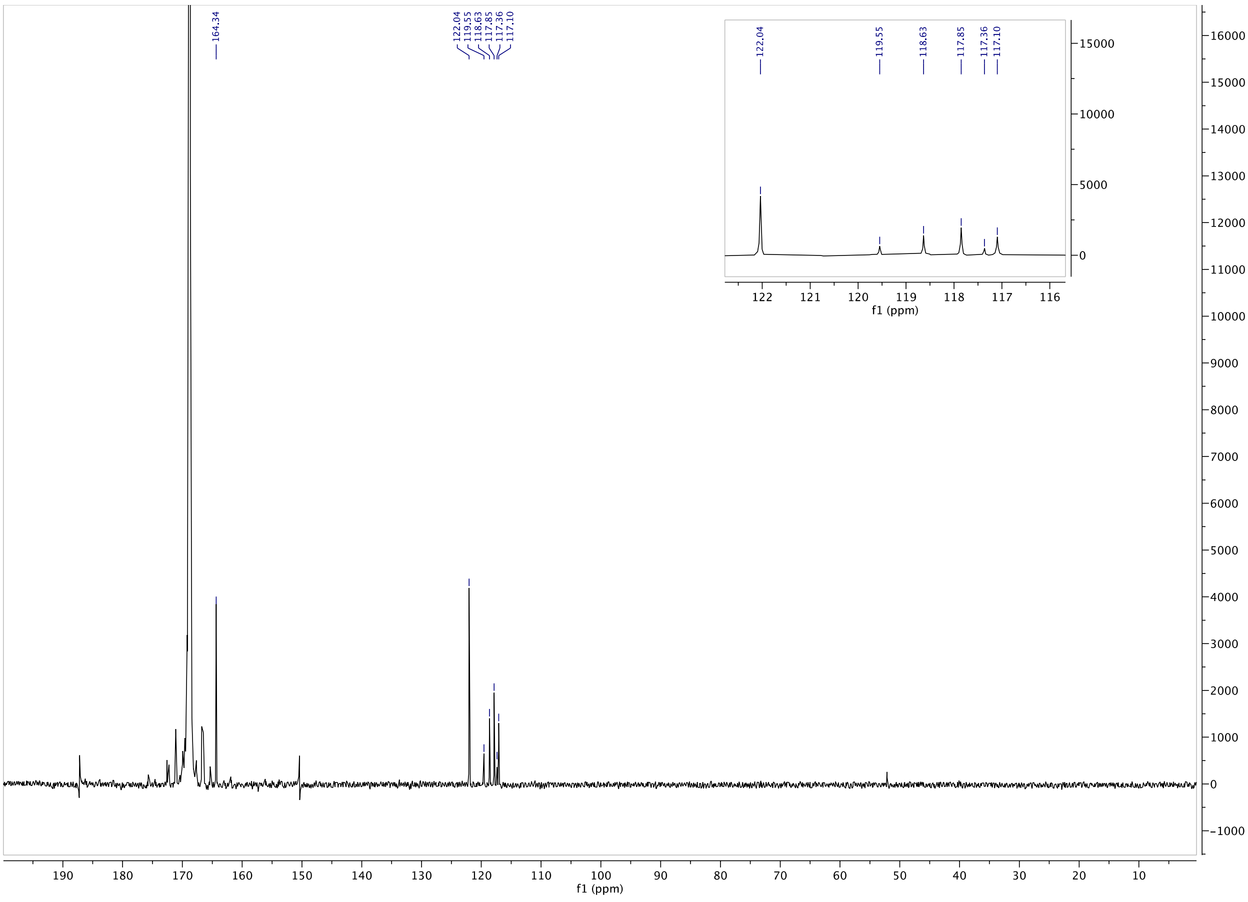

Fig S18. ^{13}C NMR spectrum of FoDHA-CN in formamide.

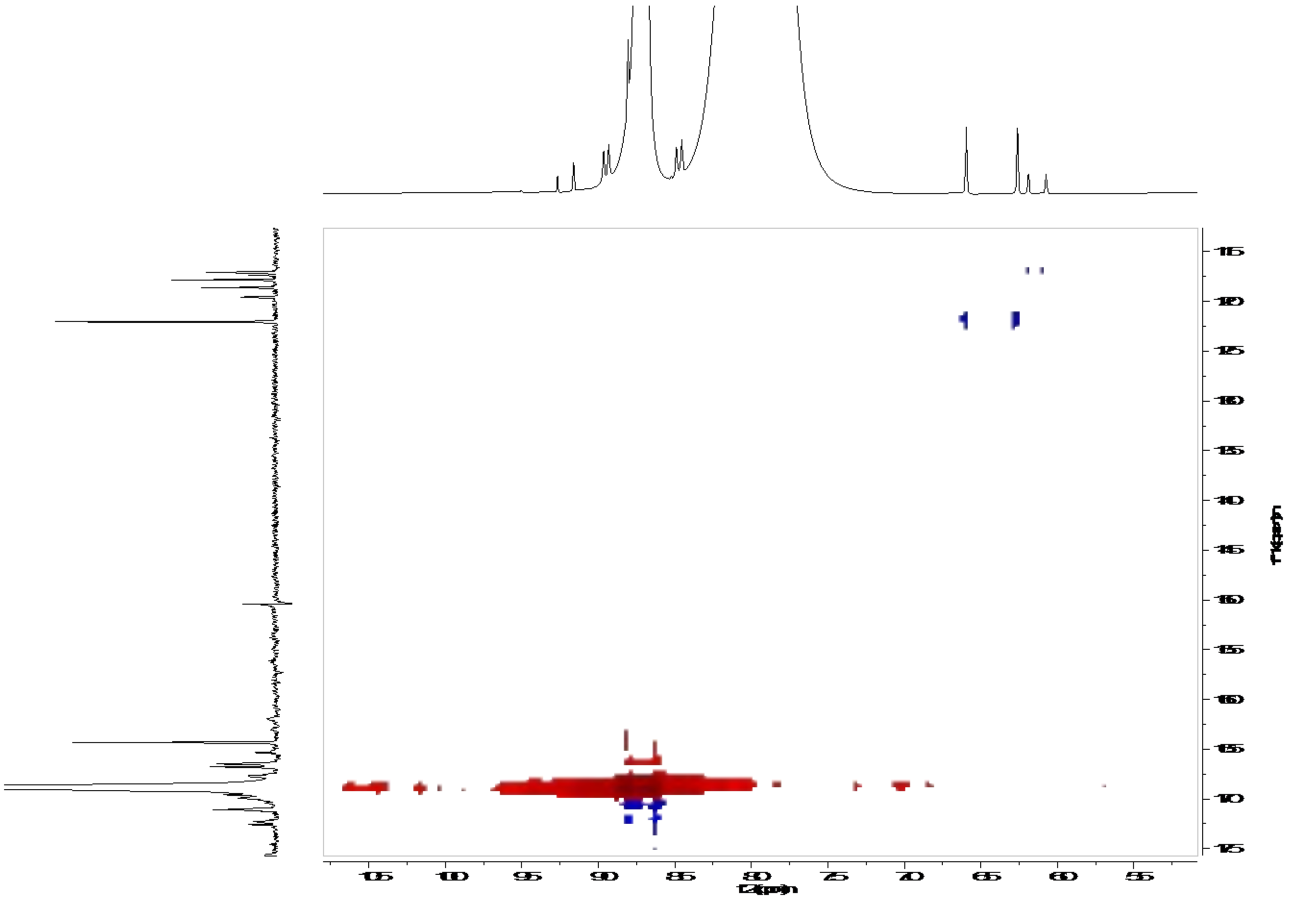

Fig S19. HSQC spectrum of FoDHA-CN in formamide.

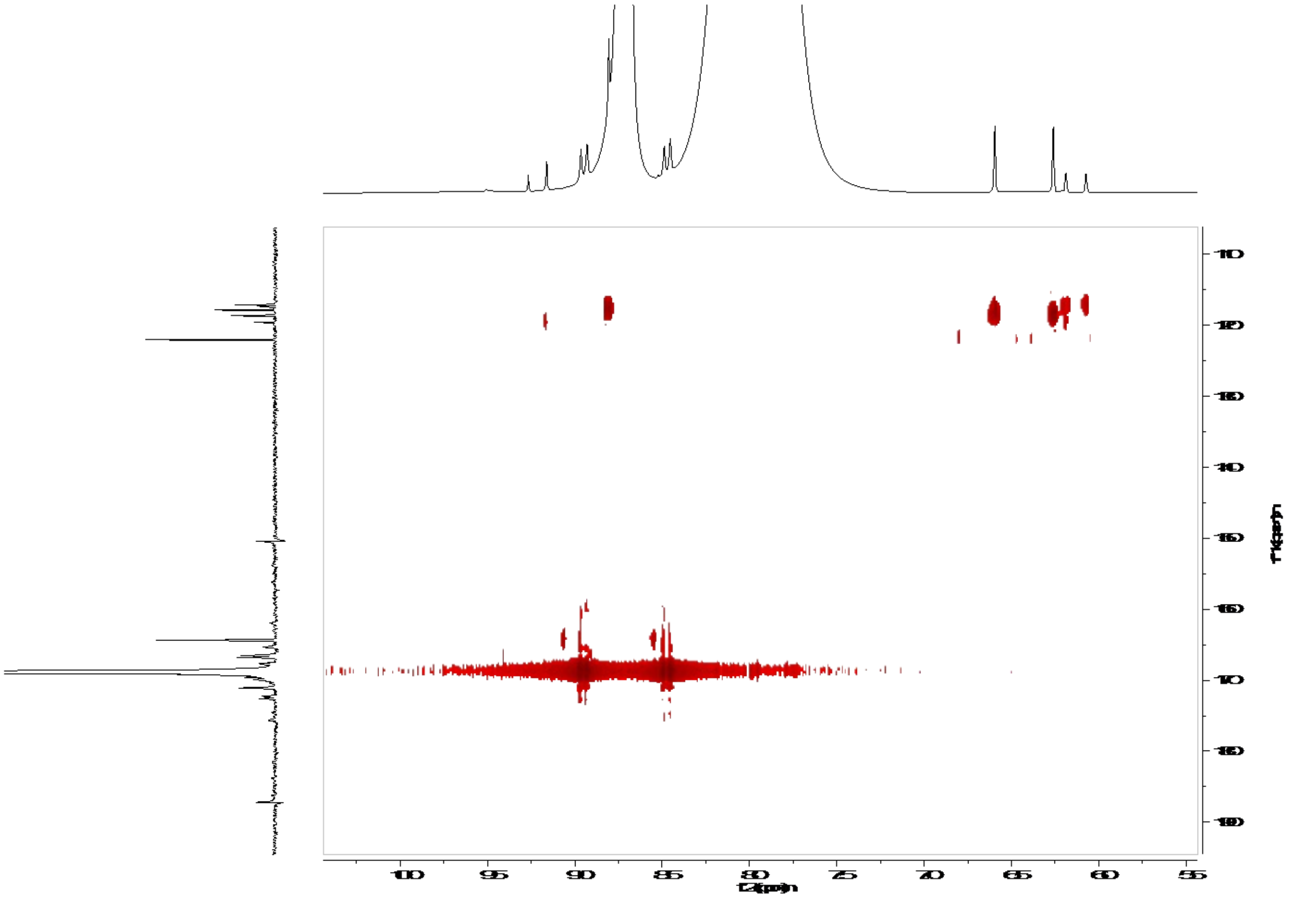

Fig S20. HMBC spectrum of FoDHA-CN in formamide.

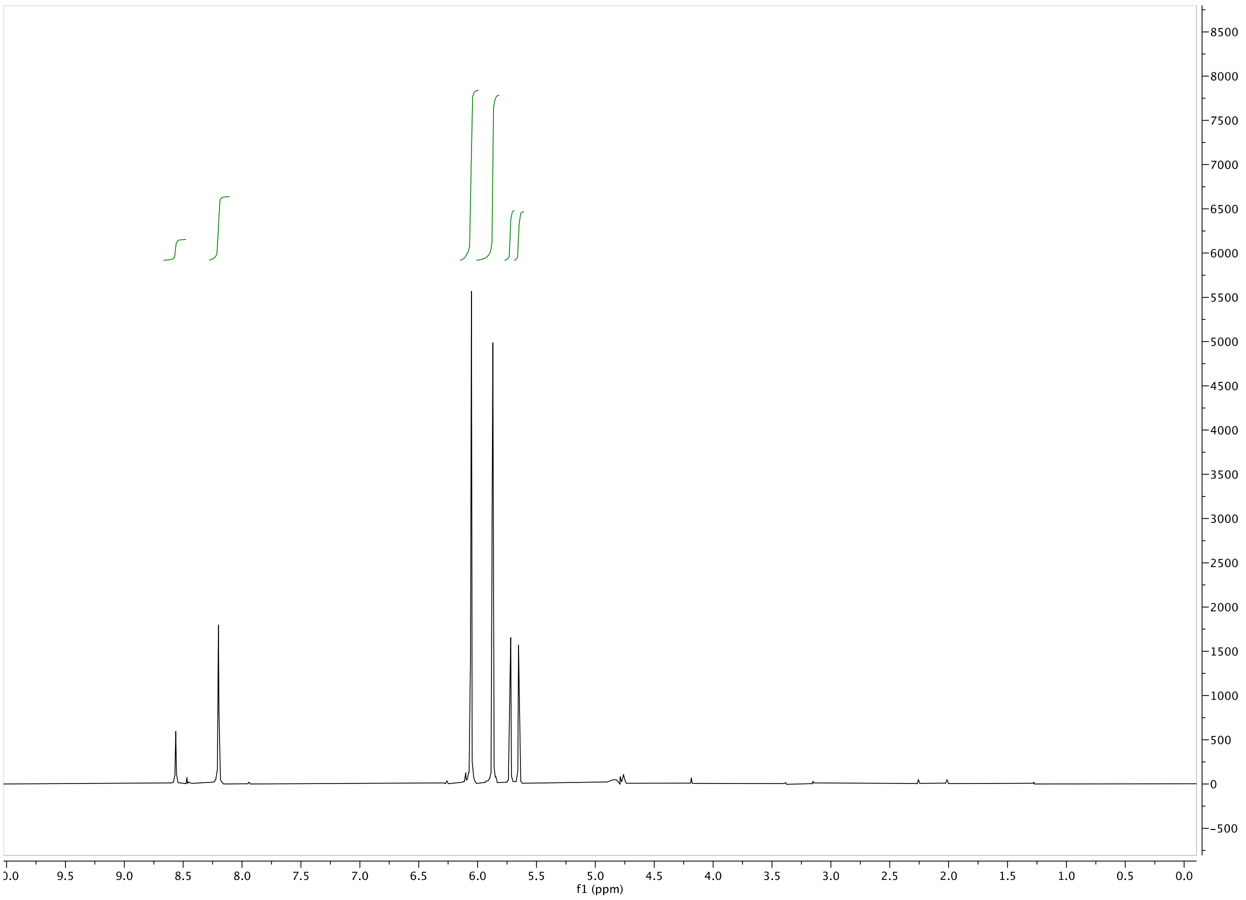

Fig S21. ^{1}H NMR spectrum of FoDHA-CN in water.

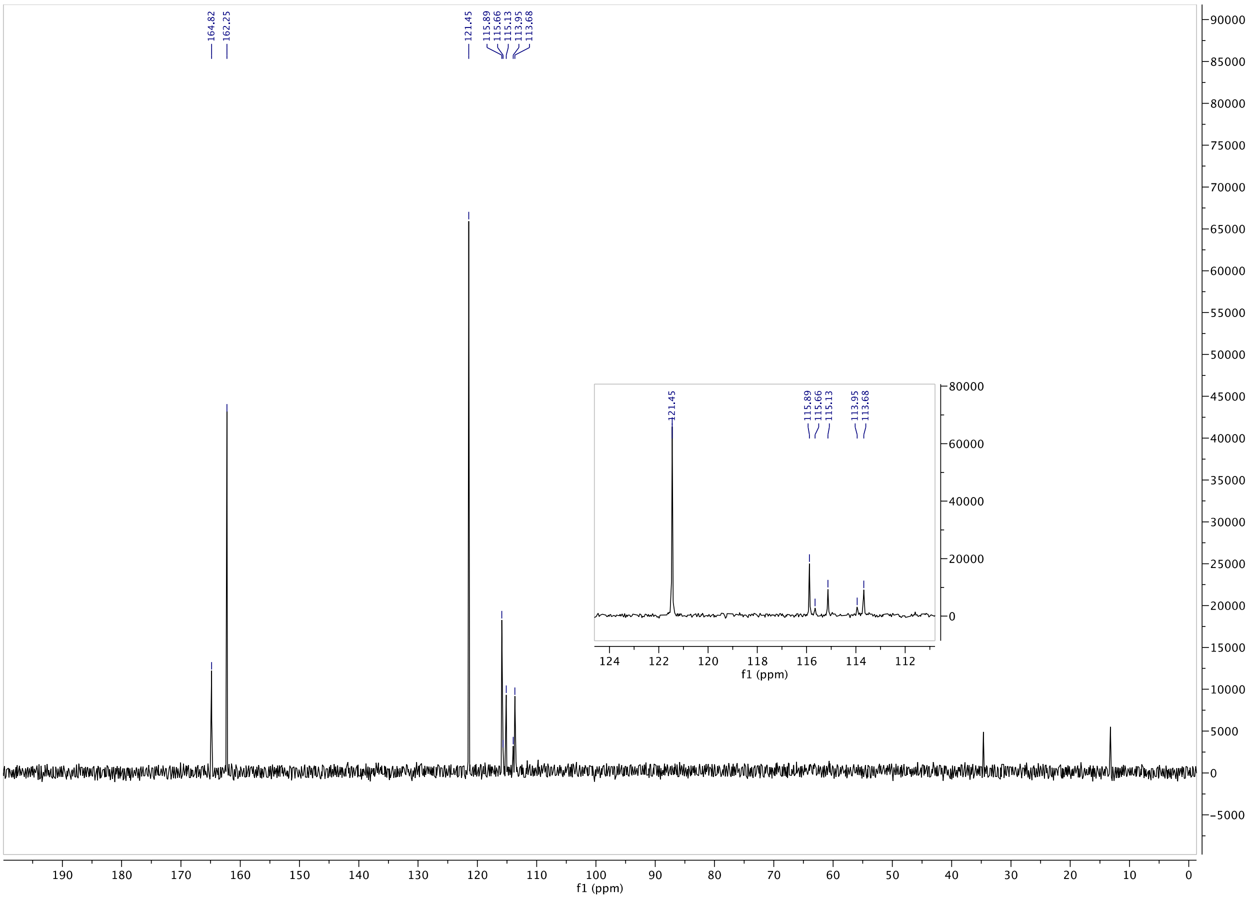

Fig S22. ^{13}C NMR spectrum of FoDHA-CN in water.

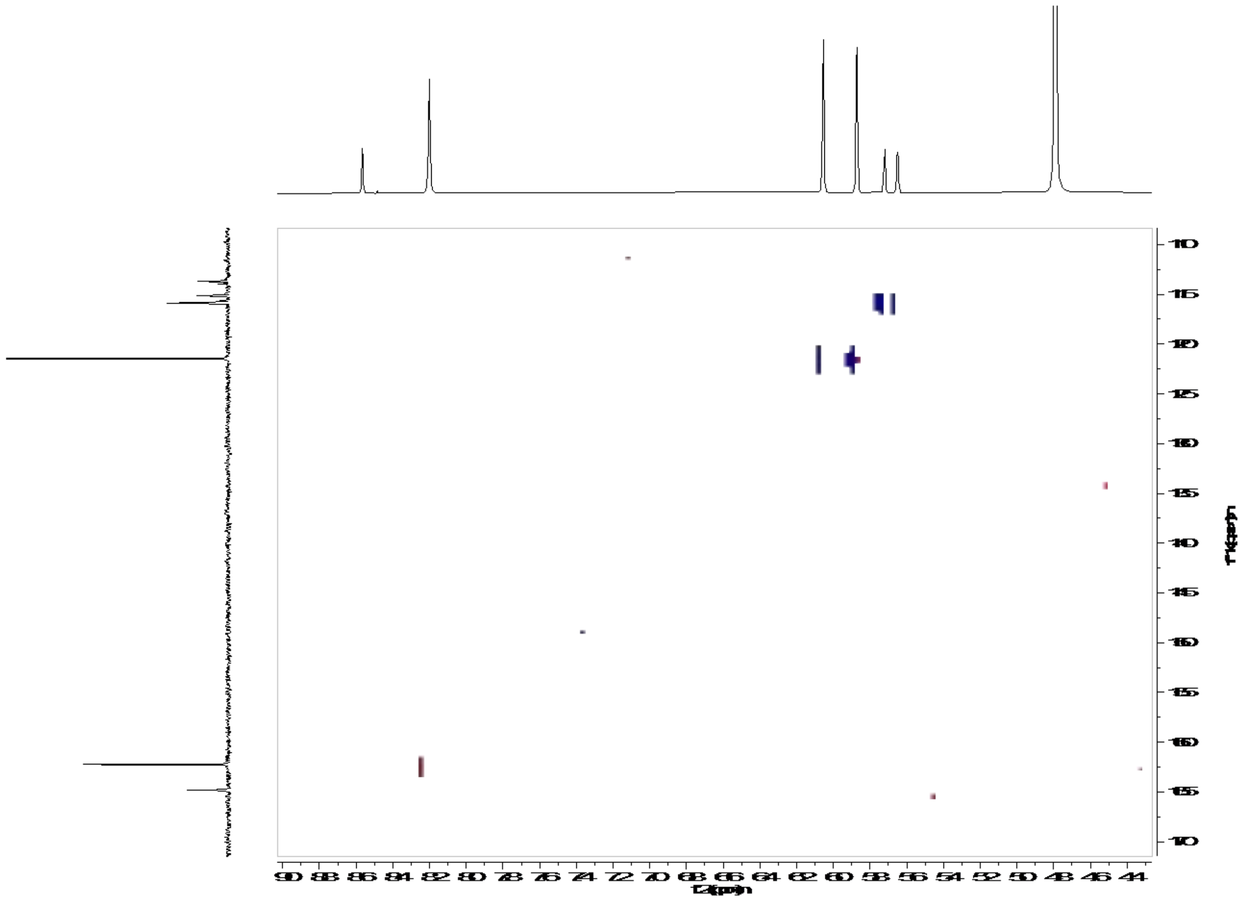

Fig S23. HSQC spectrum of FoDHA-CN in water.

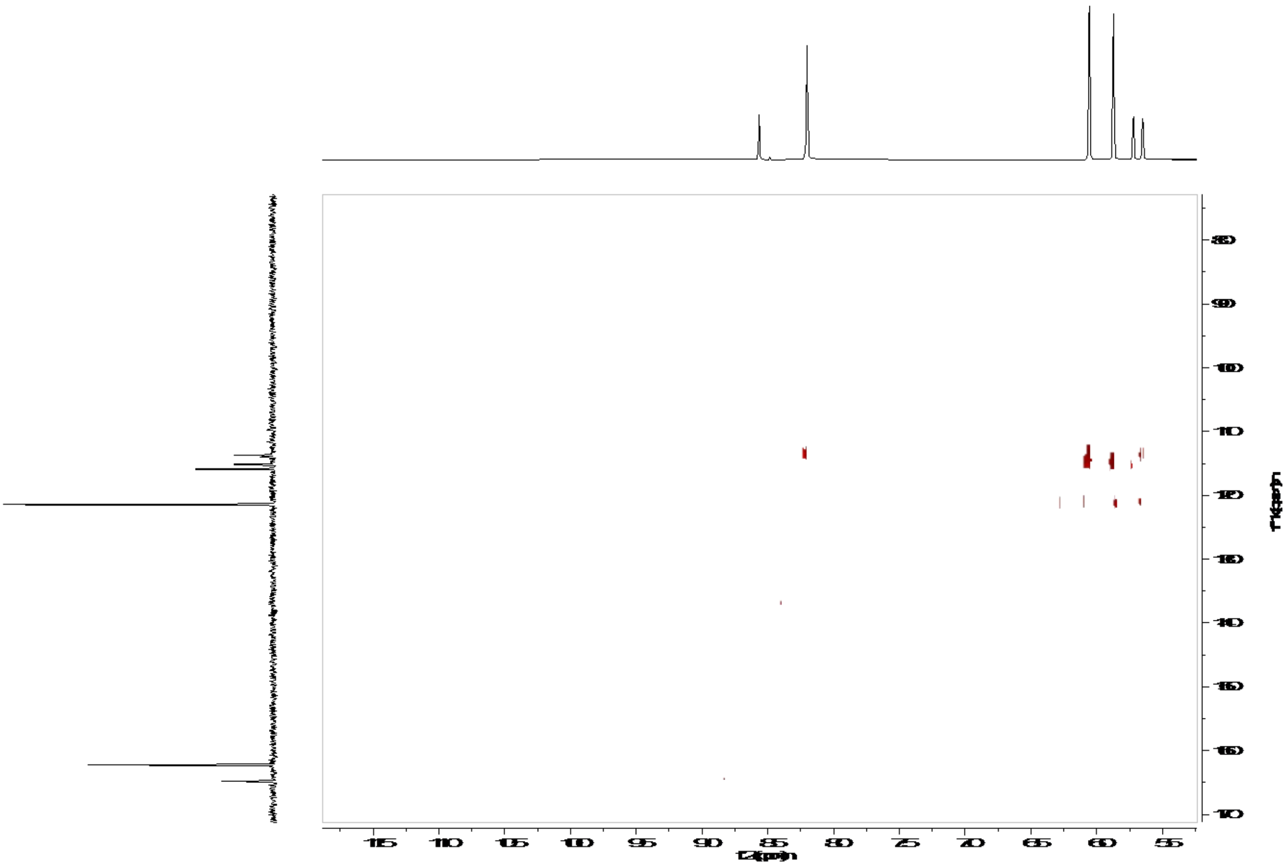

Fig S24. HMBC spectrum of FoDHA-CN in water.

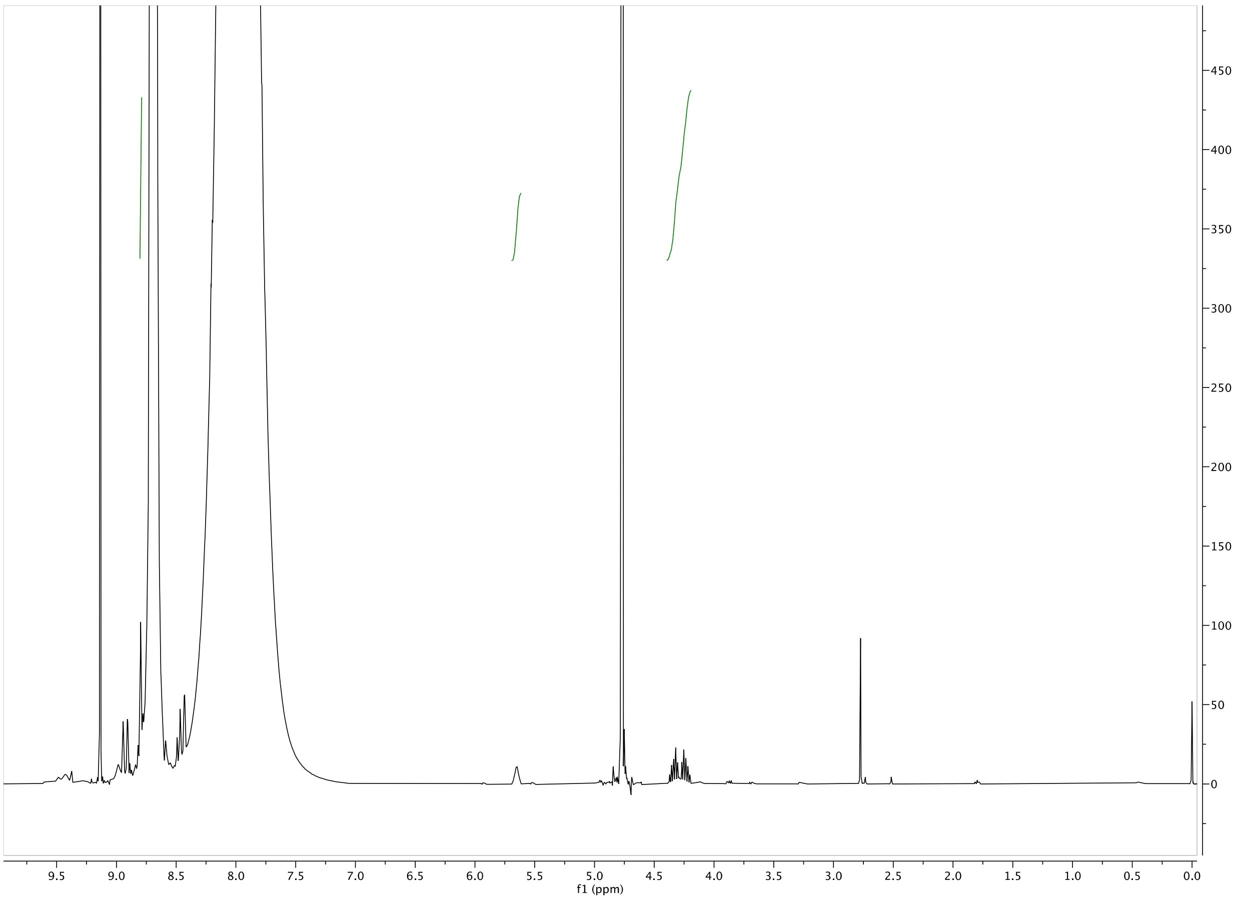

Fig S25. ^{1}H NMR spectrum of Fo(FoNH)Ala-CN in formamide.

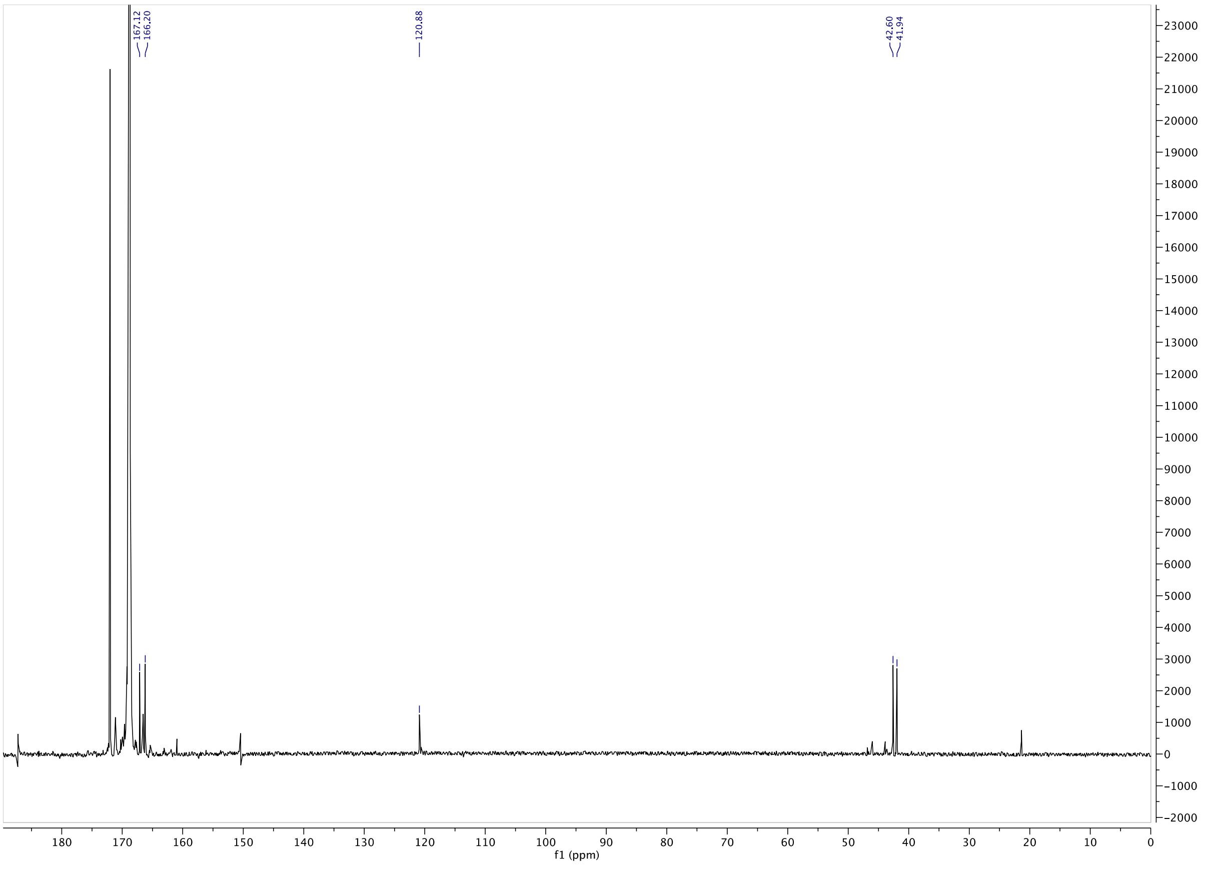

Fig S26. ^{13}C NMR spectrum of Fo(FoNH)Ala-CN in formamide.

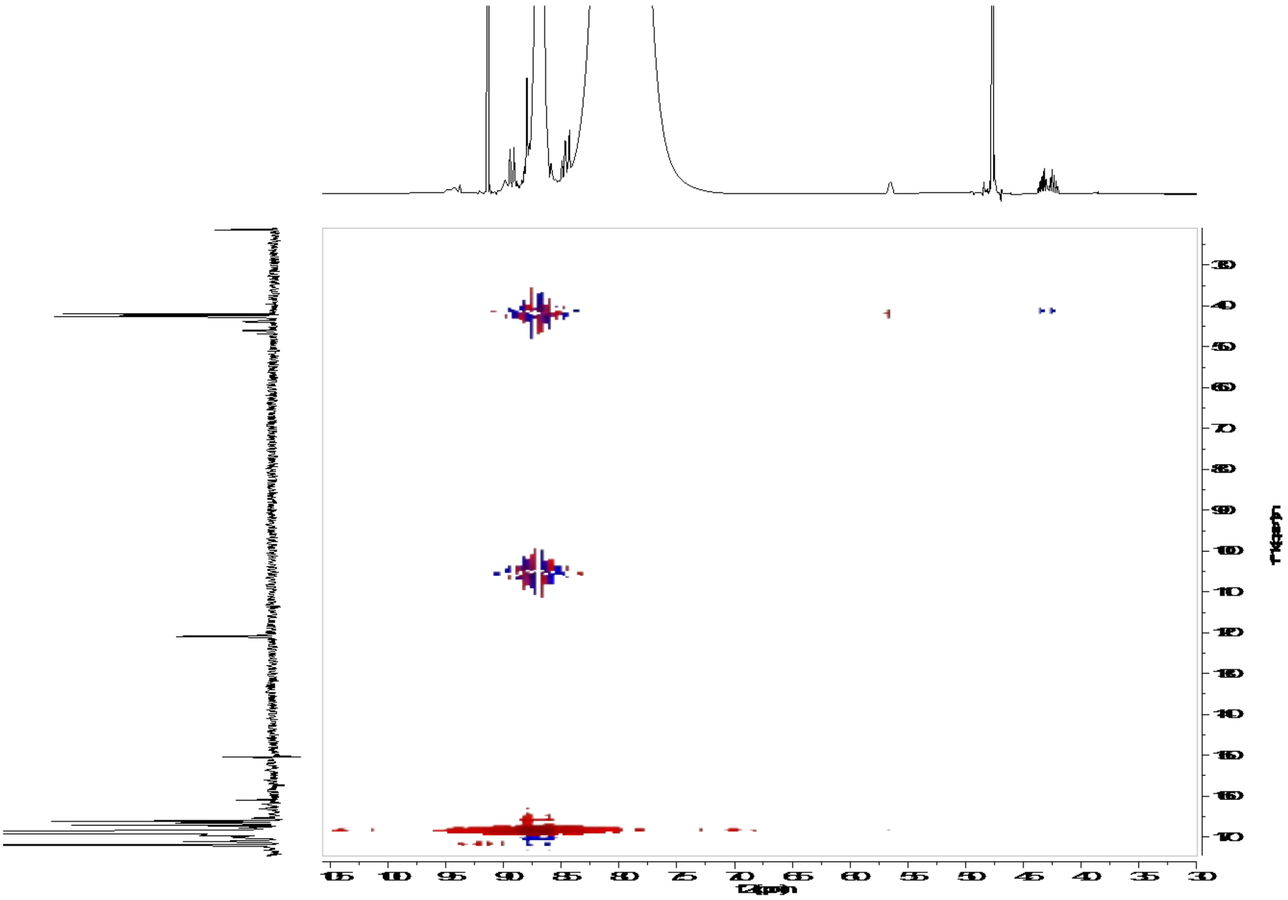

Fig S27. HSQC spectrum of Fo(FoNH)Ala-CN in formamide.

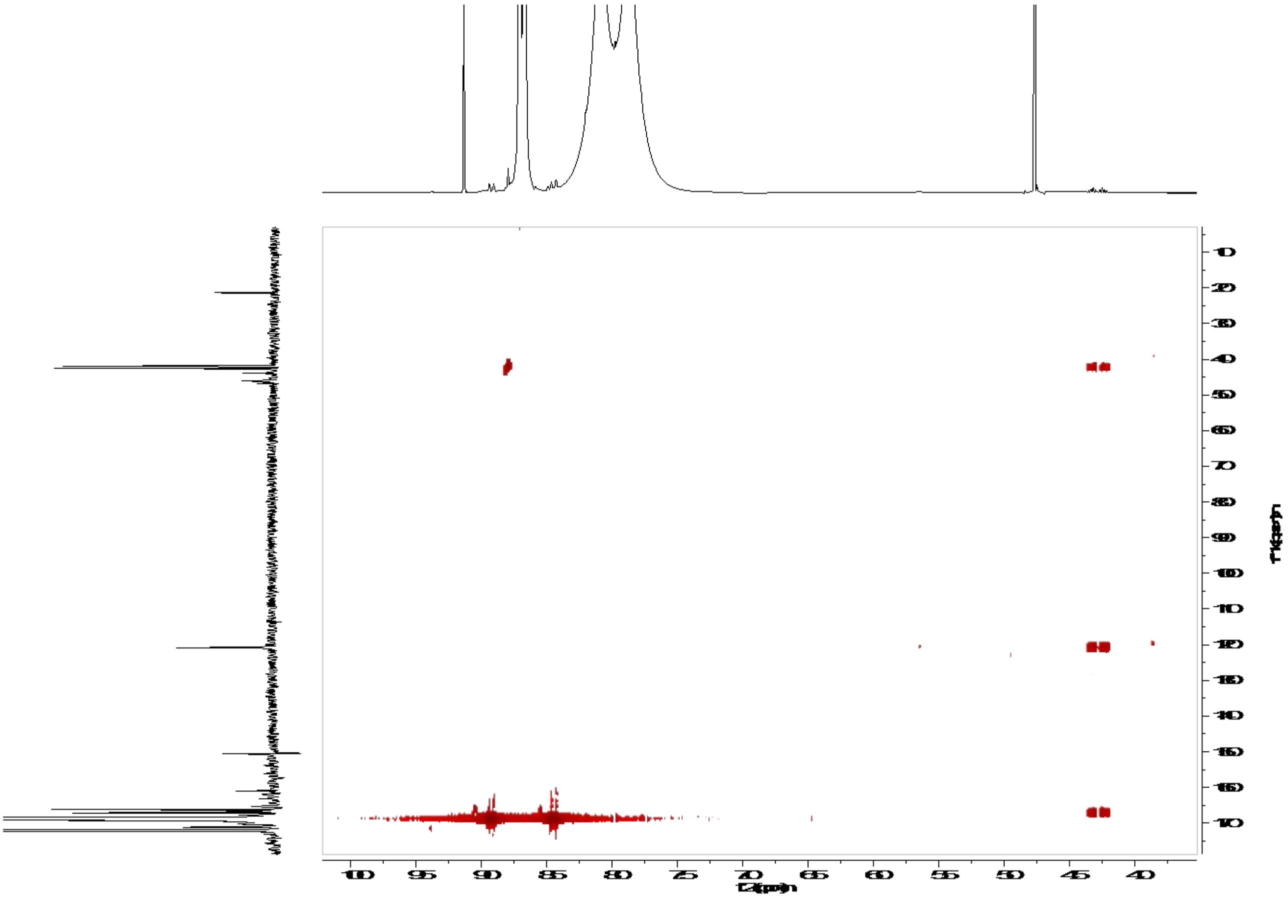

Fig S28. HMBC spectrum of Fo(FoNH)Ala-CN in formamide.

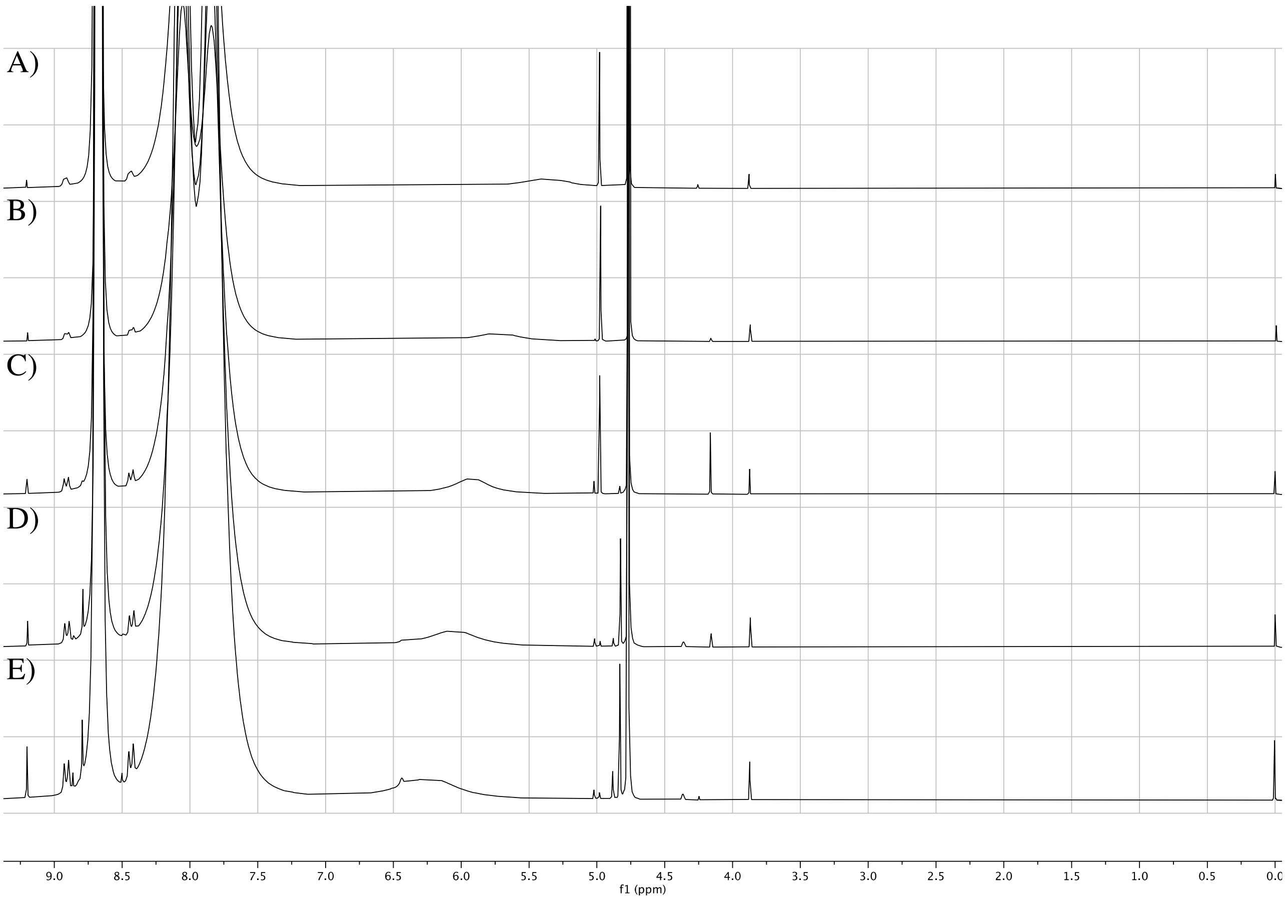

Fig S29. Stack of ^{1}H NMR spectra showing reaction between paraformaldehyde, ammonium chloride (5 eq.) and sodium cyanide (3 eq.) in formamide. A) 0h, RT; B) + 16 h RT; C) +16h 50°C; D) +16 h 80°C; E) +20h 80°C.

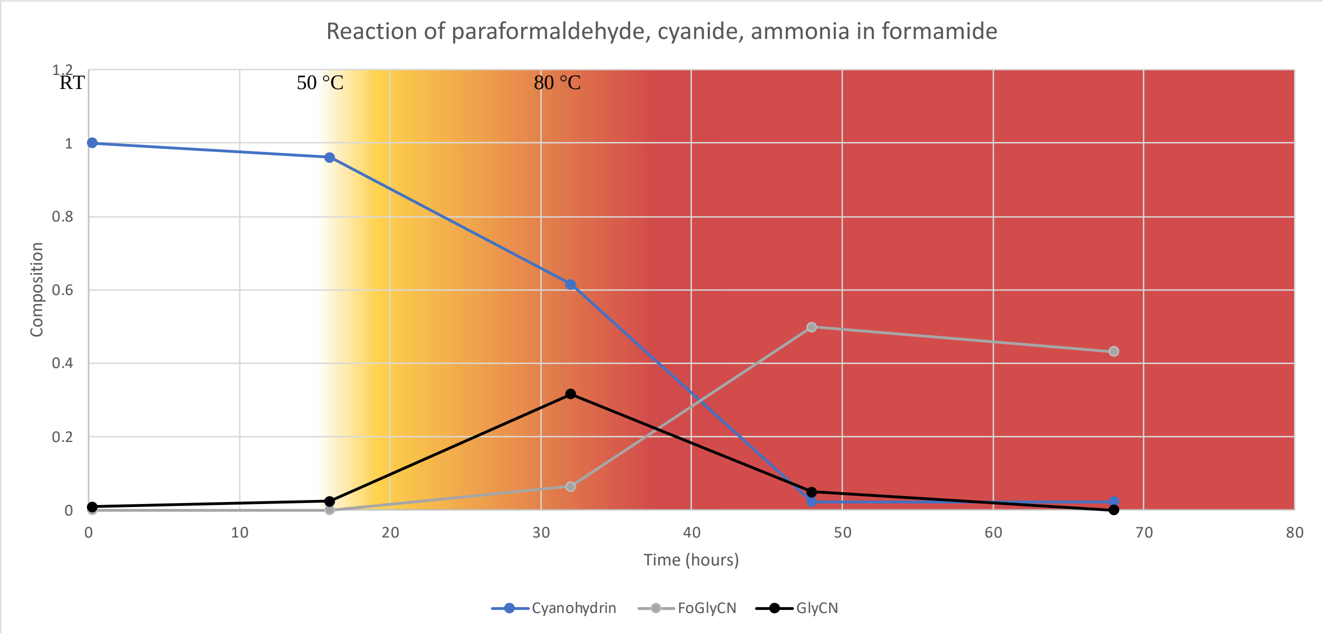

Graph S2. Reaction of paraformaldehyde with ammonium chloride (5eq.) and sodium cyanide (3 eq.) in formamide. Vertical axis is reaction composition as measured by internal standard and ^{1}H NMR.

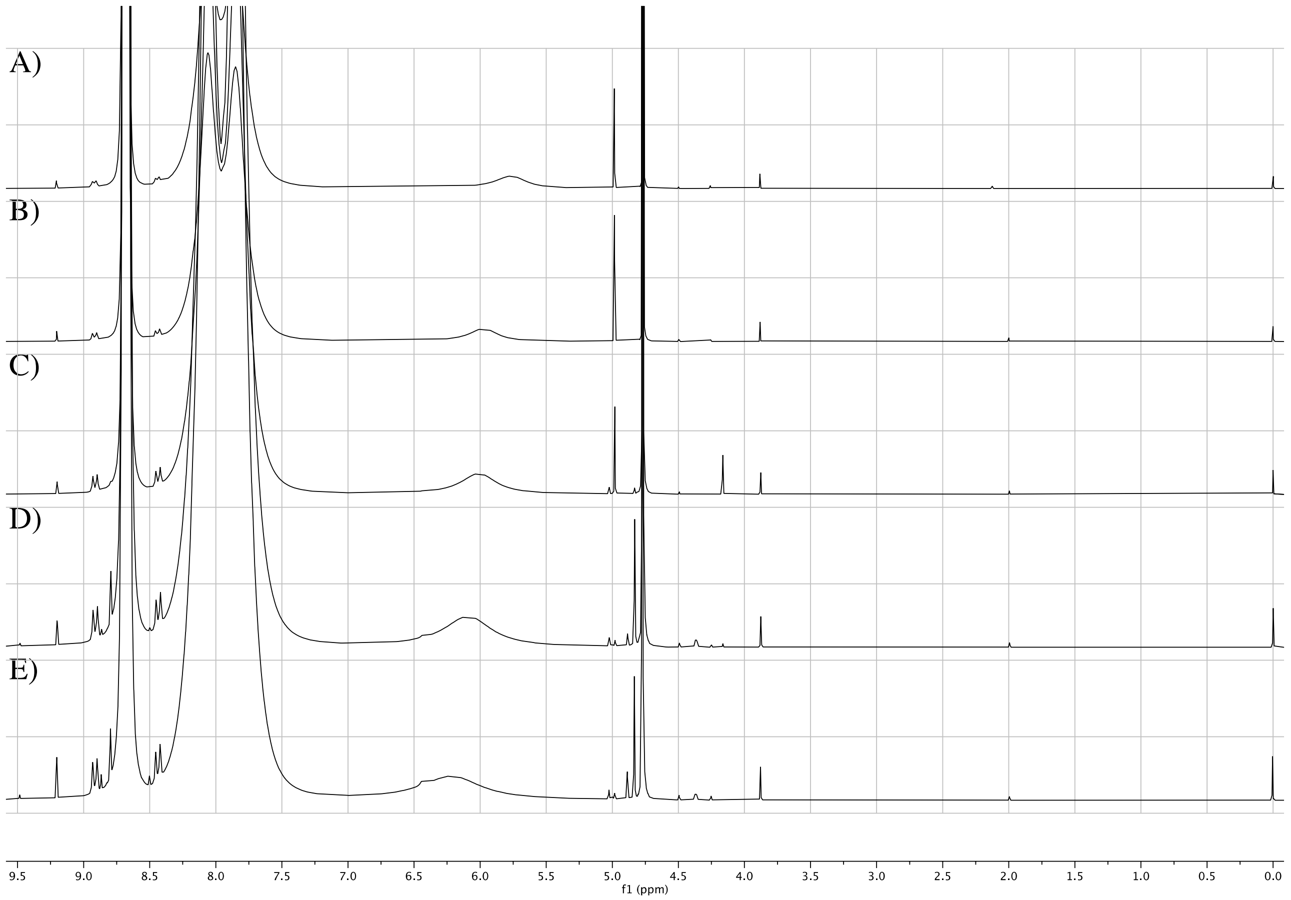

Fig S30. Stack of ^{1}H NMR spectra showing reaction between glycolonitrile, ammonium chloride (5 eq.) and sodium cyanide (2 eq.) in formamide. A) 0h, RT; B) + 16 h RT; C) +16h 50°C; D) +16 h 80°C; E) +20h 80°C.

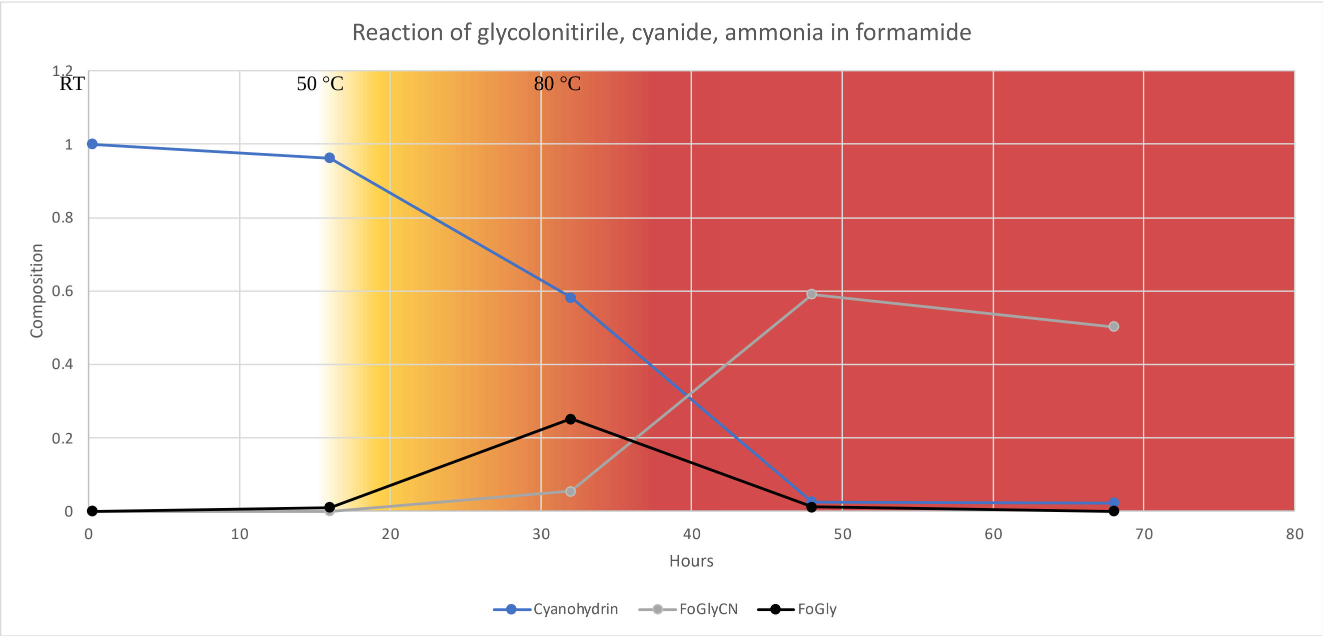

Graph S3. Reaction of glycolonitrile with ammonium chloride (5eq.) and sodium cyanide (2 eq.) in formamide. Vertical axis is reaction composition as measured by internal standard and ^{1}H NMR.

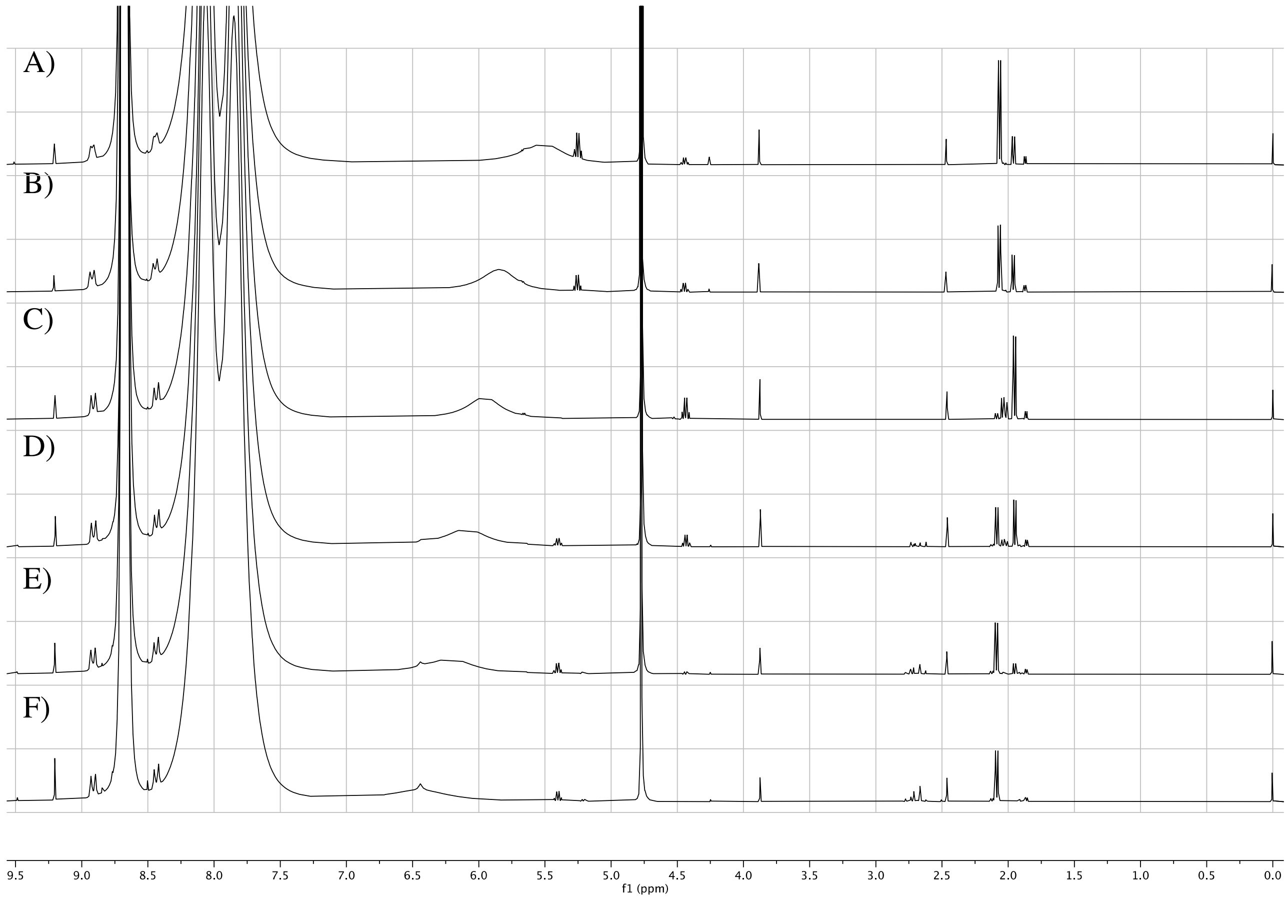

Fig S31. Stack of ^{1}H NMR spectra showing reaction between acetaldehyde, ammonium chloride (5 eq.) and sodium cyanide (2 eq.) in formamide. A) 0h, RT; B) + 16 h RT; C) +16h 50°C; D) +16 h 80°C; E) +20 h 80°C. E) +20 h 80°C.

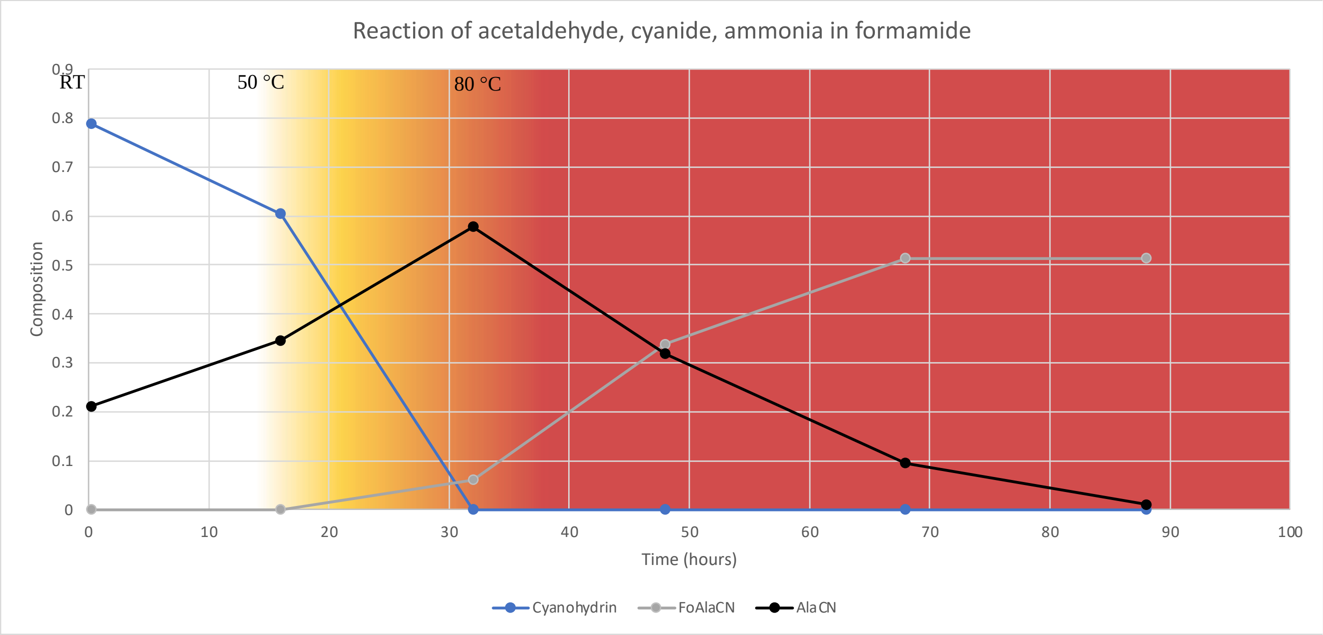

Graph S4. Reaction of acetaldehyde with ammonium chloride (5 eq.) and sodium cyanide (3 eq.) in formamide. Vertical axis is reaction composition as measured by internal standard and ^{1}H NMR.

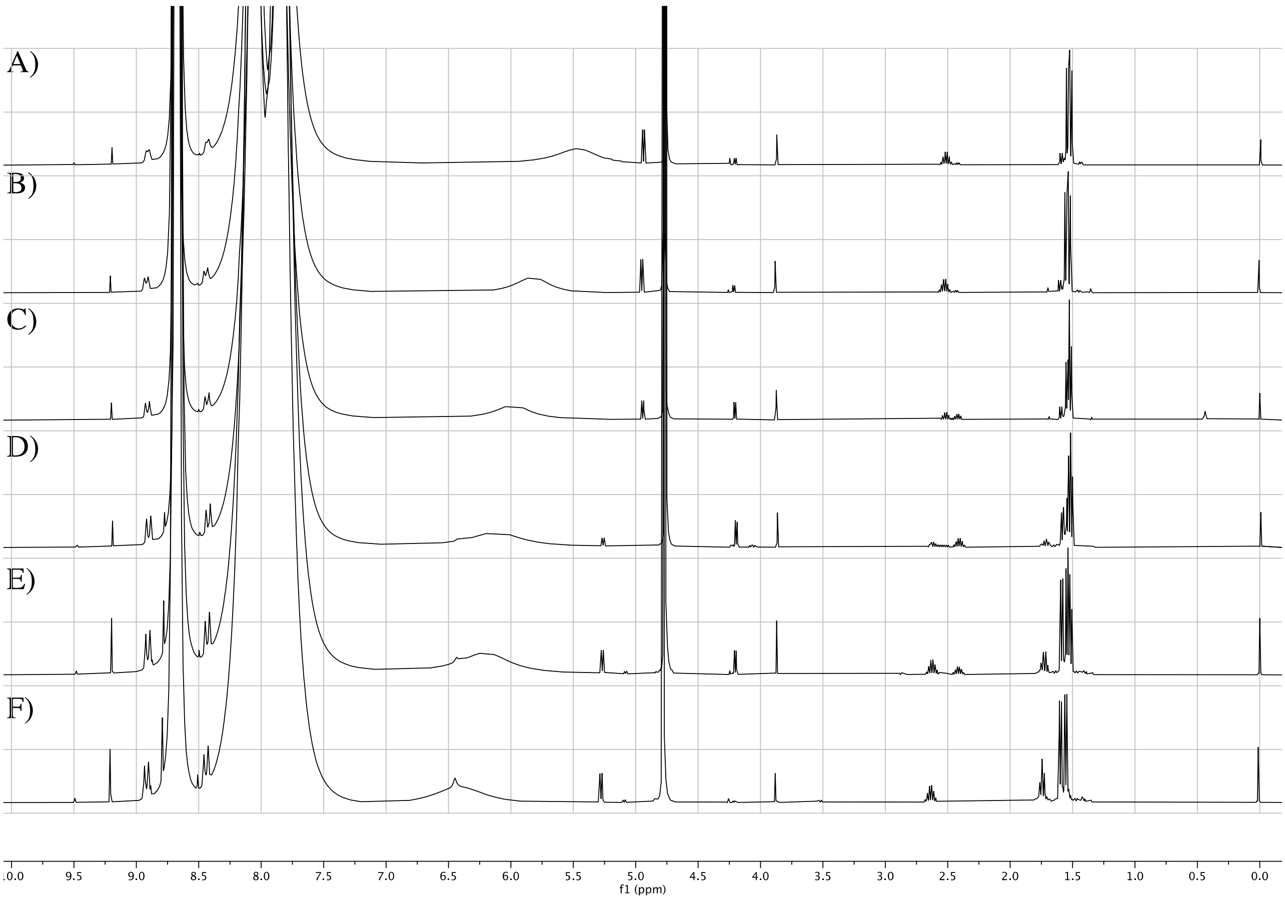

Fig S32. Stack of ^{1}H NMR spectra showing reaction between isopropaldehyde, ammonium chloride (5 eq.) and sodium cyanide (3 eq.) in formamide. A) 0h, RT; B) + 16 h RT; C) +16h 50°C; D) +16 h 80°C; E) +20 h 80°C. F) +44 h 80°C.

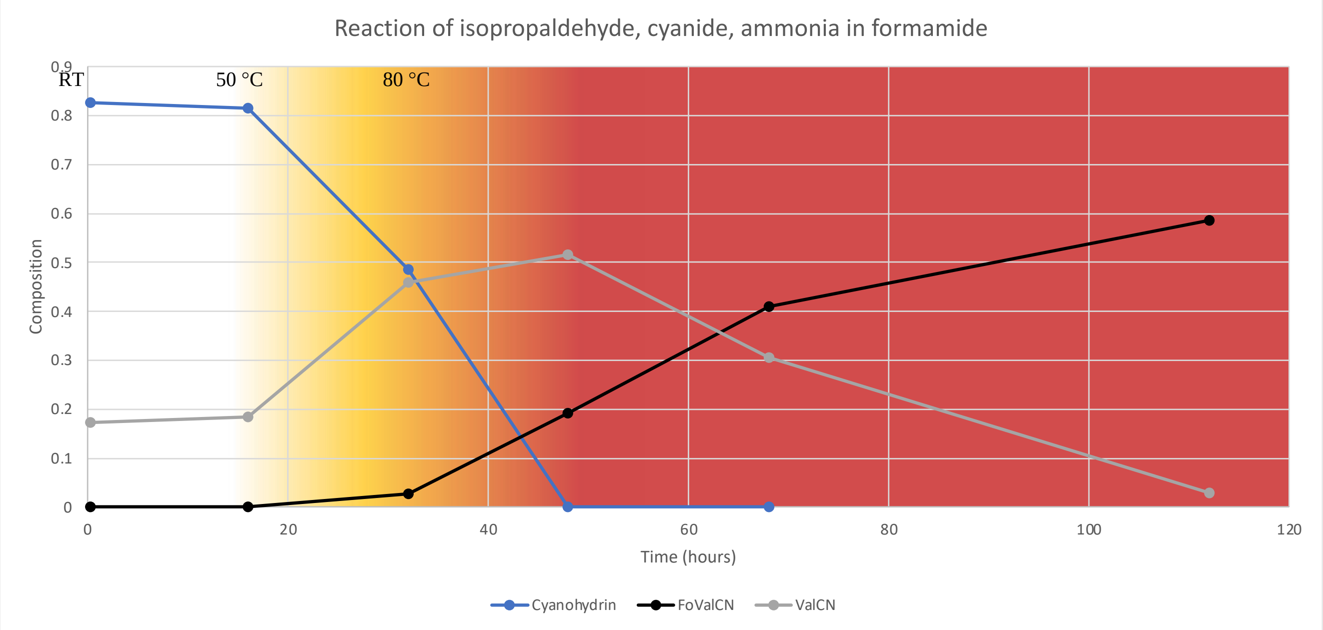

Graph S5. Reaction of isopropaldehyde with ammonium chloride (5eq.) and sodium cyanide (3 eq.) in formamide. Vertical axis is reaction composition as measured by internal standard and ^{1}H NMR.

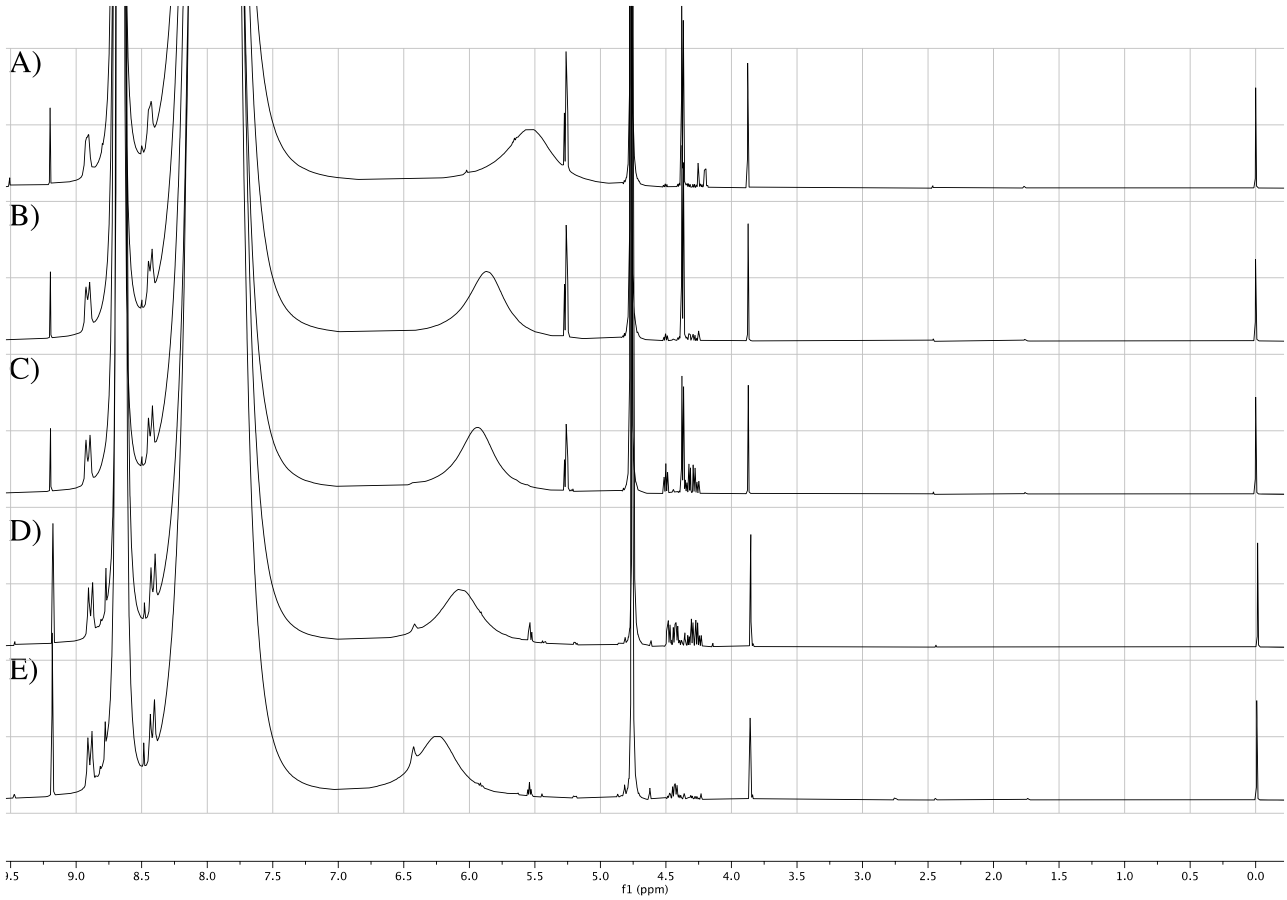

Fig S33. Stack of ^{1}H NMR spectra showing reaction between glycolaldehyde, ammonium chloride (5 eq.) and sodium cyanide (3 eq.) in formamide. A) 0h, RT; B) + 16 h RT; C) +16h 50°C; D) +16 h 80°C; E) +16 h 80°C.

Table S1. Additional reactions between glycolaldehyde, sodium cyanide, and various additives in formamide, at 60 degrees Celsius.

Entry	Additive, equivalents					FoDHA-CN yield		
	NaCN	KH_2PO4	$NH_4H_2PO_4$	FA	NH_4Cl	4d	11d	31d
**A**	1.5			10% (v/v)		-	-	-
**B**	5			10% (v/v)		-	-	-
**C**	15			10% (v/v)		trace	trace	trace
**D**	1.5			10% (v/v)		-	-	-
**E**	1.5			10% (v/v)		-	-	-
**F**	1.5	2.0			0	4%	4%	2%
**G**	1.5	2.0			0.1	3-5%	4%	3%
**H**	1.5	2.0		10% (v/v)		-	-	-
**I**	1.5		0.5			trace	2%	3%
**J**	1.5				0.1	trace	trace	2%
**K**	1.5				0.5	trace	trace	trace

FA = formic acid

Table S2. Additional reactions between glycolaldehyde, sodium cyanide (1.5 eq.), and various additives in formamide, at 80 degrees Celsius, 16 h.

Entry	NH_4X, X = , eq.	Additive	FoDHA-CN observed?	Outcome
**A1**	H_2PO_4, 5		Yes	FoSerCN, Fo(β-FoNH)Ala-CN major
**A2**	H_2PO_4, 5	H_2O 20% (v/v)	Yes	FoSerCN, Fo(β-FoNH)Ala-CN major
**B1**	NH_4HPO_4, 5		Trace	FoSerCN, Fo(β-FoNH)Ala-CN major
**B2**	NH_4HPO_4, 5	H_2O 20% (v/v)	Trace	FoSerCN, Fo(β-FoNH)Ala-CN major
**C1**	OFo 10% (w/v)		Trace	FoSerCN, Fo(β-FoNH)Ala-CN major
**C2**	OFo 10% (w/v)	H_2O 20% (v/v)	Trace	FoSerCN, Fo(β-FoNH)Ala-CN major
**D1**	NH_4HPO_4, 5; OFo 10% (w/v)		Trace	FoSerCN, Fo(β-FoNH)Ala-CN major
**D2**	NH_4HPO_4, 5; OFo 10% (w/v)	H_2O 20% (v/v)	trace	FoSerCN, Fo(β-FoNH)Ala-CN major
**E1**	--	FA, 10% (v/v)	Yes	Minimal FoSerCN and Fo(β-FoNH)Ala-CN
**E2**	--	H_2O 20% (v/v)	No	Fo(β-FoNH)Ala-CN major
**F1**	--	K_3PO_4 (5 eq.) FoOH 10%	Yes, major	Minimal FoSerCN and Fo(β-FoNH)Ala-CN
**F2**	--	H_2O 20% (v/v)	Trace	Fo(β-FoNH)Ala-CN major

FA = formic acid, OFo = formate

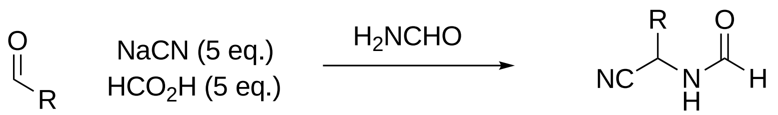

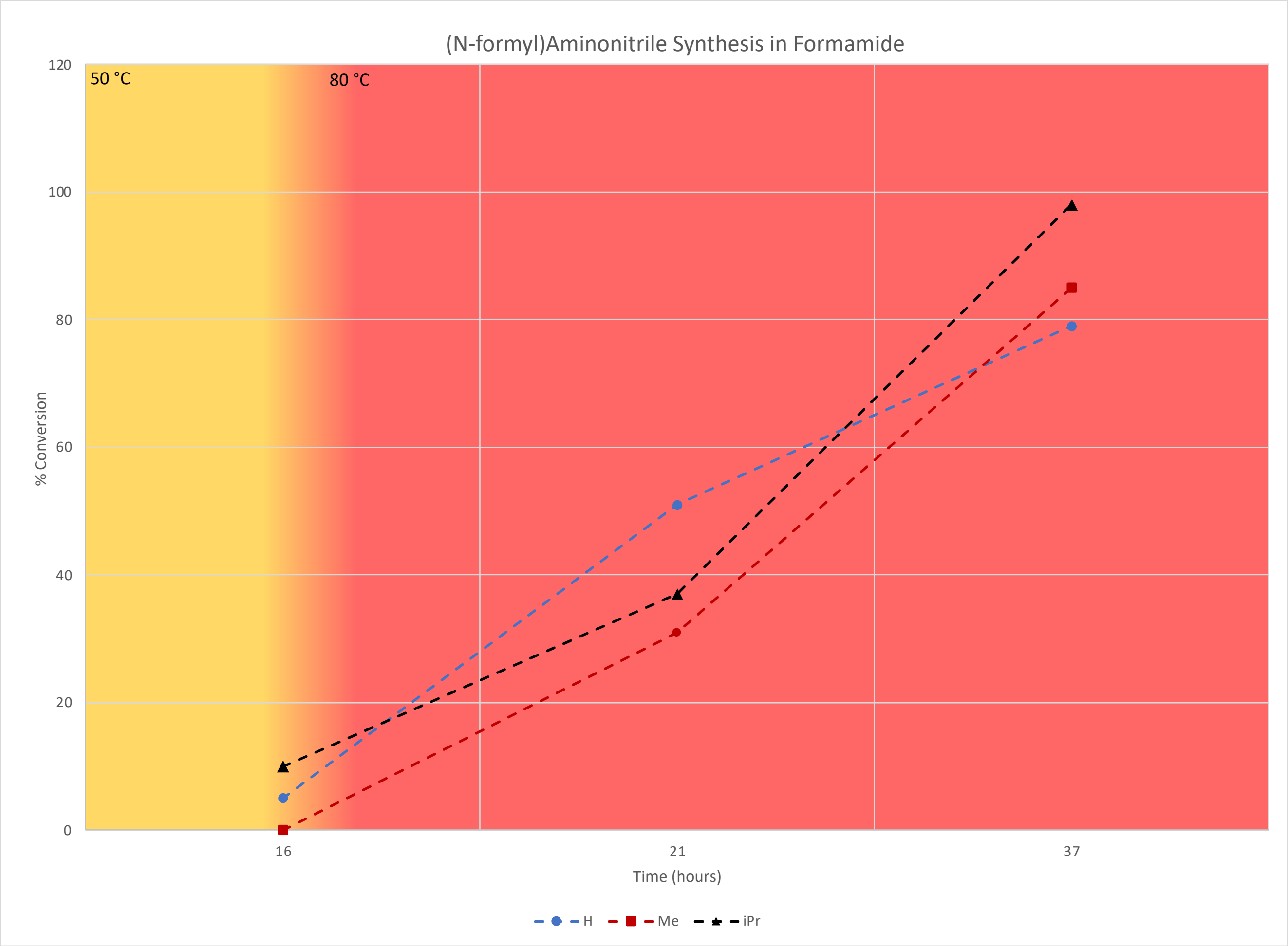

Graph S6. Formation of (N-formyl)aminonitriles in formamide from aldehydes and cyanide.

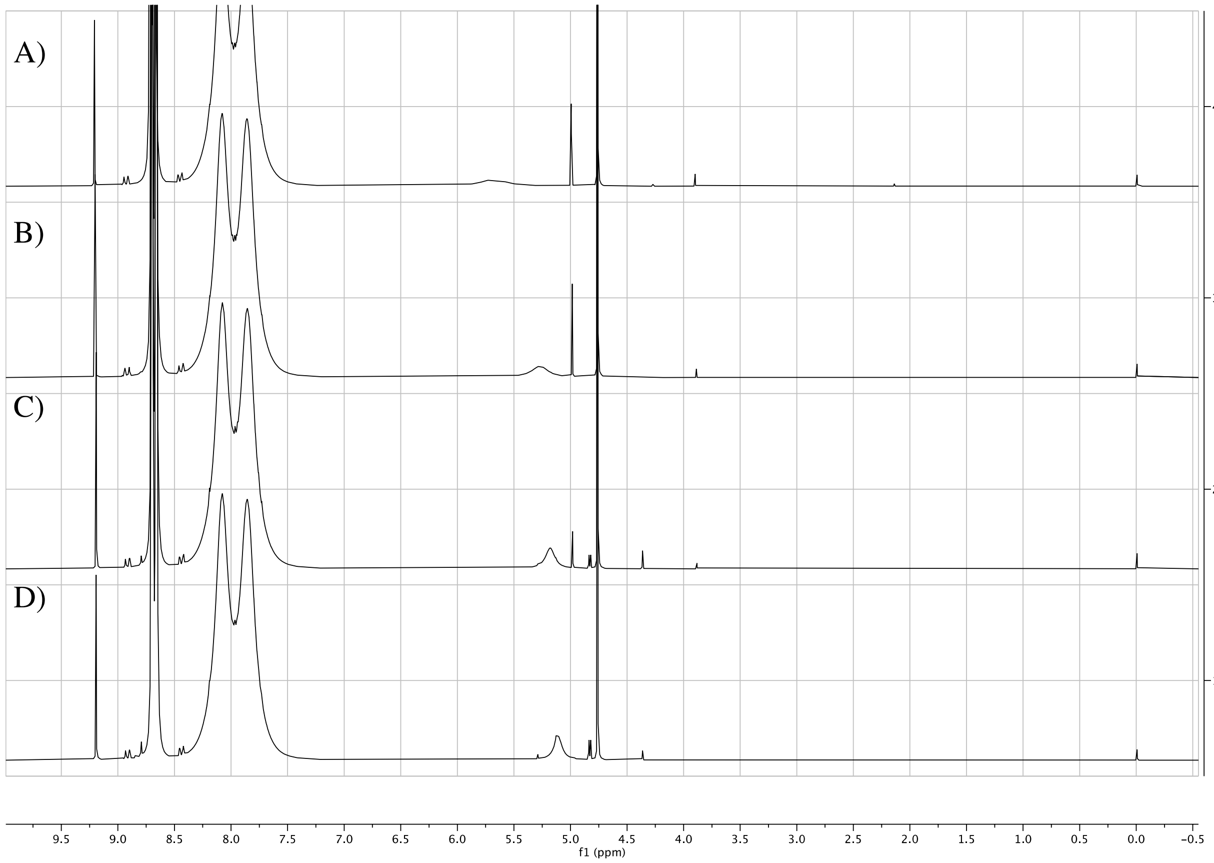

Fig S34. Stack of ^{1}H NMR spectra showing conversion of a mixture of formaldehyde, sodium cyanide and formic acid in formamide to FoGlyCN. A) 1h, RT; B) + 16 h 50°C; C) +5h 80°C; D) +16 h 80°C.

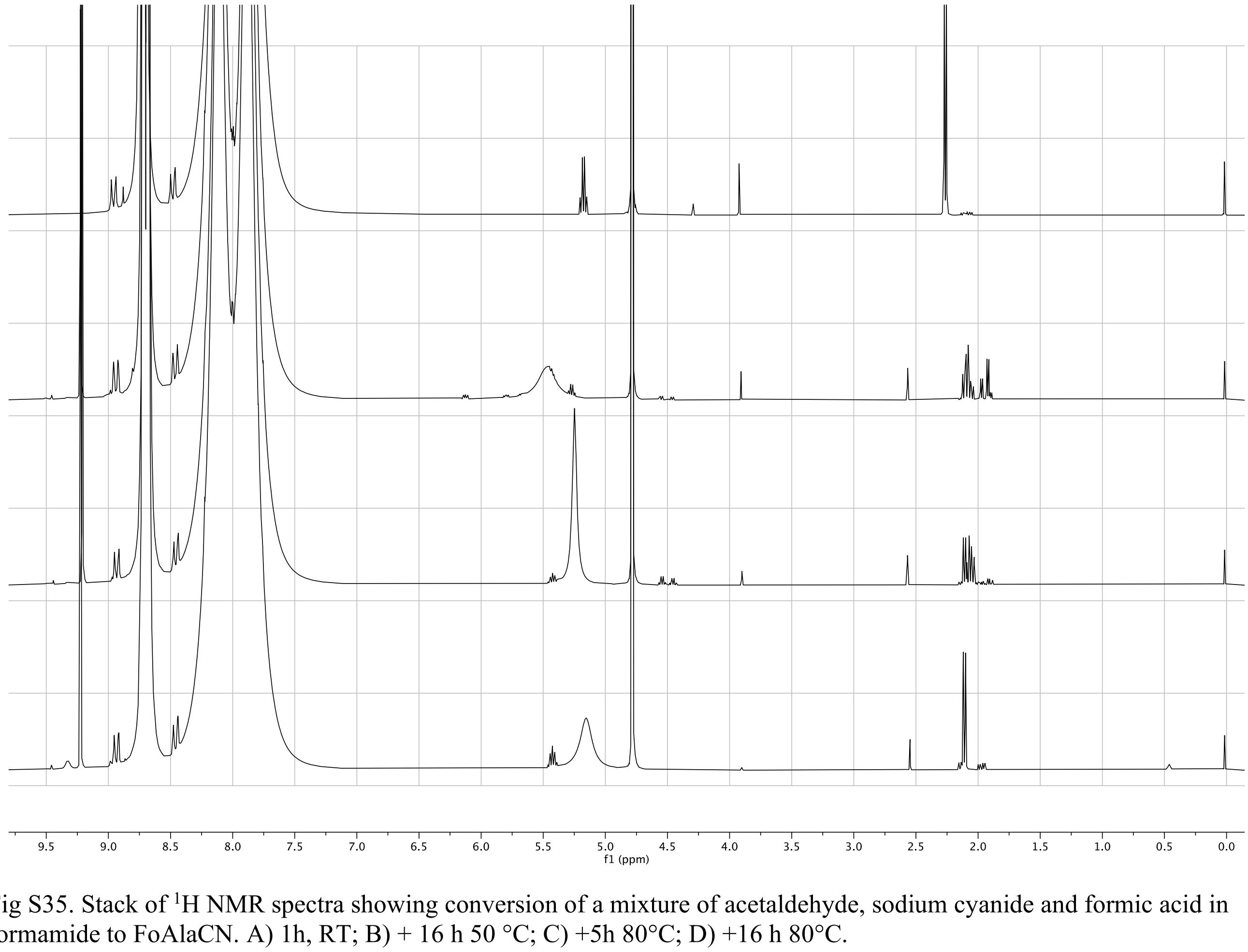

Fig S35. Stack of ^{1}H NMR spectra showing conversion of a mixture of acetaldehyde, sodium cyanide and formic acid in formamide to FoAlaCN. A) 1h, RT; B) + 16 h 50 °C; C) +5h 80°C; D) +16 h 80°C.

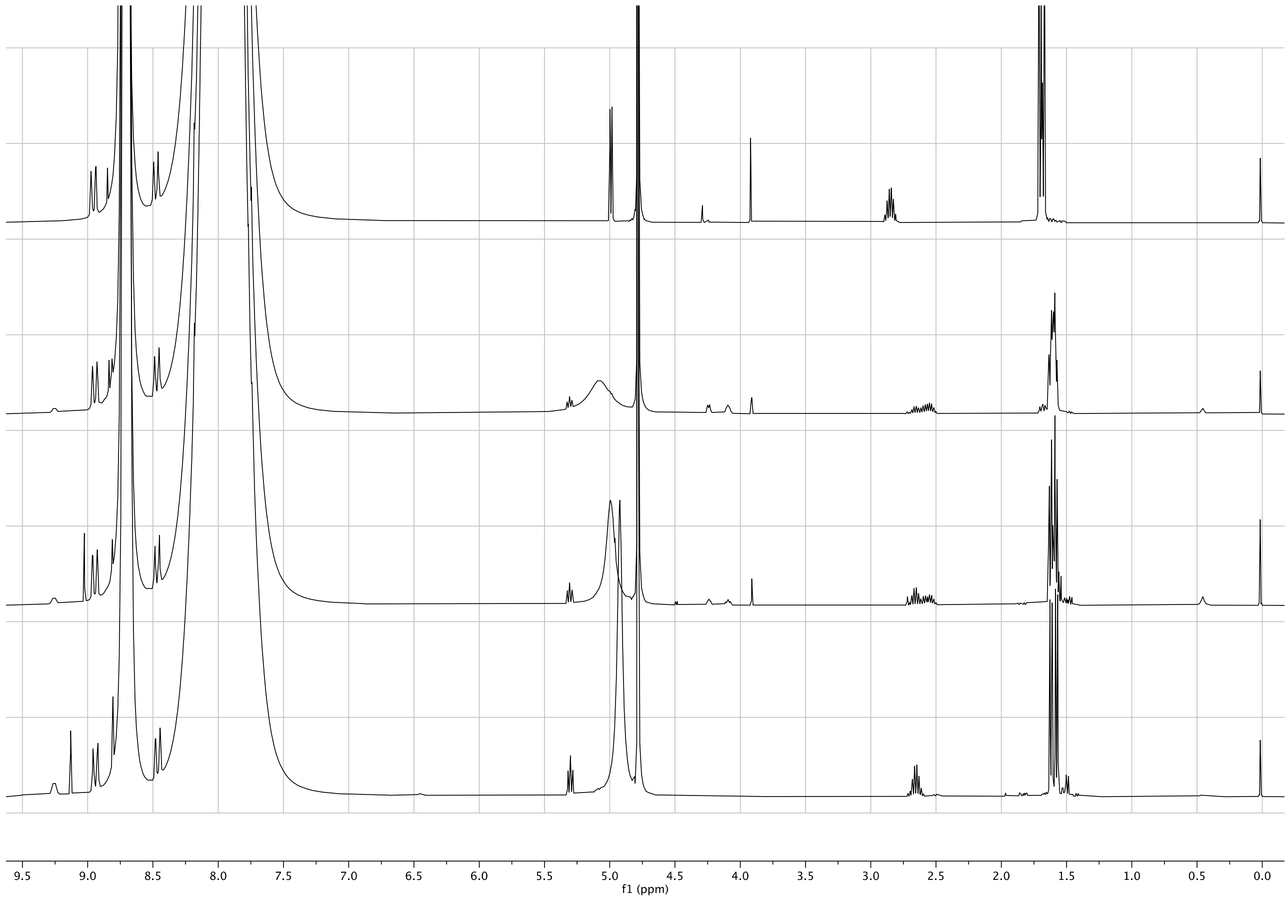

Fig S36. Stack of ^{1}H NMR spectra showing conversion of a mixture of isopropaldehyde, sodium cyanide, and formic acid in formamide to FoValCN. A) 1h, RT; B) + 16 h 50°C; C) +5h 80°C; D) +16 h 80°C.

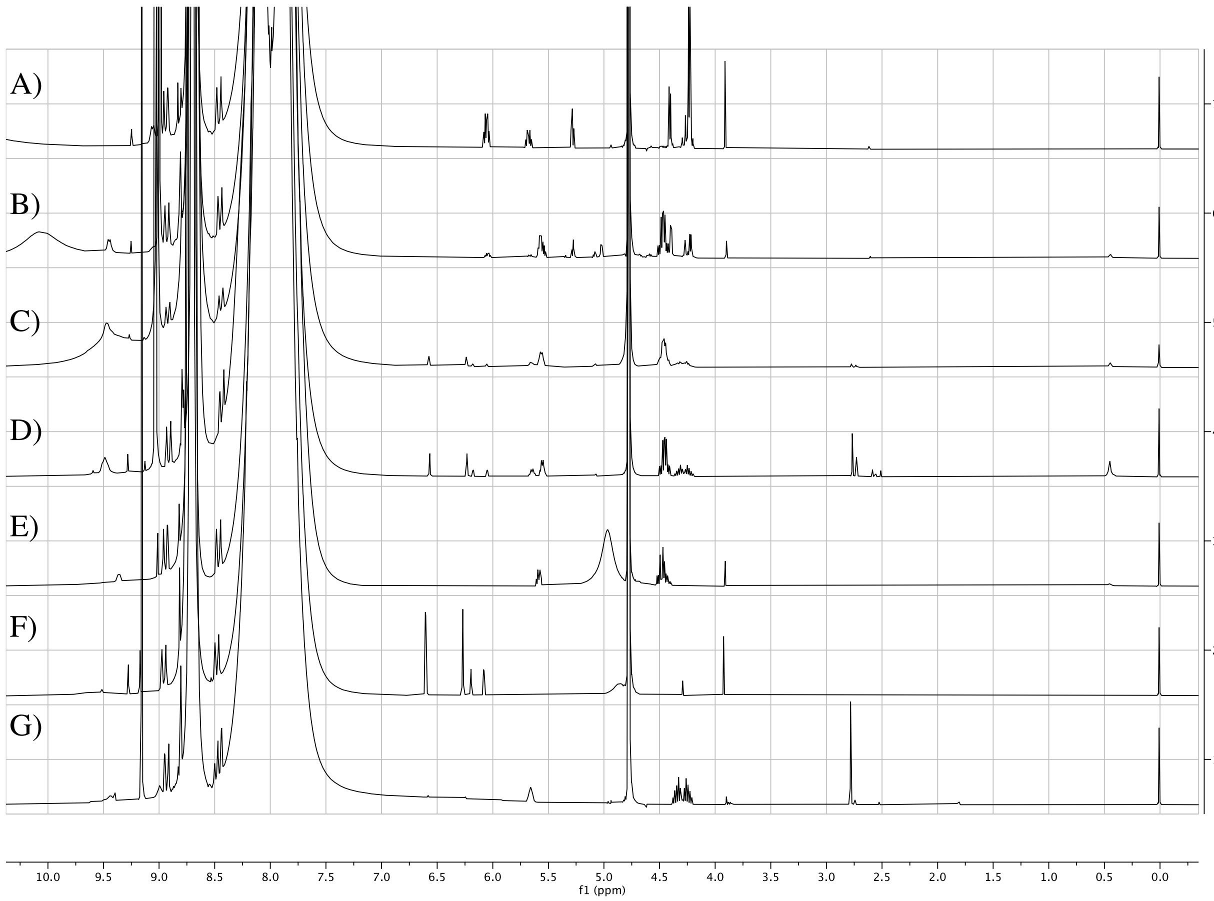

Fig S37. Stack of ^{1}H NMR spectra showing conversion of a mixture of glycolaldehyde, NaCN, formic acid, and $MgCl_2$ in formamide to FoSerCN, FoDHA-CN, and Fo-NH_2-Ala-CN. A) 1h RT; B) +4h 80° C; C) +12h 80°C; D) +20h 80°C; E) Fo-Ser-CN standard in formamide; F) Fo-DHA-CN standard in formamide; G) Fo(FoNH)Ala-CN standard in formamide.

Synthetic preparation of FoDHA-CN

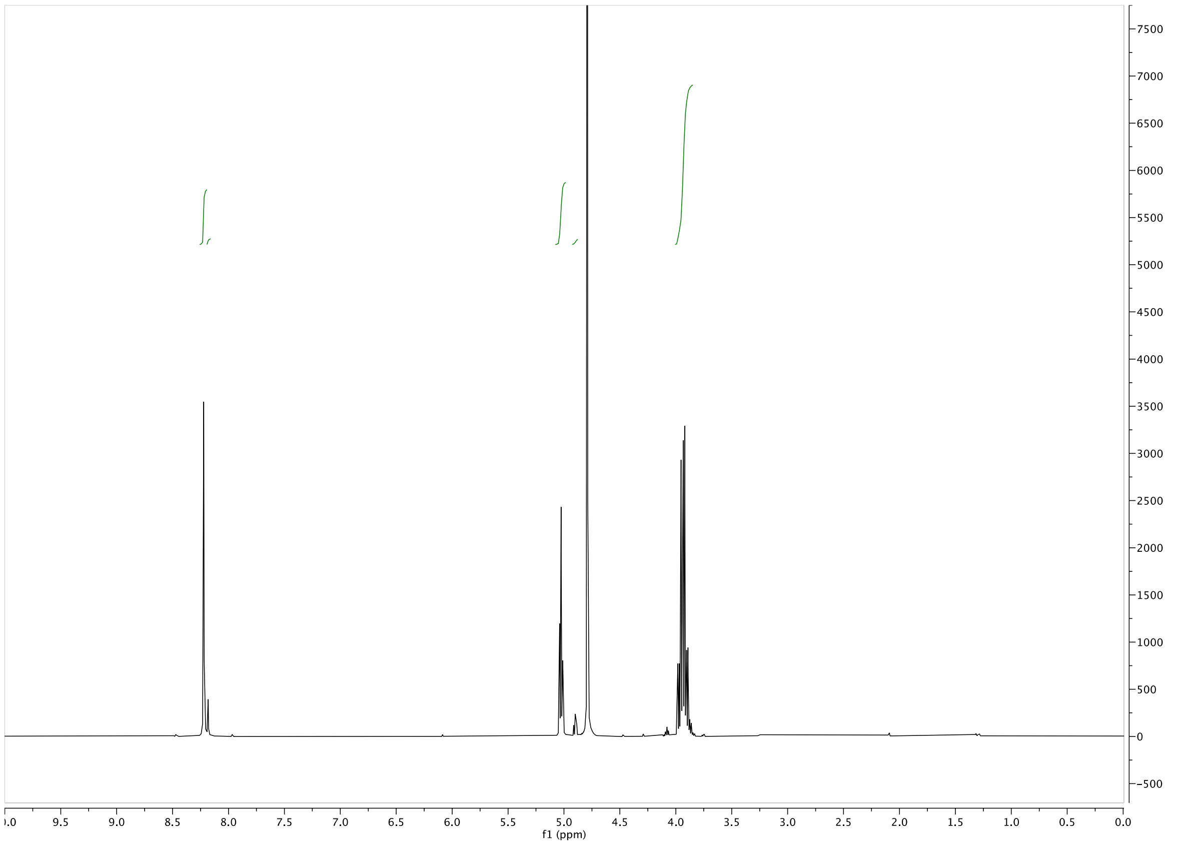

Fig S38. ^{1}H NMR spectrum of FoSer-CN in water.

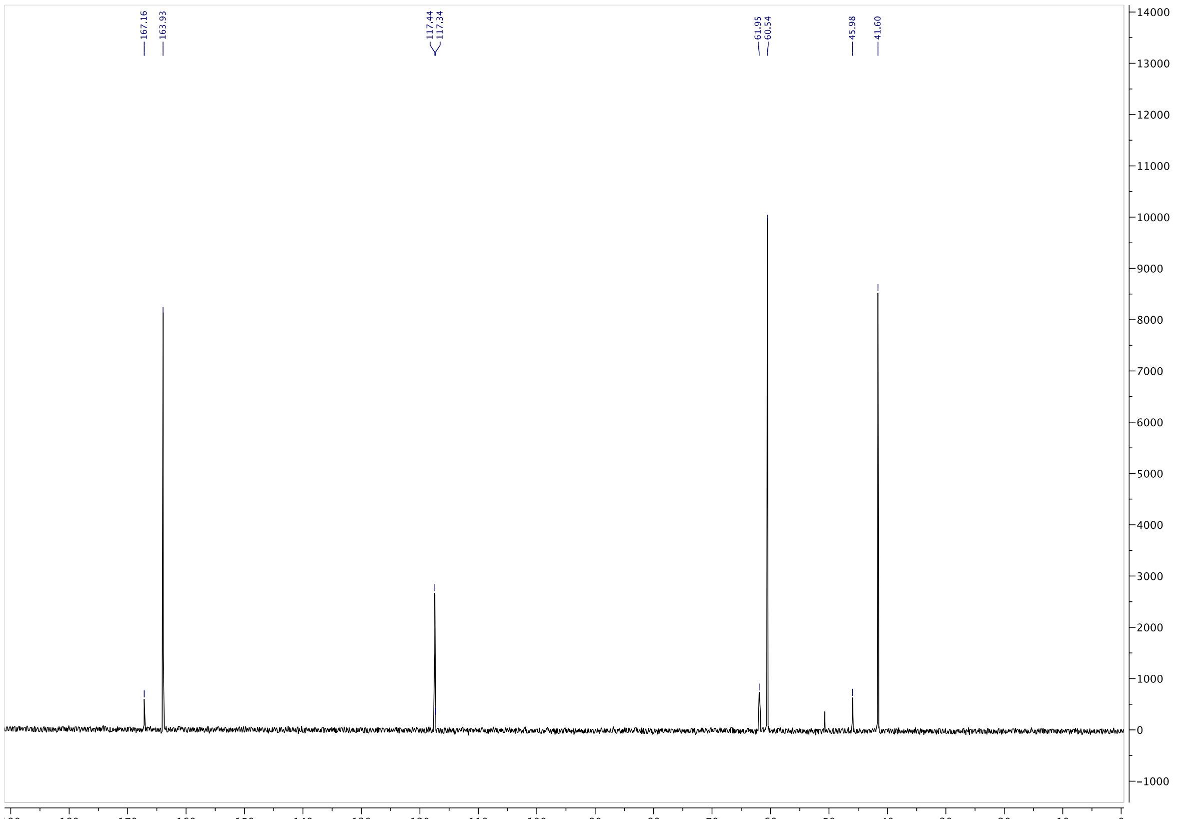

Fig S39. ^{13}C NMR spectrum of FoSer-CN in water.

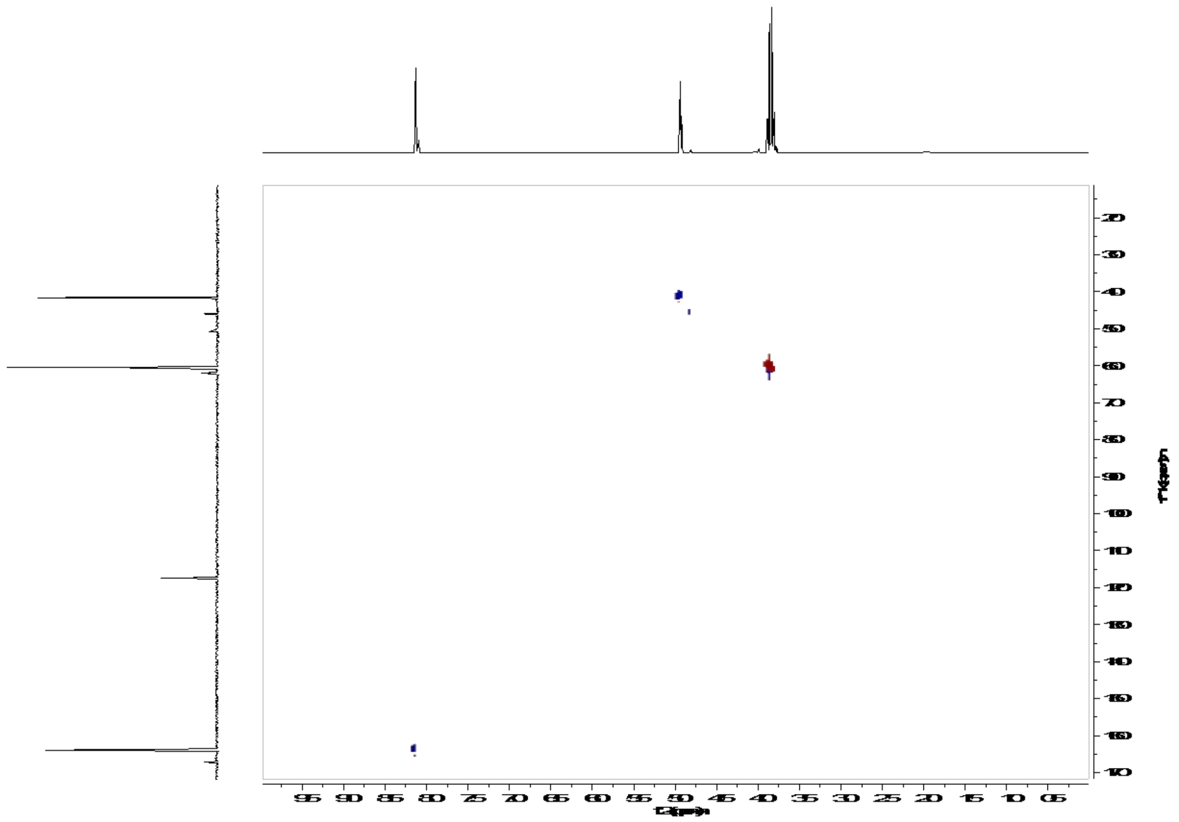

Fig S40. HSQC spectrum of FoSer-CN in water.

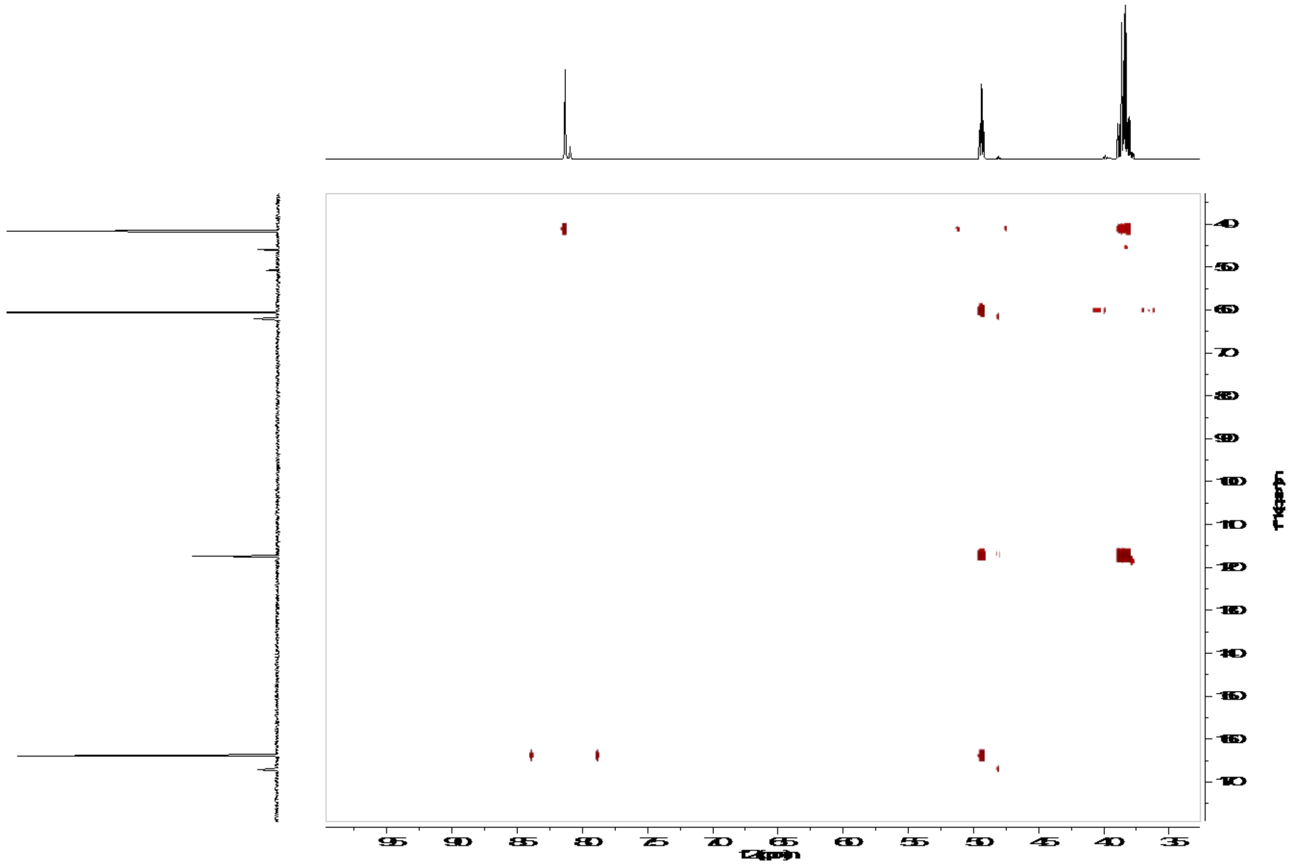

Fig S41. HMBC spectrum of FoSer-CN in water.

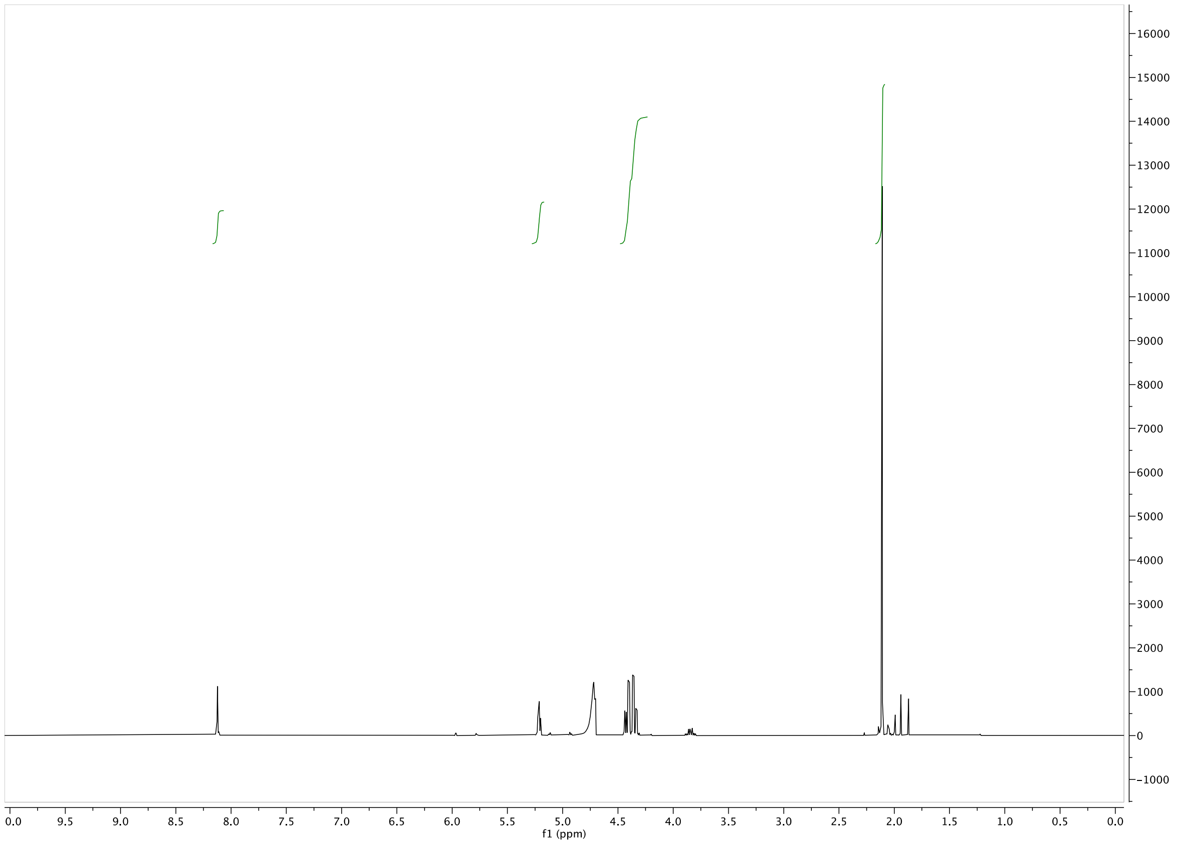

Fig S42. ^{1}H NMR spectrum of FoSer(Ac)-CN in water.

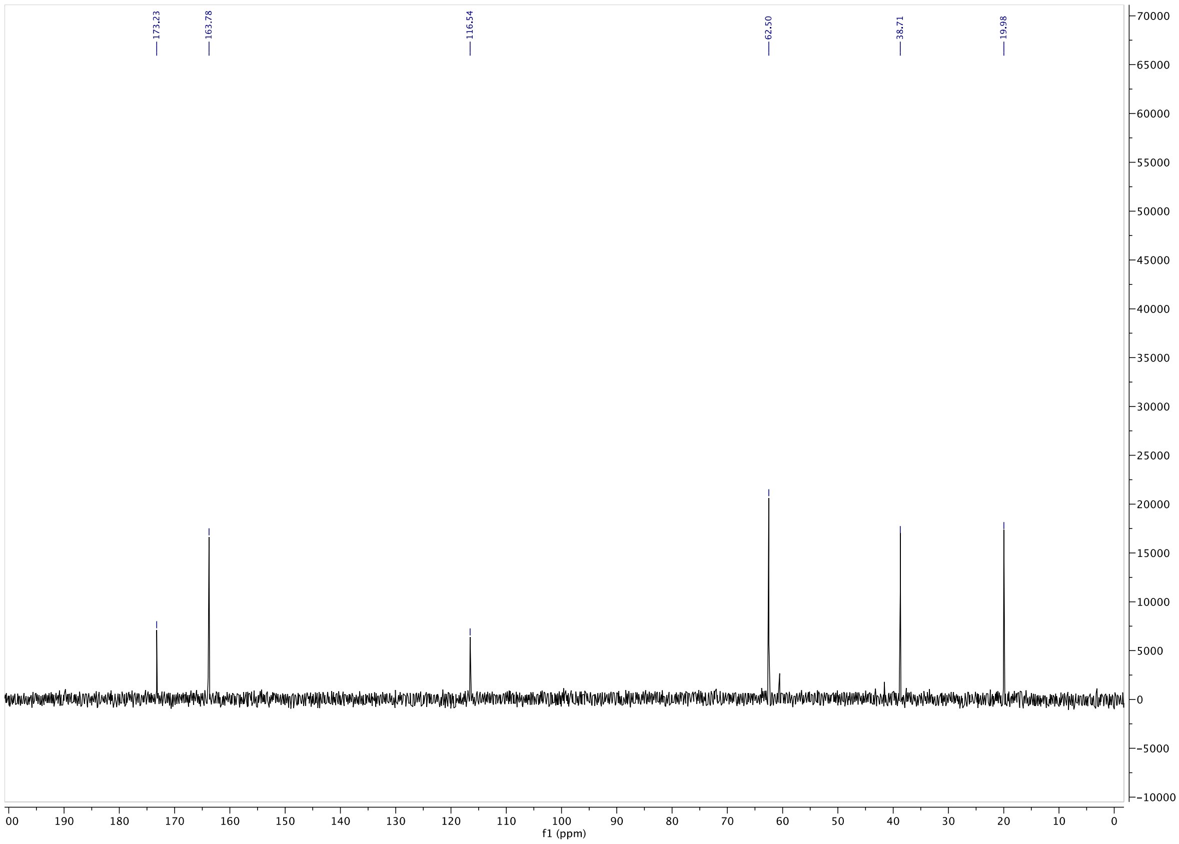

Fig S43. ^{13}C NMR spectrum of FoSer(Ac)-CN in water.

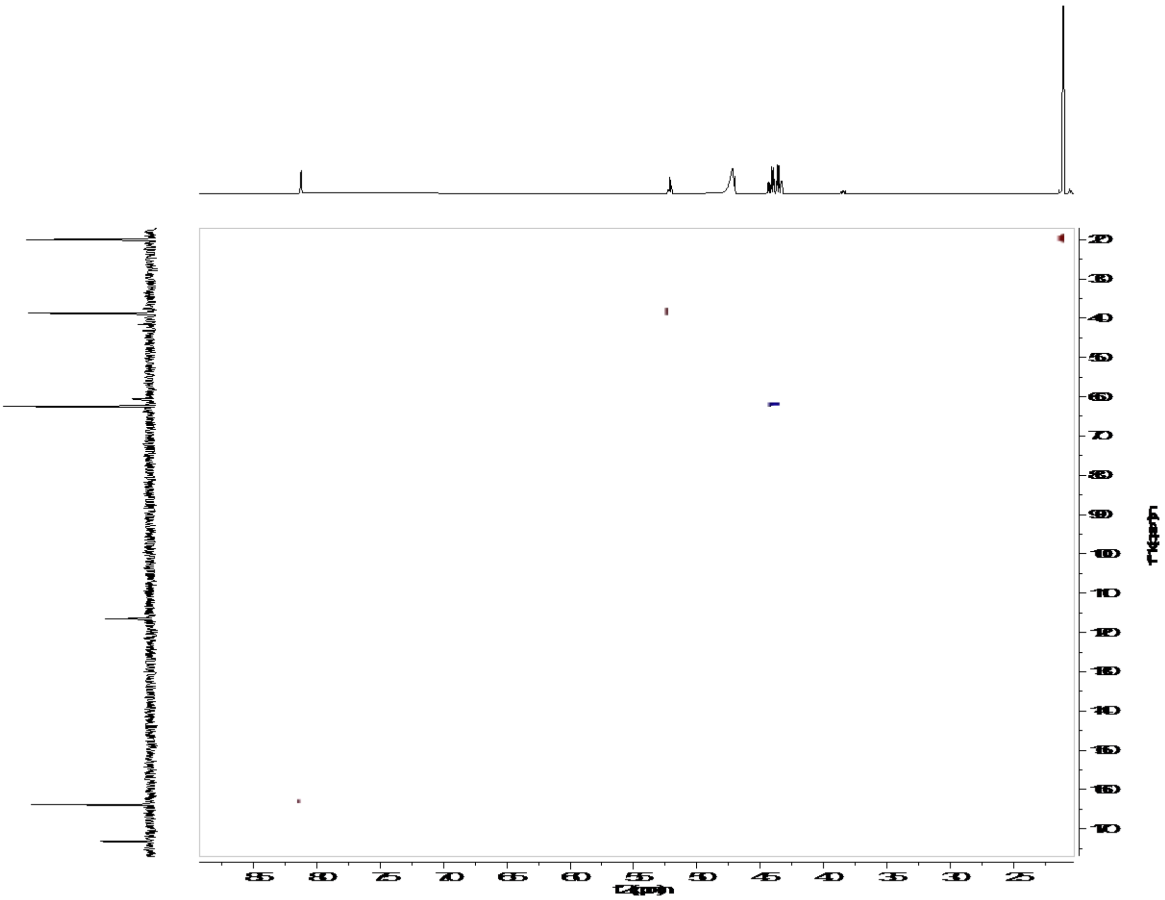

Fig S44. HSQC spectrum of FoSer(Ac)-CN in water.

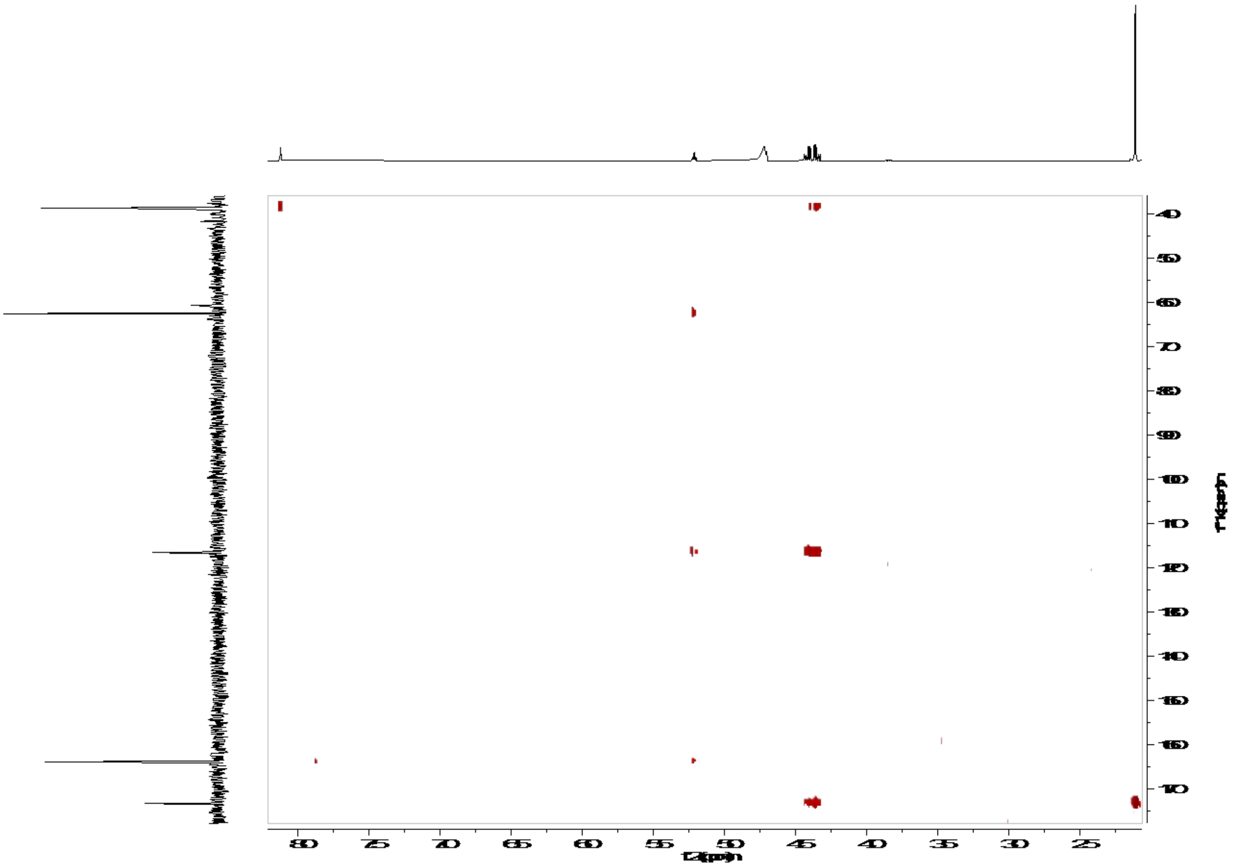

Fig S45. HMBC spectrum of FoSer(Ac)-CN in water.

Formyl amino acid derivatives

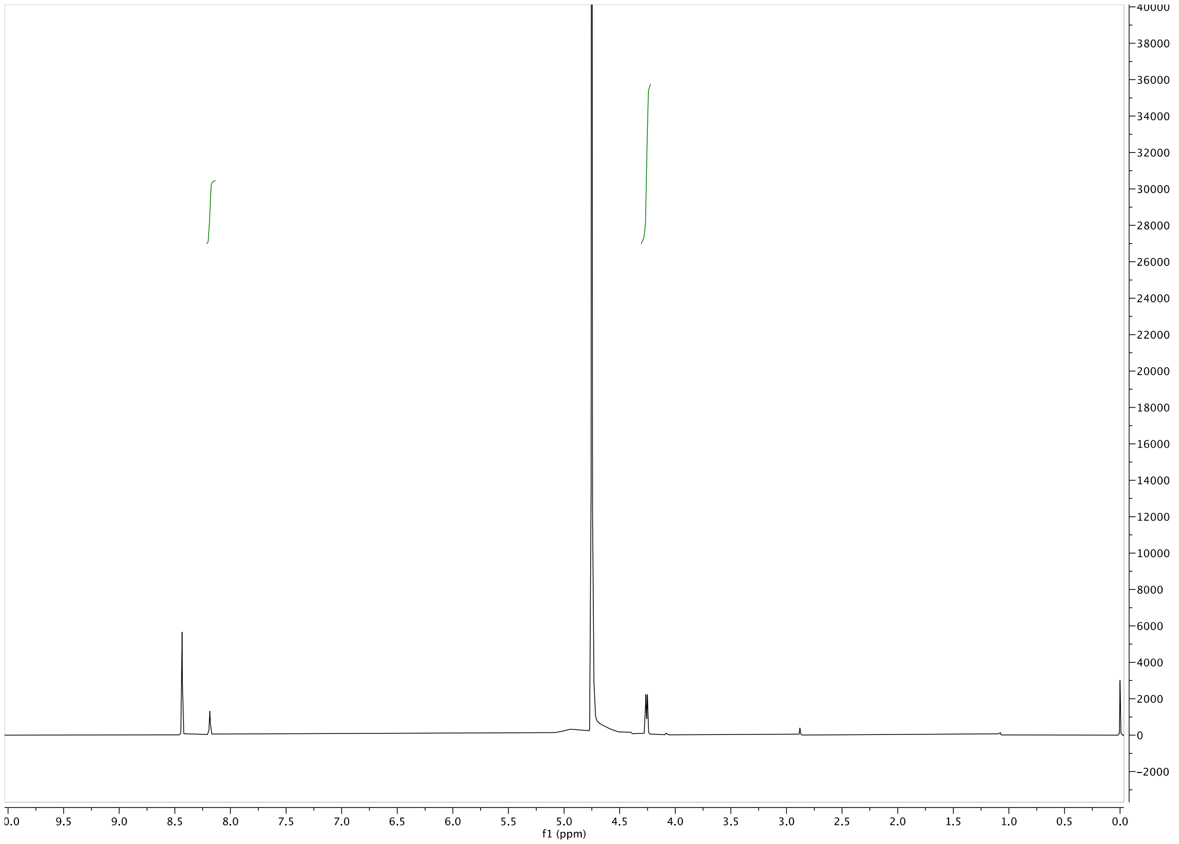

Fig S46. ^{1}H NMR spectrum of FoGly-CN in water.

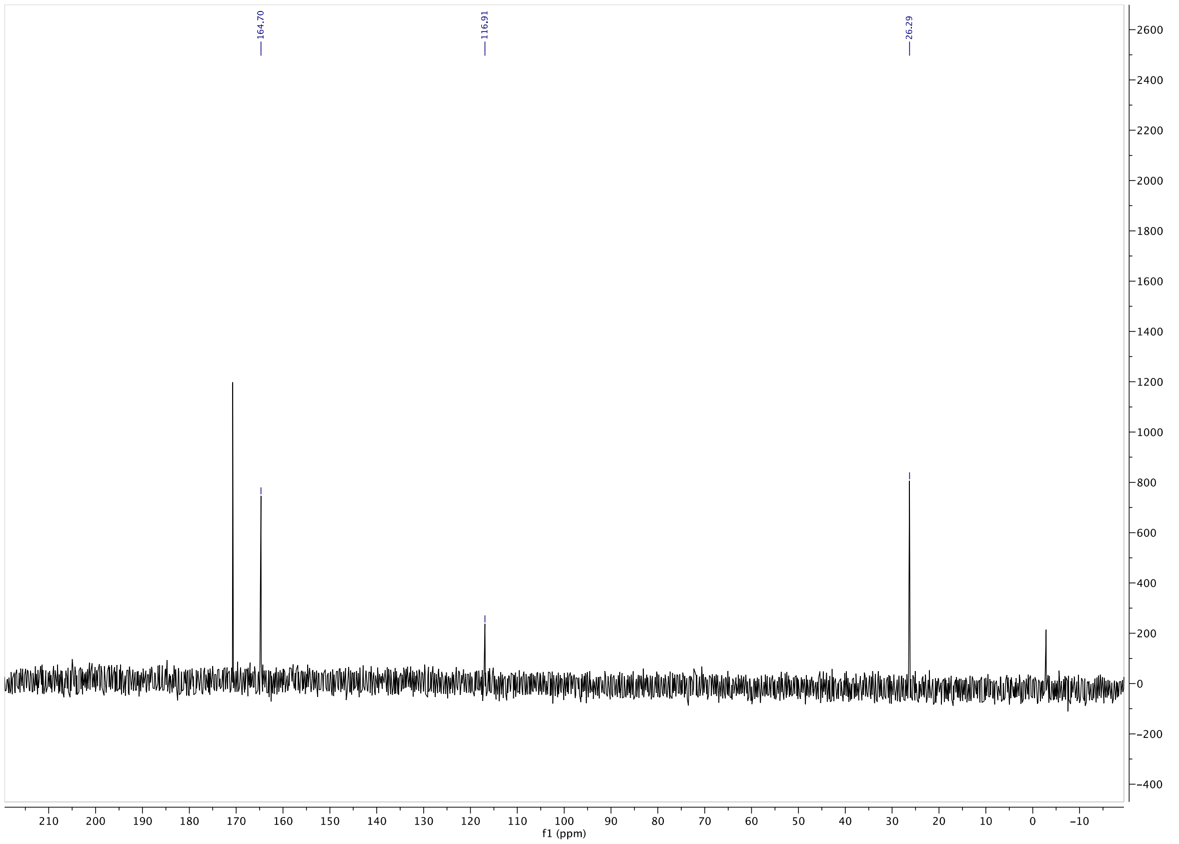

Fig S47. ^{13}C NMR spectrum of FoGly-CN in water.

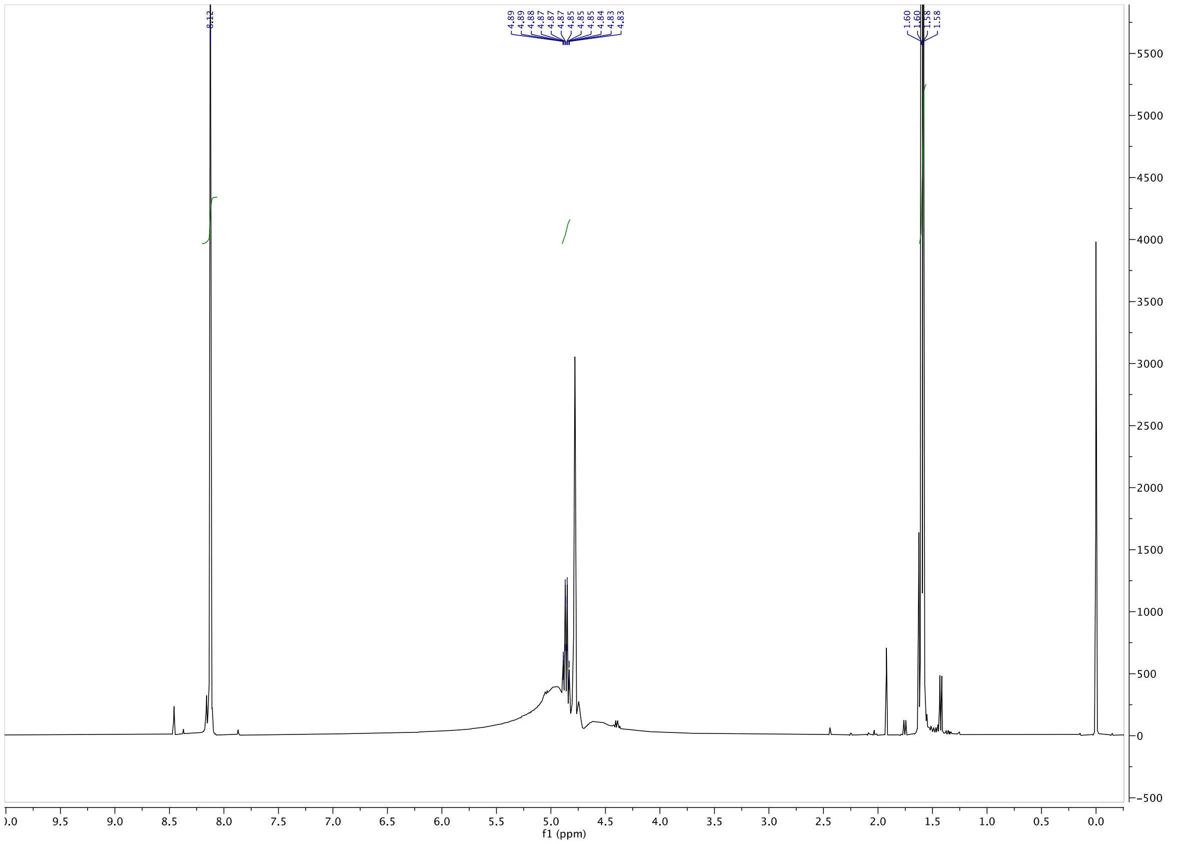

Fig S48. ^{1}H NMR spectrum of FoAla-CN in water.

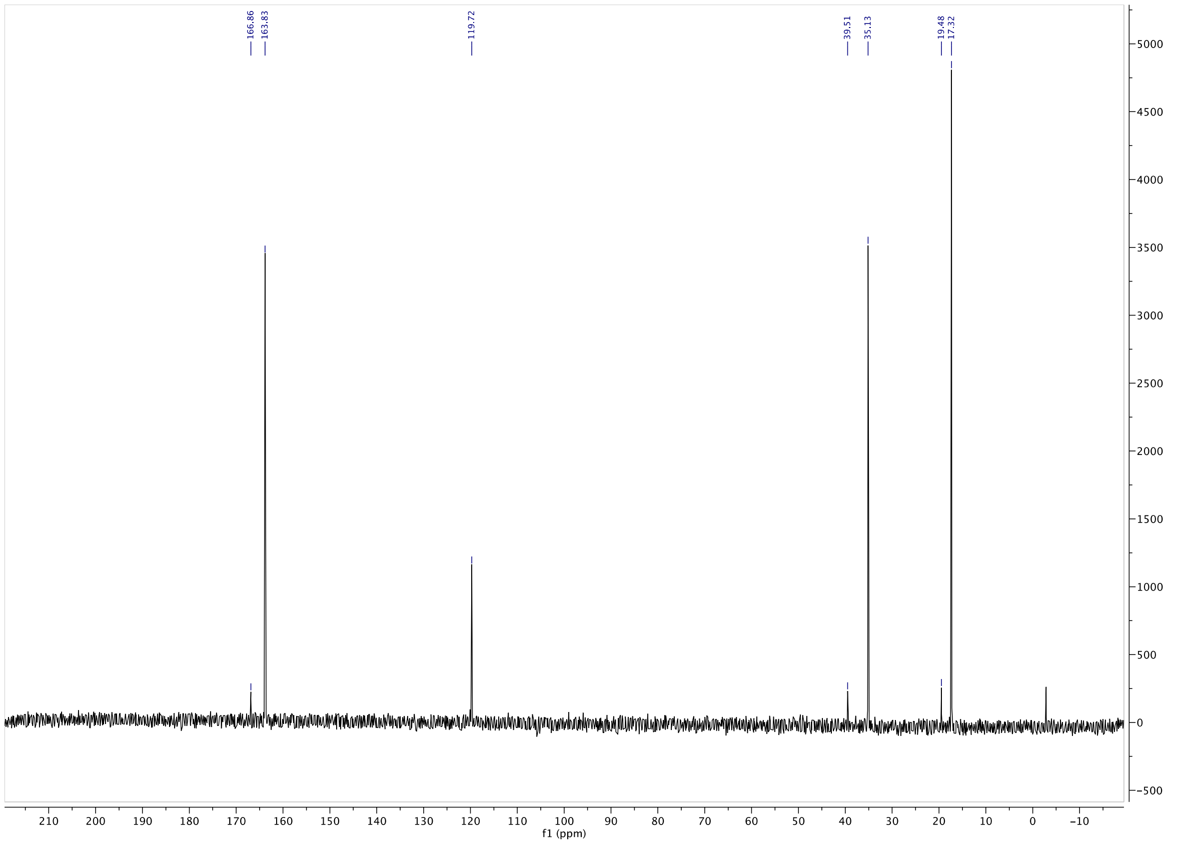

Fig S49. ^{13}C NMR spectrum of FoAla-CN in water.

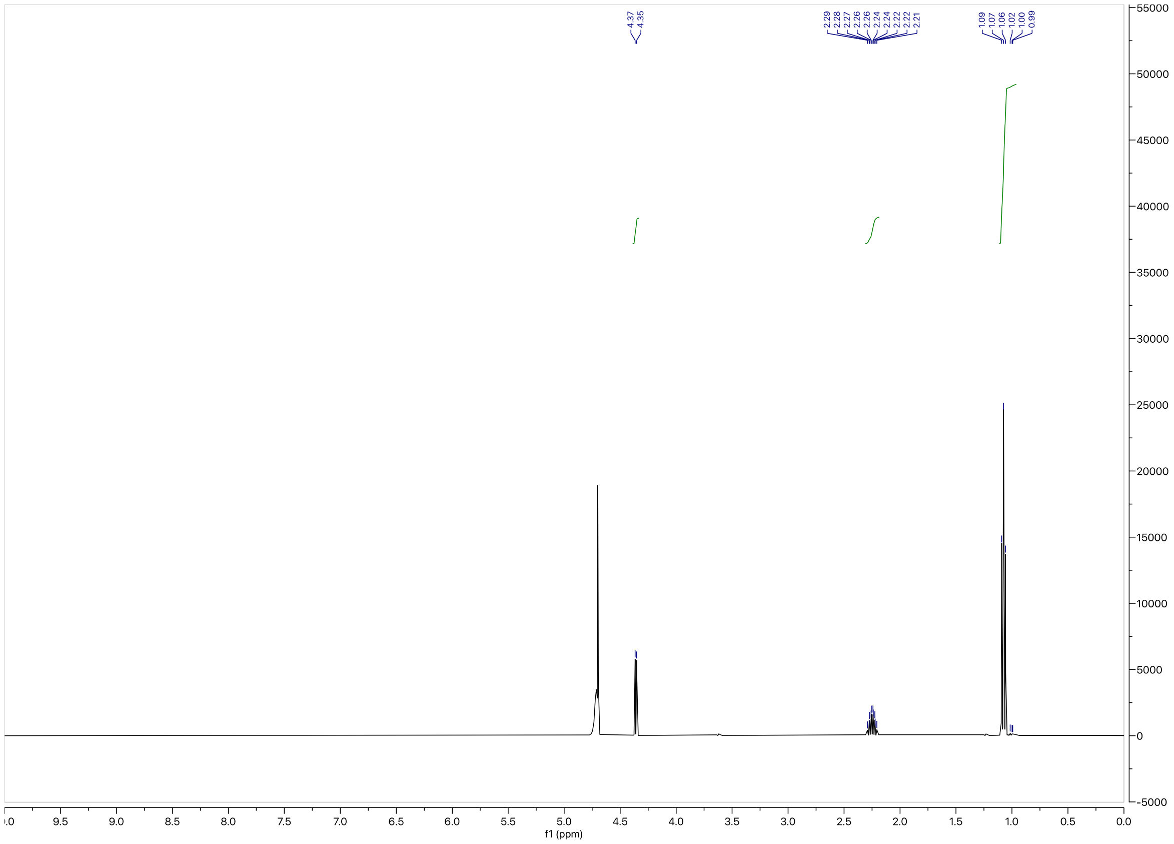

Fig S50. ^{1}H NMR spectrum of Val-CN.HCl in water.

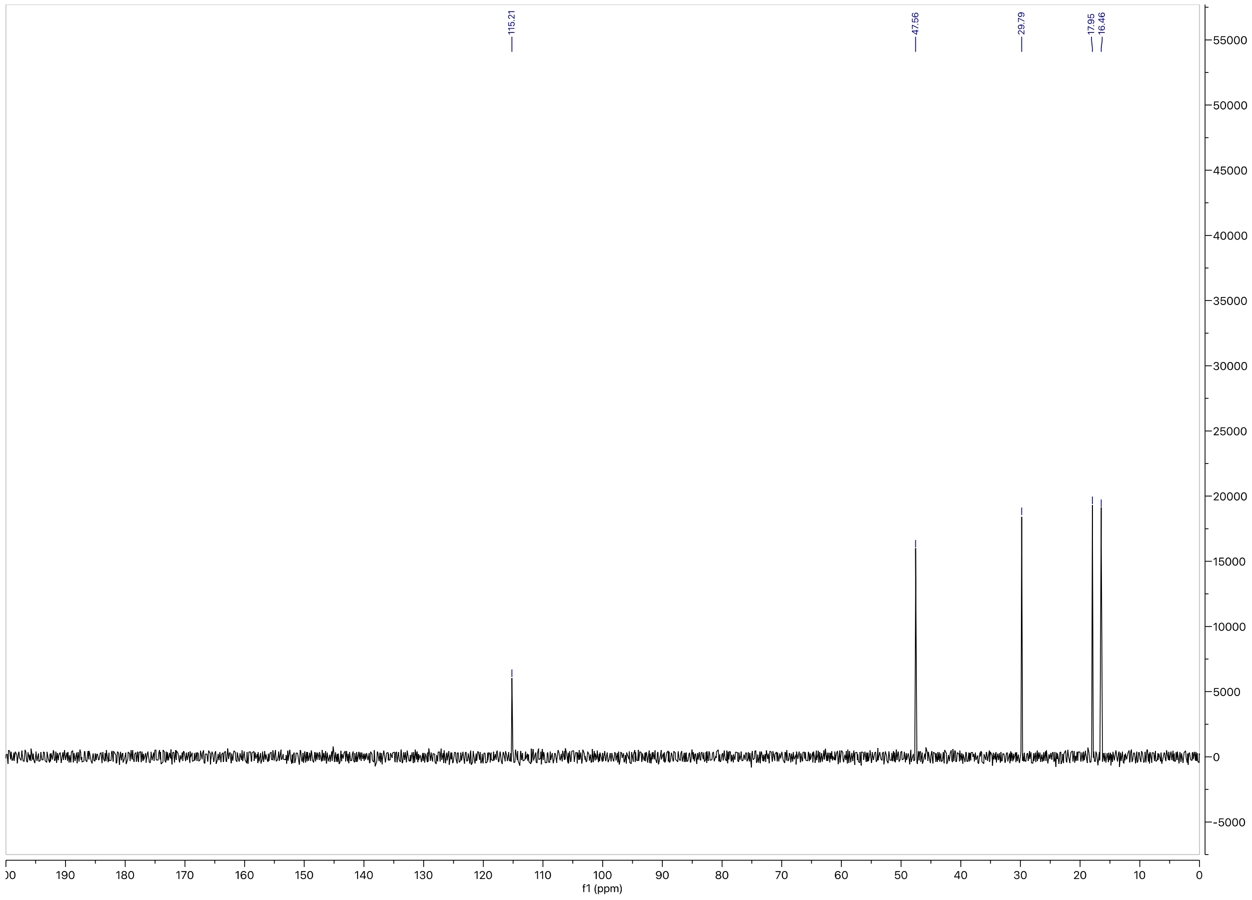

Fig S51. ^{13}C NMR spectrum of Val-CN.HCl in water.

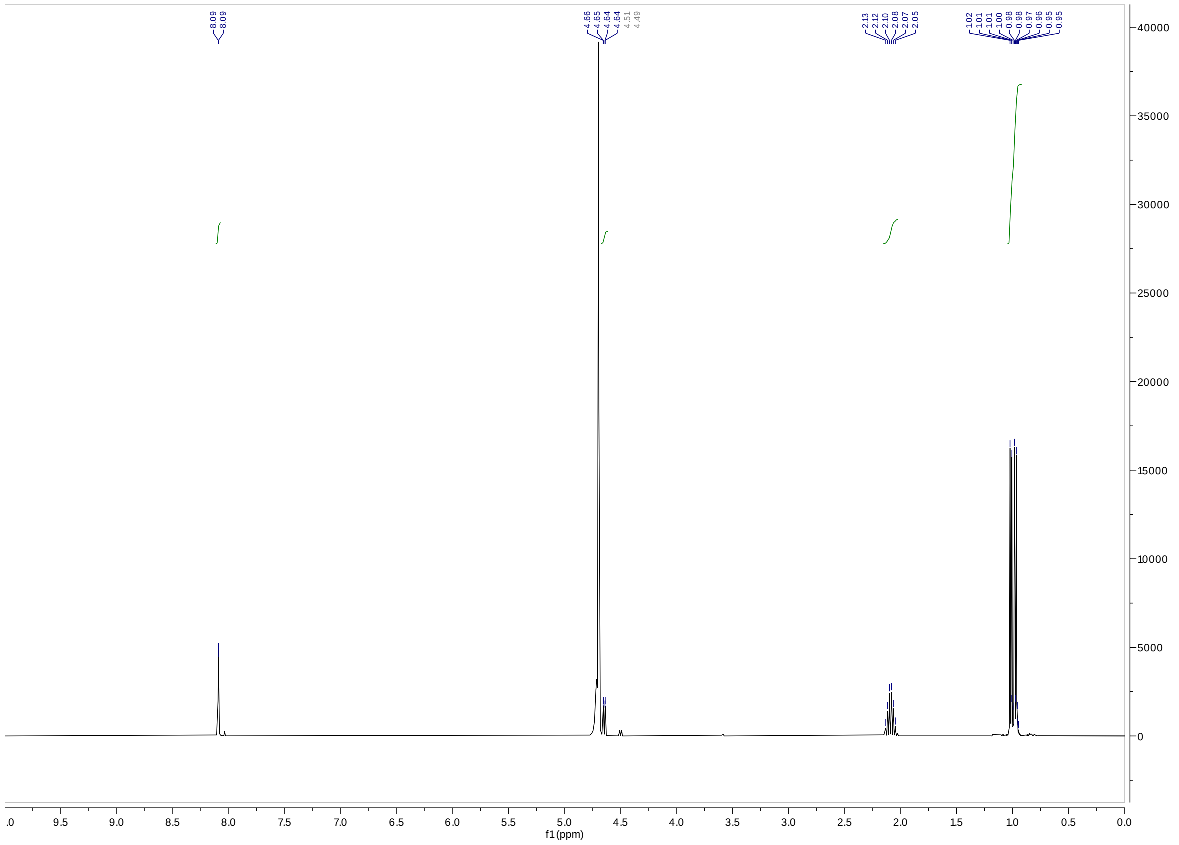

Fig S52. ^{1}H NMR spectrum of FoVal-CN in water.

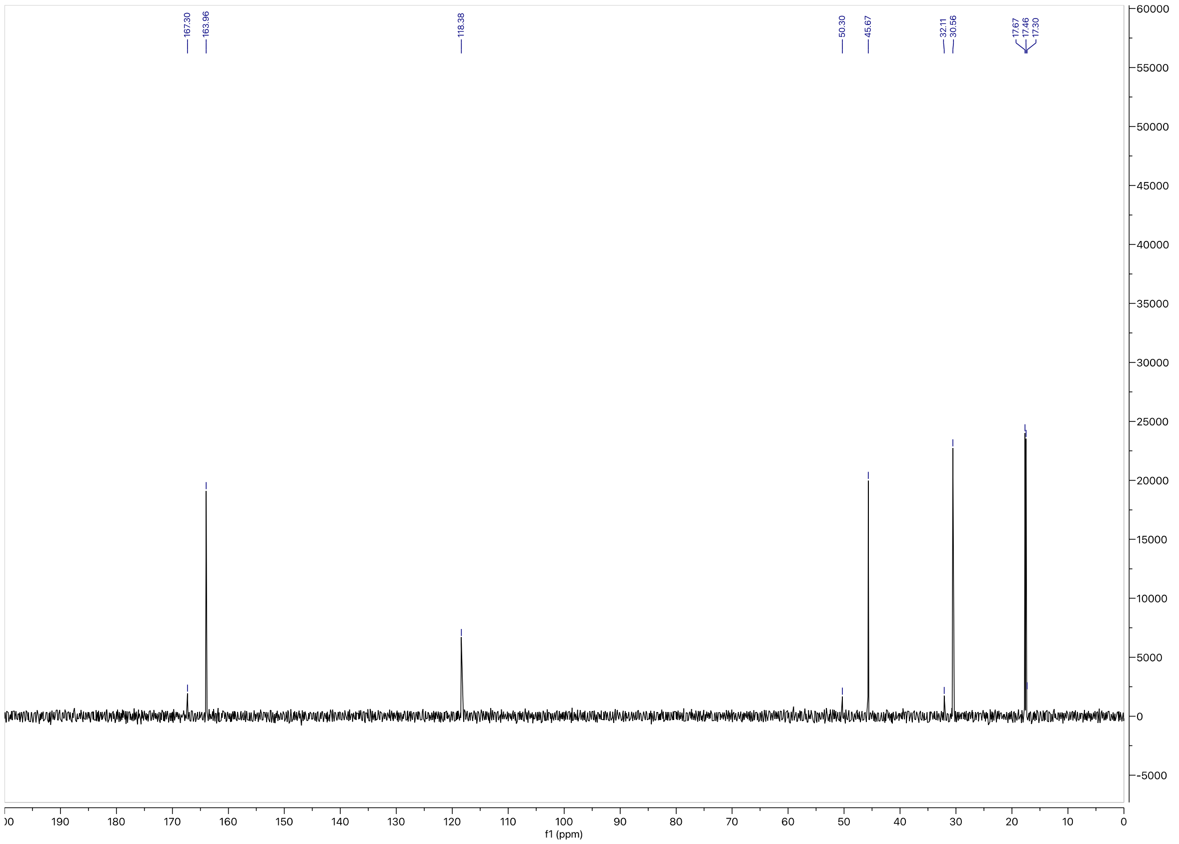

Fig S53. ^{13}C NMR spectrum of FoVal-CN in water.

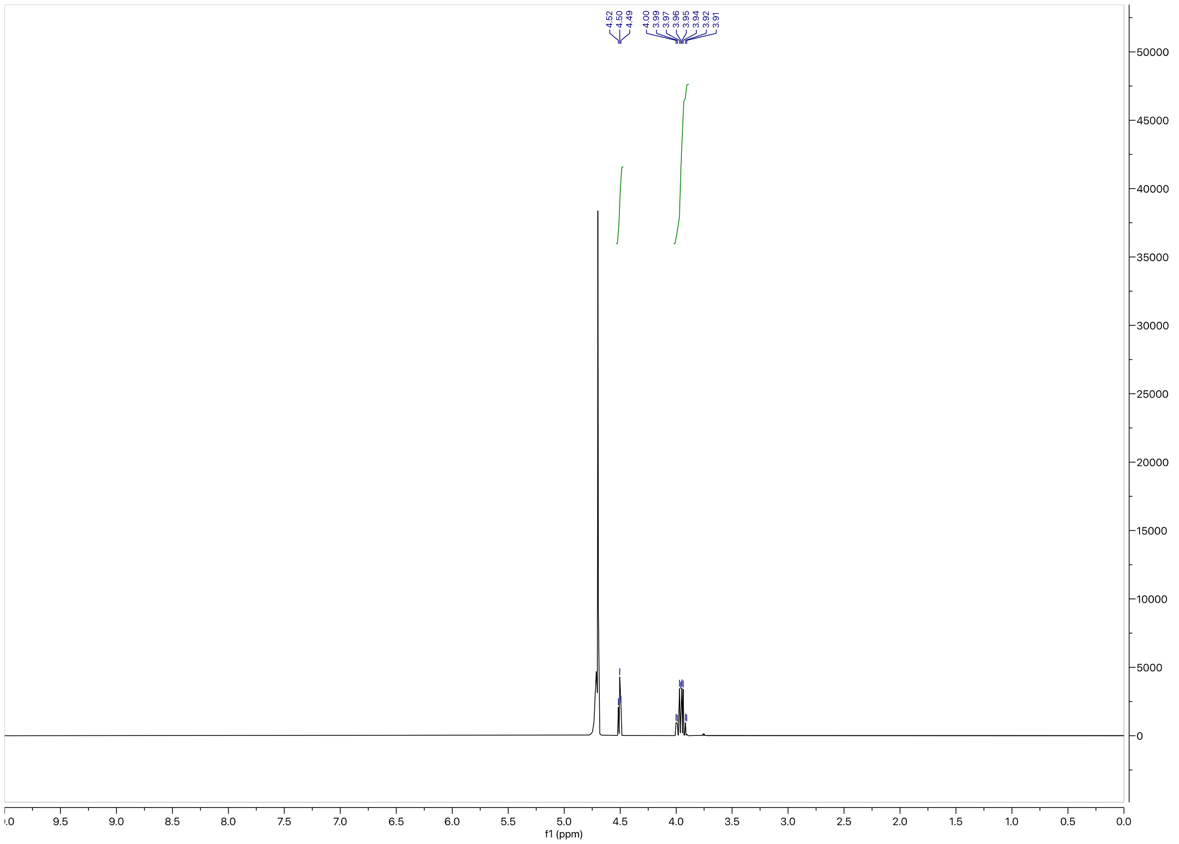

Fig S54. ^{1}H NMR spectrum of Ser-CN.HCl in water.

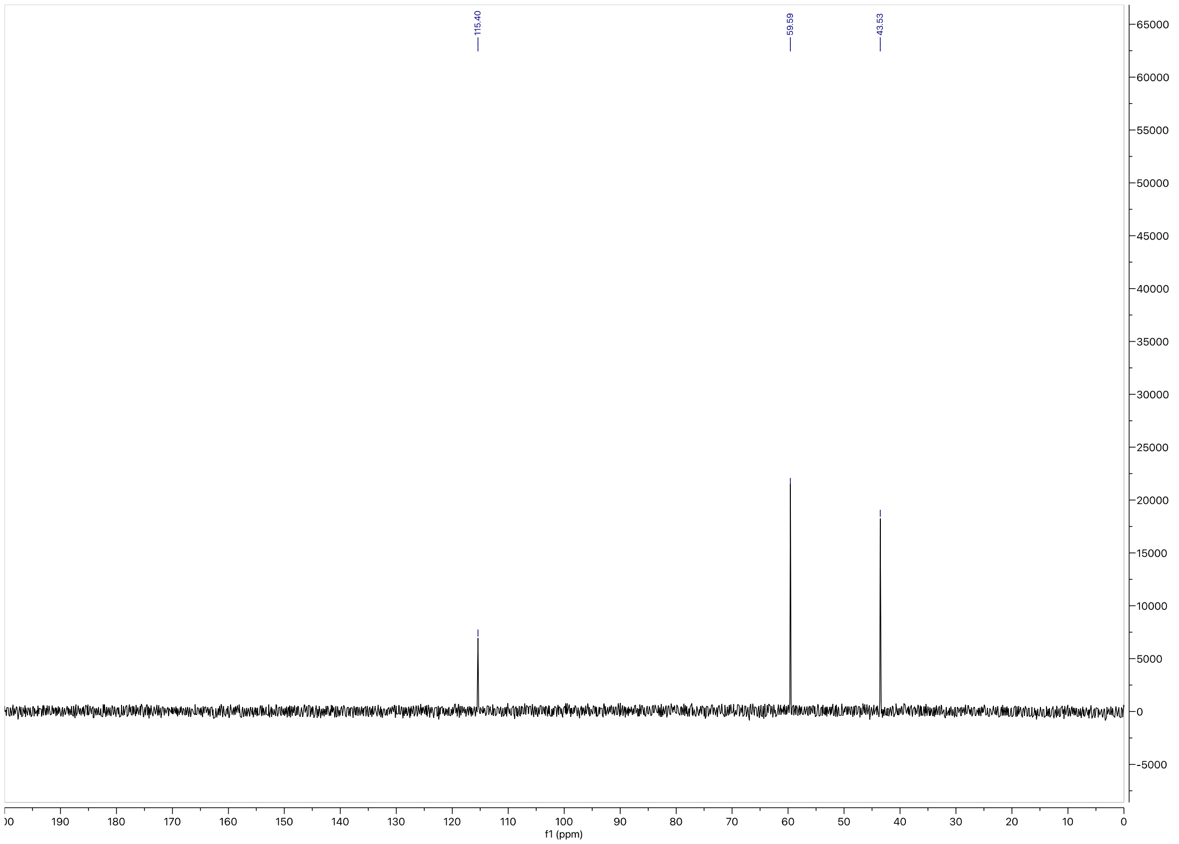

Fig S55. ^{13}C NMR spectrum of Ser-CN.HCl in water.

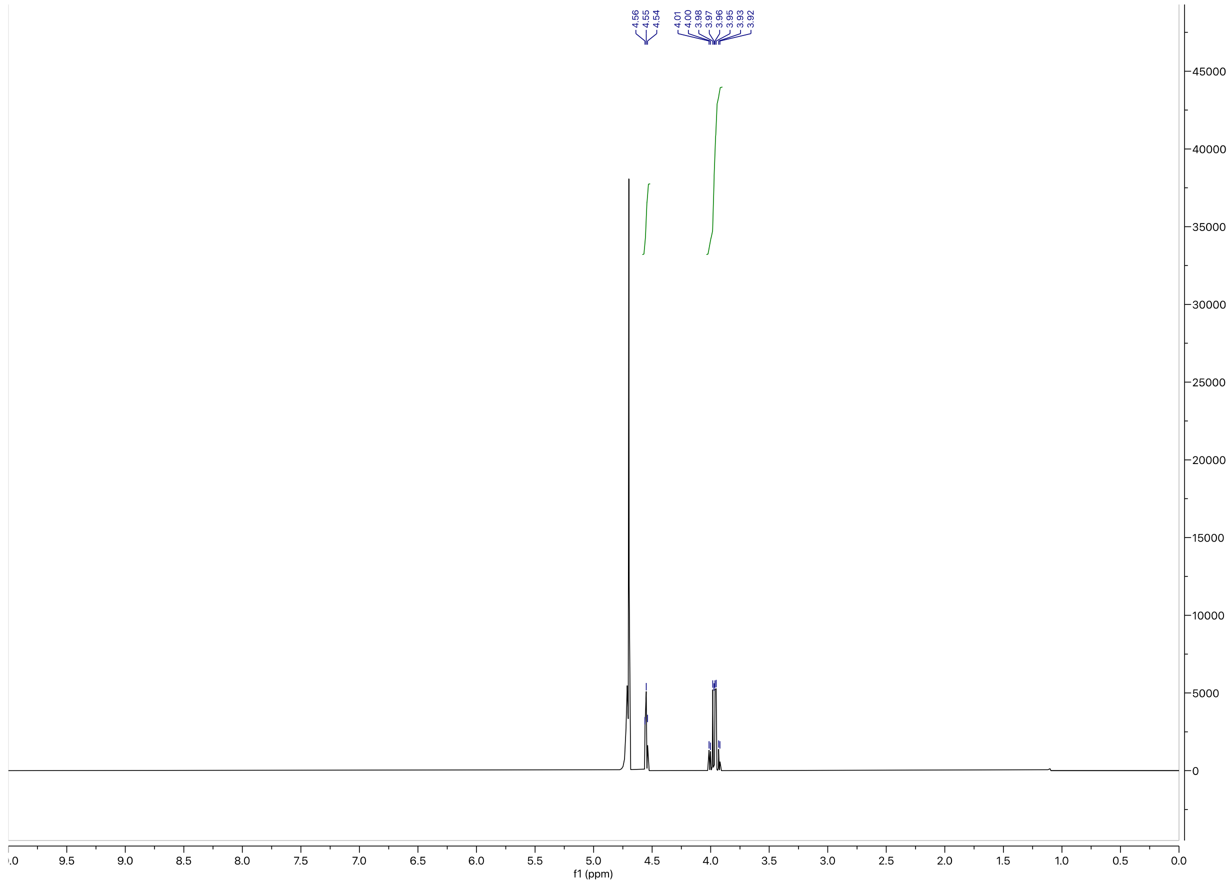

Fig S56. ^{1}H NMR spectrum of Ser-CN.HNO_3 in water.

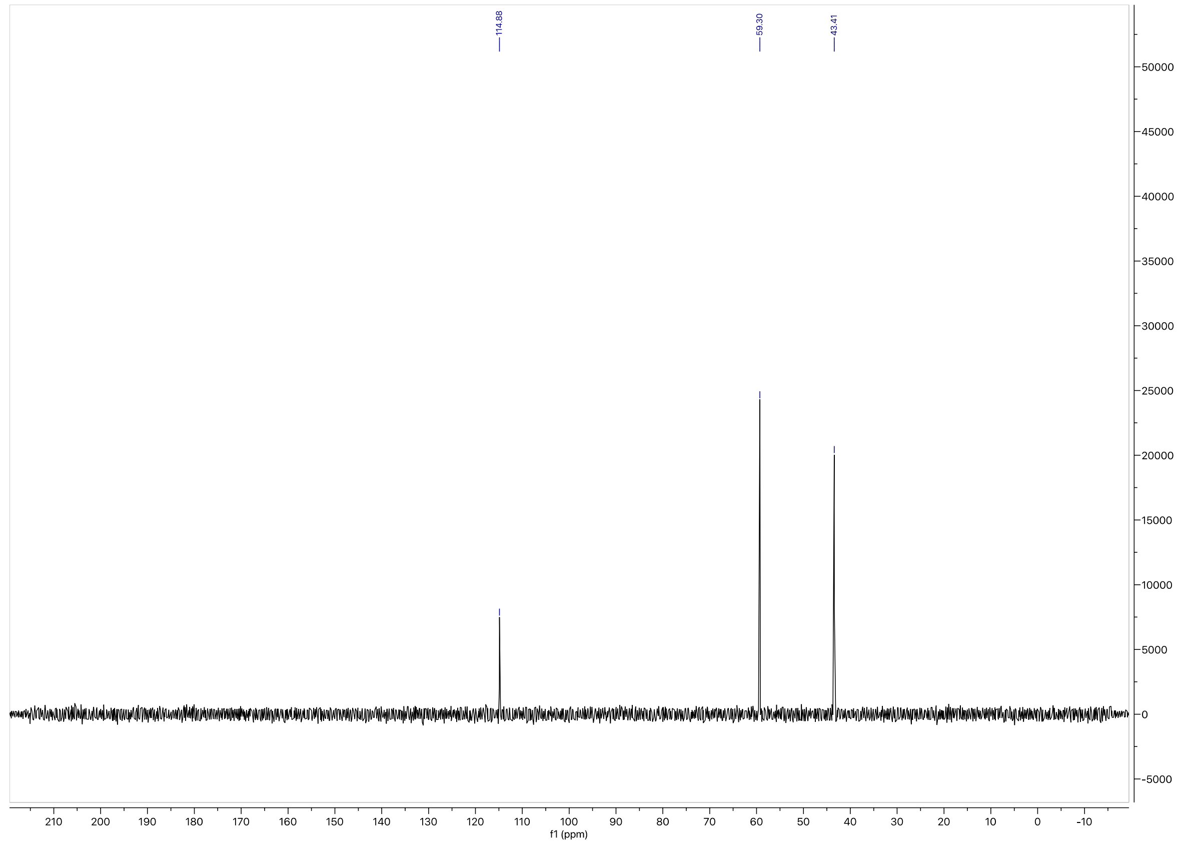

Fig S57. ^{13}C NMR spectrum of Ser-CN.HNO_3 in water.

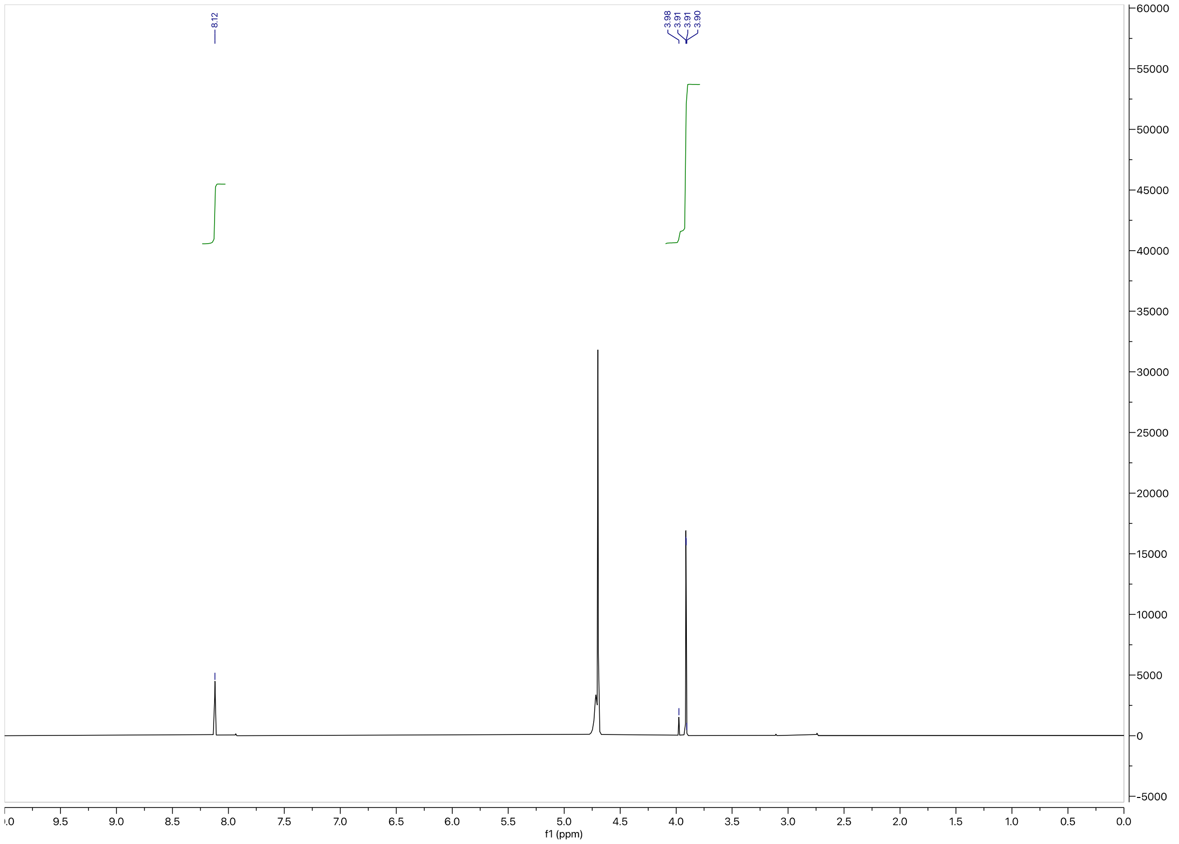

Fig S58. ^{1}H NMR spectrum of (N-formyl)glycinamide in water.

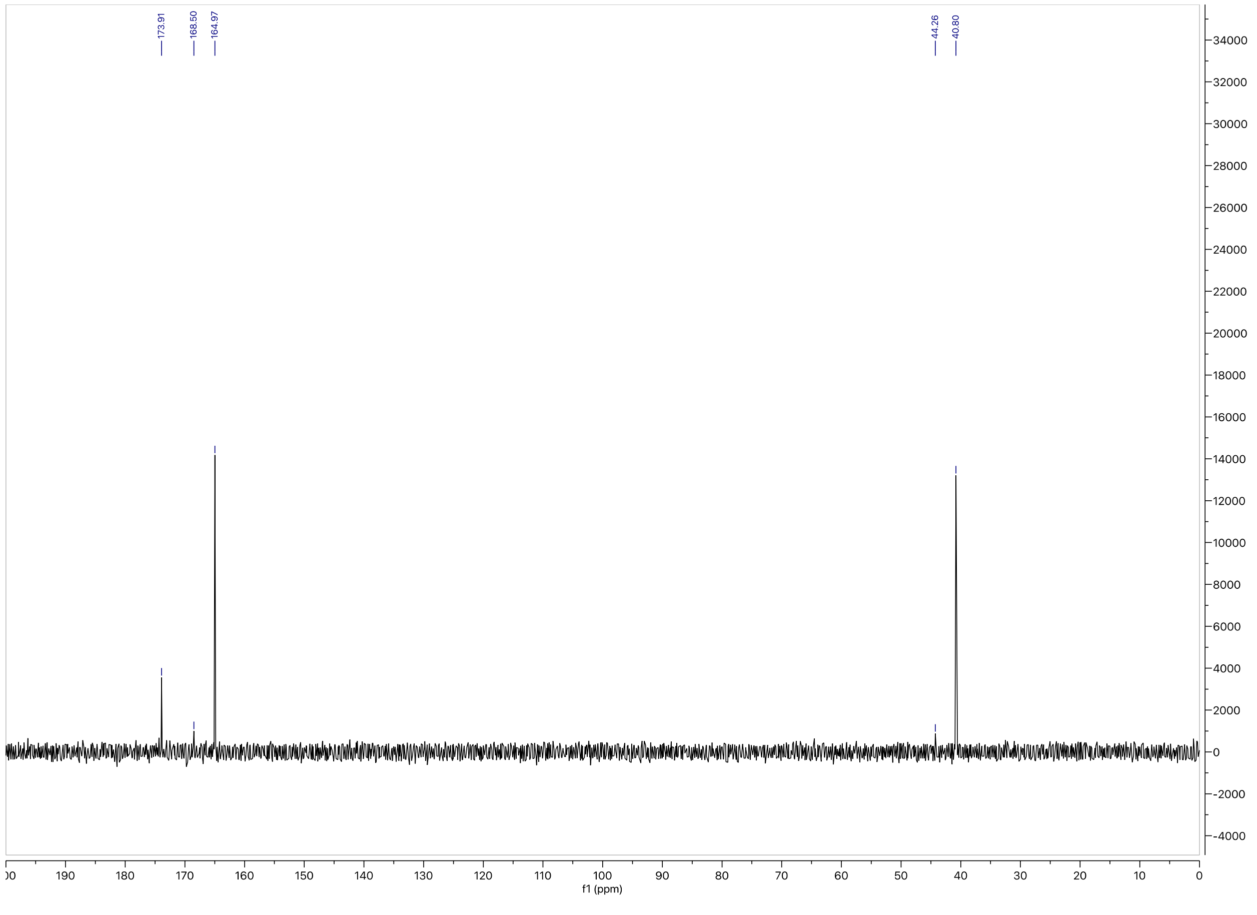

Fig S59. ^{13}C NMR spectrum of (N-formyl)glycinamide in water.

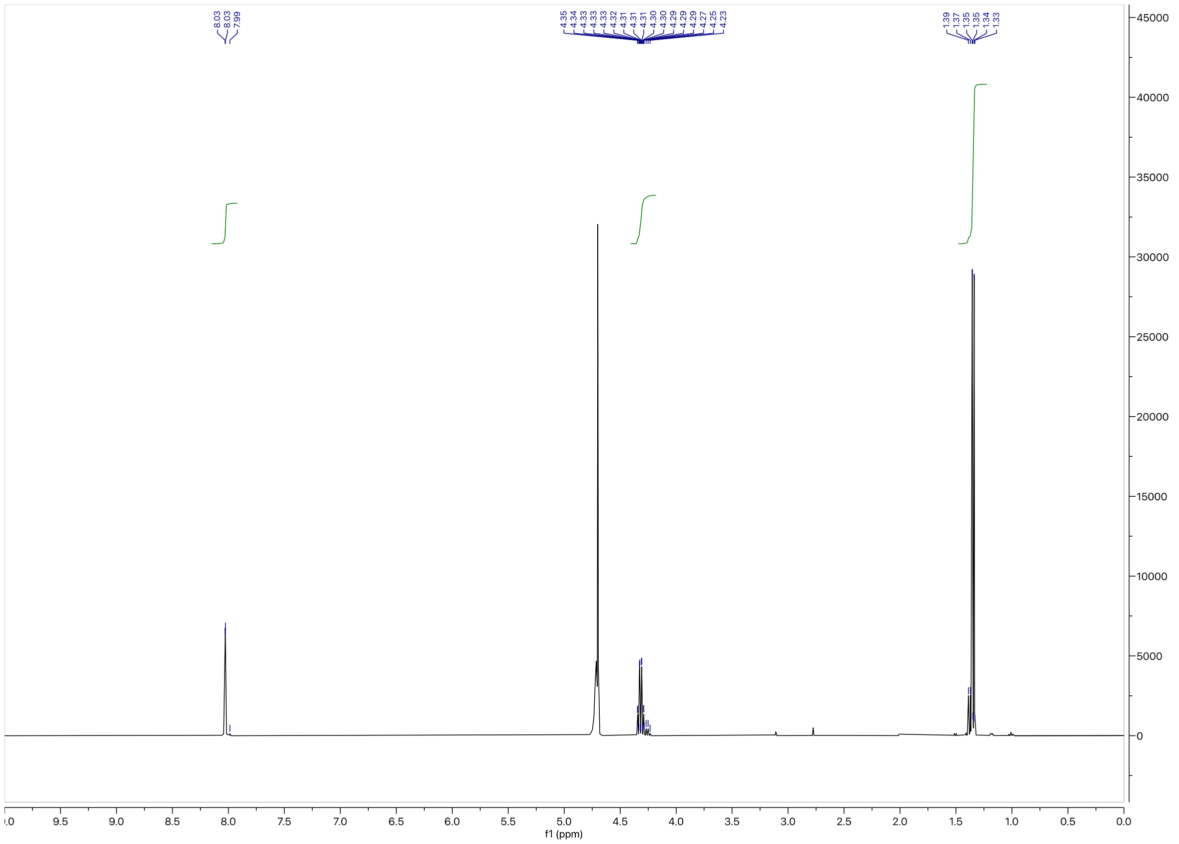

Fig S60. ^{1}H NMR spectrum of (N-formyl)alaninamide in water.

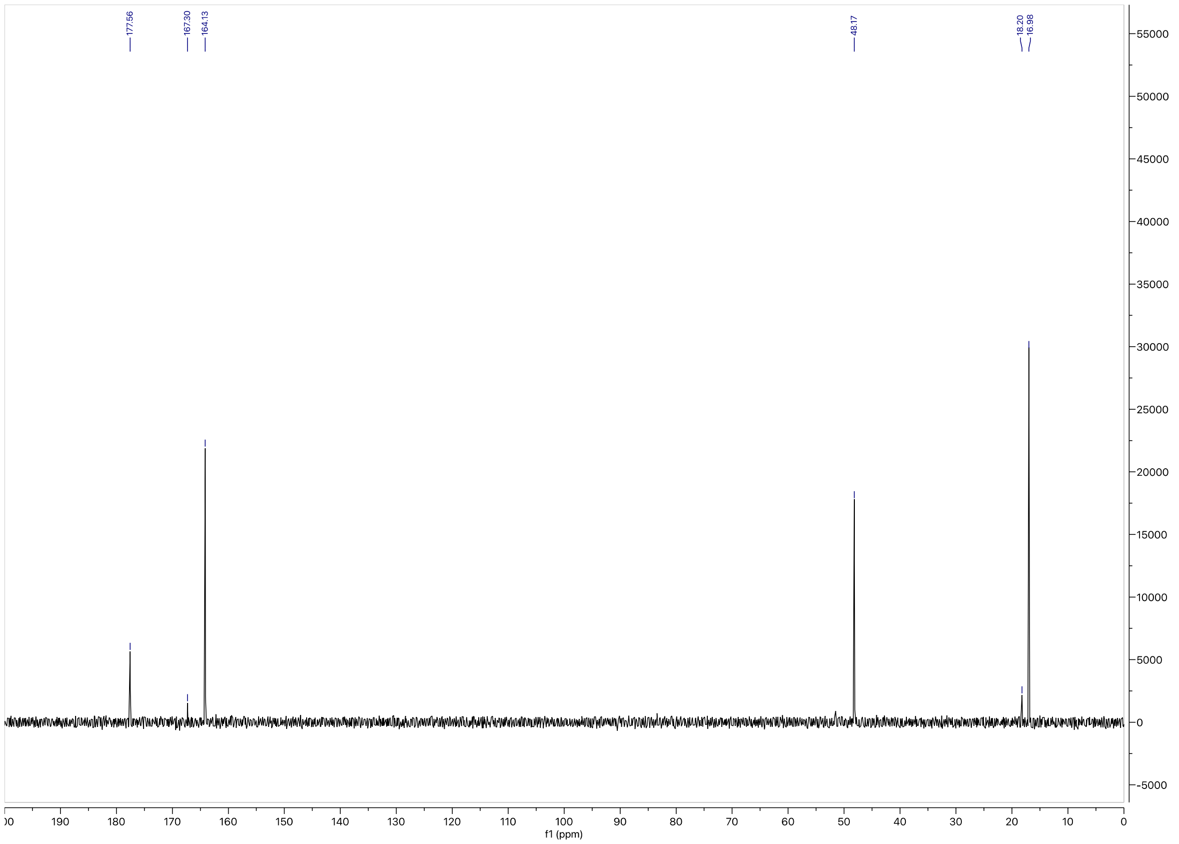

Fig S61. ^{13}C NMR spectrum of (N-formyl)alaninamide in water.

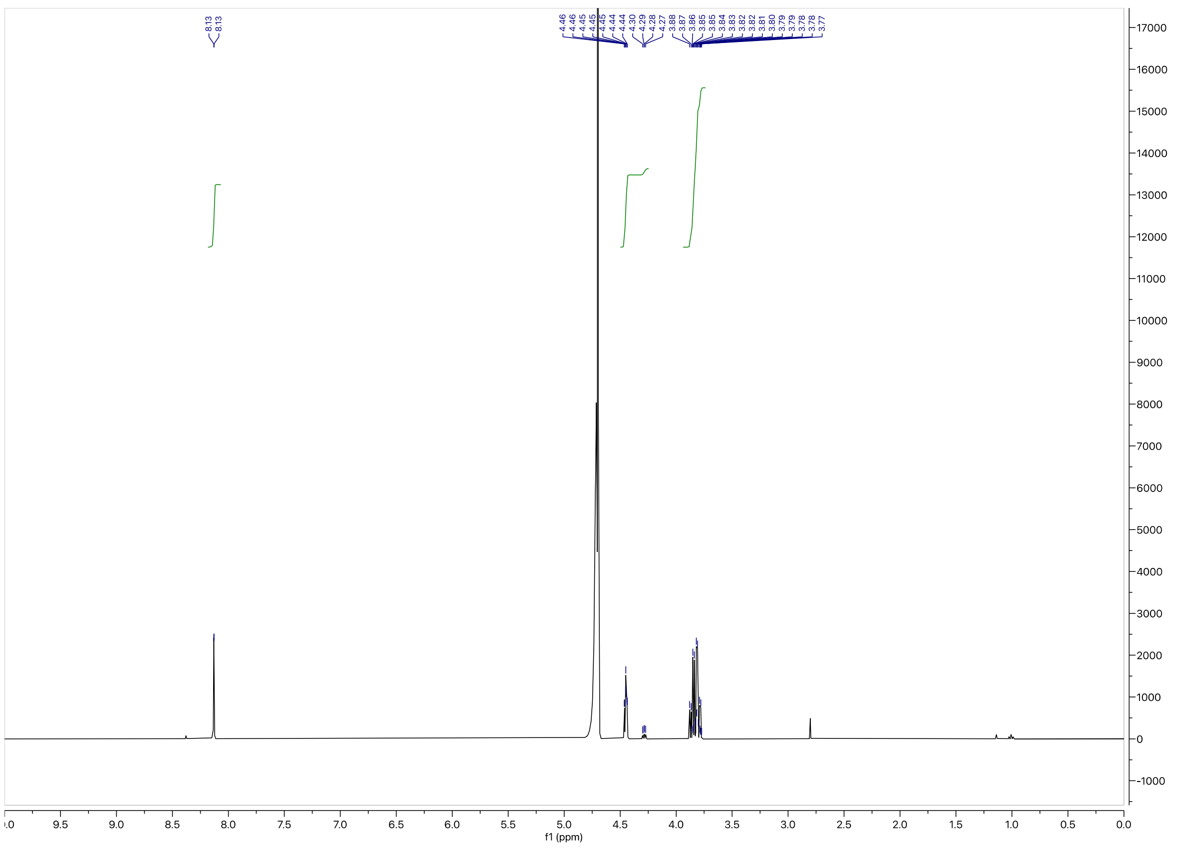

Fig S62. ^{1}H NMR spectrum of (N-formyl)serinamide in water.

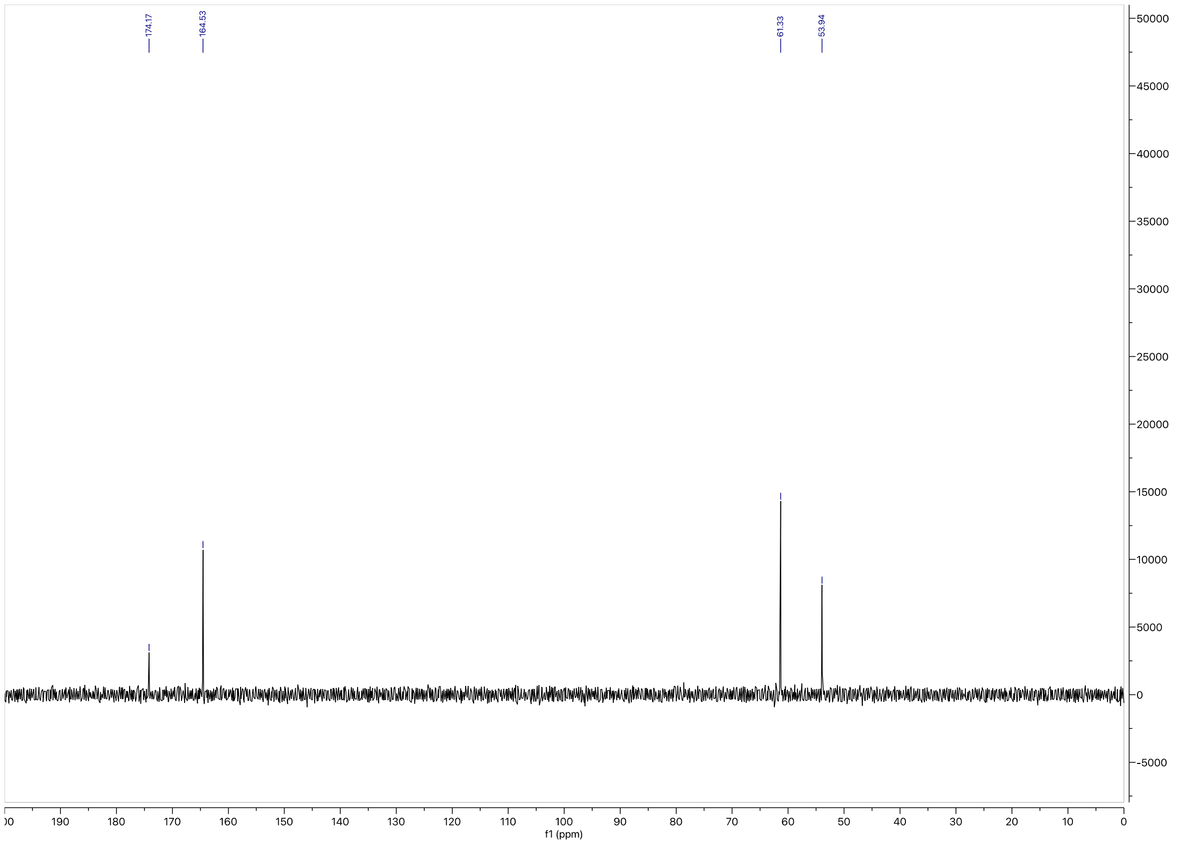

Fig S63. ^{13}C NMR spectrum of (N-formyl)serinamide in water.

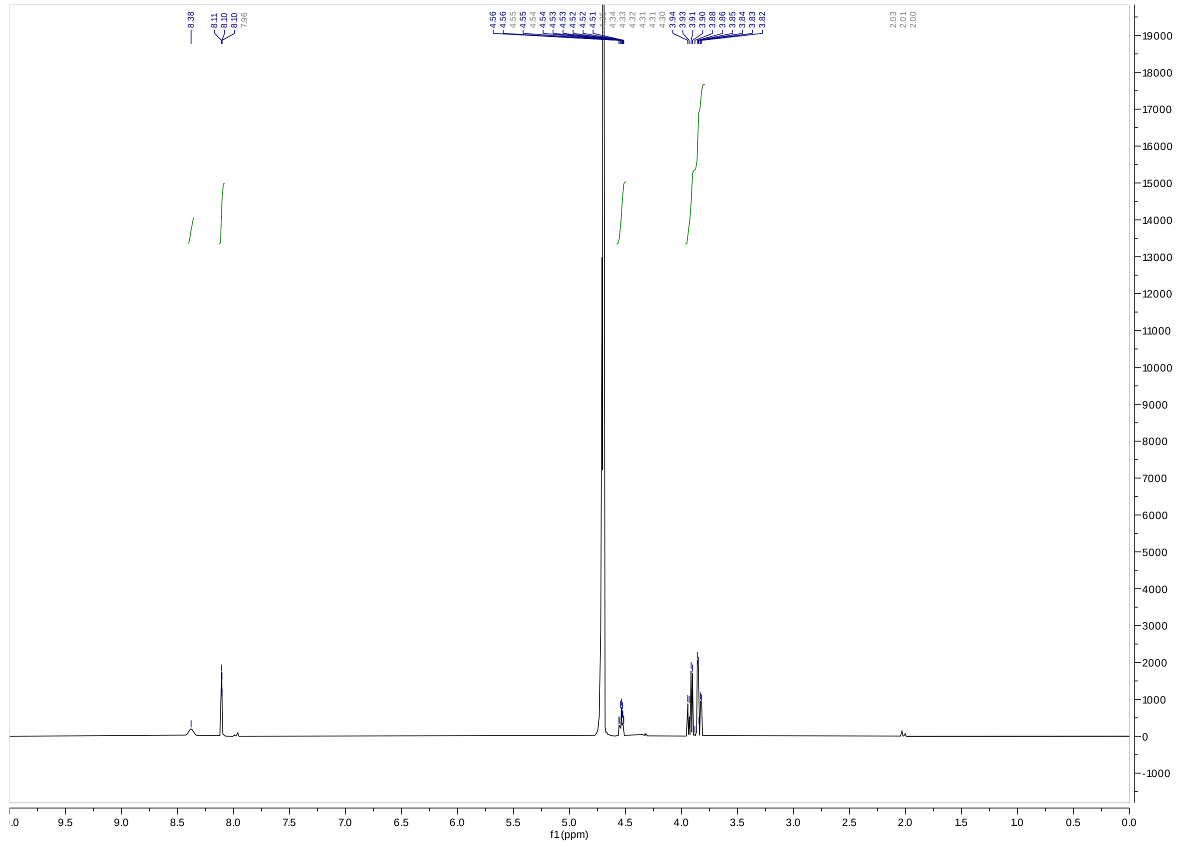

Fig S64. ^{1}H NMR of (N-formyl)serine in water.

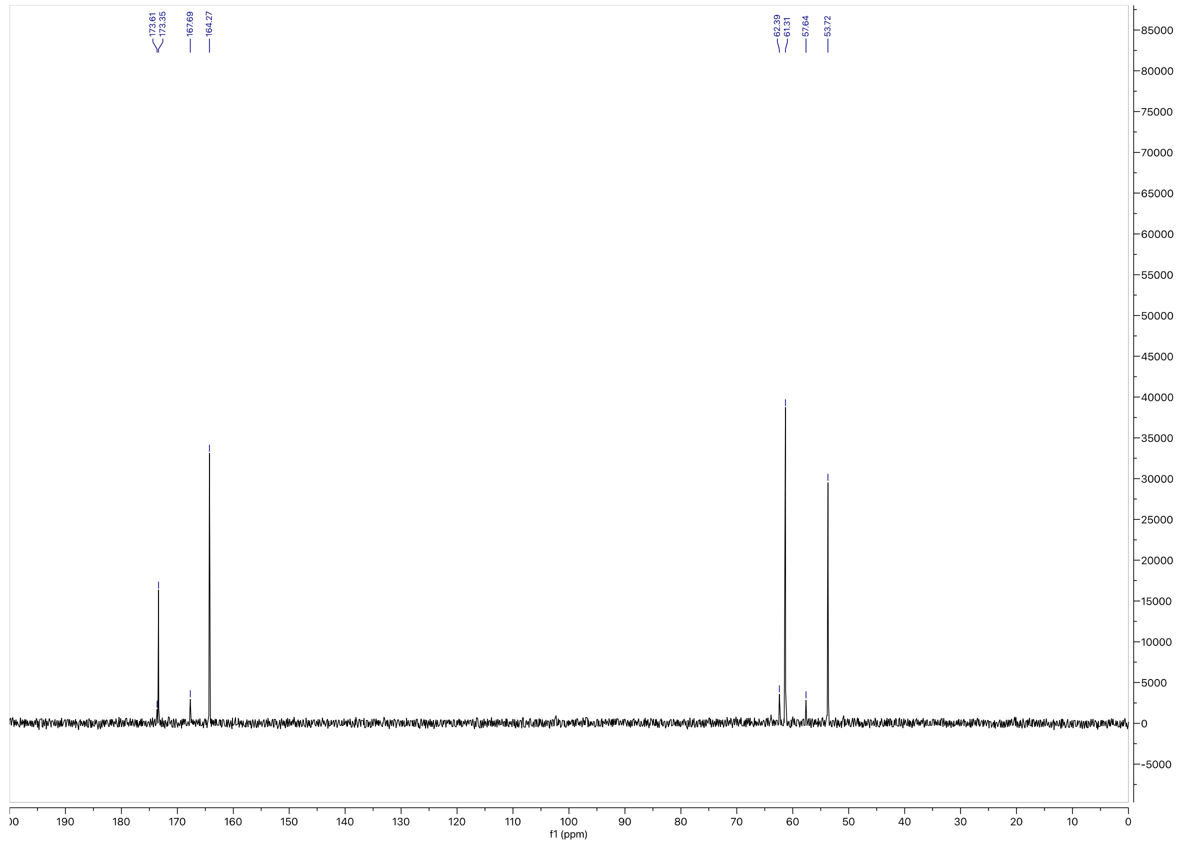

Fig S65. ^{13}C NMR of (N-formyl)serine in water.

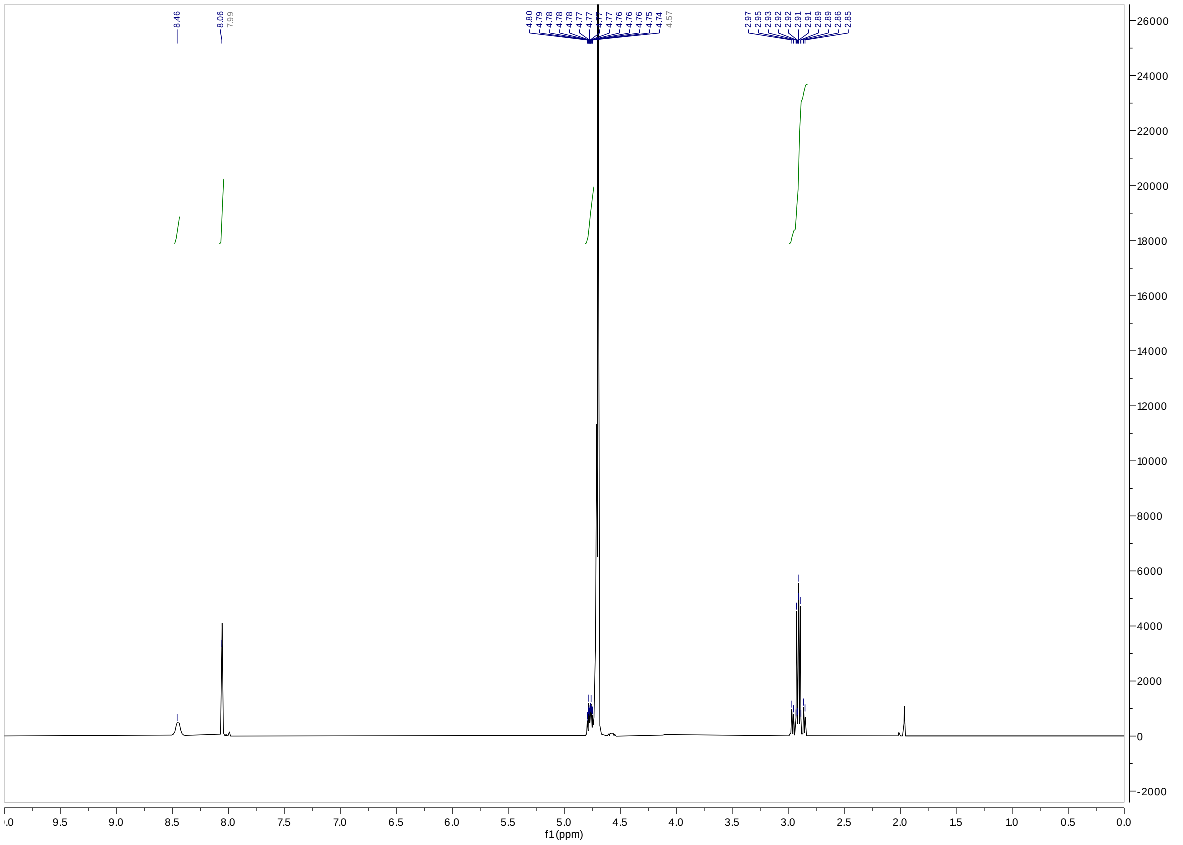

Fig S66. ^{1}H NMR spectrum of (N-formyl)aspartic acid in water.

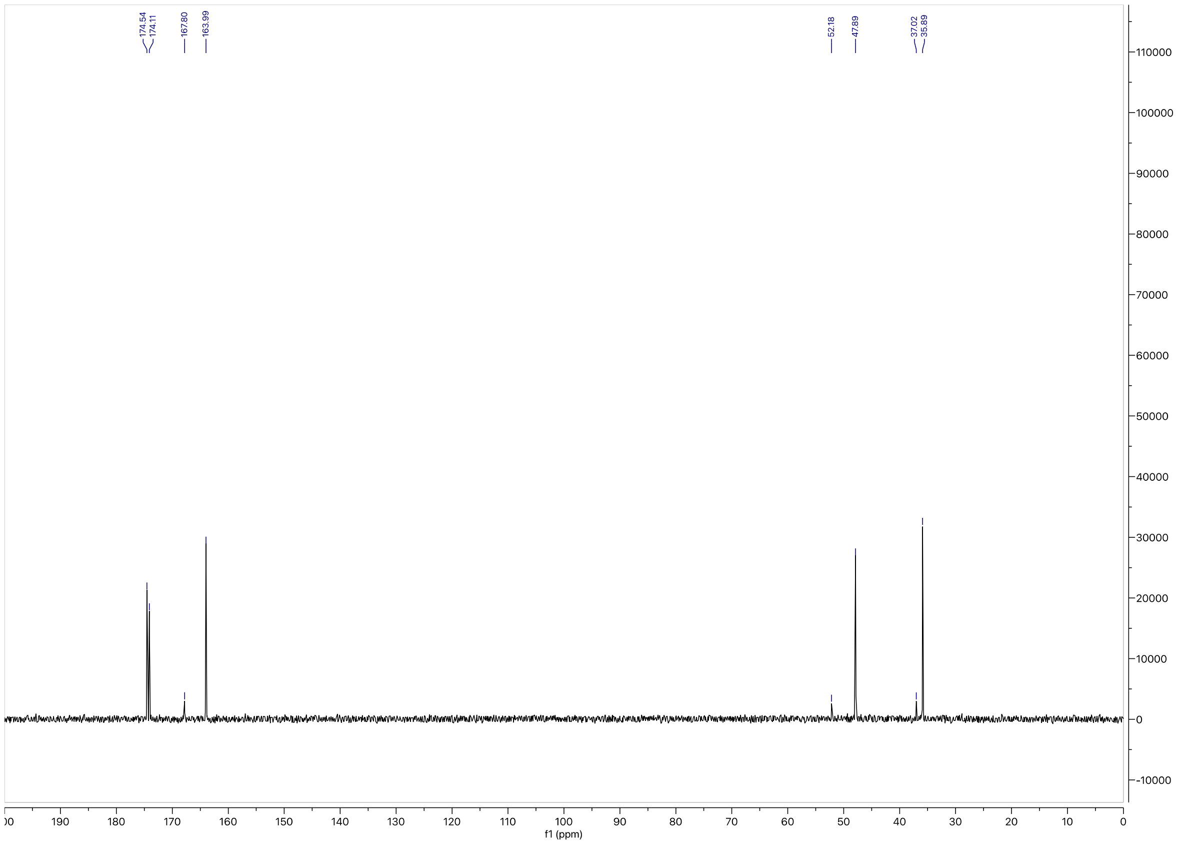

Fig S67. ^{13}C NMR spectrum of (N-formyl)aspartic acid in water.

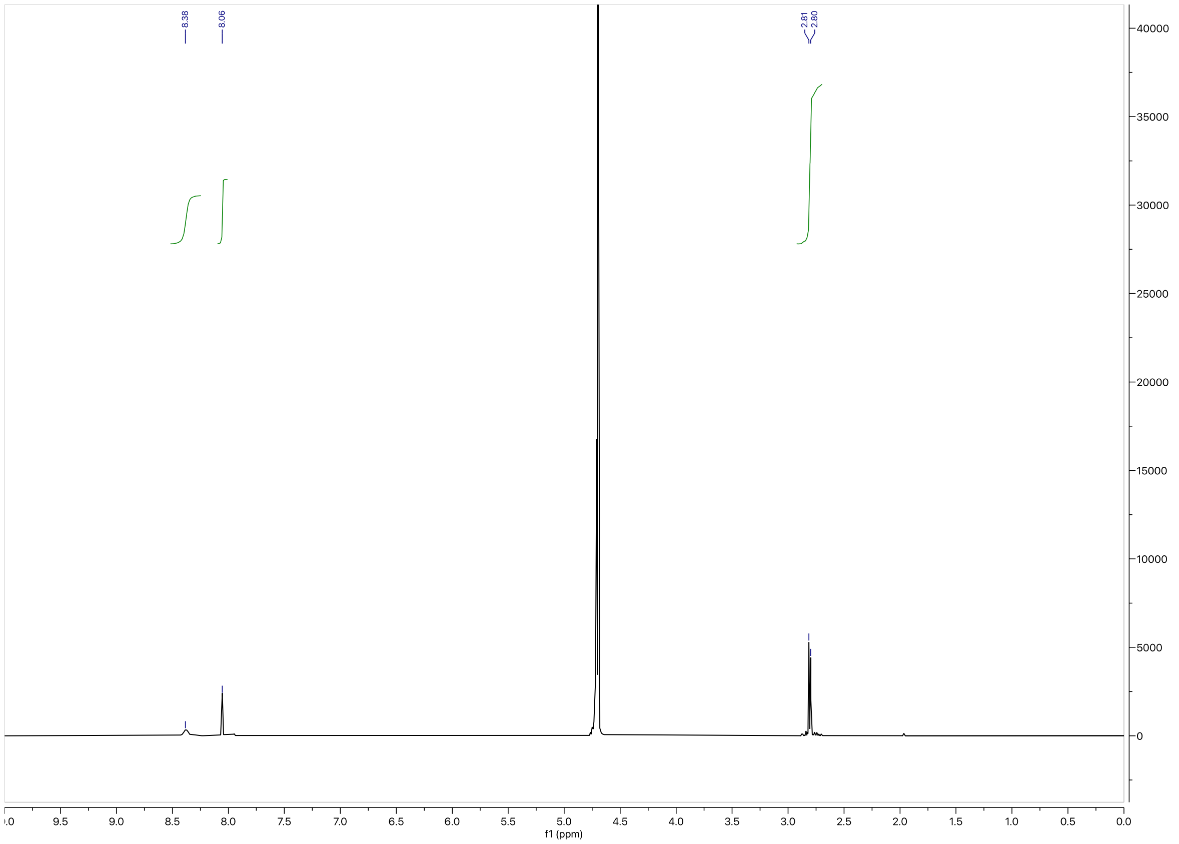

Fig S68. ^{1}H NMR spectrum of (N-formyl)asparagine in water.

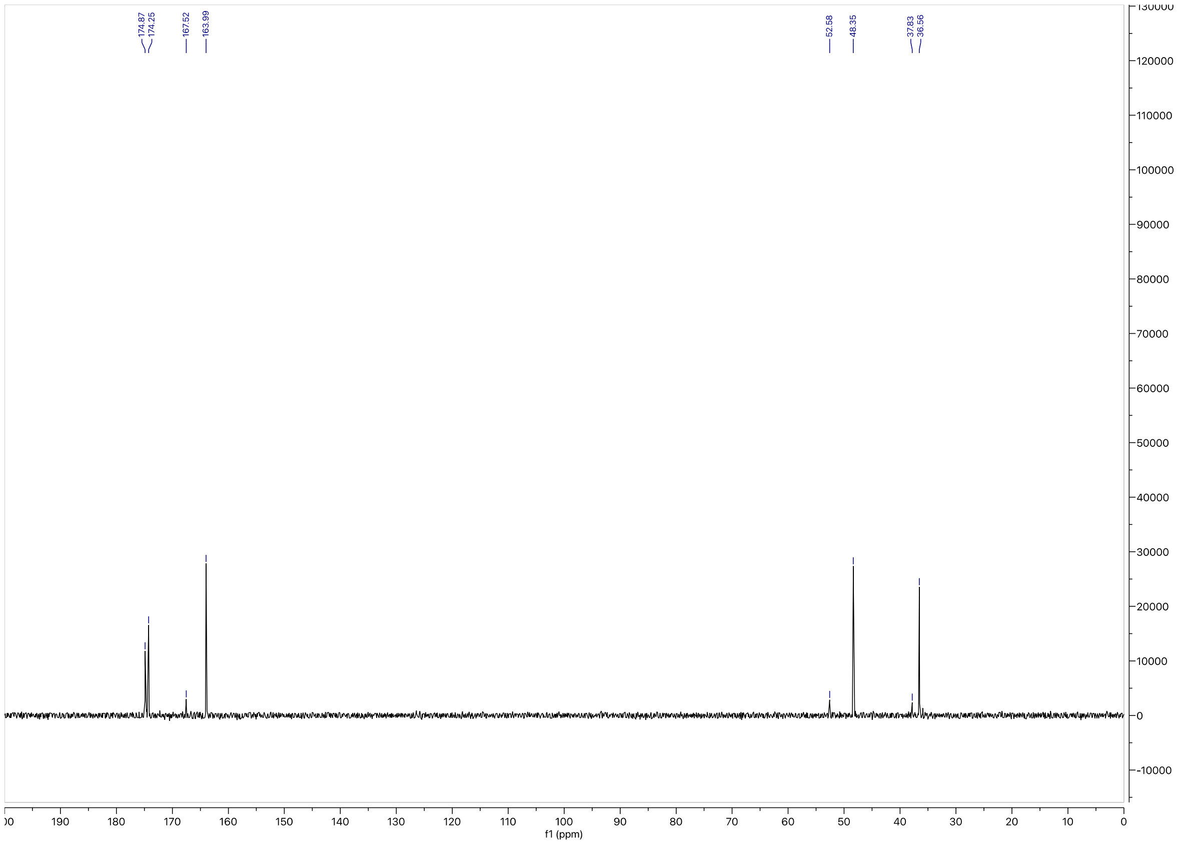

Fig S69. ^{13}C NMR spectrum of (N-formyl)asparagine in water.

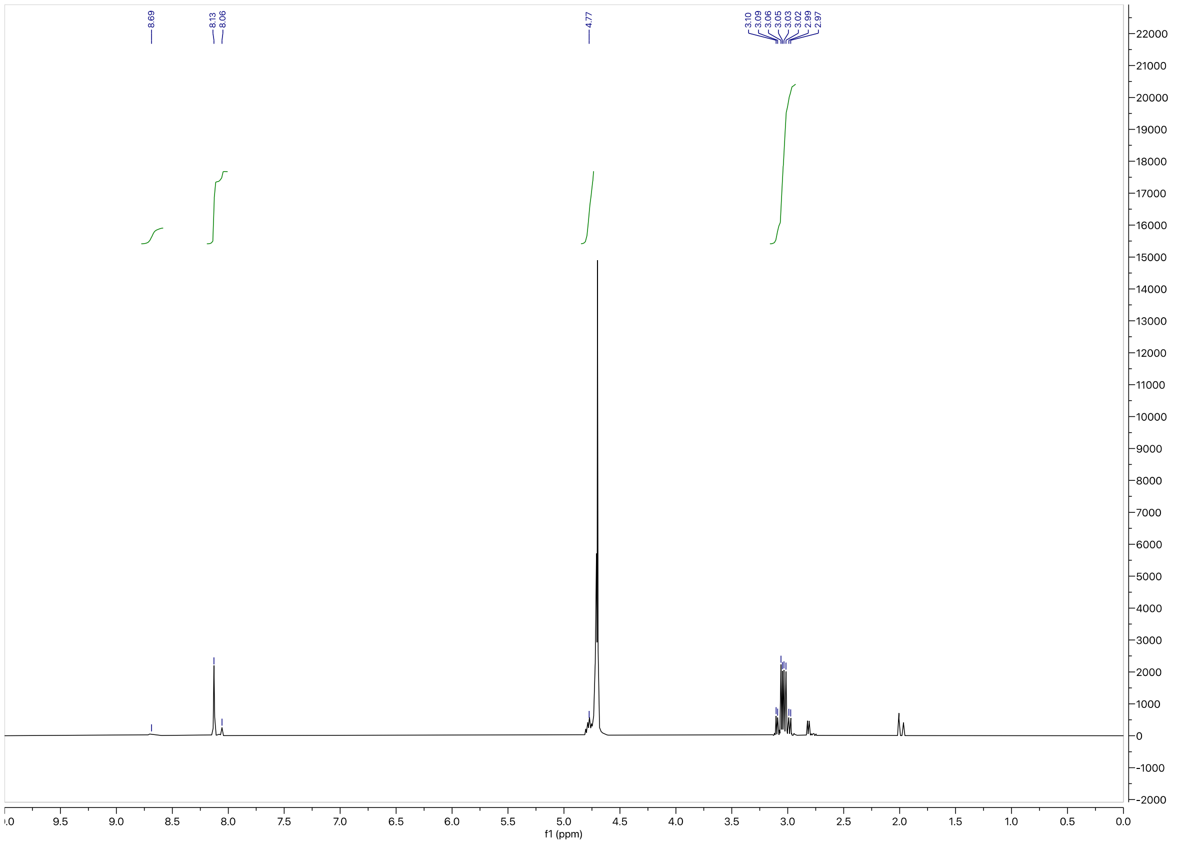

Fig S70. ^{1}H NMR spectrum of Fo(β-CN)Ala in water.

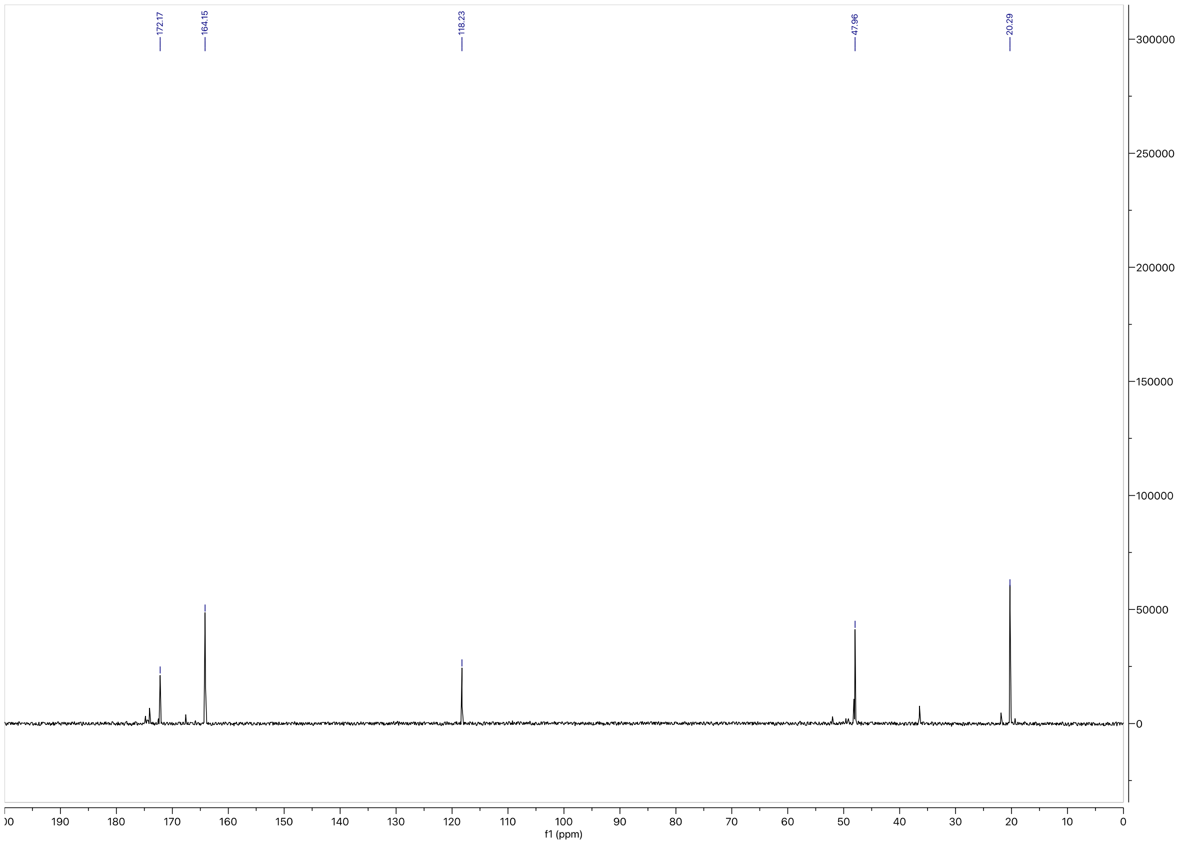

Fig S71. ^{13}C NMR spectrum of Fo(β-CN)Ala in water.

Nucleophile Additions to FoDHA-CN

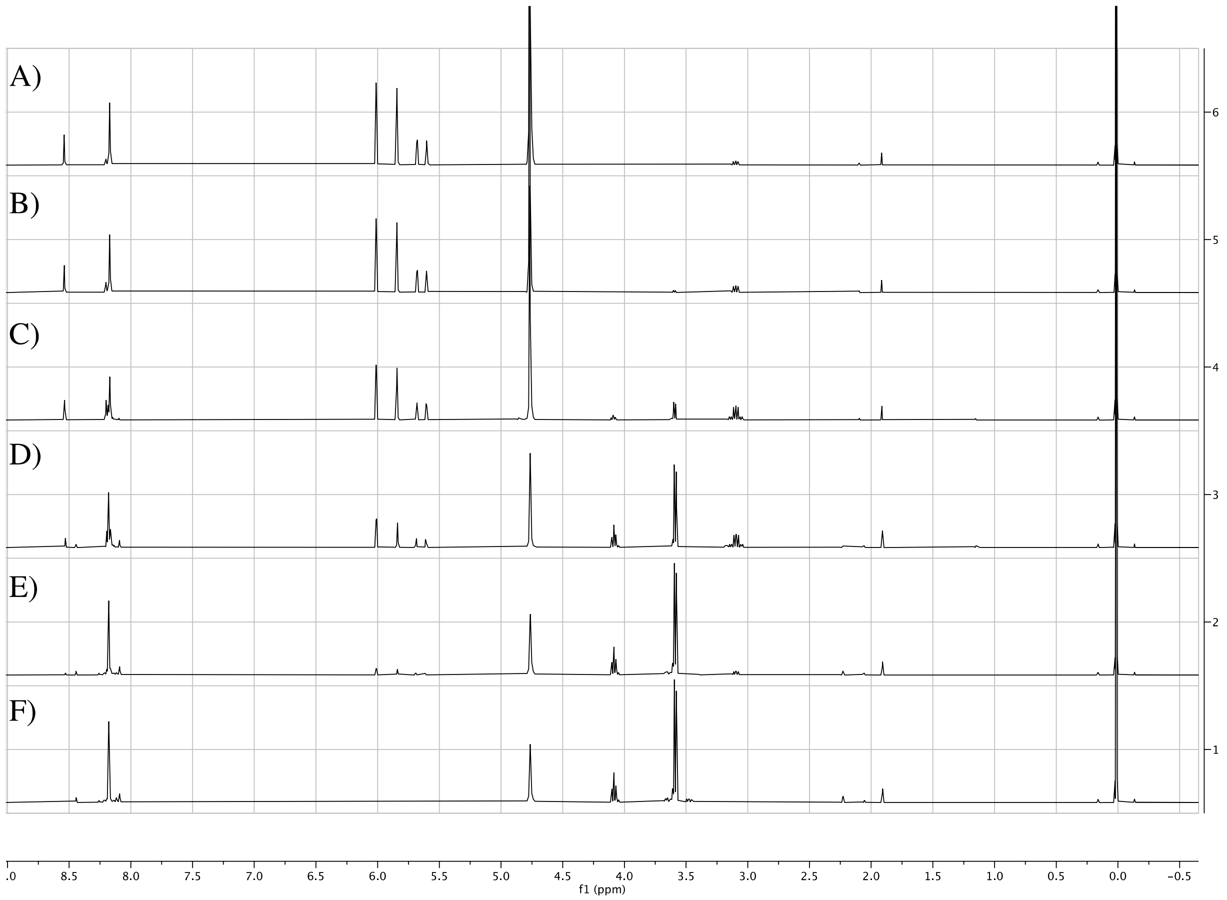

Fig S72. Stack of ^{1}H NMR spectra showing the progress of the reaction between FoDHA-CN and NH_4Cl (5 eq.) at room temperature and pH 9.2. A) 20 minutes, showing FoDHA-CN and trace ammonia adduct Fo(β-NH_2)Ala; B) 1h; C) 4h. The ammonia adduct and rearranged adduct (β-FoNH)Ala-CN are visible; D) 16h; E) 48h; F) 96h, showing complete conversion to the rearranged product.

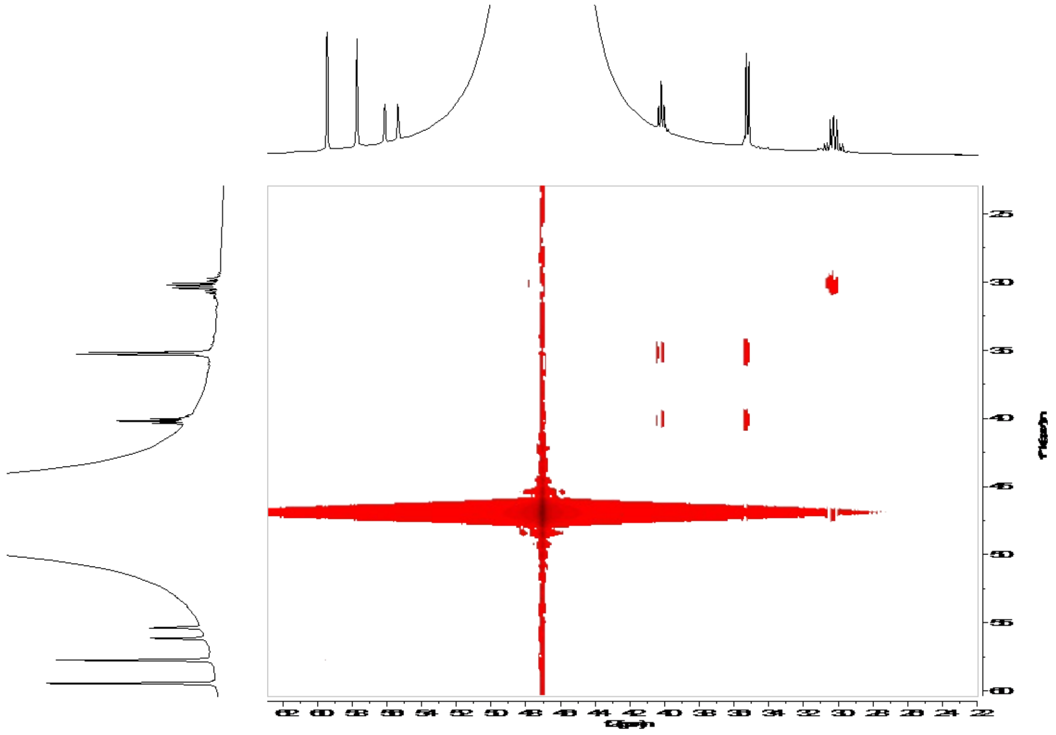

Fig S73. COSY spectrum of the reaction between NH_4Cl and FoDHA-CN, pH 9.2 after 10 hours, showing intermediate and product.

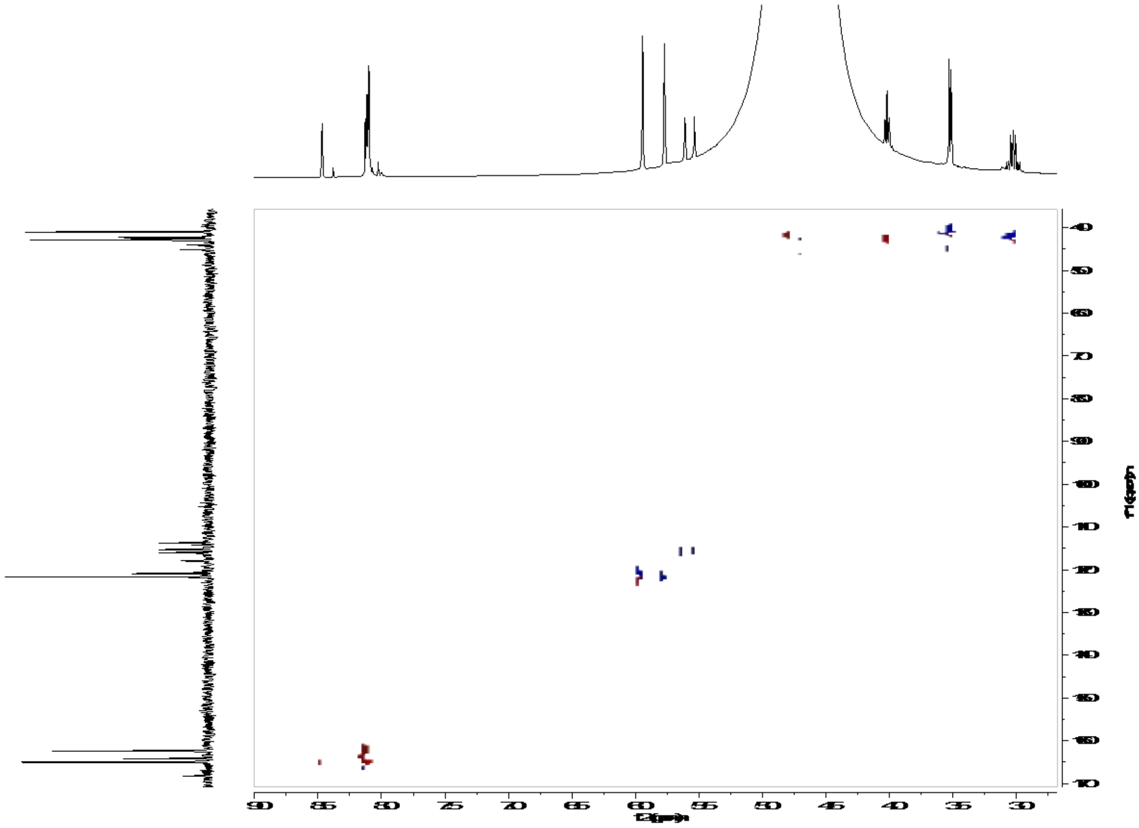

Fig S74. HSQC spectrum for partially reacted FoDHA-CN with ammonia at pH 9.2. Signals for the intermediate and product facilitate the assignments.

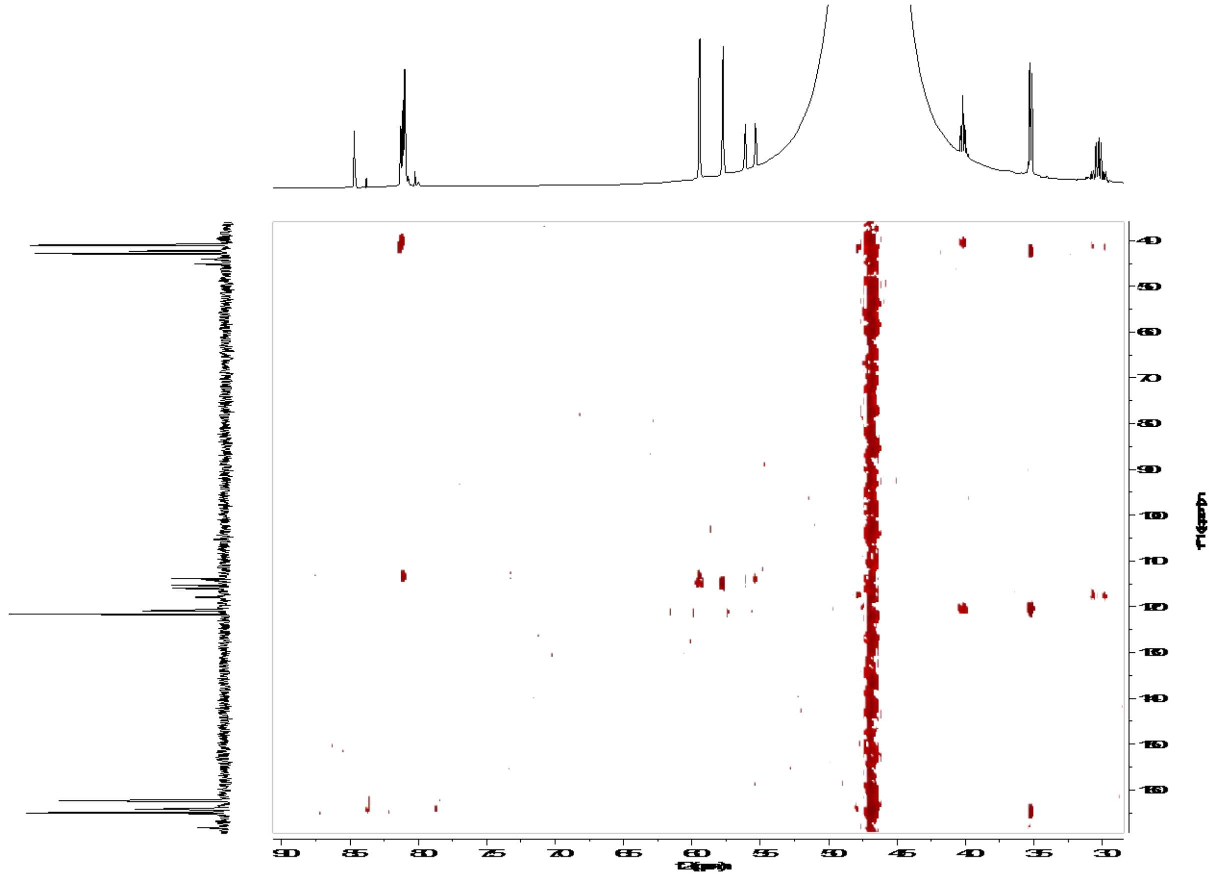

Fig S75. HMBC spectrum for partially reacted FoDHA-CN with ammonia at pH 9.2. Signals for the intermediate and product facilitate the assignments.

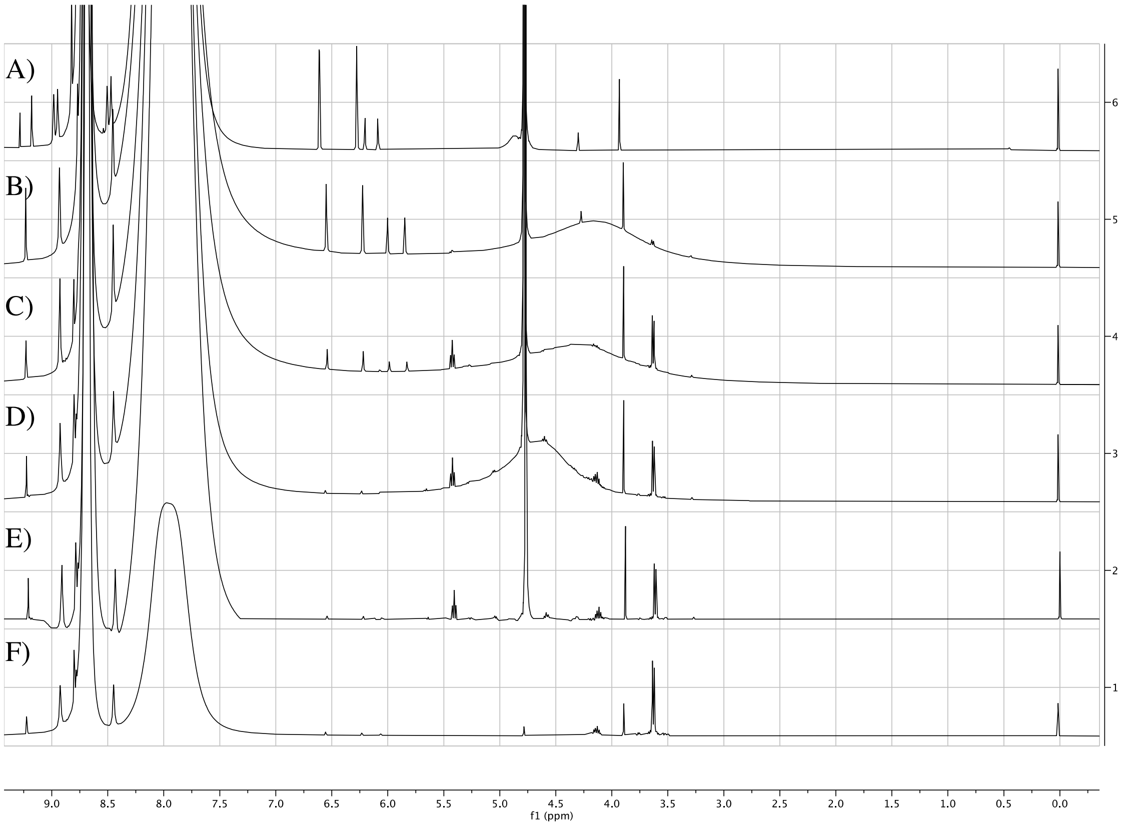

Fig S76. Stack of ^{1}H NMR spectra showing the progress of the reaction between FoDHA-CN and conc. NH_4OH (5 eq.) at room temperature in formamide. A) Before addition of NH_4OH; B) 5min; C) 4h; D) 20h; E) 20 h with baseline correction; F) 20 h with solvent suppression.

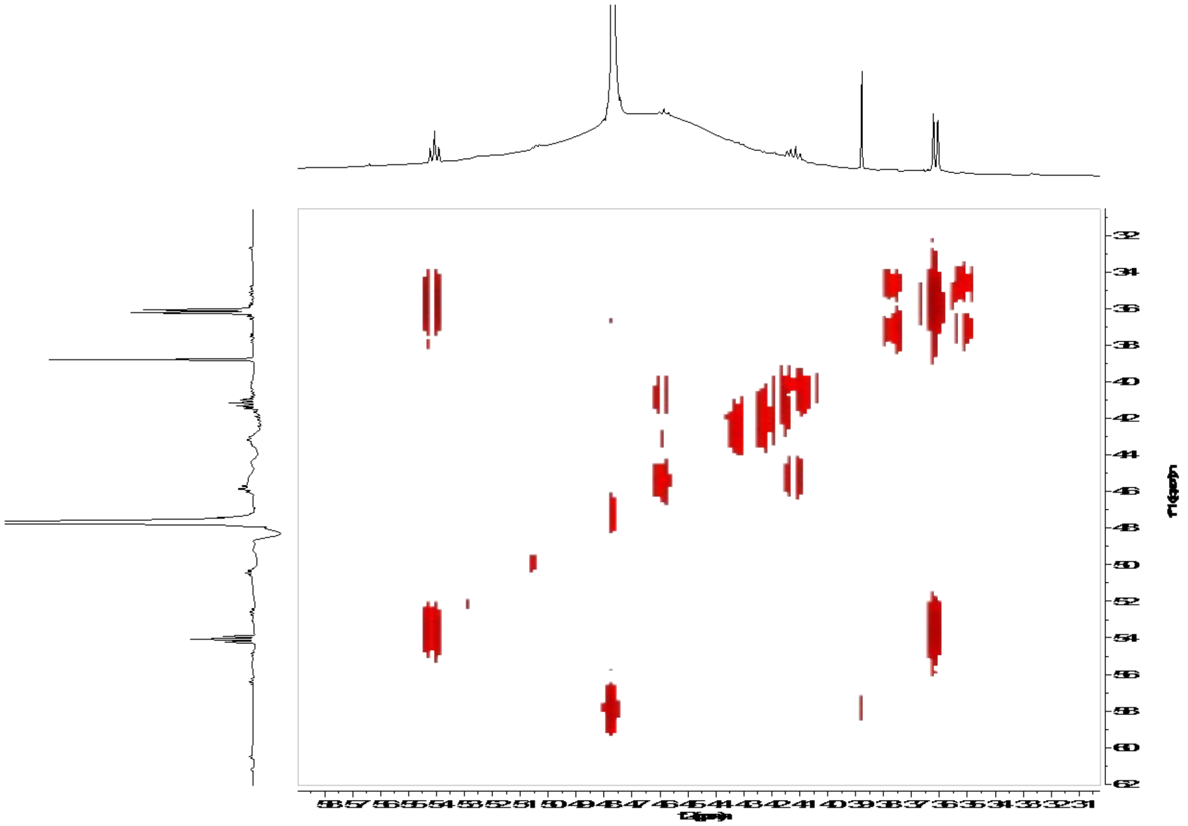

Fig S77. COSY spectrum for the reaction of FoDHA-CN with NH_4OH in formamide (4h). Signals for the intermediate and products facilitate the assignments.

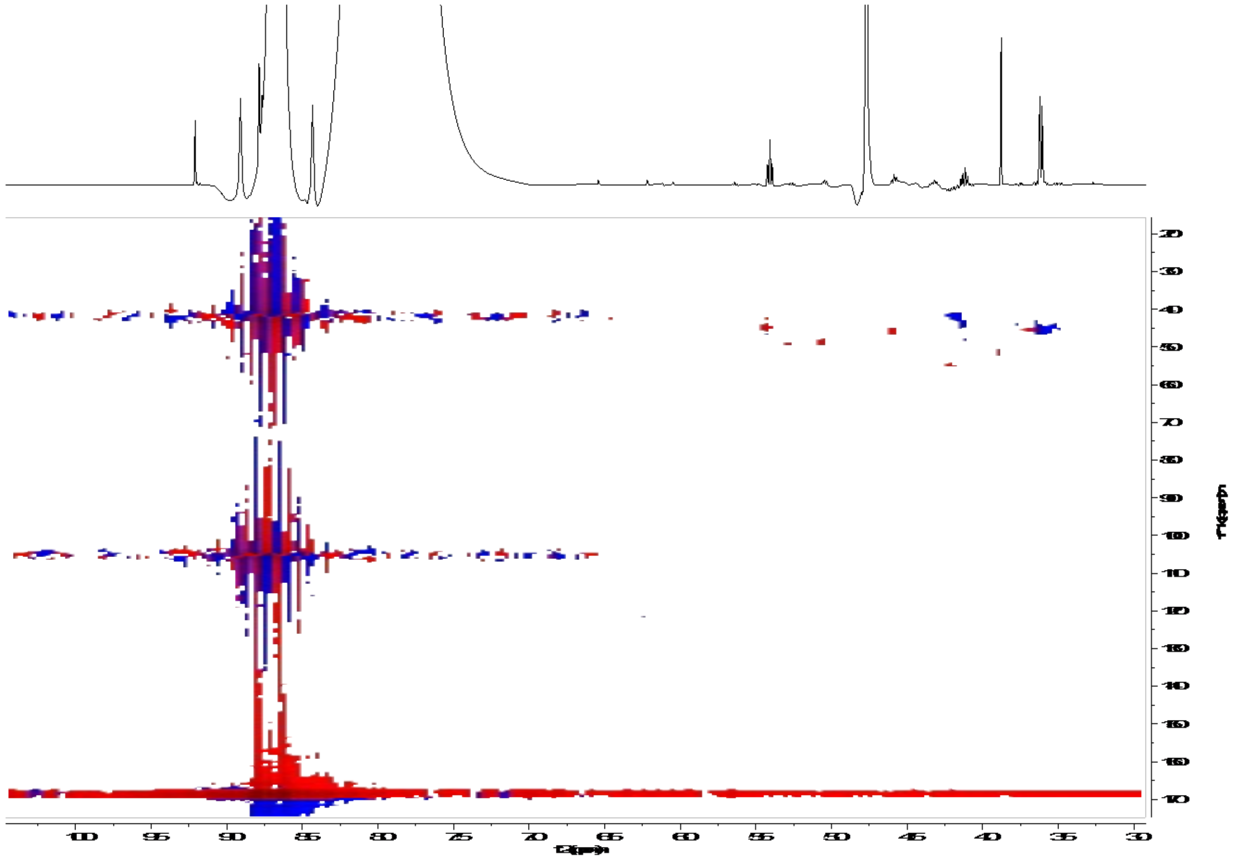

Fig S78. HSQC spectrum of the reaction of FoDHA-CN with NH_4OH in formamide (4h). Signals for the intermediate and products facilitate the assignments.

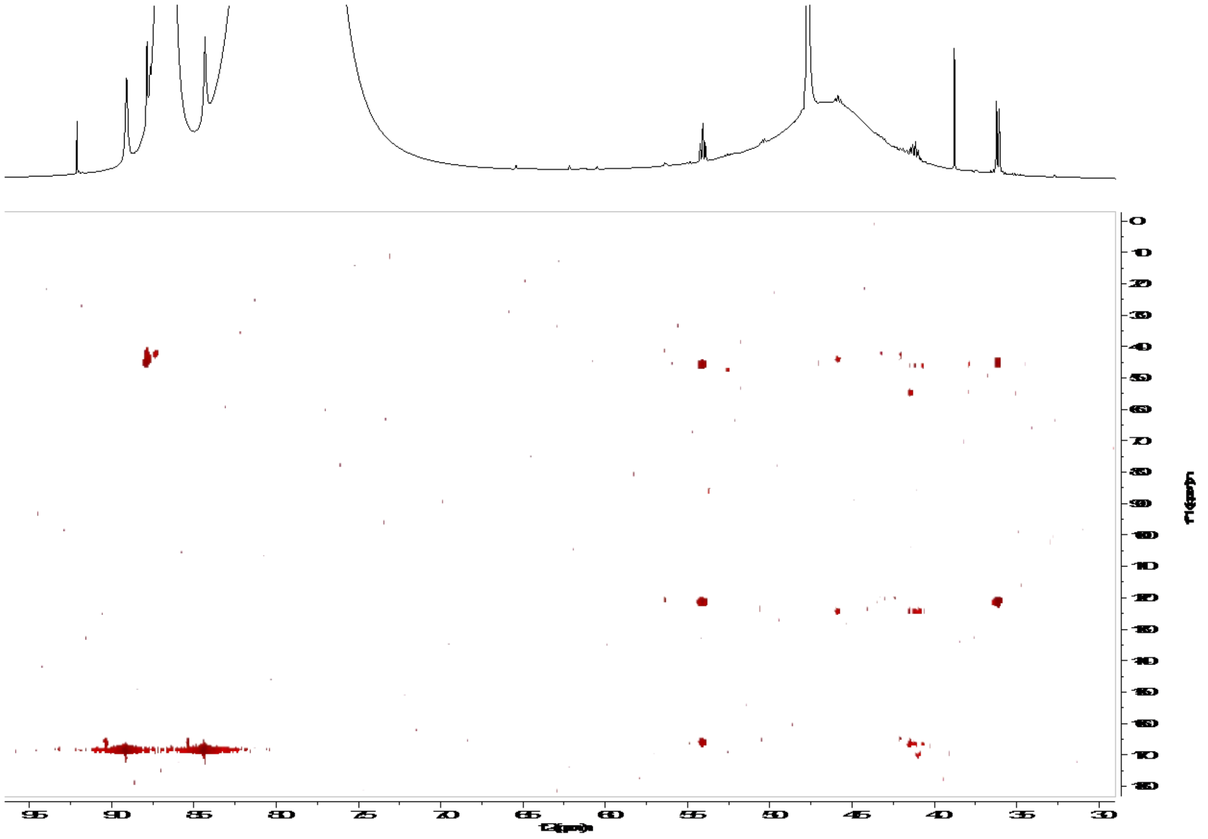

Fig S79. HMBC spectrum of the reaction of FoDHA-CN with NH_4OH in formamide. Signals for the intermediate and products facilitate the assignments.

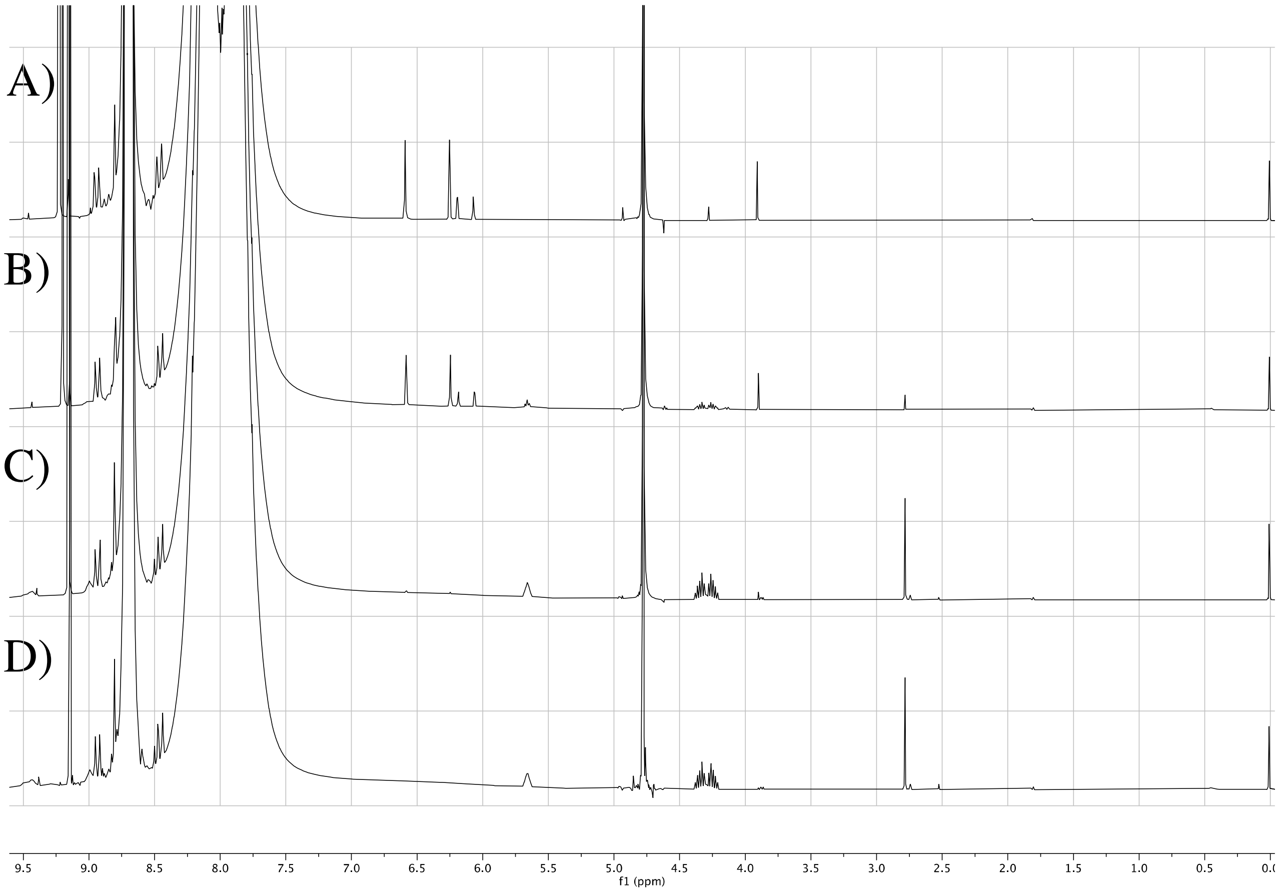

Fig S80. Stack of ^{1}H NMR spectra showing the progress of the reaction between FoDHA-CN with ammonium formate (5 eq.) in formamide. A) Time zero. B) 16h, 50°C C) +16h, 80 °C D) +20h, 80°C.

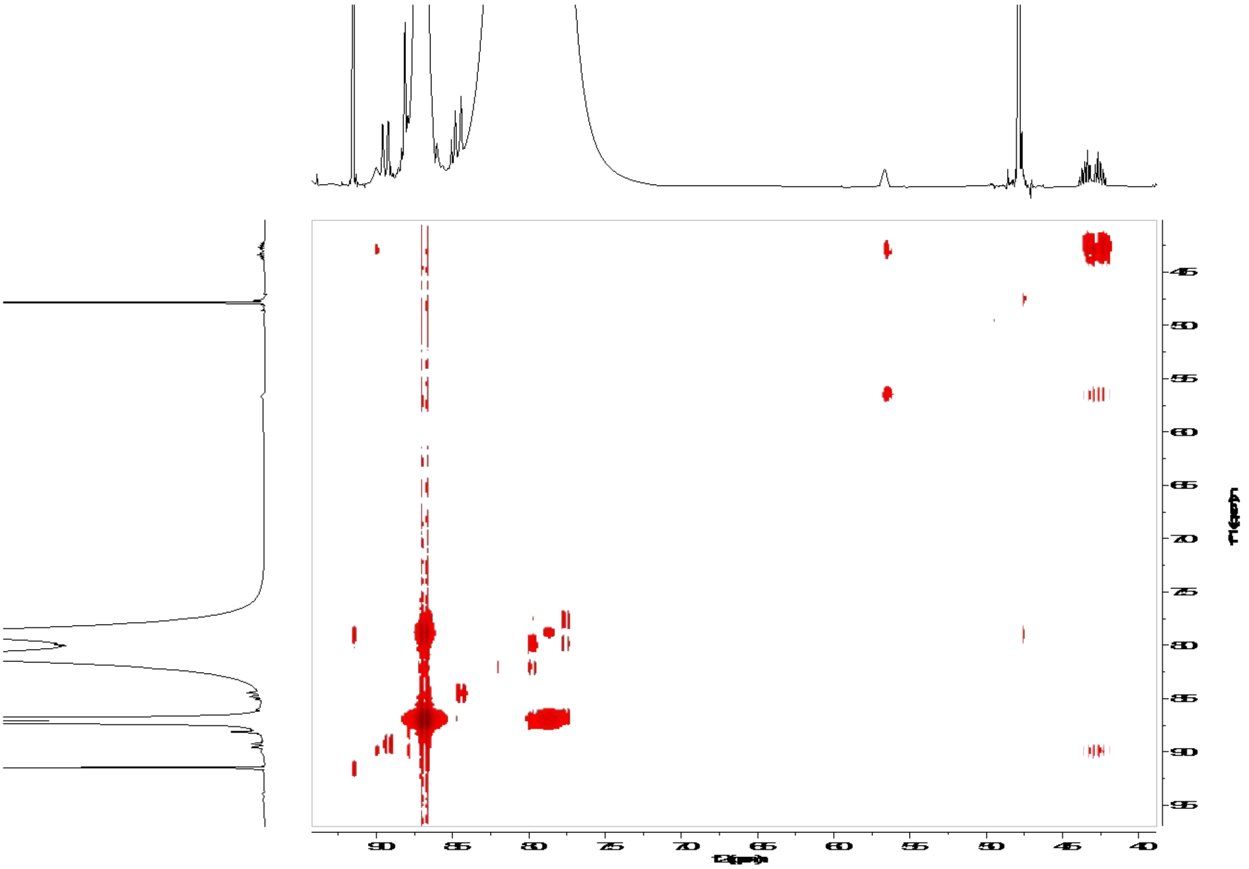

Fig S81. COSY spectrum of the reaction of FoDHA-CN with ammonium formate (5 eq.) in formamide, 36 h 80°C.

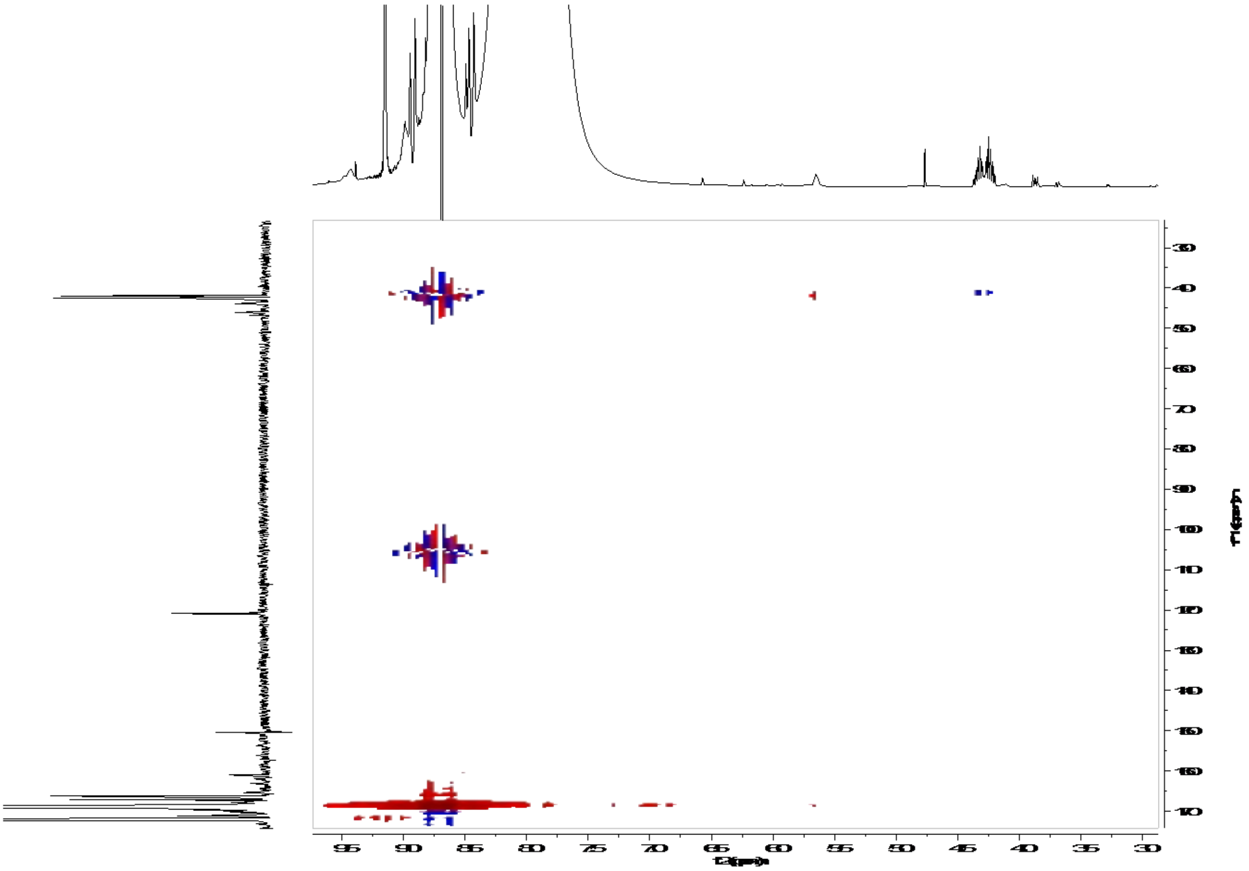

Fig S82. HSQC spectrum of the reaction of FoDHA-CN with ammonium formate (5 eq.) in formamide, 36 h 80°C.

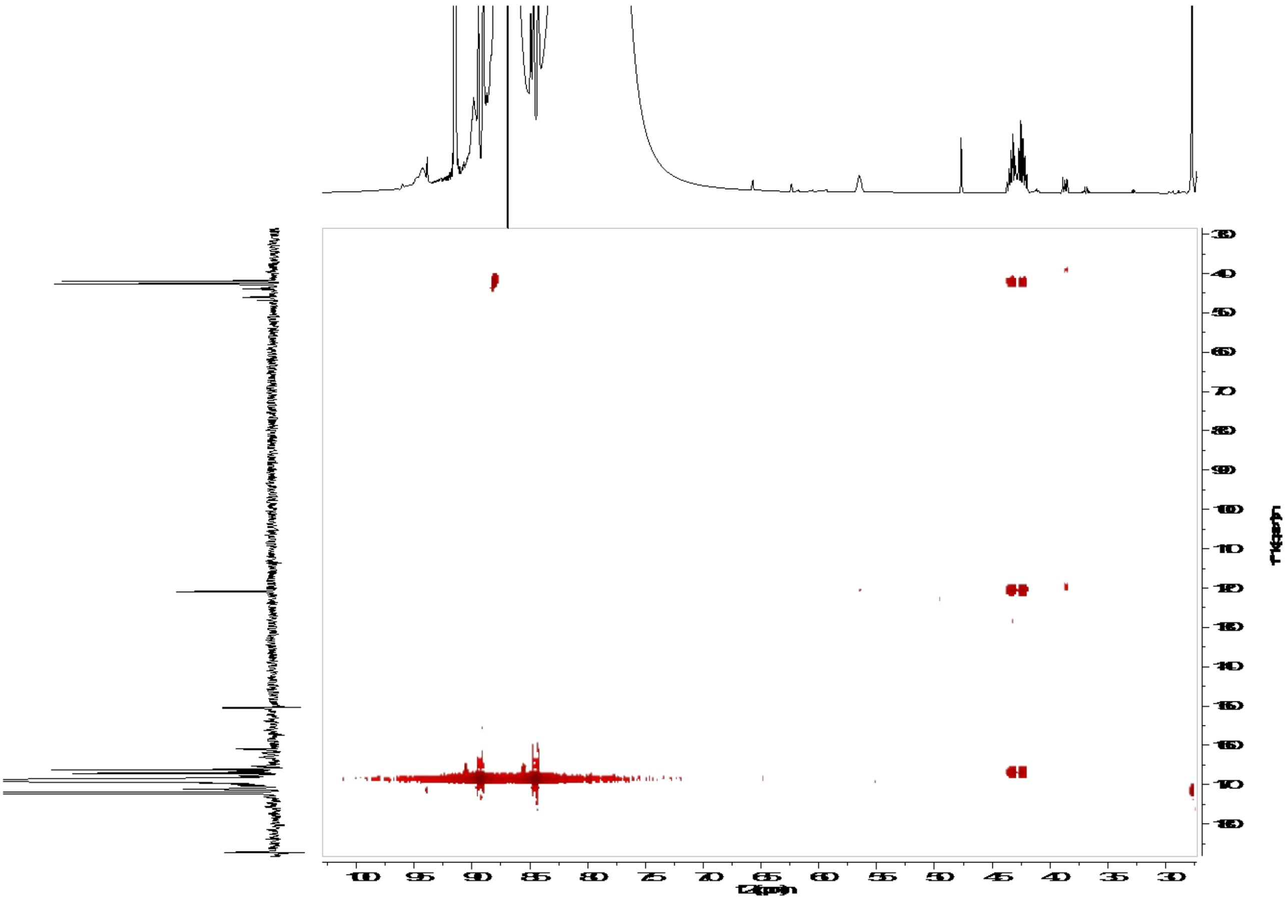

Fig S83. HMBC spectrum of the reaction of FoDHA-CN with ammonium formate (5 eq.) in formamide.

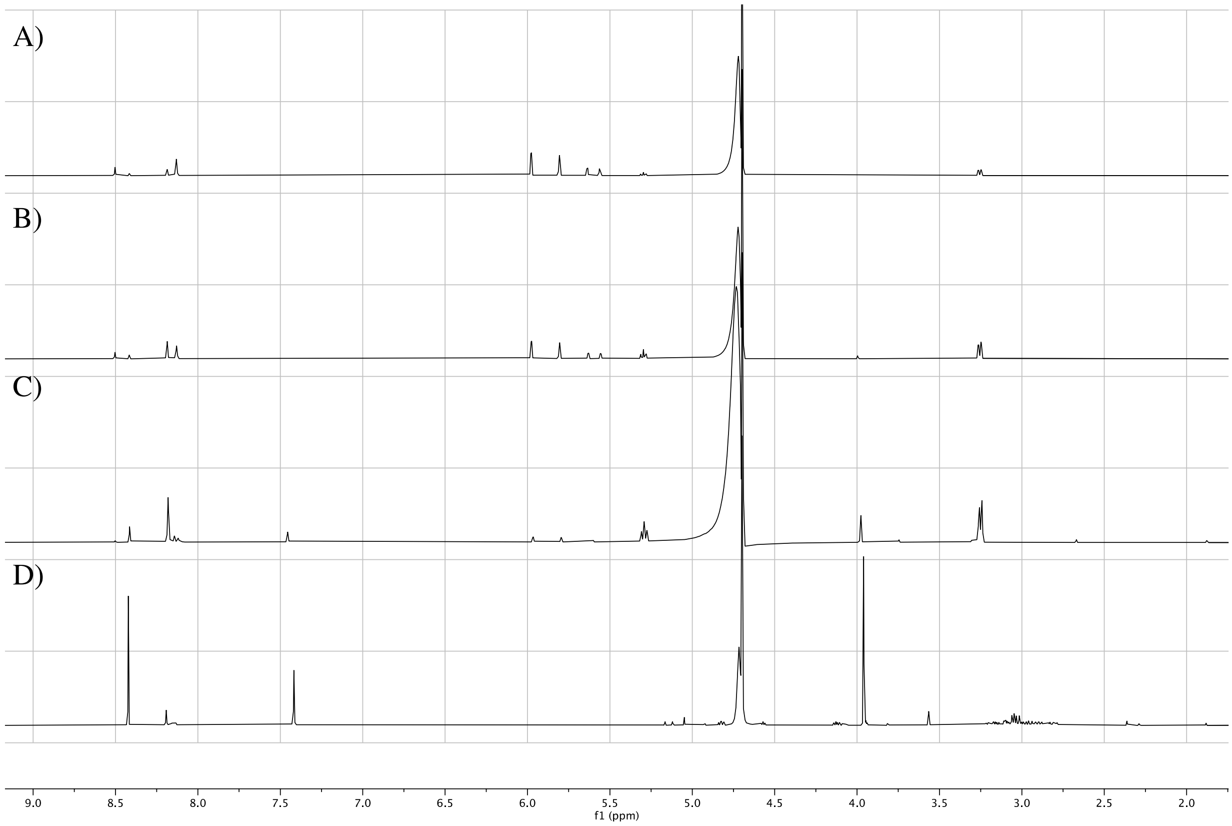

Fig S84. Stack of ^{1}H NMR spectra showing the reaction of FoDHA-CN with NaCN (5.0 equiv.), pH 9.2. A) 1h, RT. The cyanide adduct and starting material are visible; B) 3h, RT. The cyanide adduct, 5-(cyanomethyl)-1H-imidazole-4-carbonitrile and starting material are visible; C) 13 h, RT; D) 96h. Imidazole product and hydration product are visible.

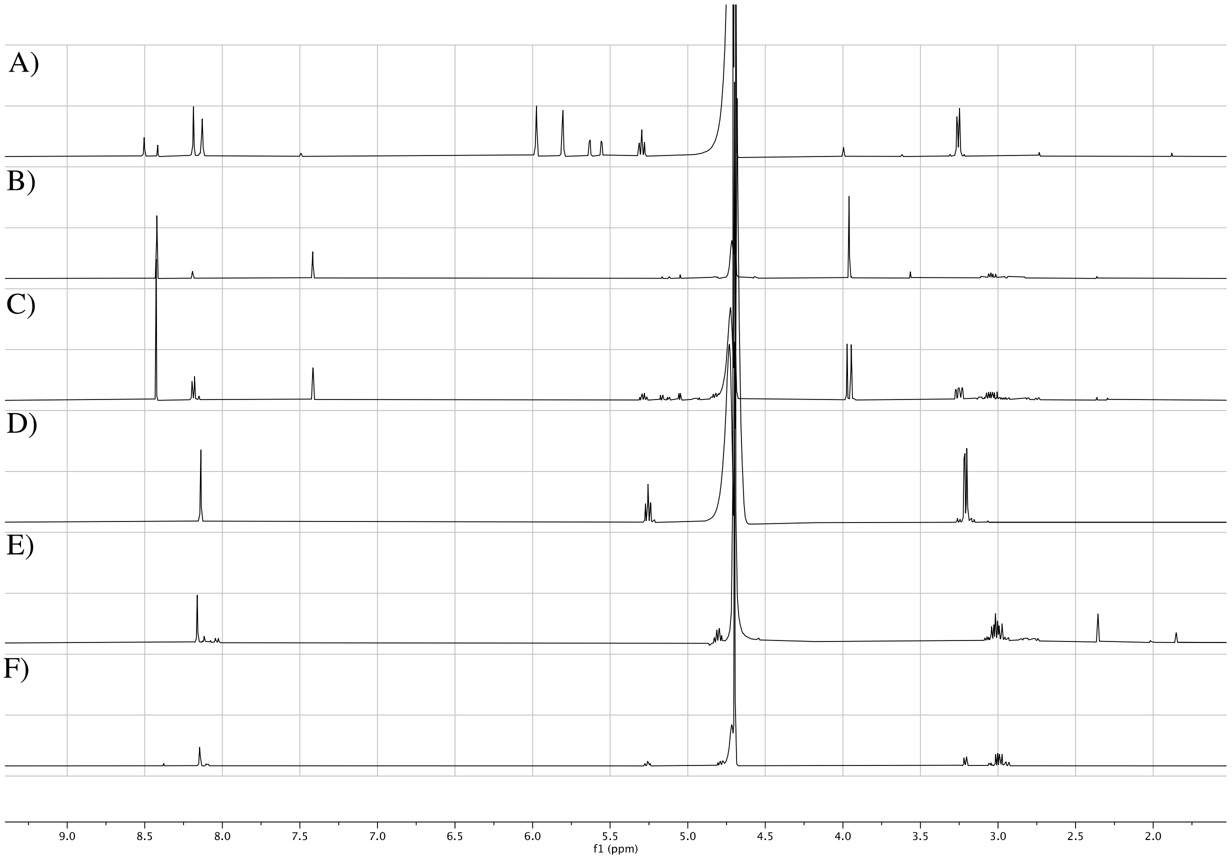

Fig S85: Stack of ^{1}H NMR spectra showing the reaction between FoDHA-CN and NaCN. A) Partial conversion after 16h showing cyanide adduct and trace imidazole product; B) Reaction after 96 h; C) Reaction using labelled $K^{13}CN$ after 48h; coupling to ^{13}C is visible in the products; D) Synthetic standard of cyanide adduct **12**; E) Standard of cyanide addition/α–hydration product amide (^{13}C labelled); F) Mixture of cyanide adduct and α–hydration product amide, generated from prepared cyanide adduct and cyanide at pH 10.

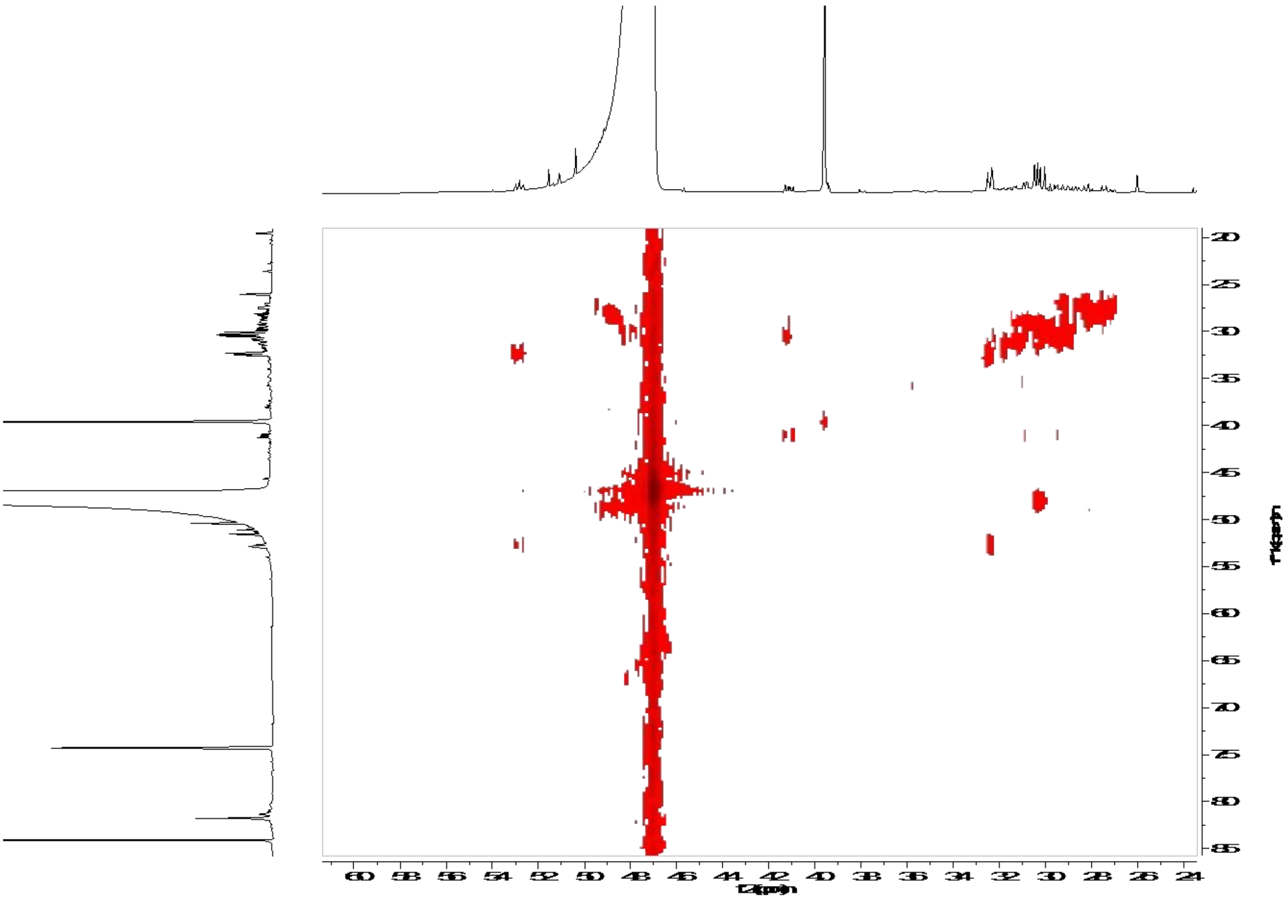

Fig S86. COSY spectrum of the reaction between FoDHACN and NaCN in water, pH 9.2, 72h.

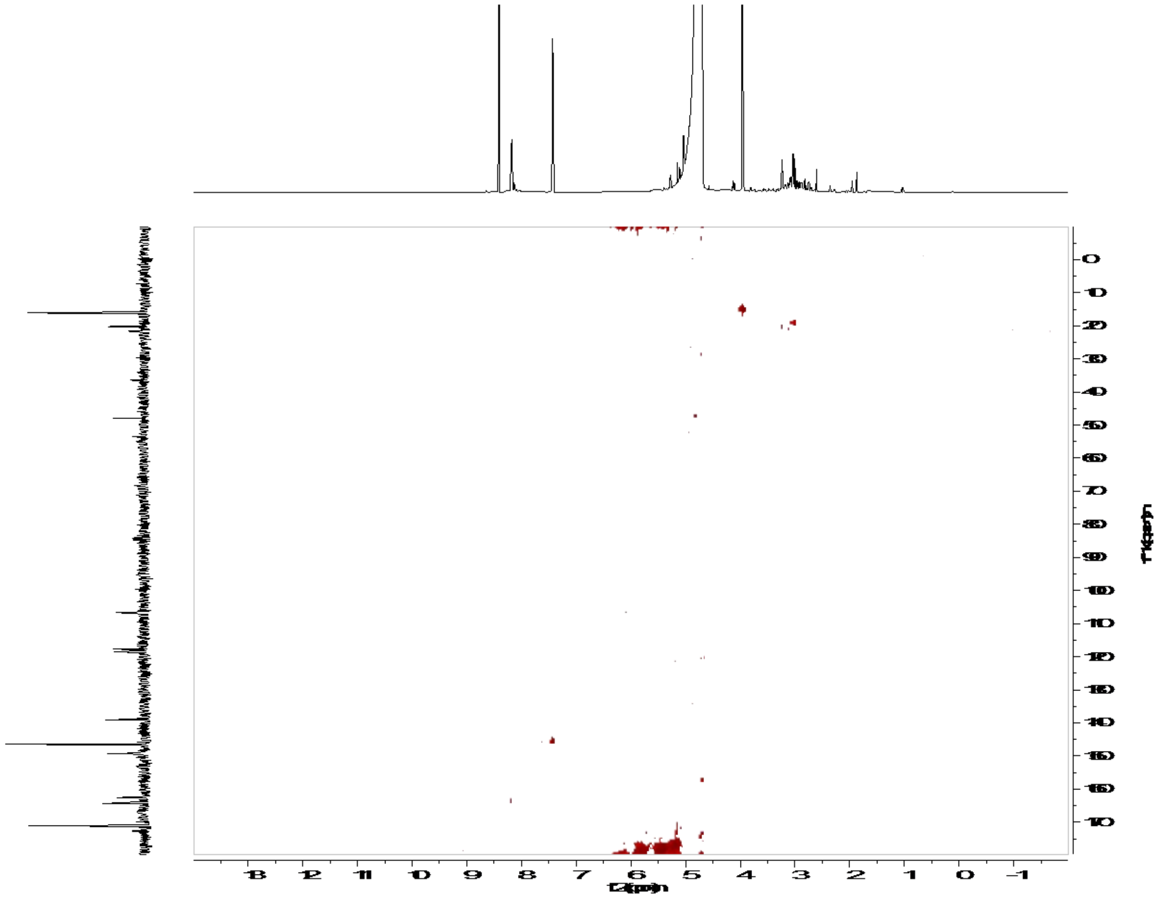

Fig S87. HSQC spectrum of the reaction between FoDHA-CN and NaCN, in water, pH 9.2, 72h.

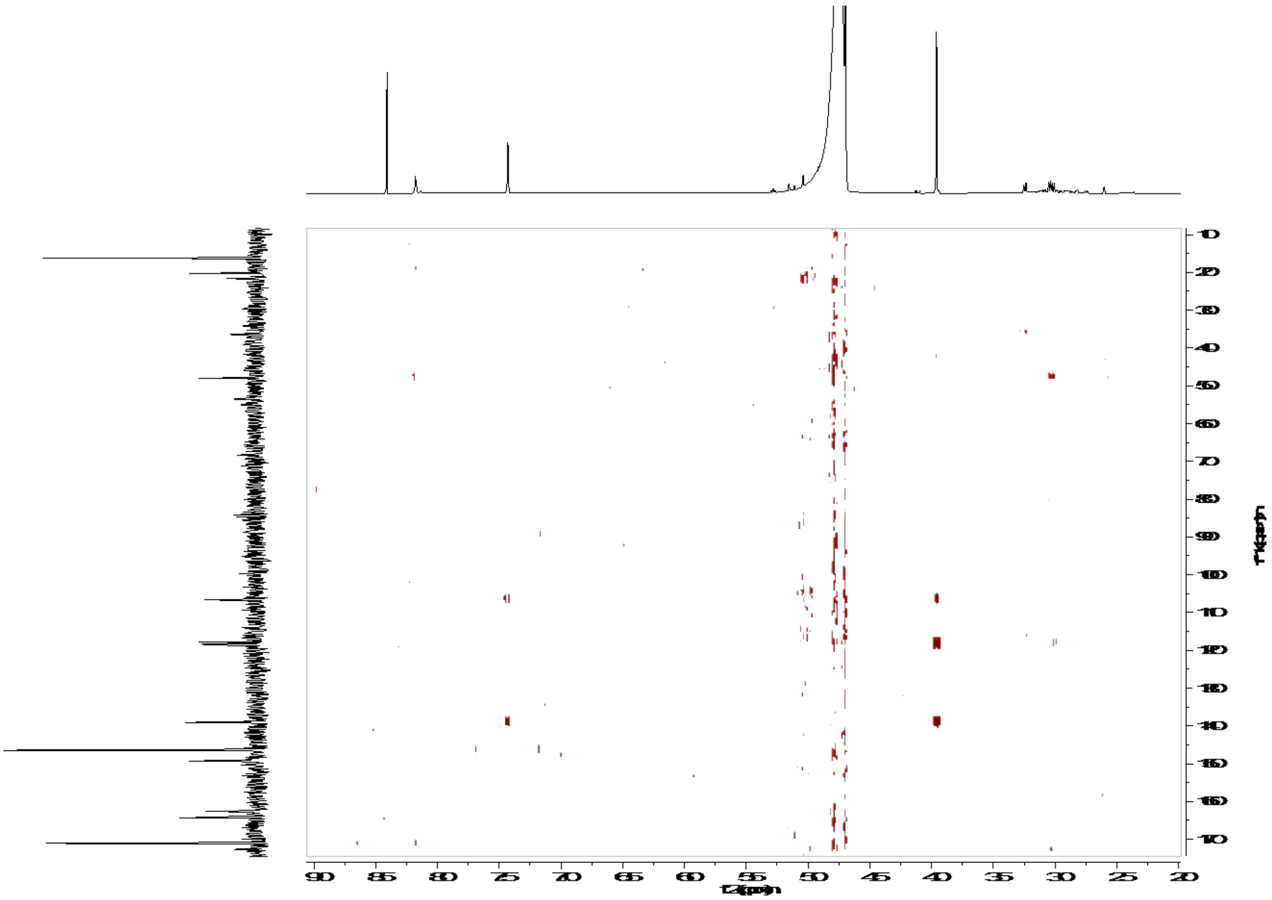

Fig S88. HMBC spectrum of the reaction between FoDHACN and NaCN, in water, pH 9.2, 72h.

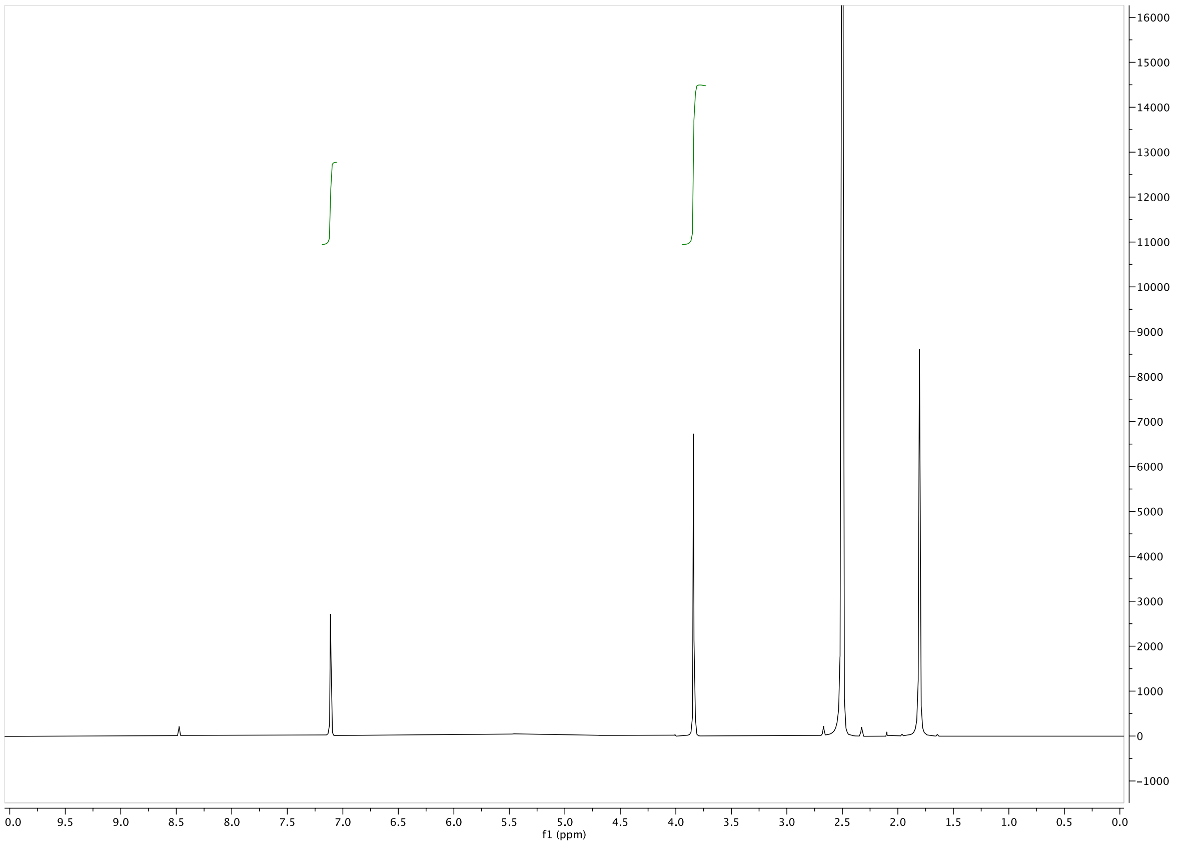

Fig S89. ^{1}H NMR spectrum of 5-(cyanomethyl)-1H-imidazole-4-carbonitrile in DMSO-d_6. Unmarked peaks are acetate and trace formate.

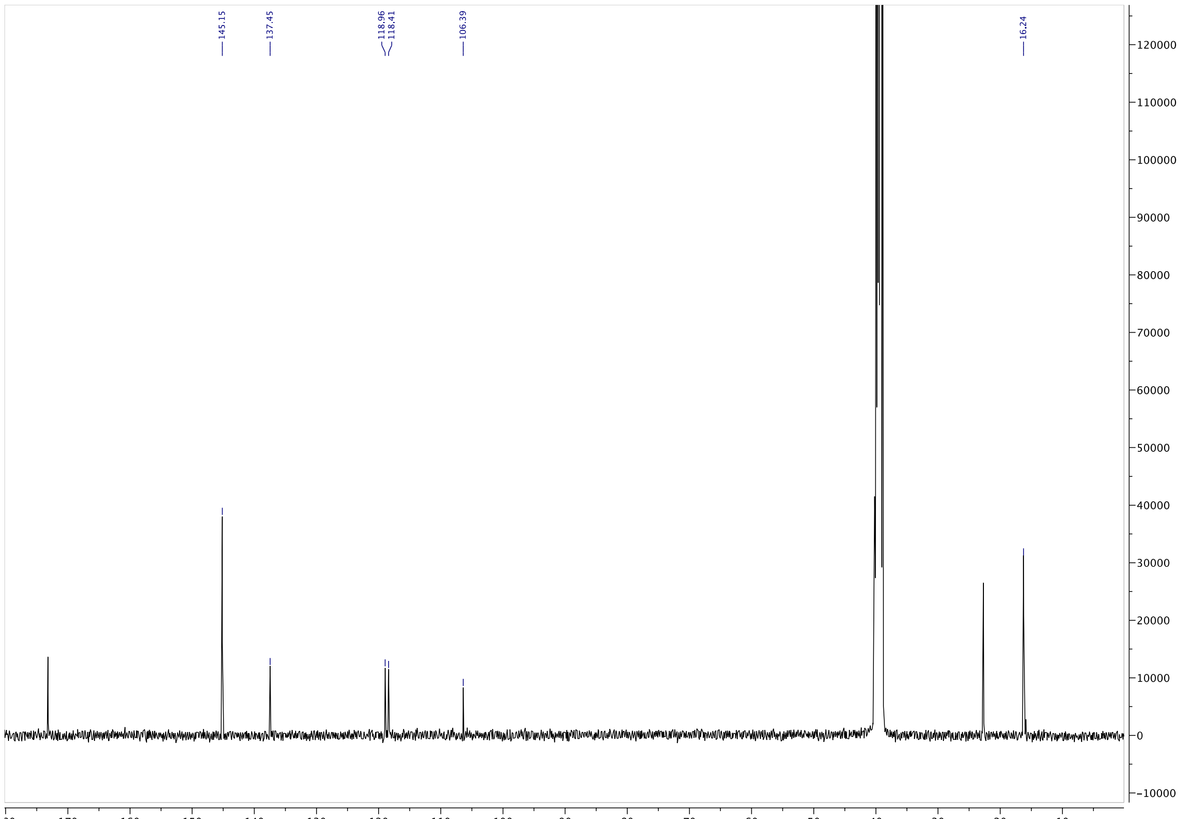

Fig S90. ^{13}C NMR spectrum of 5-(cyanomethyl)-1H-imidazole-4-carbonitrile in DMSO-d_6. Unmarked peaks are acetate.

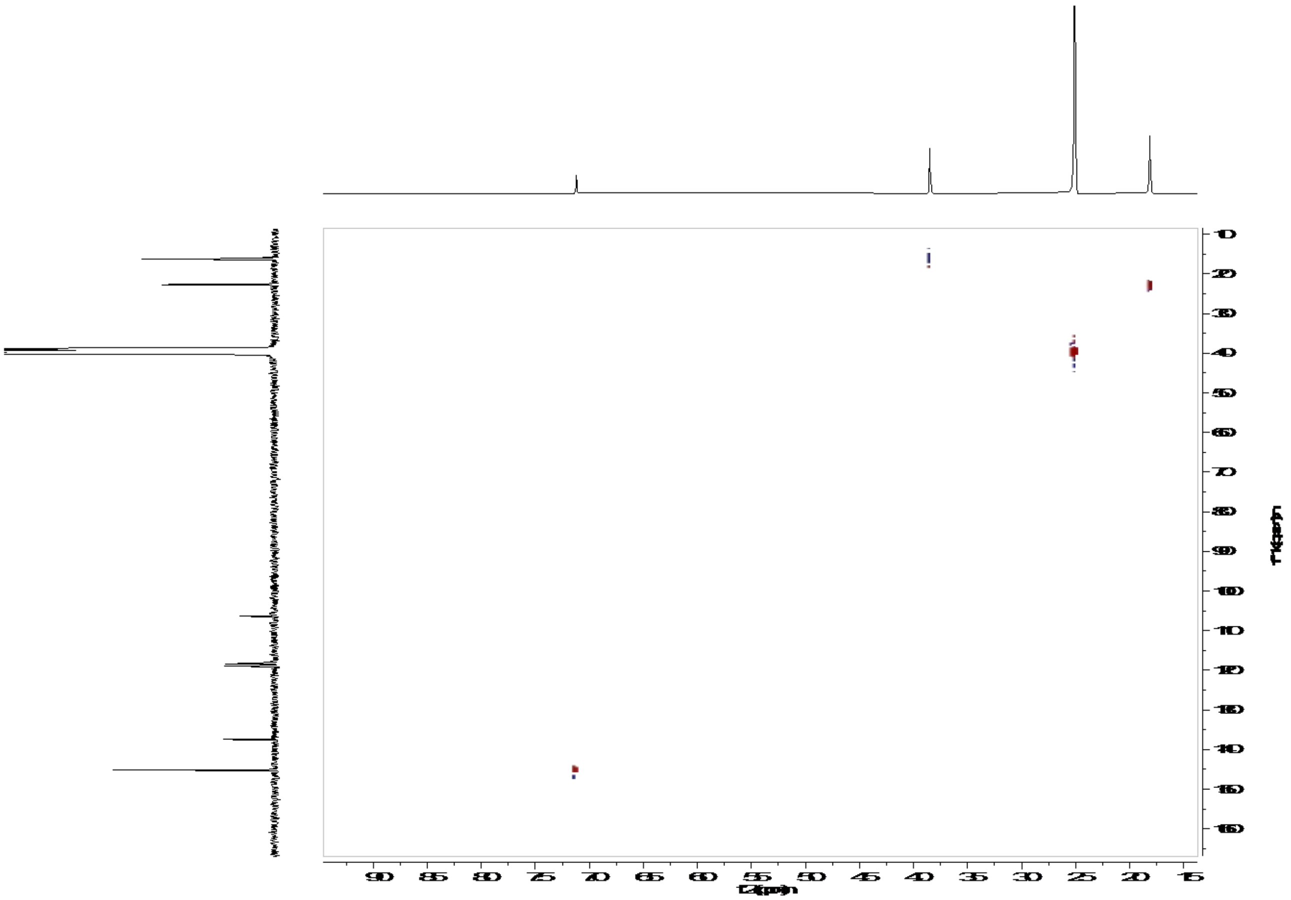

Fig S91. HSQC spectrum of 5-(cyanomethyl)-1H-imidazole-4-carbonitrile in DMSO-d$_6$.

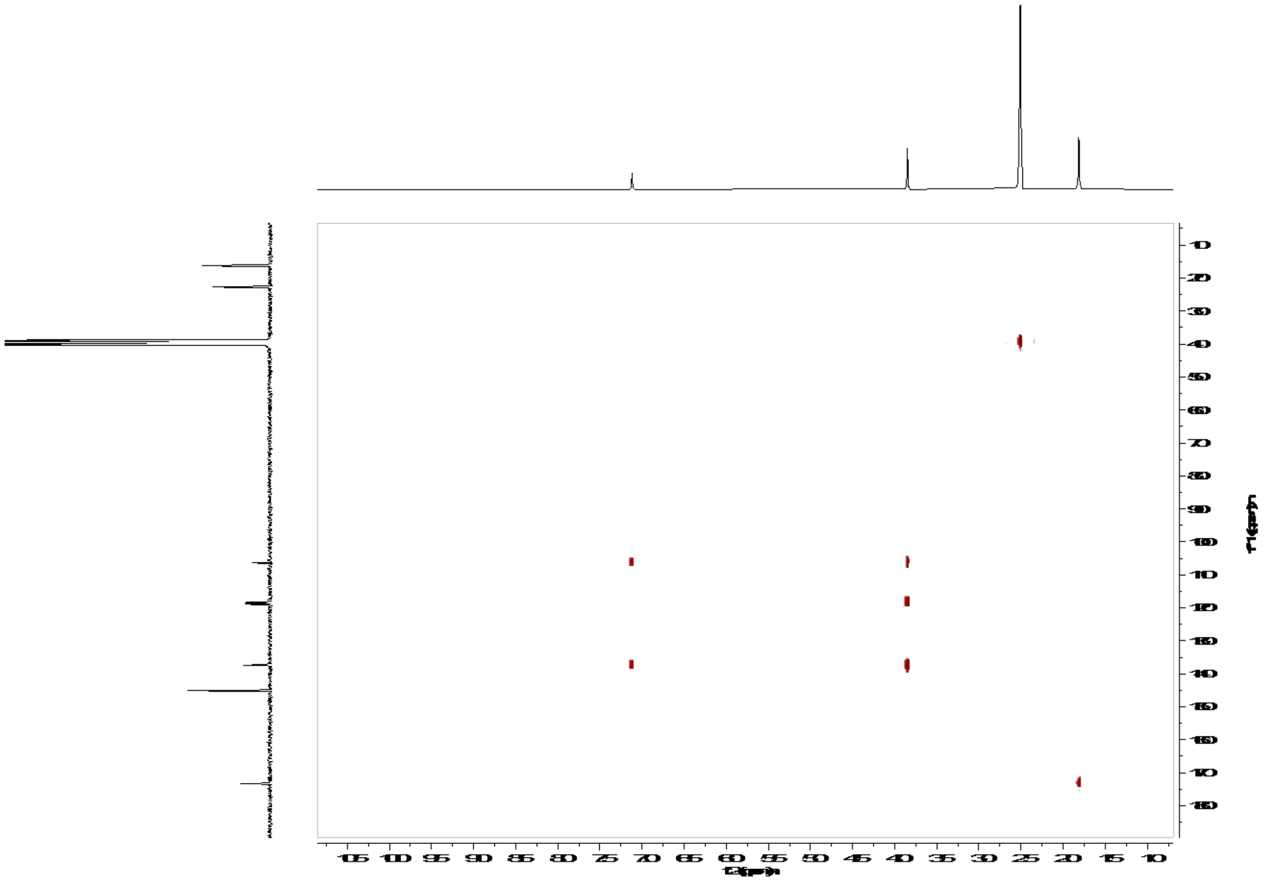

Fig S92. HMBC spectrum of 5-(cyanomethyl)-1H-imidazole-4-carbonitrile in DMSO-d$_6$.

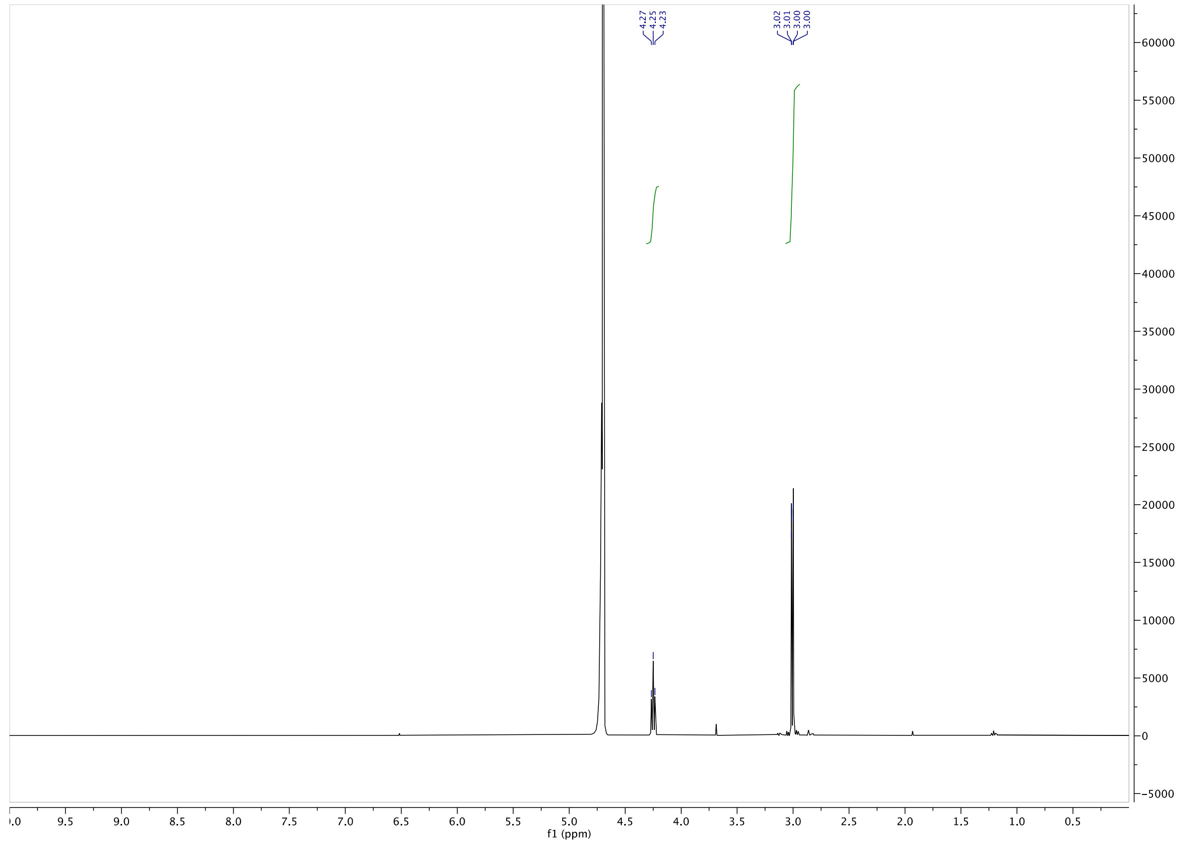

Fig S93. ^{1}H NMR spectrum of (β-CN)Ala-CN in water.

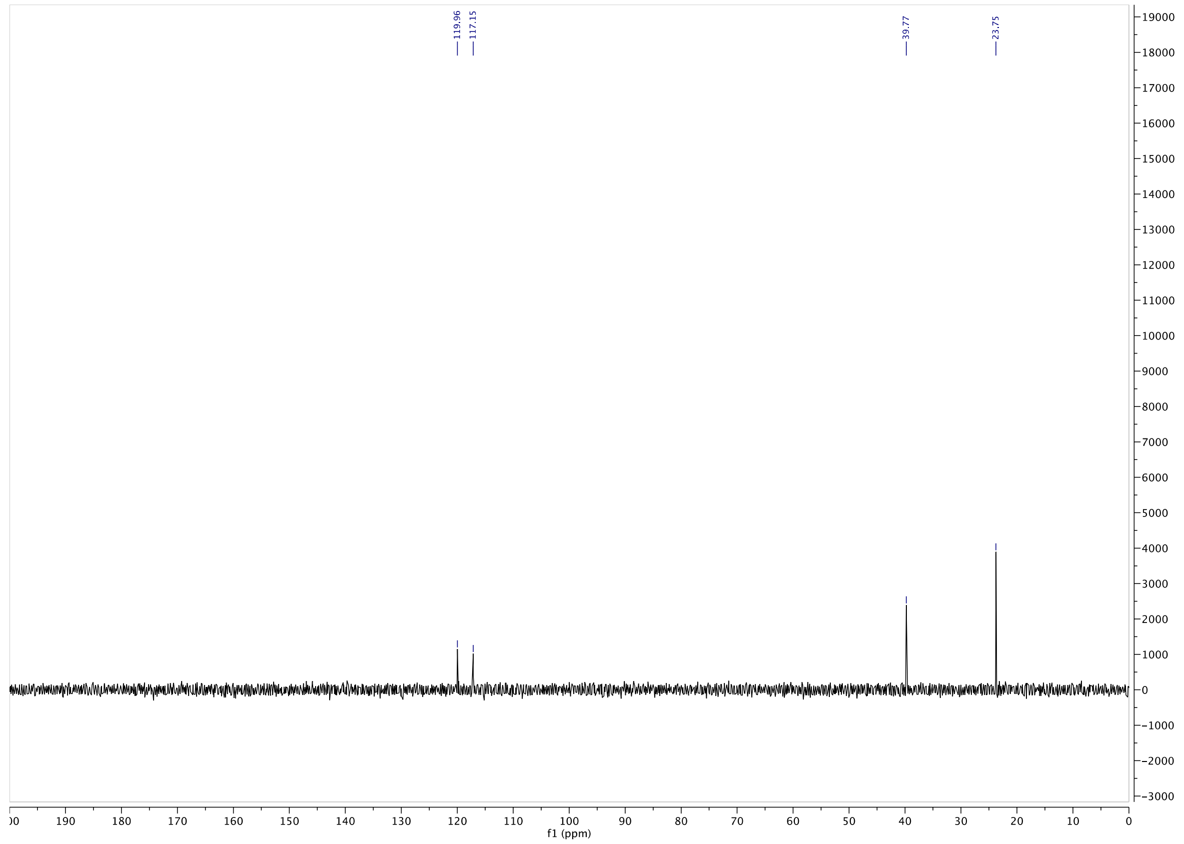

Fig S94. ^{13}C NMR spectrum of (β-CN)Ala-CN in water.

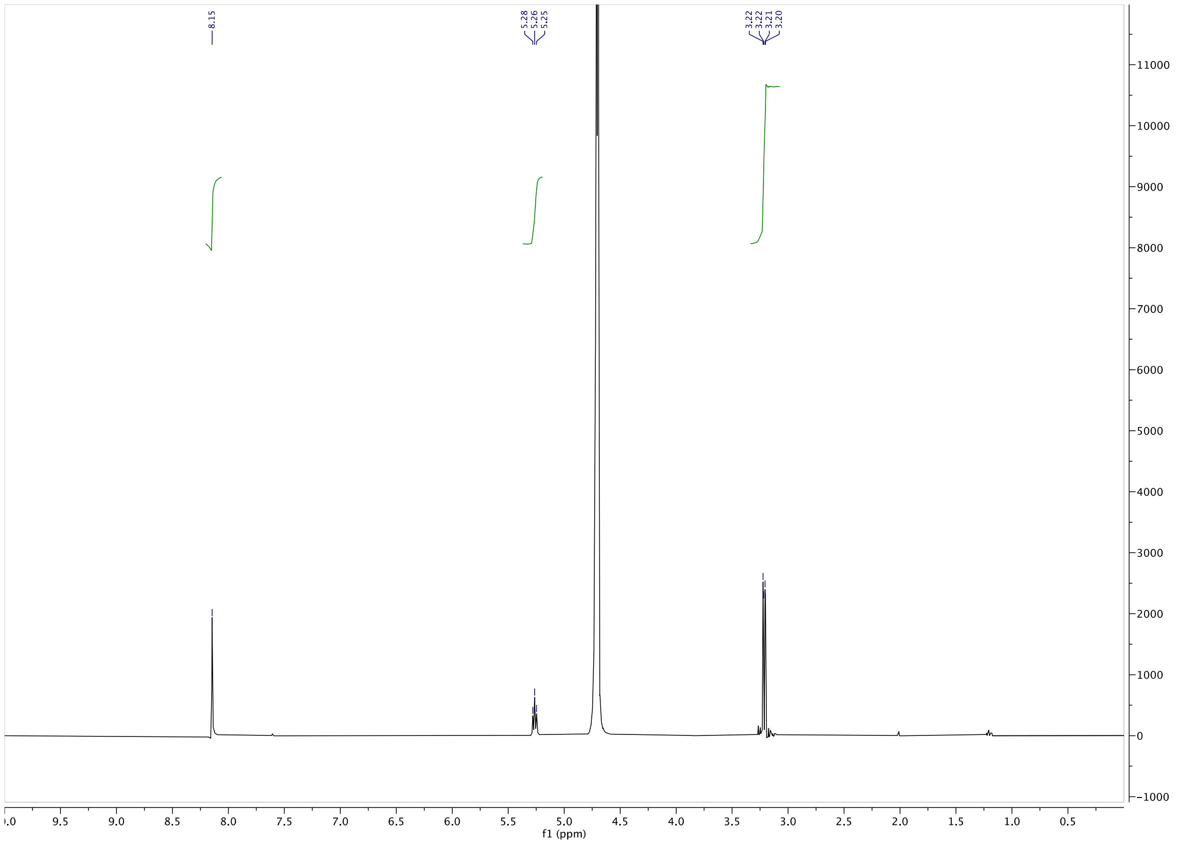

Fig S95. ^{1}H NMR spectrum of Fo(β-CN)Ala-CN in water.

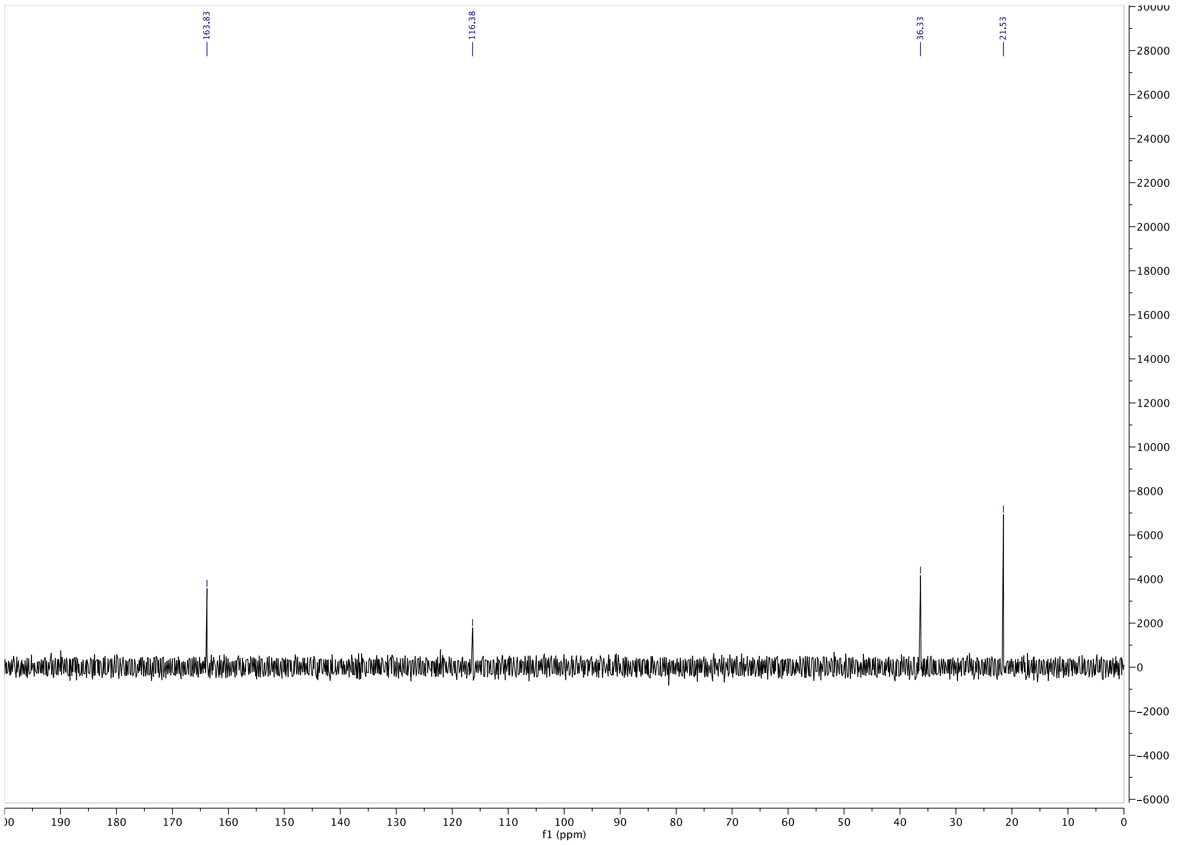

Fig S96. ^{13}C NMR spectrum of Fo(β-CN)Ala-CN acid dinitrile in water.

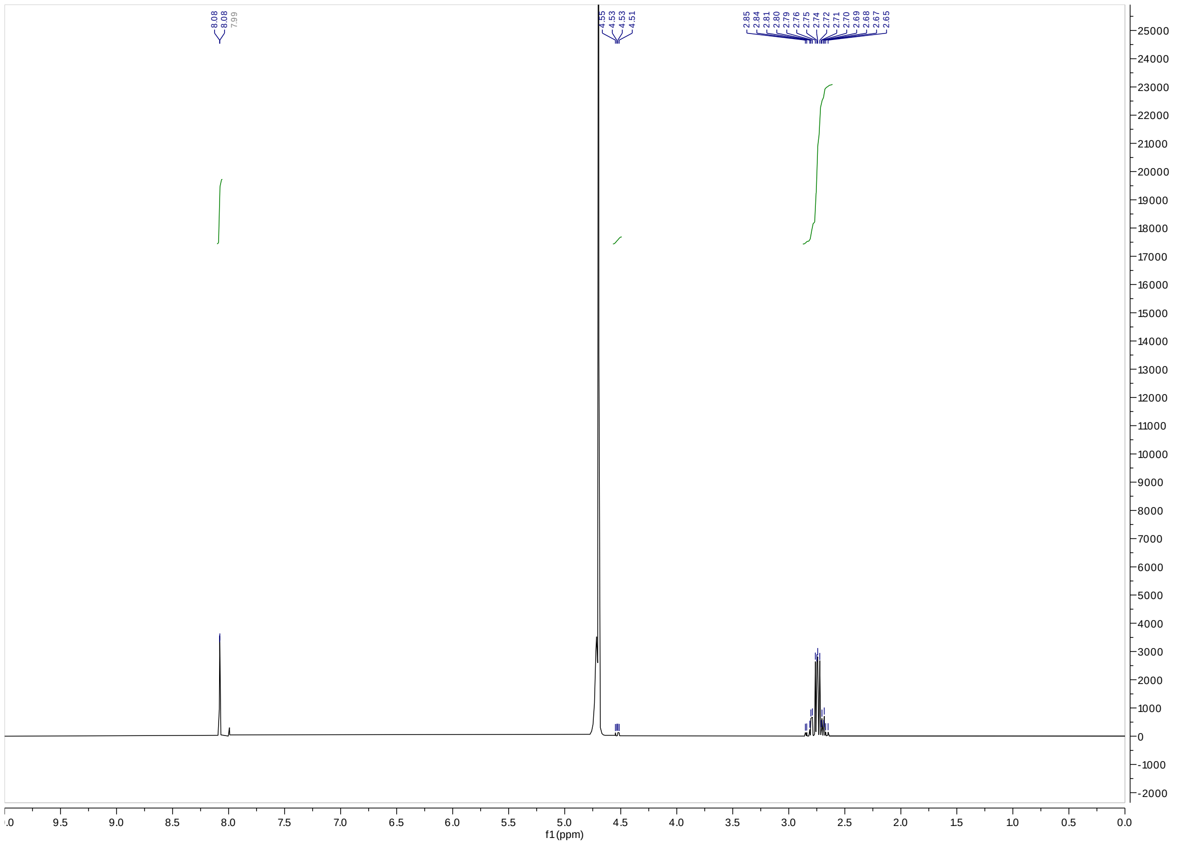

Fig S97. ^{1}H NMR spectrum of (N-formyl)asparaginamide in water.

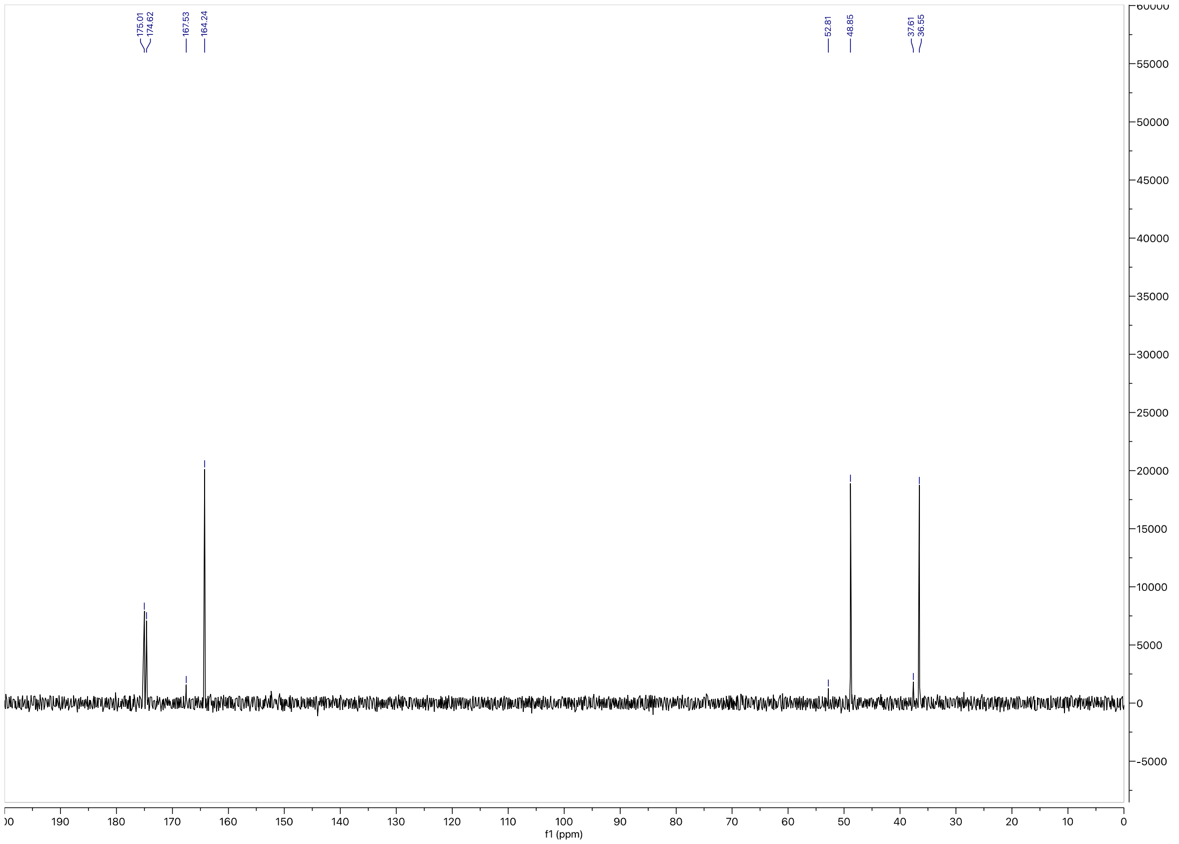

Fig S98. ^{13}C NMR spectrum of (N-formyl)asparaginamide in water.

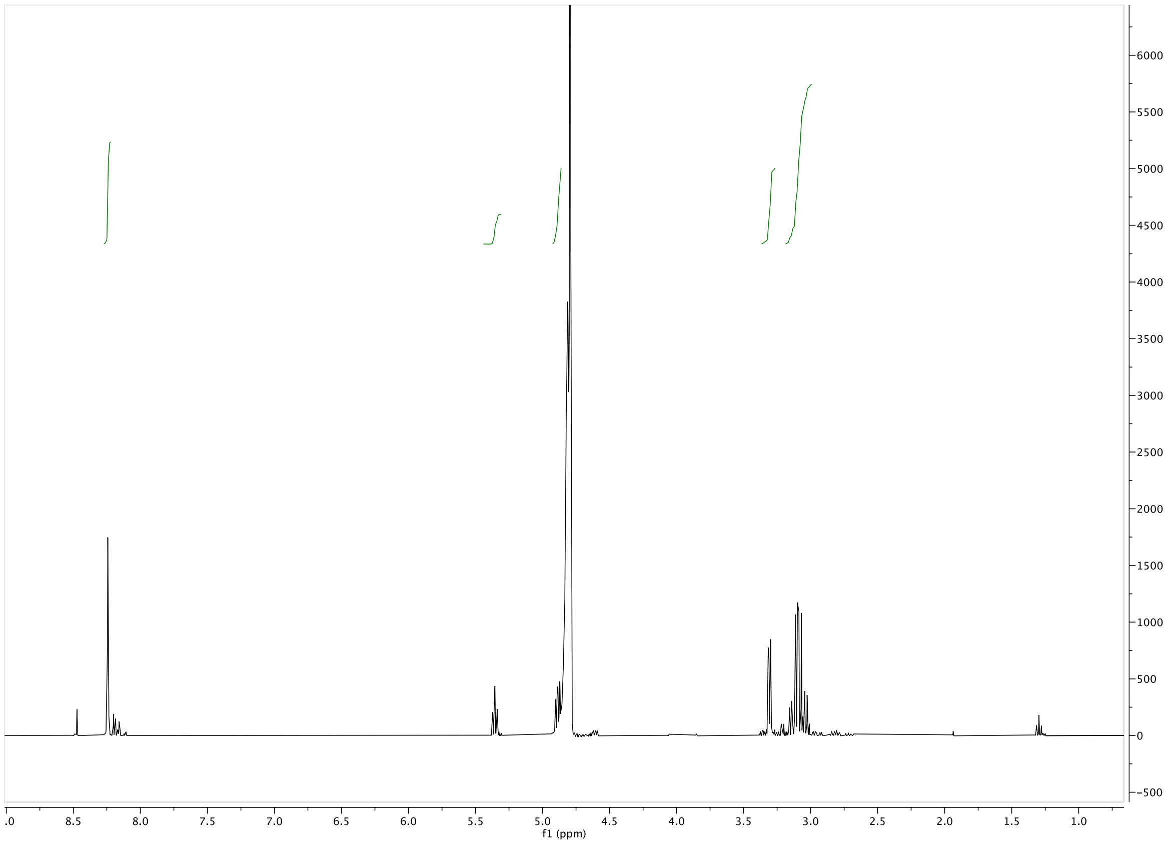

Fig S99. ^{1}H NMR spectrum showing partial hydration of N-formyl aspartate dinitrile to Fo(β-CN)AlaNH$_2$ (24 days, pH 10, RT).

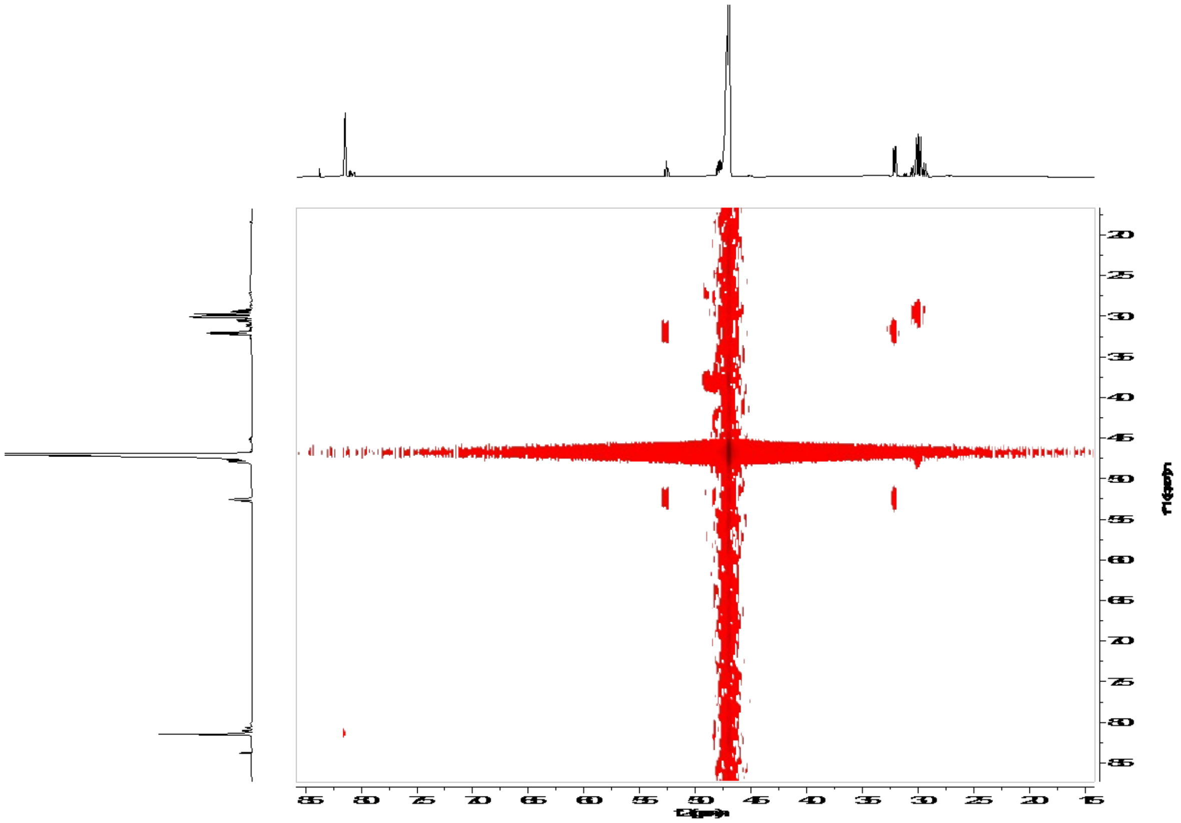

Fig S100. COSY spectrum showing partial hydration of N-formyl aspartate dinitrile to Fo(β-CN)AlaNH$_2$.

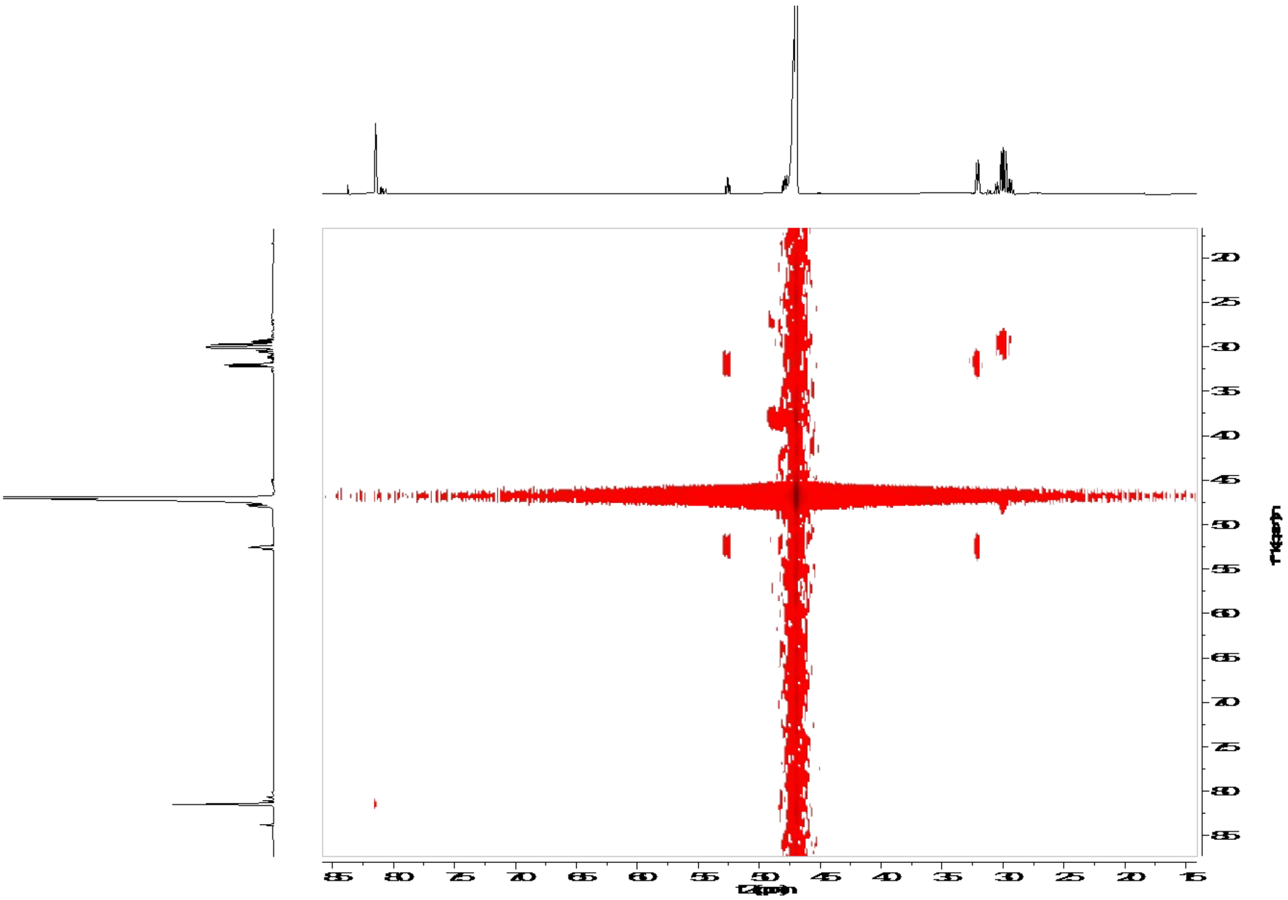

Fig S101. HSQC spectrum showing partial hydration of N-formyl aspartate dinitrile to Fo(β-CN)AlaNH$_2$.

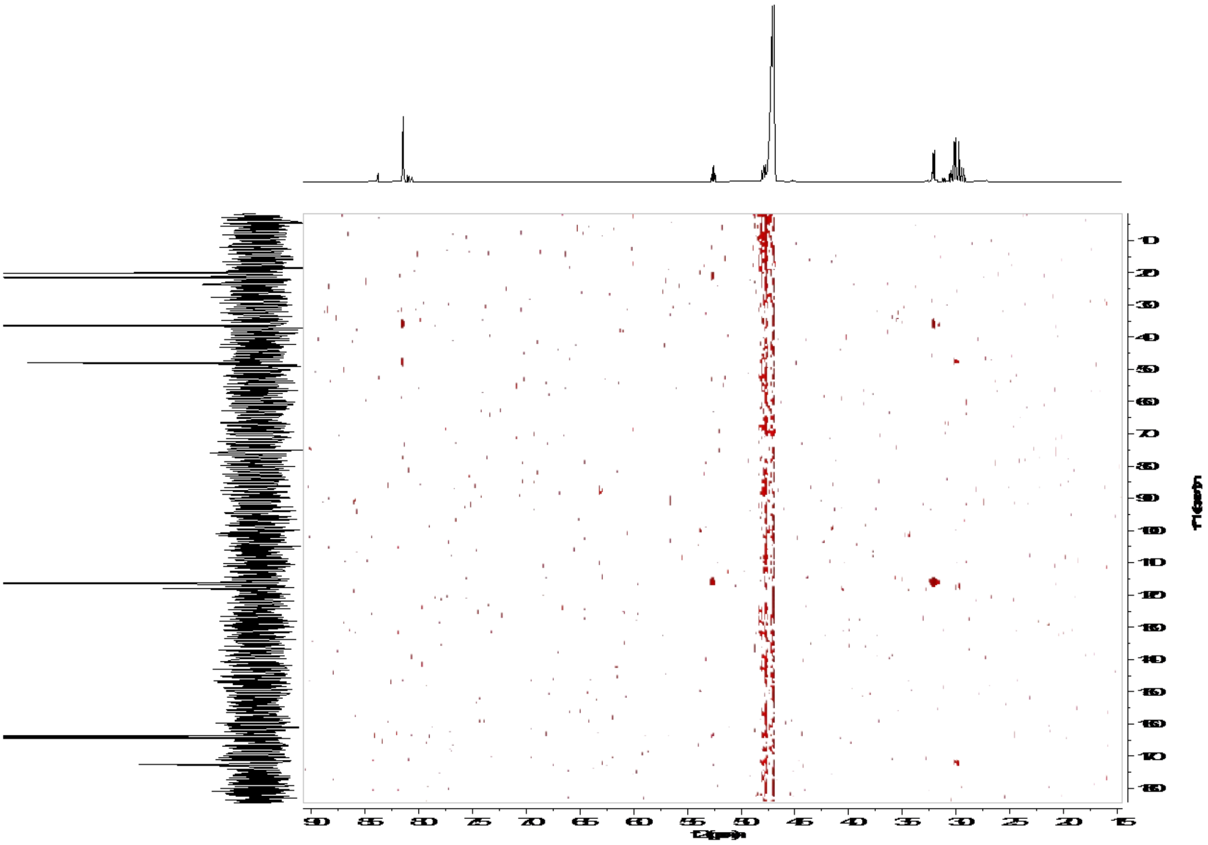

Fig S102. HMBC spectrum showing partial hydration of N-formyl aspartate dinitrile to Fo(β-CN)AlaNH$_2$.

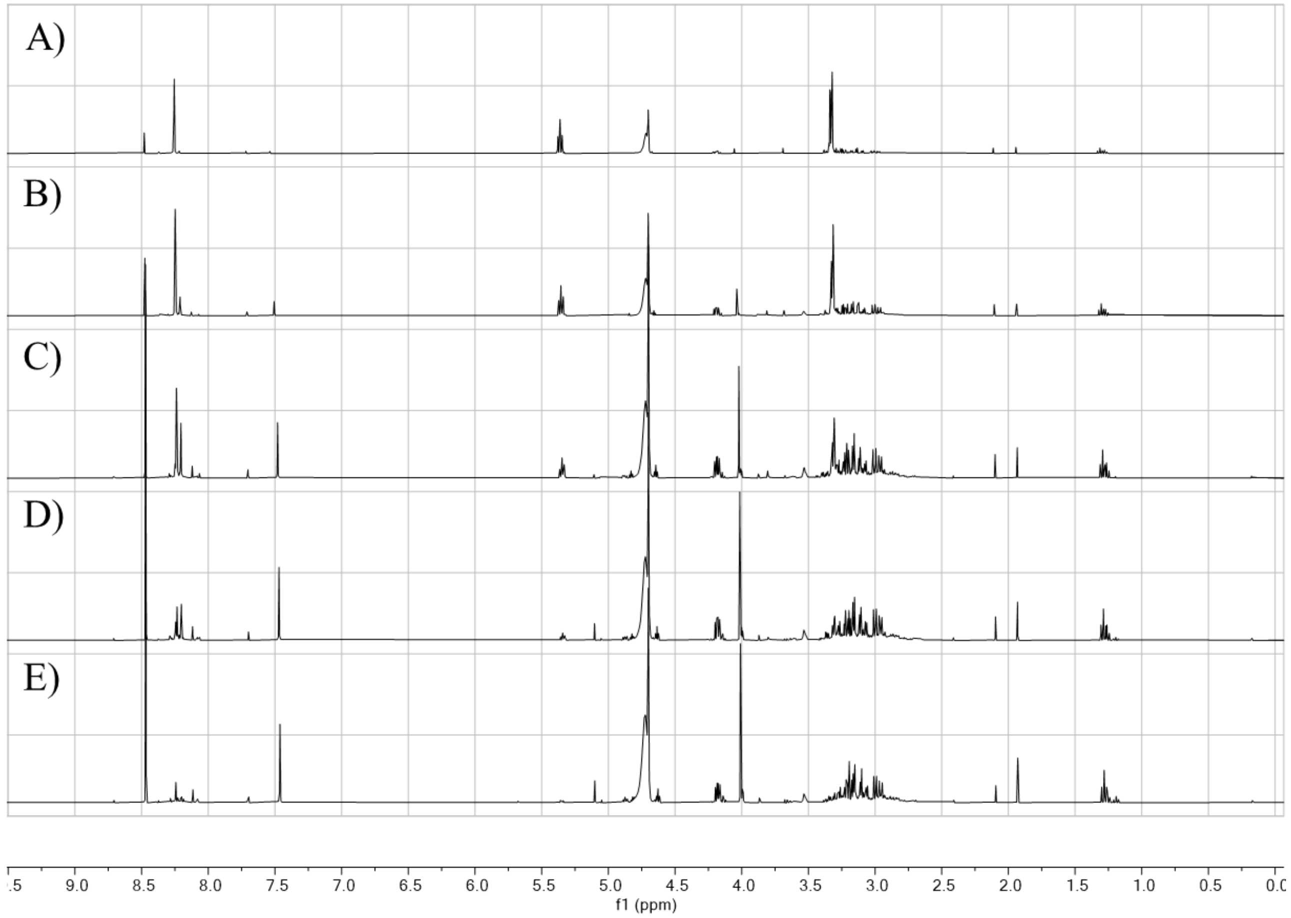

Fig S103. Stack of ^{1}H NMR spectra showing the reaction of (*N*-formyl)aspartic acid dinitrile with NaCN (3.0 equiv.), pH 9.2, room temperature. A) 30 minutes; B) 2 hr; C) 6 hr; D) 12 hr; E) 24 hr.

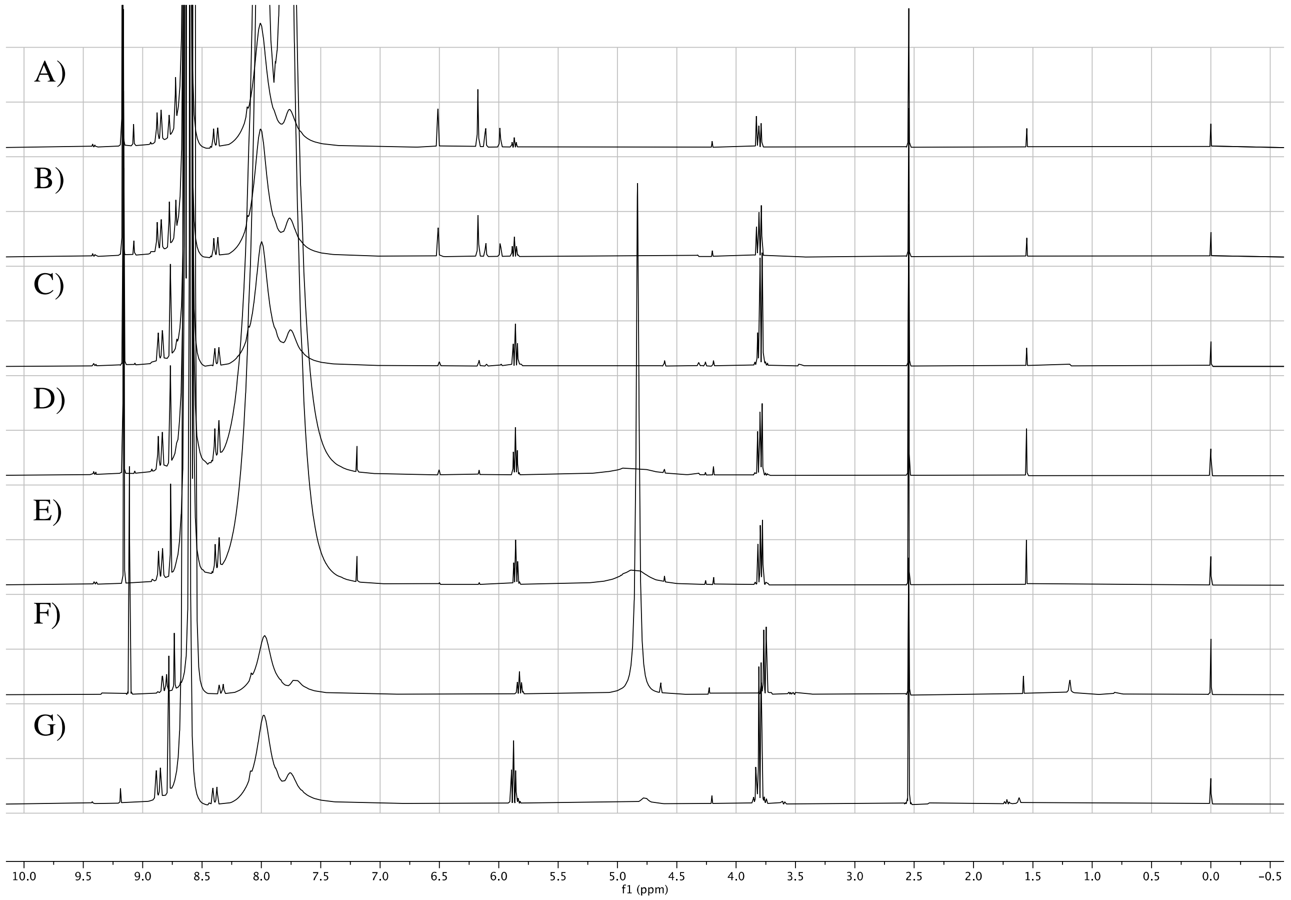

Fig S104. Stack of ^{1}H NMR spectra showing reaction of FoDHA-CN with NaCN (3.0 eq.) and formic acid (3.0 eq.) in formamide. A) 1h; B) 2h; C) 4h; D) 12h; E) 20h; F) 14 days; G) Standard of Fo(β-CN)AlaCN in formamide.

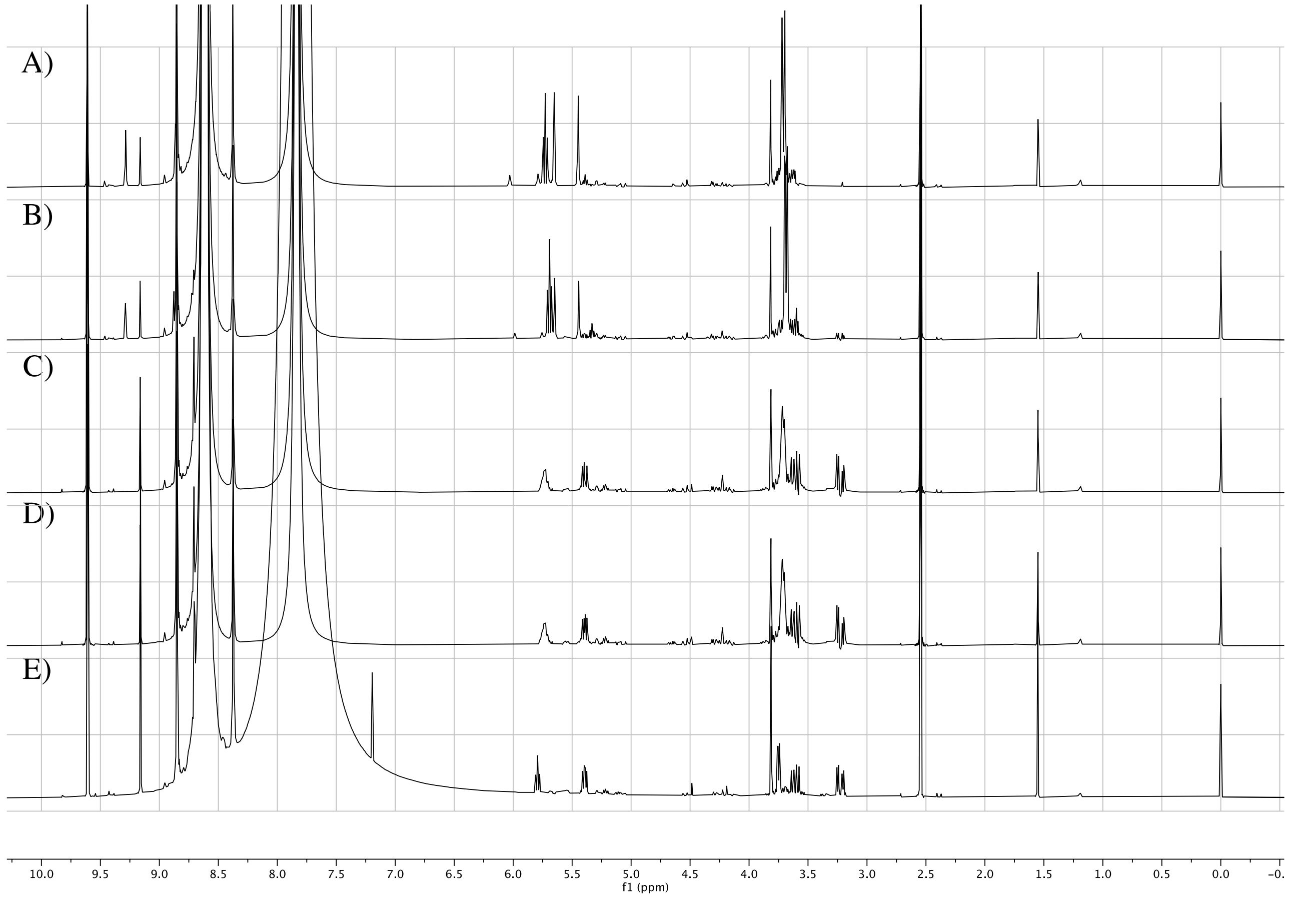

Fig S105. Stack of ^{1}H NMR spectra showing the reaction of FoDHA-CN with NaCN (3.0 eq.) in formamide at room temperature. A) 10 min; B) 2 h; C) 4 h; D) 12 h; E) 48 h.

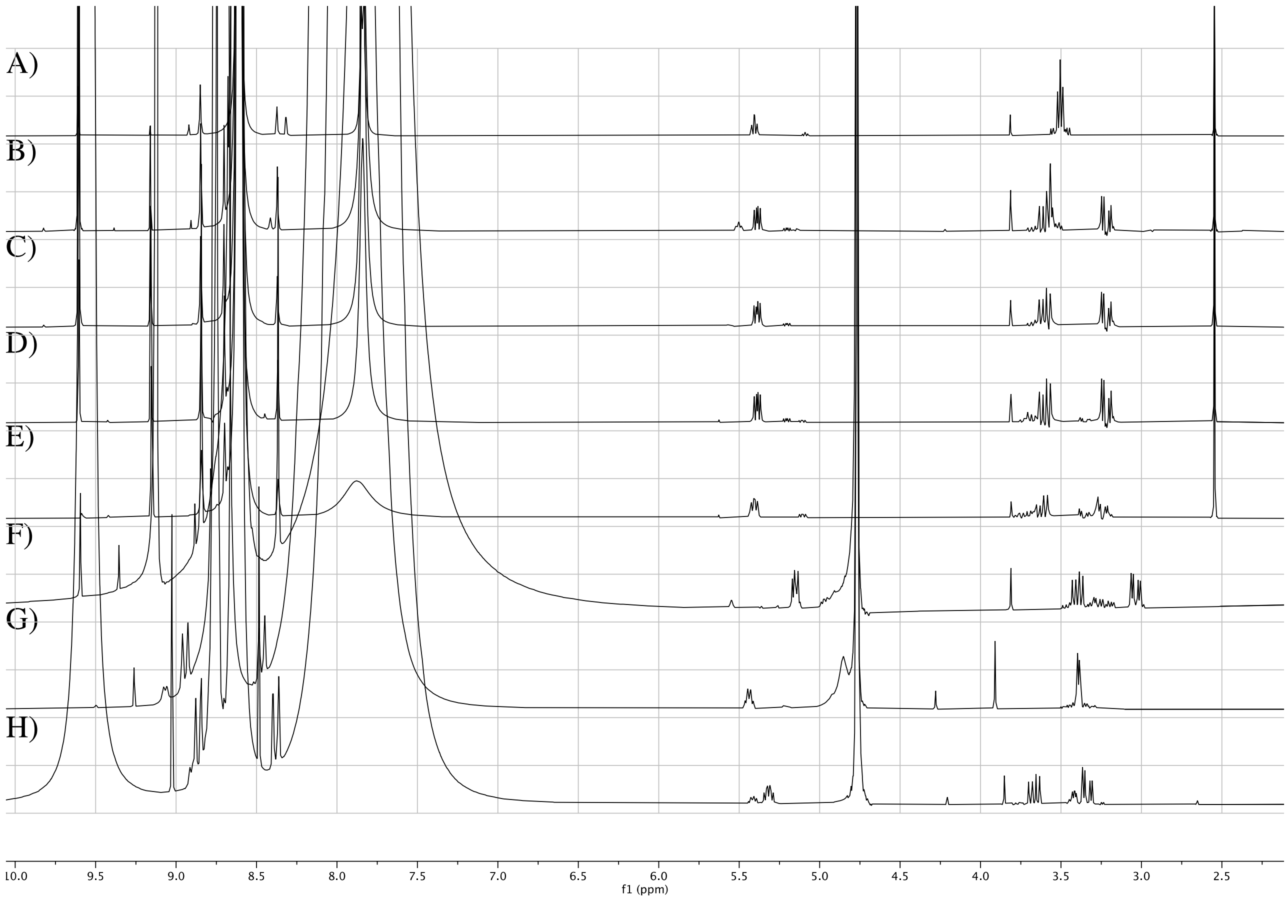

Fig S106. Stack of ^{1}H NMR spectra showing the reaction of Fo(β-CN)AlaCN with NaOH (5.0 eq.) in formamide at room temperature, forming $FoAsnNH_2$. A) 10 min; B) 16h; C) 32h; D) 3 days; E) 7 days; F) $FoAsnNH_2$ standard + NaOH; G) $FoAsnNH_2$; H) $FoAsnNH_2$ standard + NaOH, then + formic acid to partially neutralise.

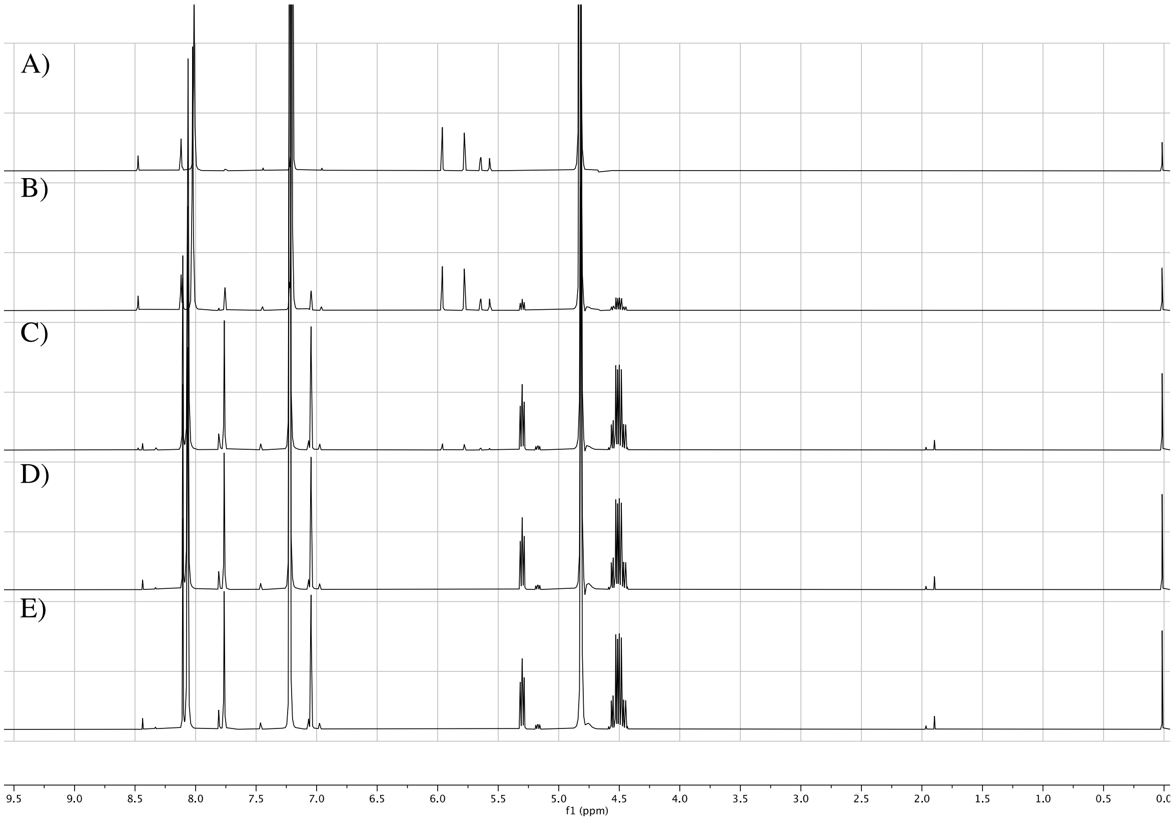

Fig S107. Stack of ^{1}H NMR spectra showing the reaction of FoDHA-CN with imidazole (5.0 eq.) in water, pH 7.5, at room temperature. A) 5 min; B) 16h; C) 48h; D) 96h; E) 6 days.

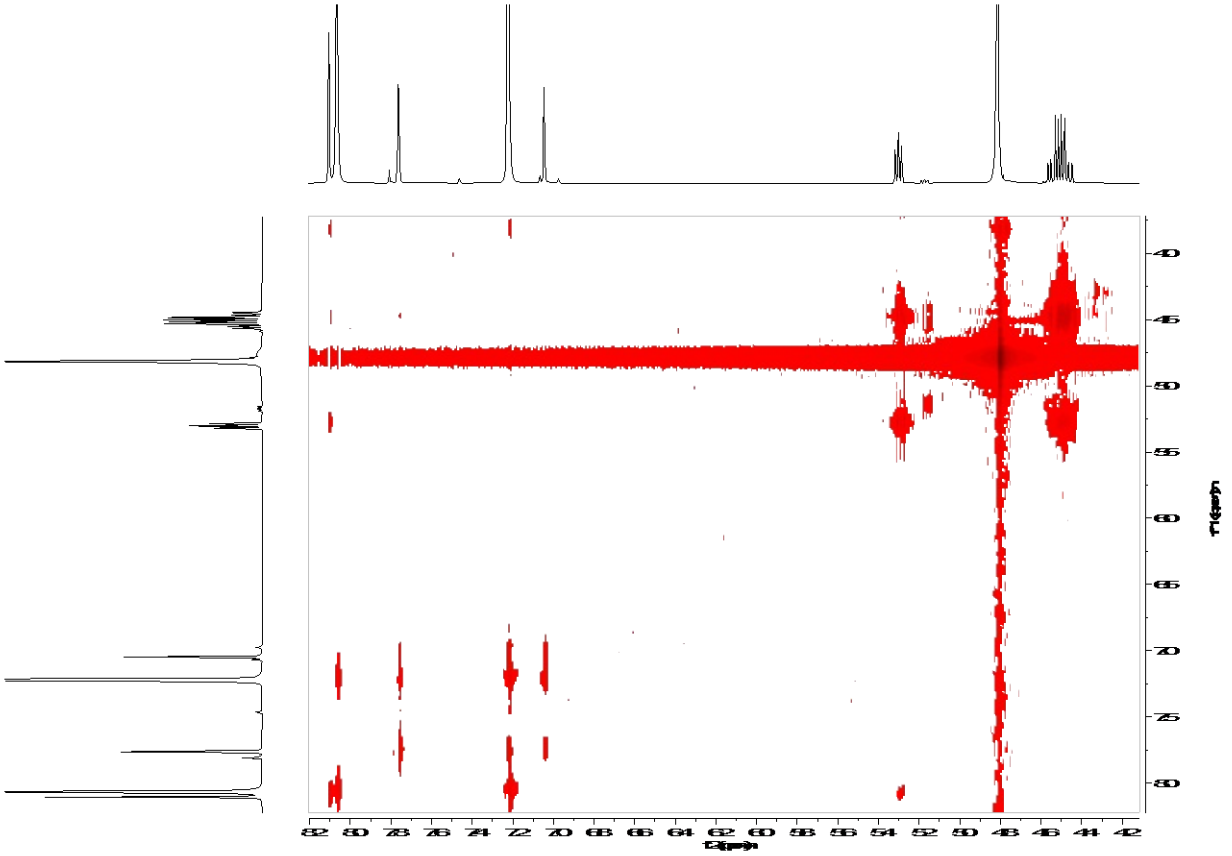

Fig S108. COSY spectrum of the reaction of FoDHA-CN with imidazole (5.0 eq.) in water, pH 7.5, at room temperature for 6 days.

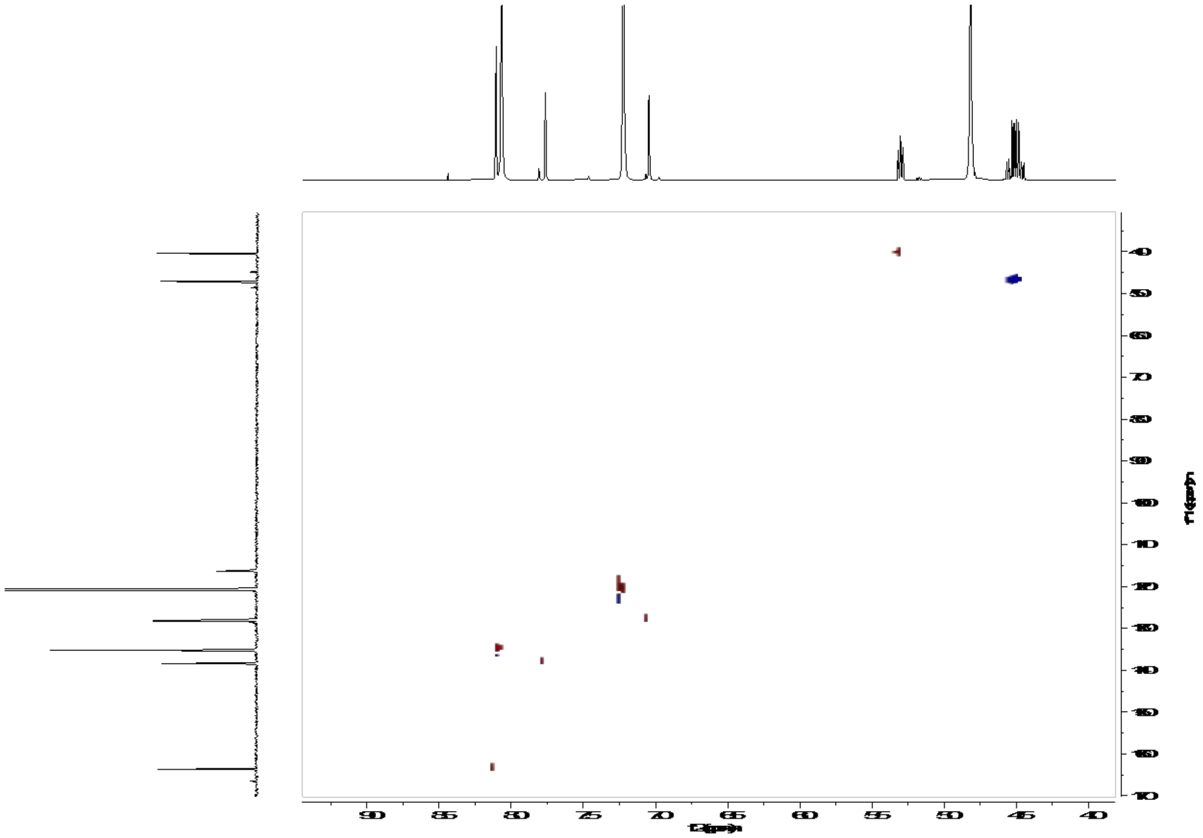

Fig S109. HSQC spectrum of the reaction of FoDHA-CN with imidazole (5.0 eq.) in water, pH 7.5, at room temperature for 6 days.

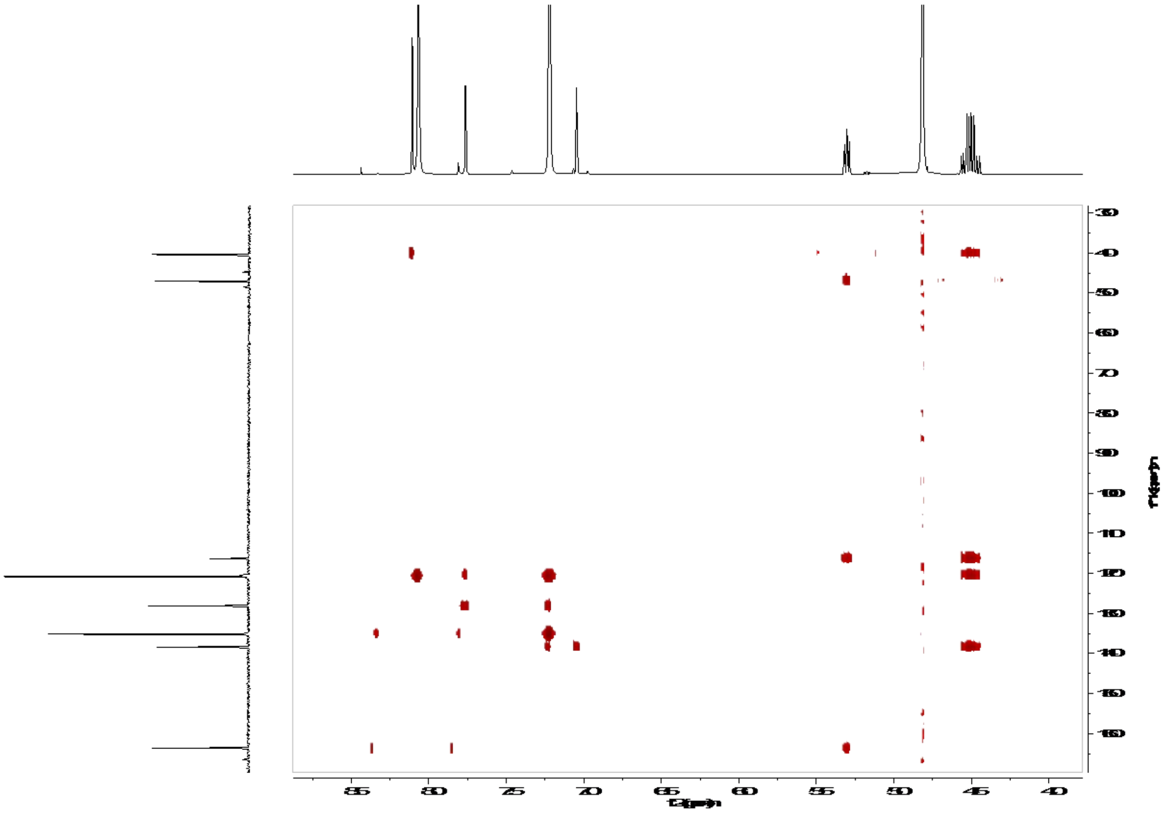

Fig S110. HMBC spectrum of the reaction of FoDHA-CN with imidazole (5.0 eq.) in water, pH 7.5, at room temperature for 6 days.

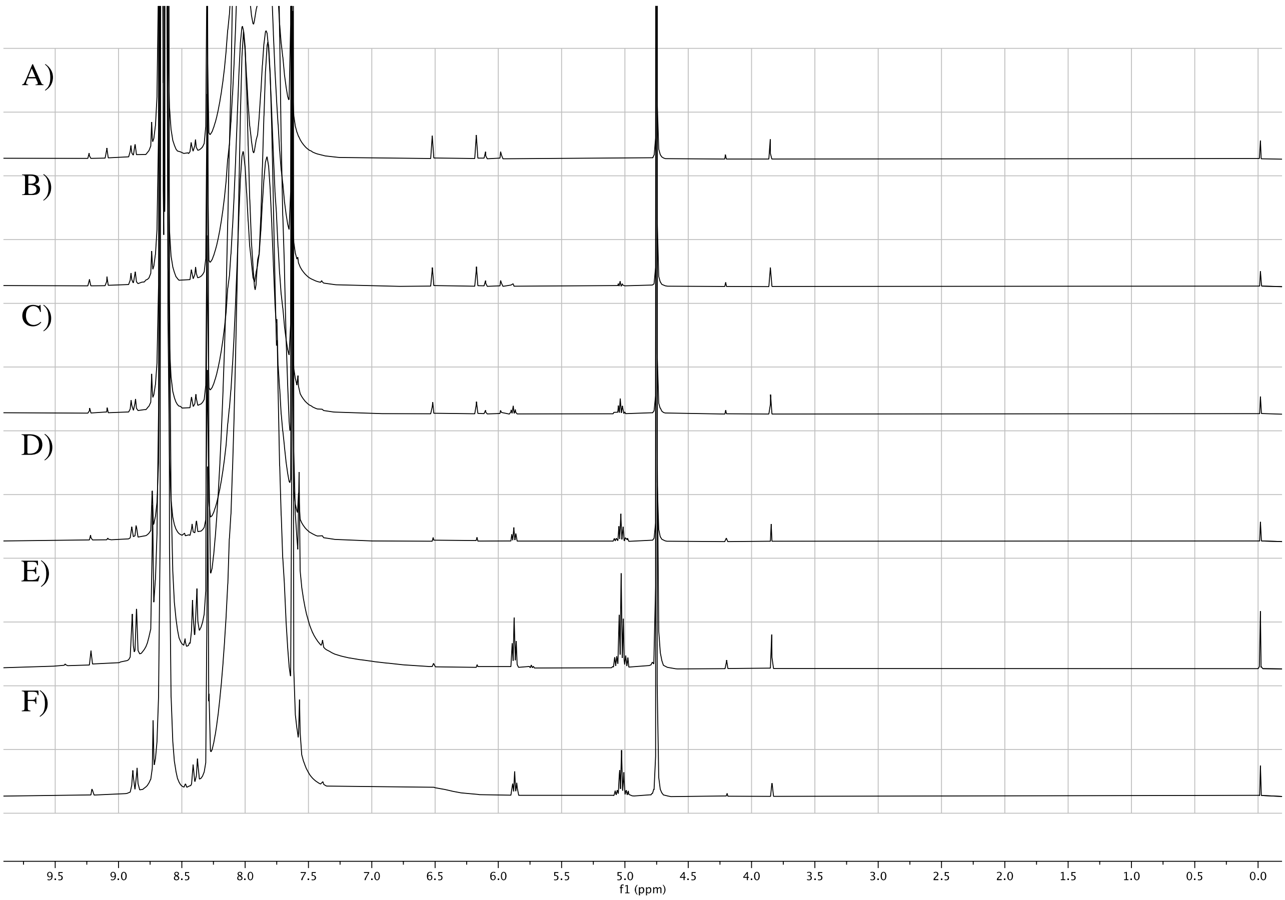

Fig S111. Stack of ^{1}H NMR spectra showing the reaction of FoDHA-CN with imidazole (5.0 eq.) in formamide at room temperature. A) 5 min; B) 4h; C) 16h; D) 48h; E) 96h; F) 7 days.

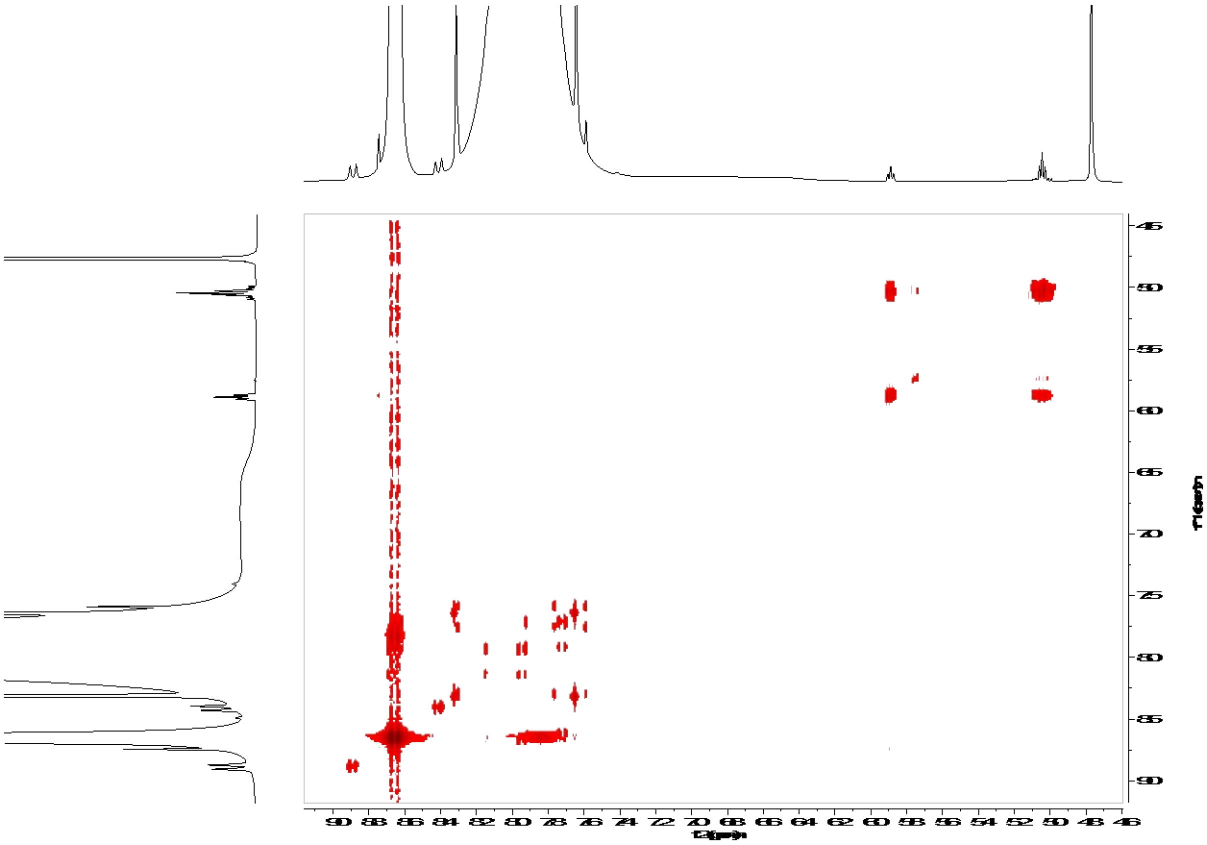

Fig S112. COSY spectrum of the reaction between imidazole and FoDHA-CN after 7 days.

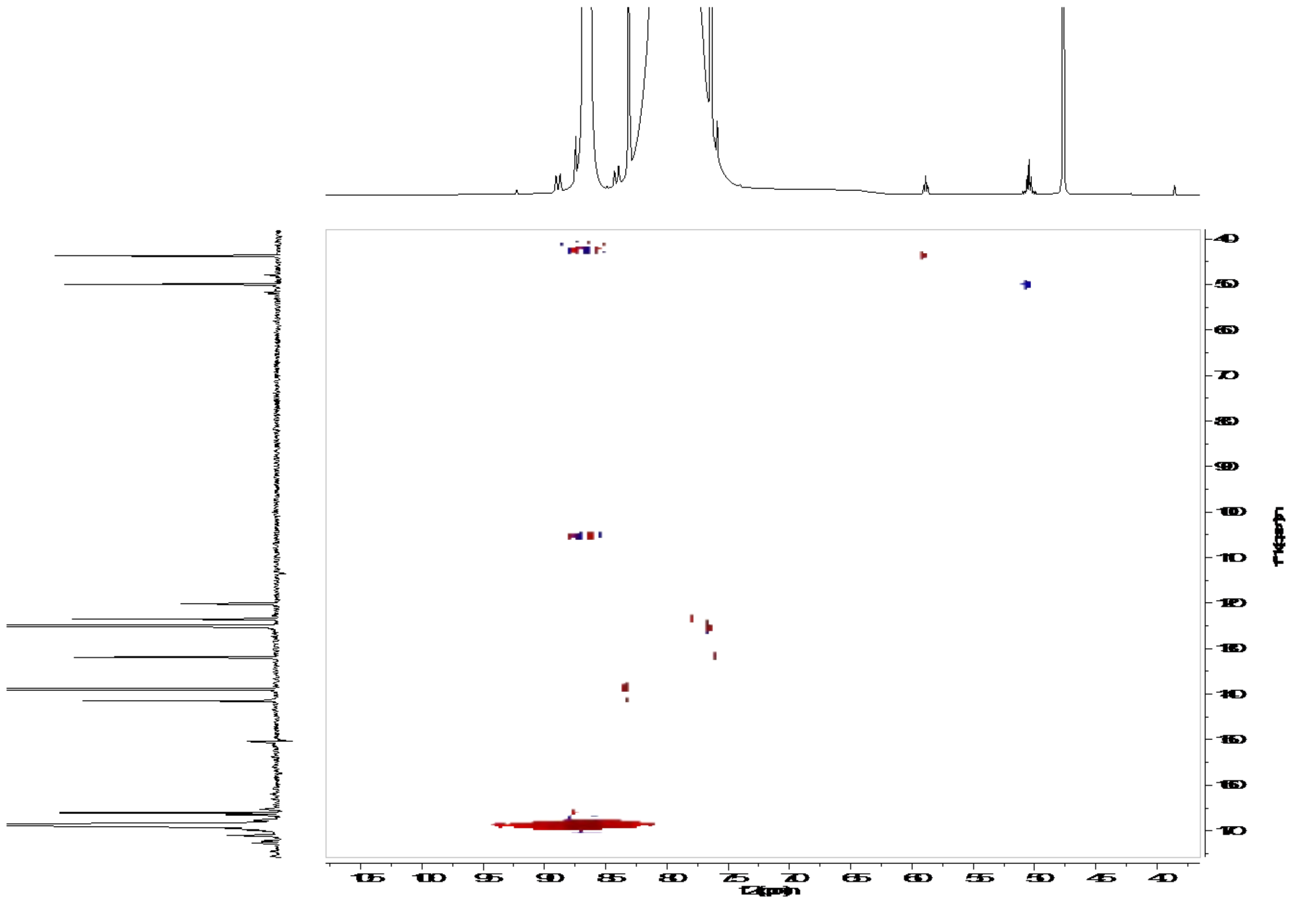

Fig S113. HSQC of the reaction between imidazole and FoDHA-CN after 7 days.

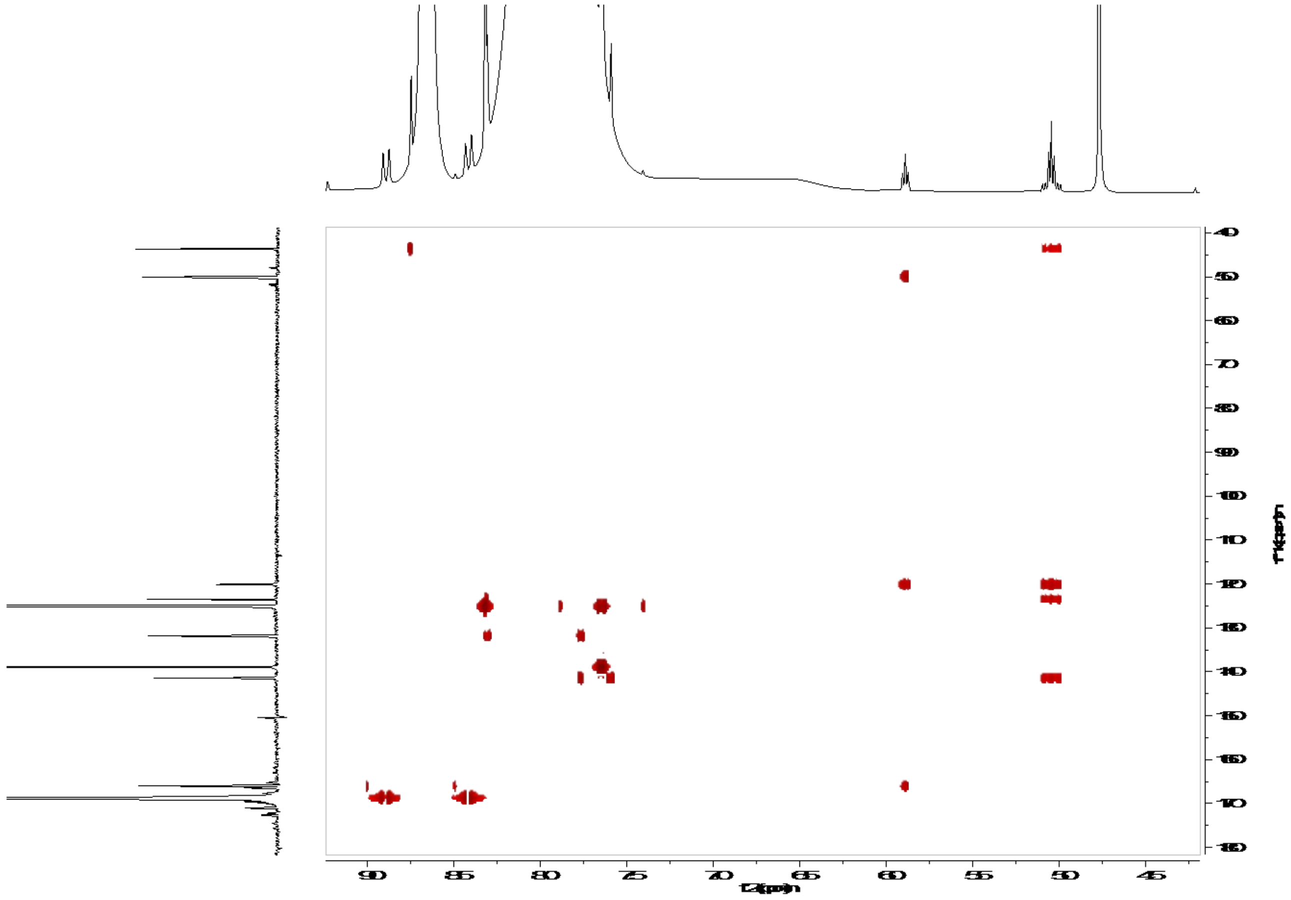

Fig S114. HMBC spectrum of the reaction between imidazole and FoDHA-CN after 7 days.

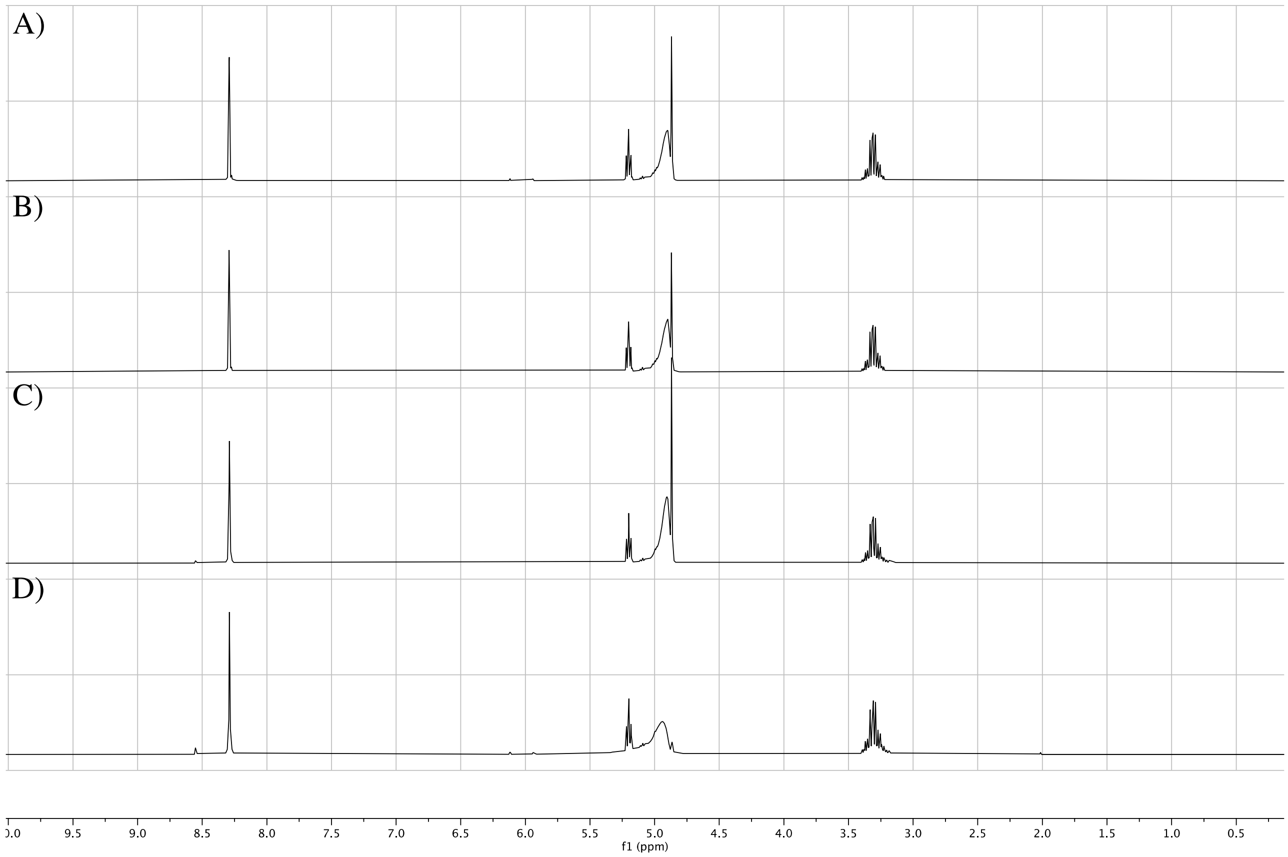

Fig S115. Stack of ^{1}H NMR spectra showing the reaction between FoDHA-CN and $NaSH.H_2O$ (1.5 eq.), pH 7. A) 30 min; B) 4h; C) 8h; D) 3 days.

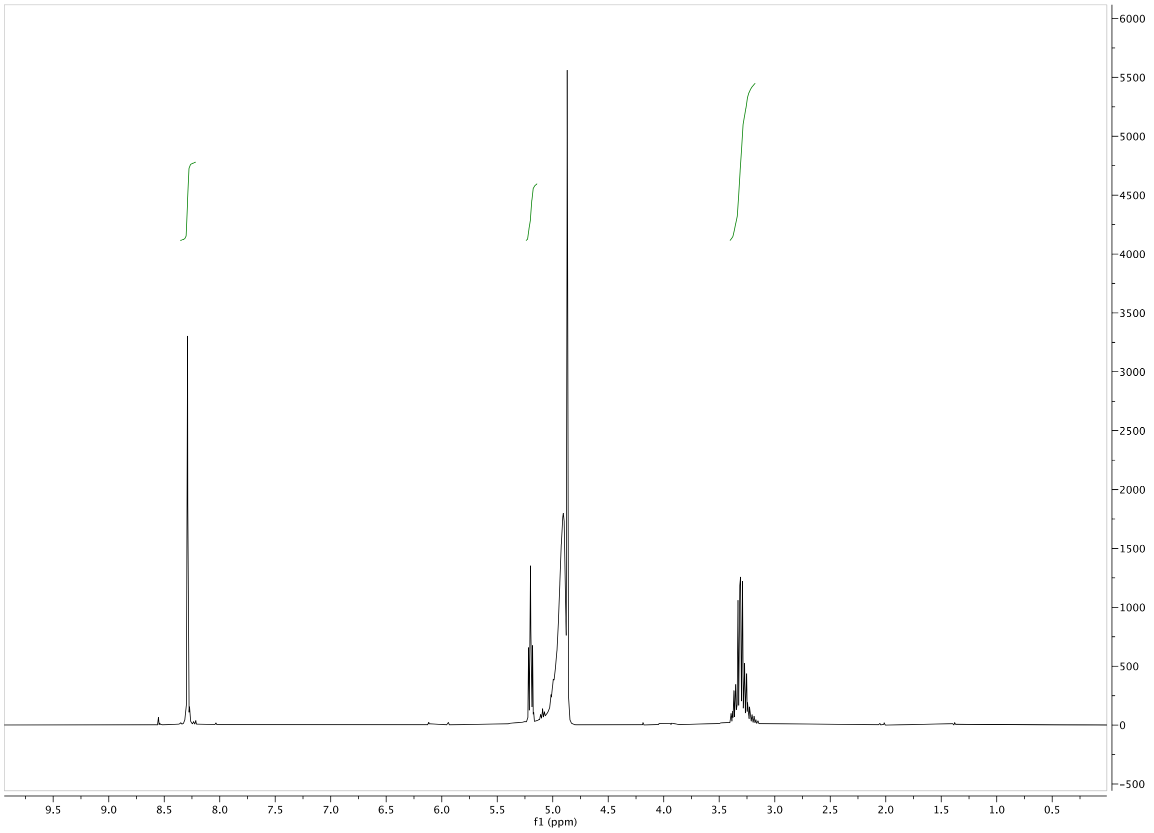

Fig S116. ^{1}H NMR spectrum of the reaction between FoDHA-CN and NaSH.H_2O (1.5 eq.), pH 7, 8h.

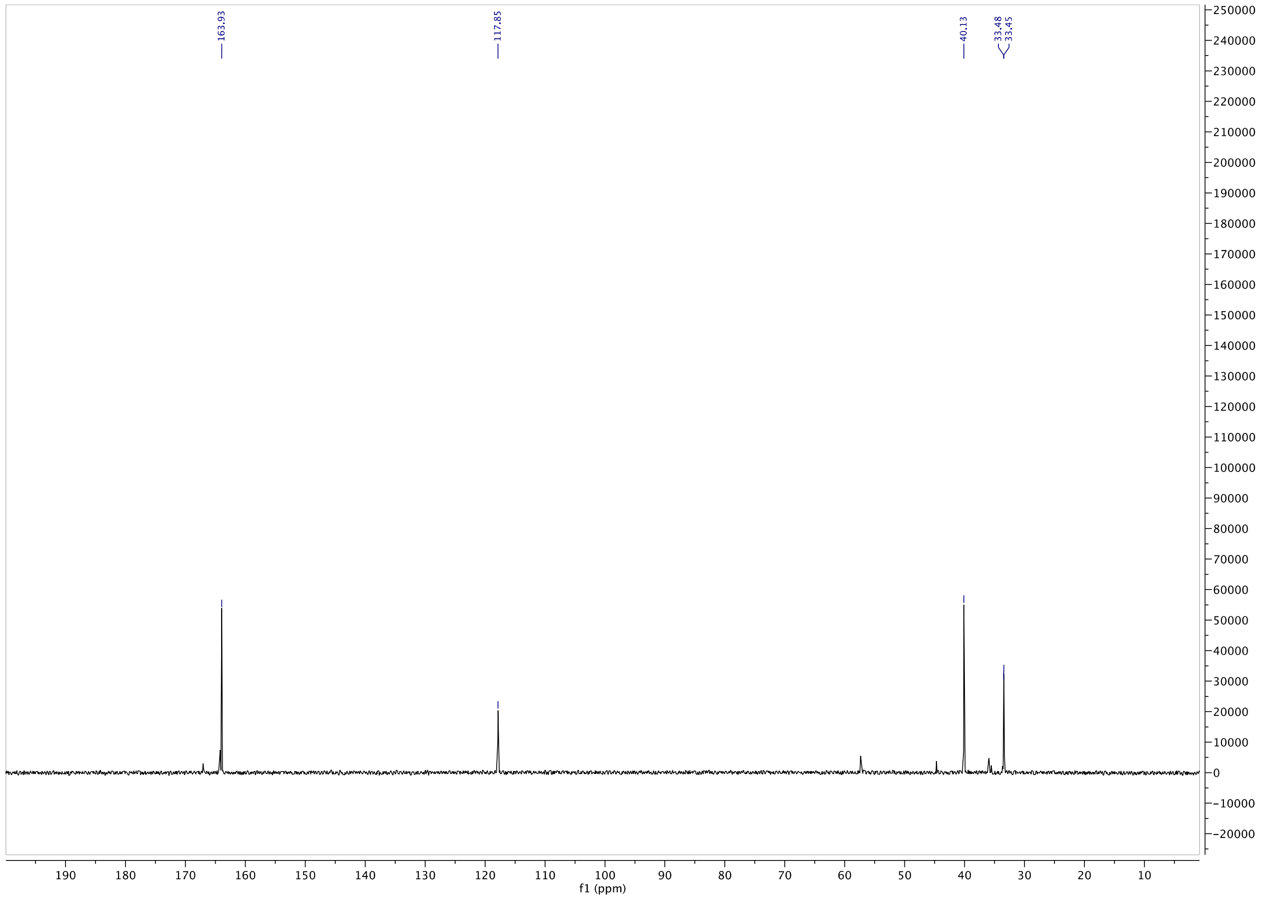

Fig S117. ^{13}C NMR spectrum of the reaction between FoDHA-CN and NaSH.H_2O (1.5 eq.), pH 7, 8h.

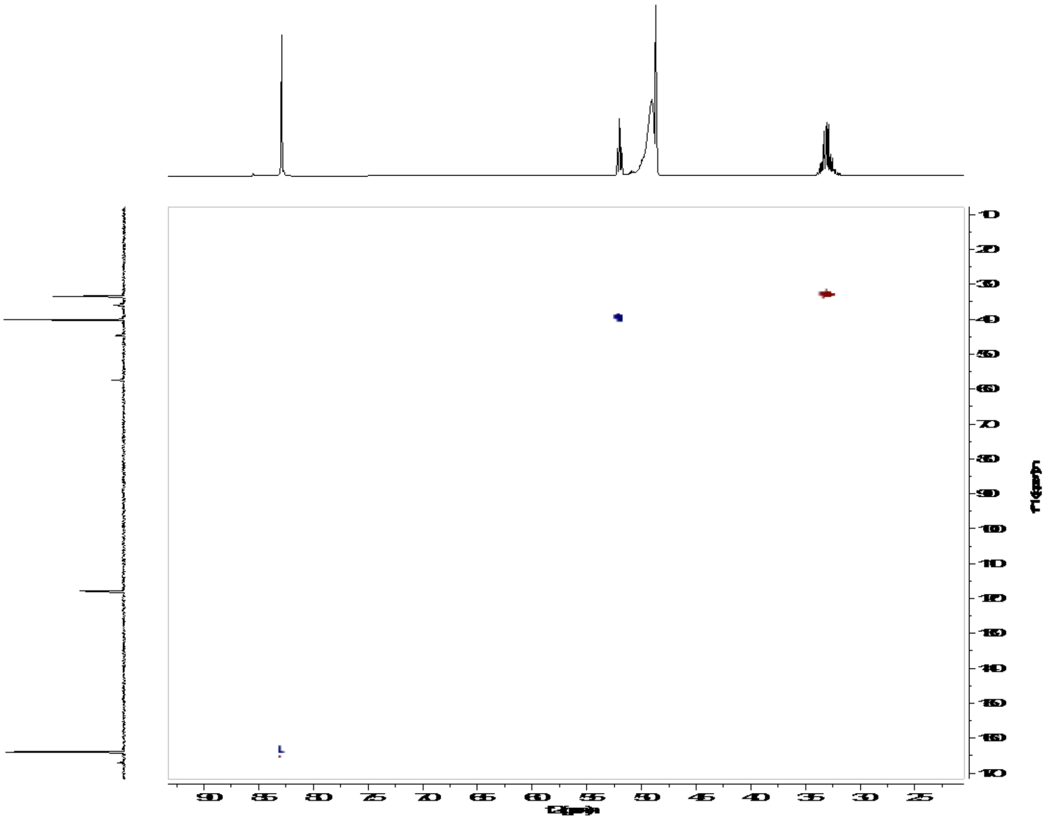

Fig S118. HSQC spectrum of the reaction between FoDHA-CN and NaSH.H_2O (1.5 eq.), pH 7, 8h.

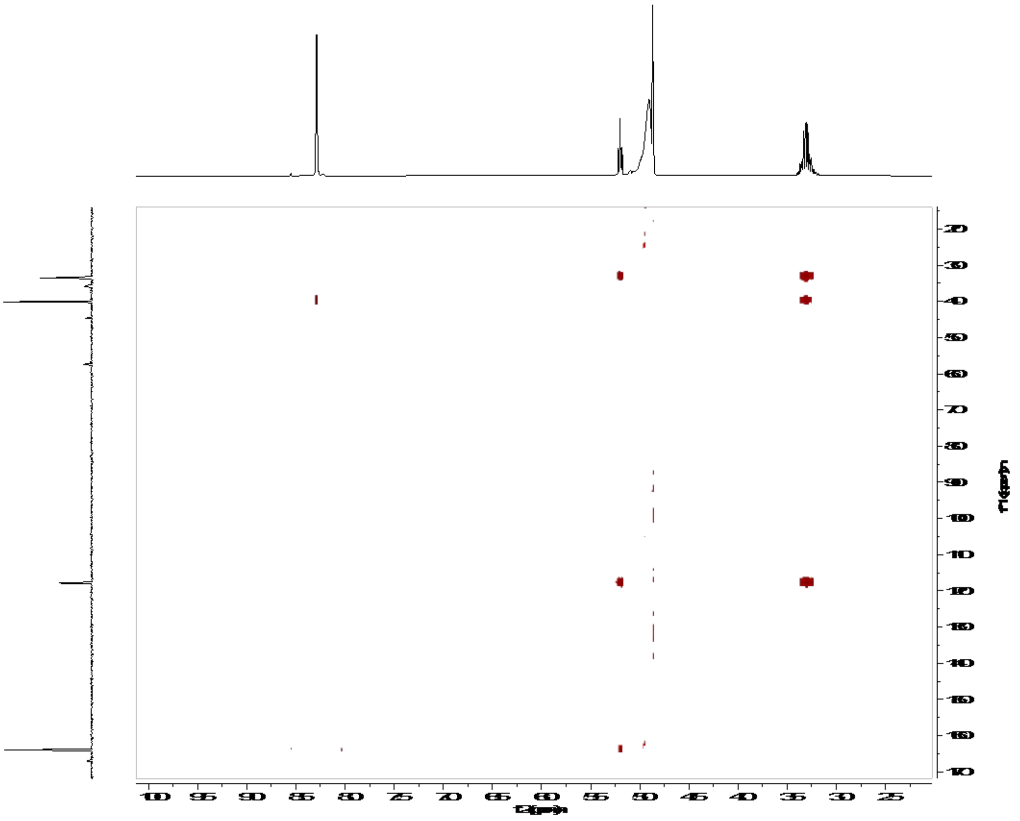

Fig S119. HMBC spectrum of the reaction between FoDHA-CN and NaSH.H_2O (1.5 eq.), pH 7, 8h.

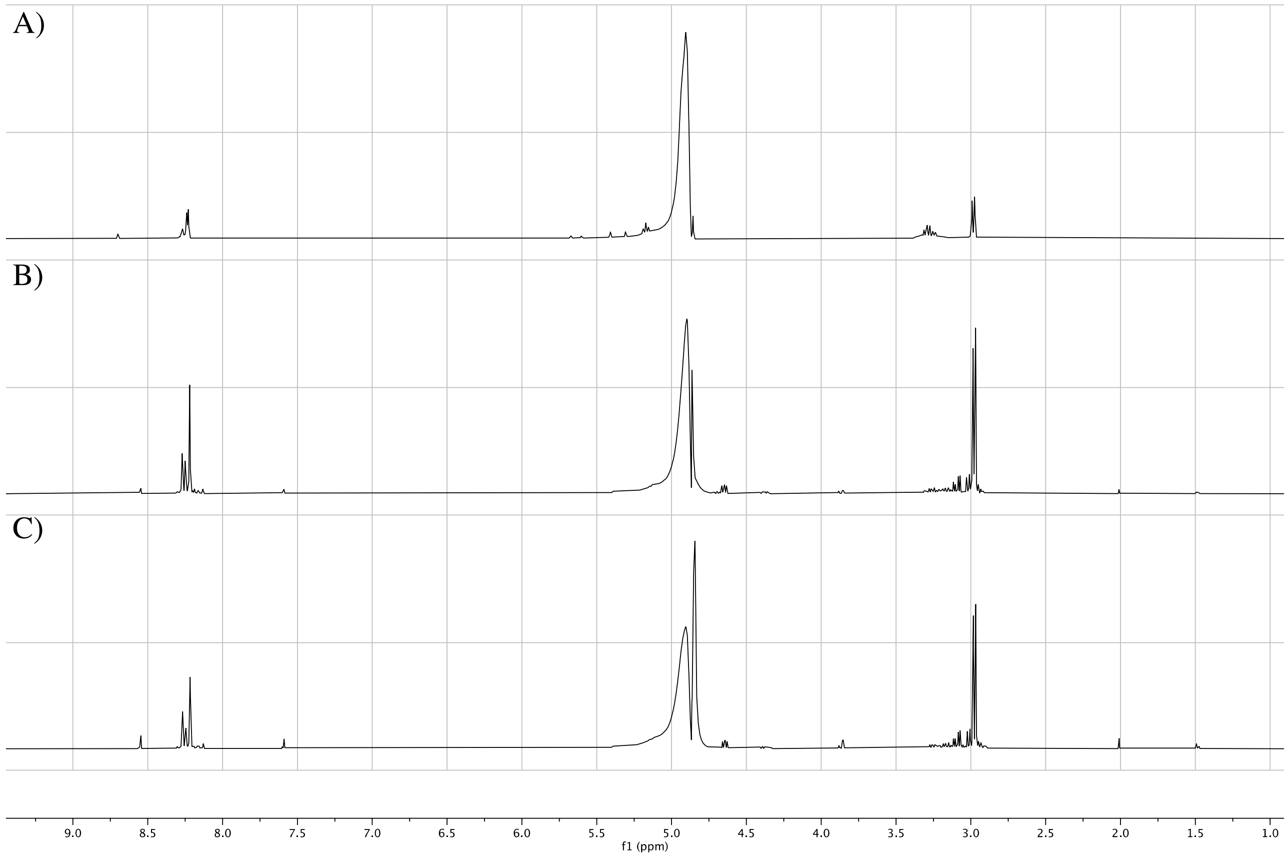

Fig S120. Stack of ^{1}H NMR spectra showing the reaction of FoDHA-CN with NaSH.H_2O (1.5 eq.) in water, pH 9, at room temperature. A) 20 min; B) 2h; C) 6h.

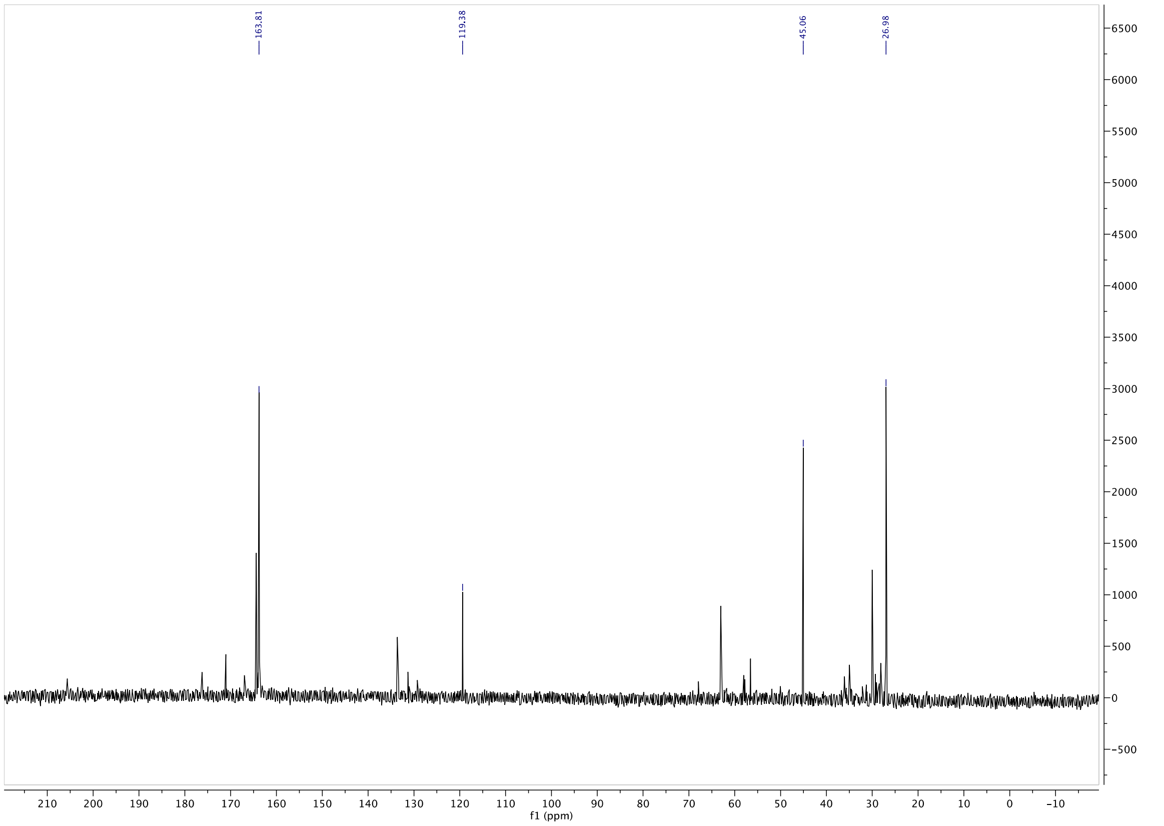

Fig S121. ^{13}C NMR spectrum of the reaction of FoDHA-CN with NaSH.H_2O (1.5 eq.) in water, pH 9, at room temperature, 14h.

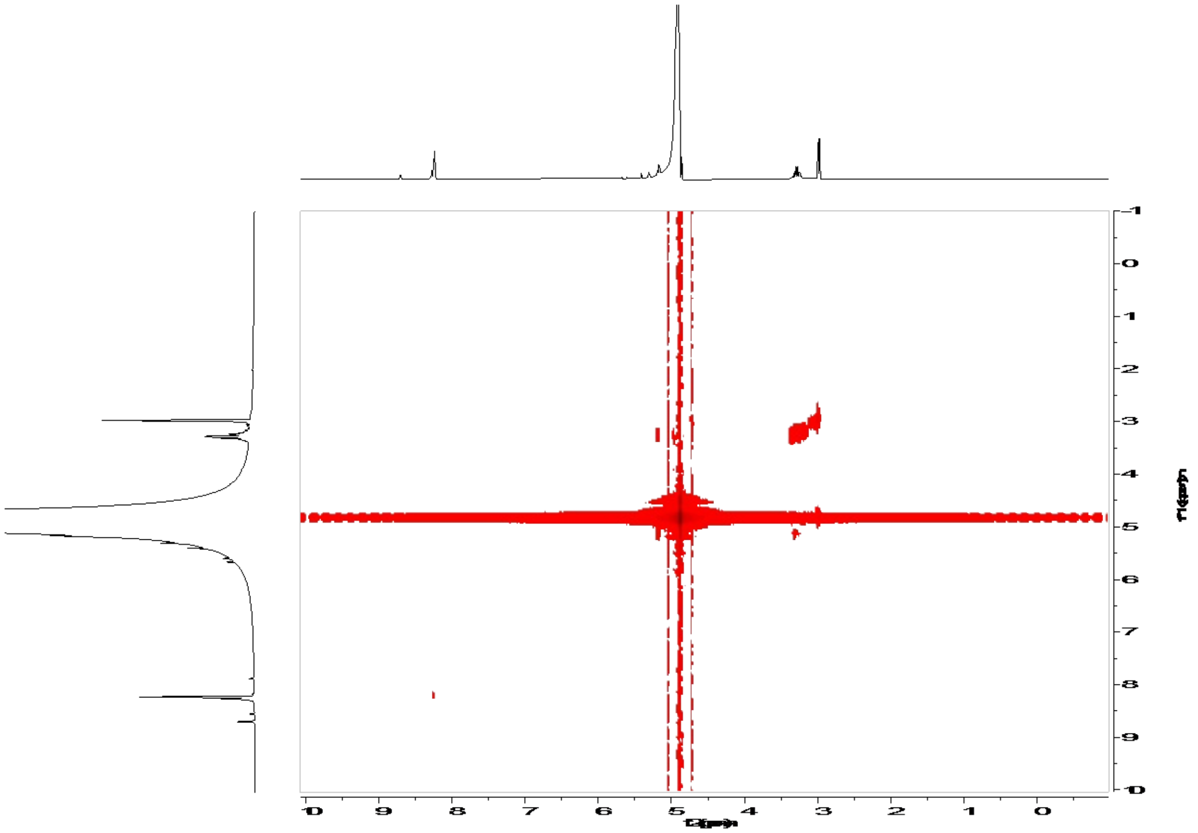

Fig S122. COSY spectrum of the reaction of FoDHA-CN with NaSH.H_2O (1.5 eq.) in water, pH 9, at room temperature, 20 min.

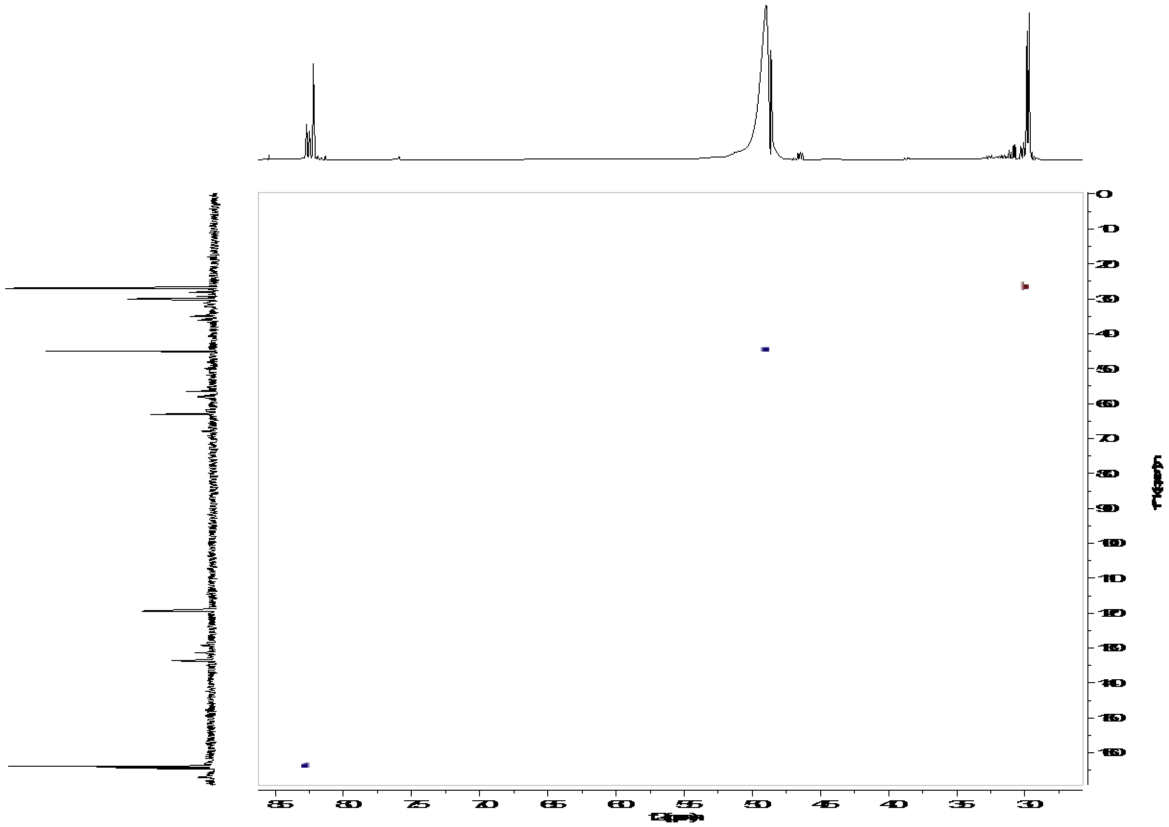

Fig S123. HSQC spectrum of the reaction of FoDHA-CN with NaSH.H_2O (1.5 eq.) in water, pH 9, at room temperature, 20 min.

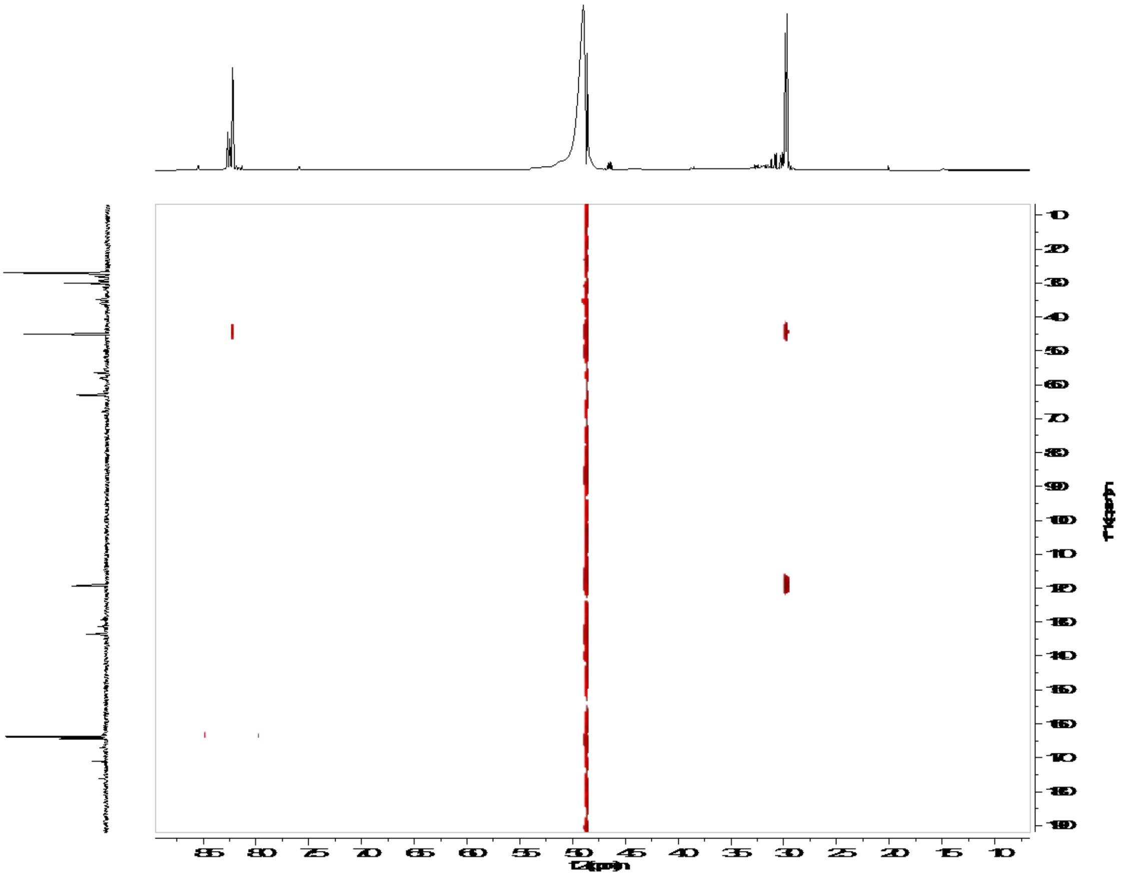

Fig S124. HMBC spectrum of the reaction of FoDHA-CN with $NaSH.H_2O$ (1.5 eq.) in water, pH 9, at room temperature, 20 min.

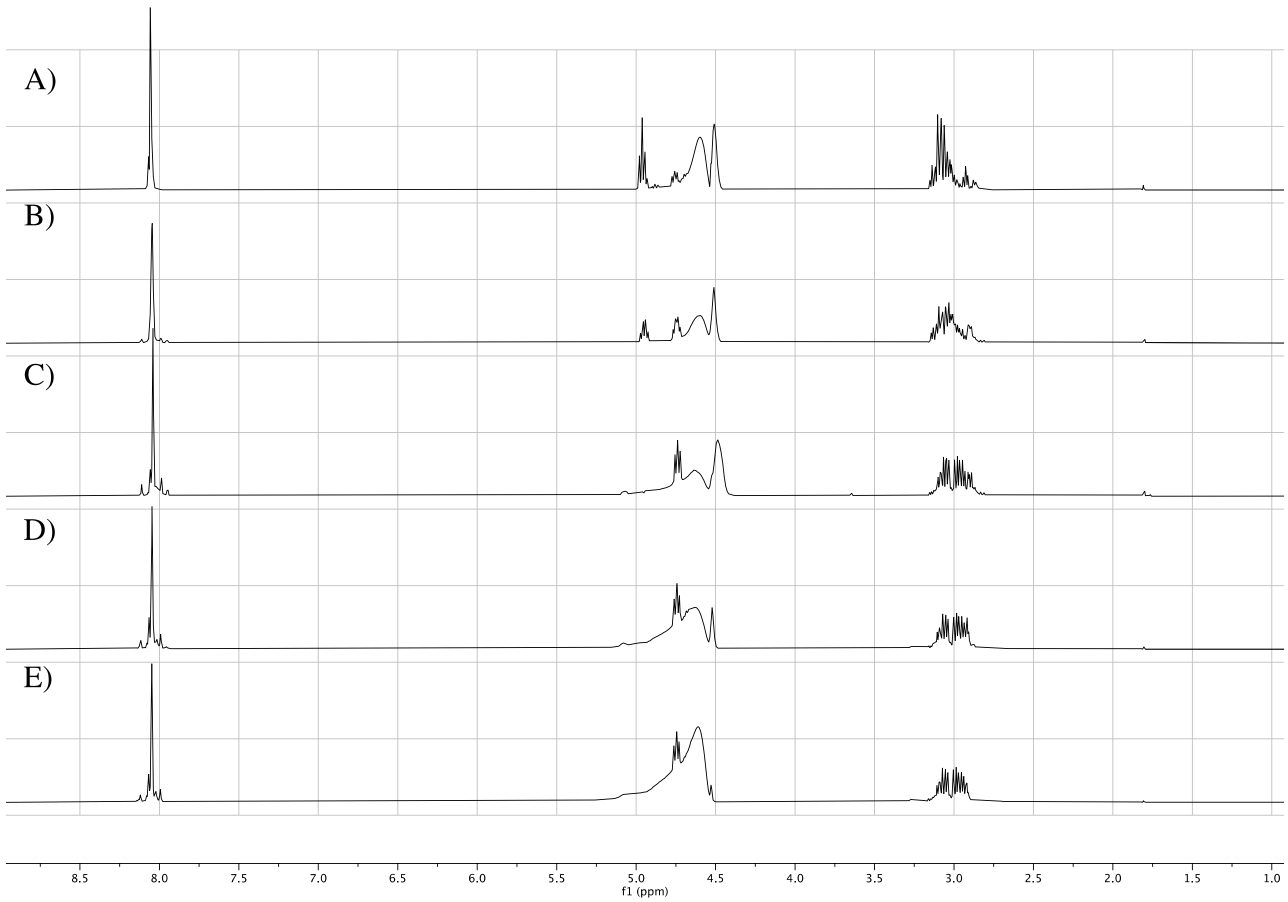

Fig S125. Stack of ^{1}H NMR spectra showing the reaction between FoDHA-CN and NaSH.H_2O (10 eq.), pH 7. A) 5 min; B) 30 min; C) 4h; D) 16h; E) 2 days.

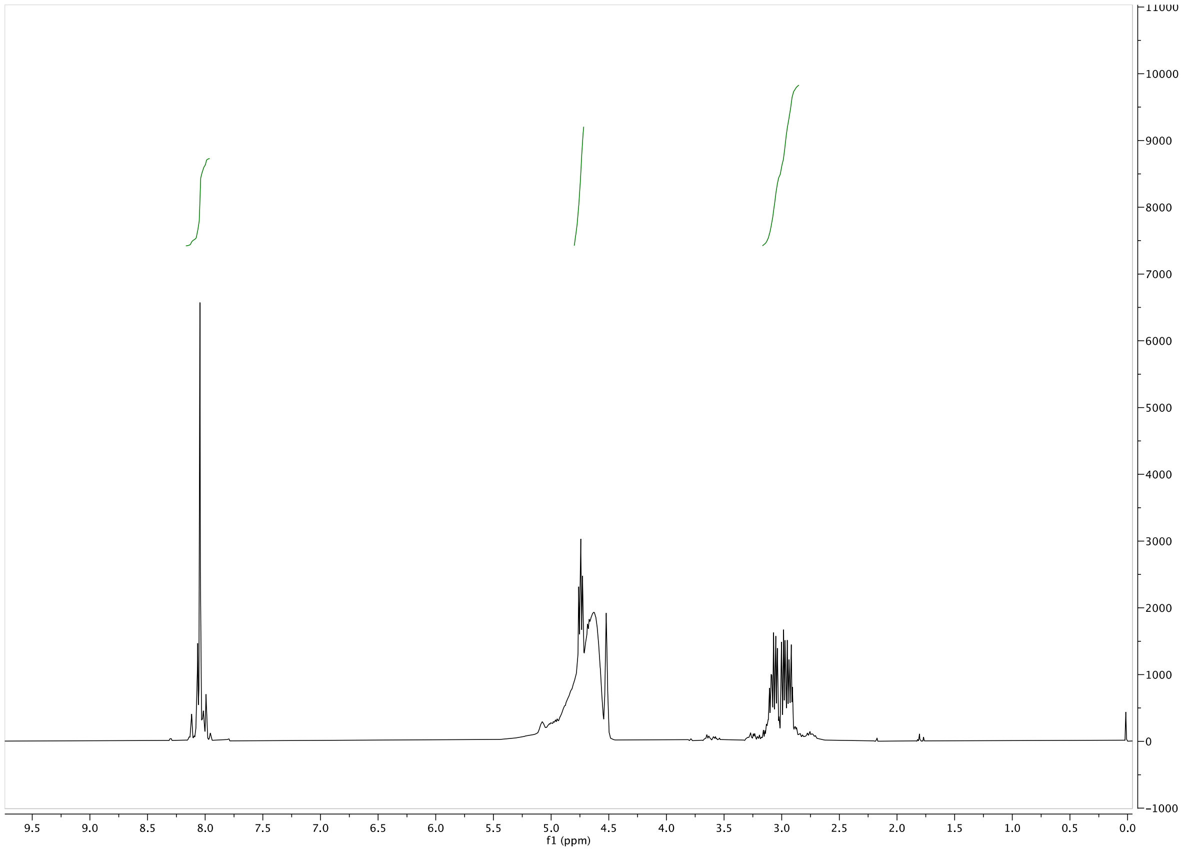

Fig S126. ^{1}H NMR spectrum of reaction between FoDHA-CN and $NaSH.H_2O$ (10 eq.), pH 7 after 2 days.

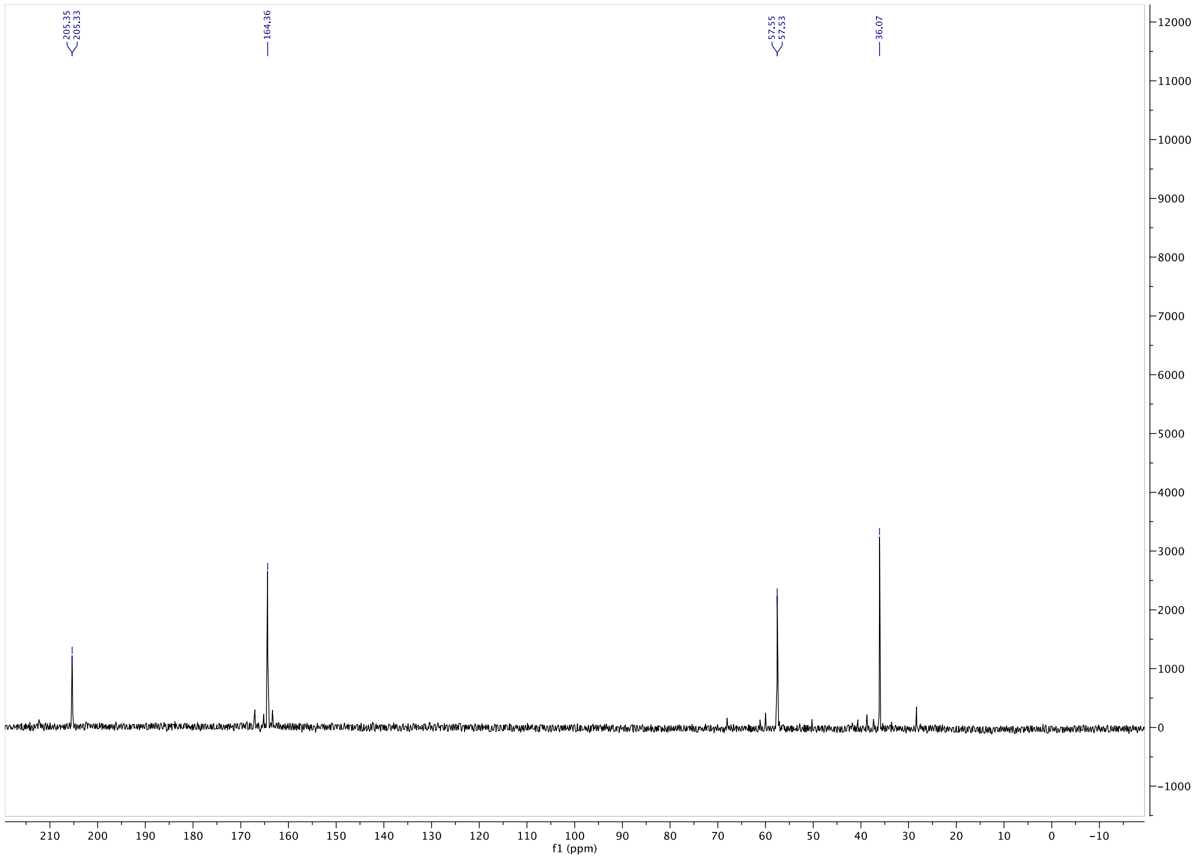

Fig S127. ^{13}C NMR spectrum of reaction between FoDHA-CN and $NaSH.H_2O$ (10 eq.), pH 7 after 2 days.

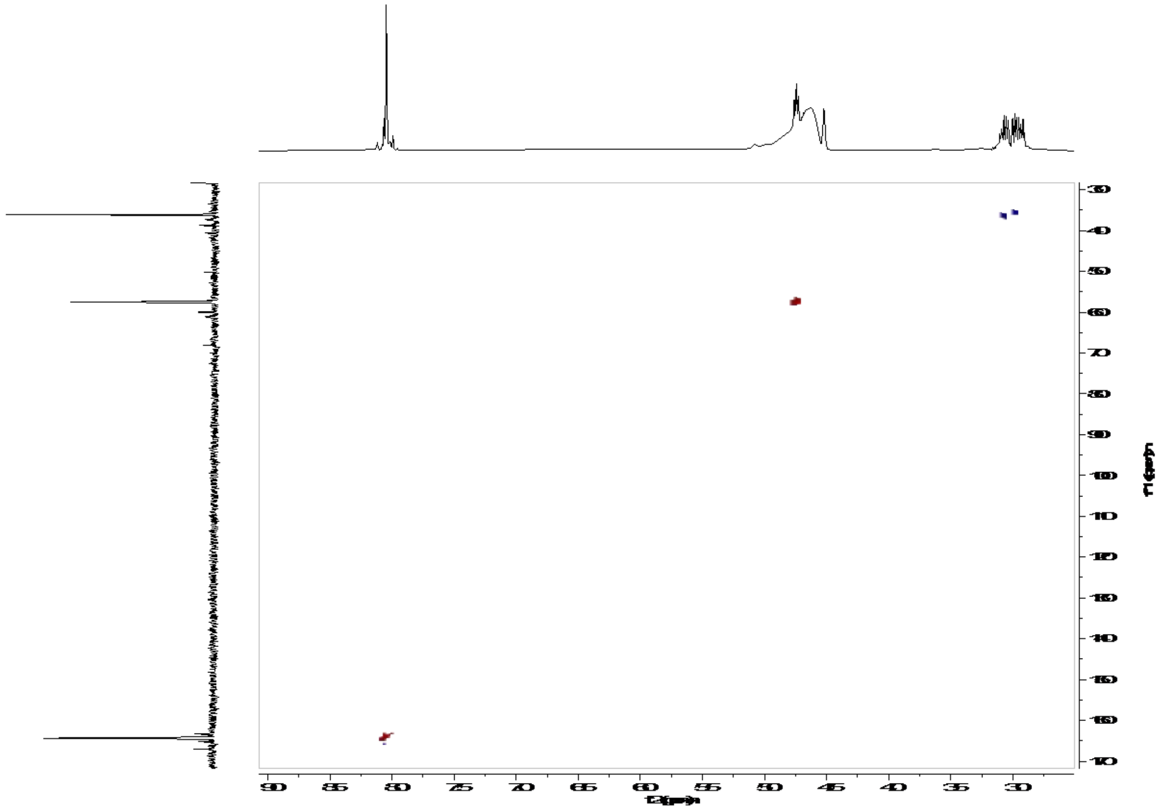

Fig S128. HSQC spectrum of reaction between FoDHA-CN and NaSH.H_2O (10 eq.), pH 7 after 2 days.

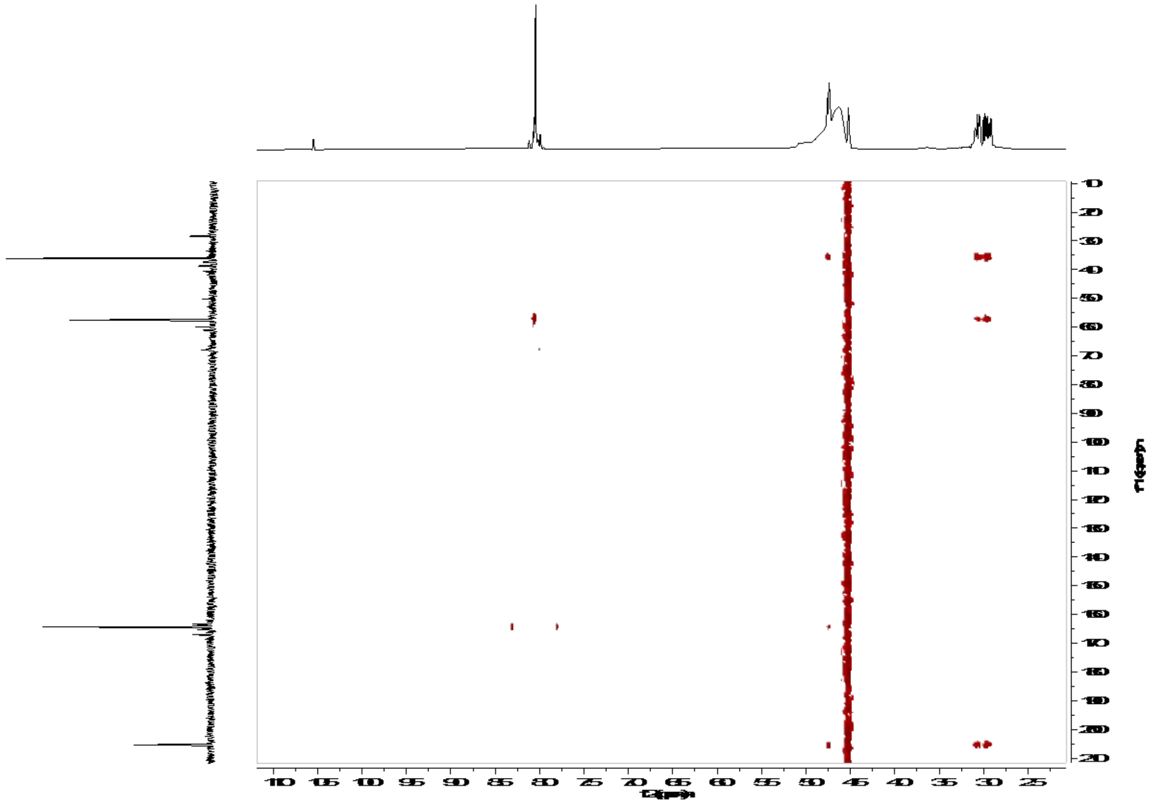

Fig S129. HMBC spectrum of reaction between FoDHA-CN and NaSH.H_2O (10 eq.), pH 7 after 2 days.

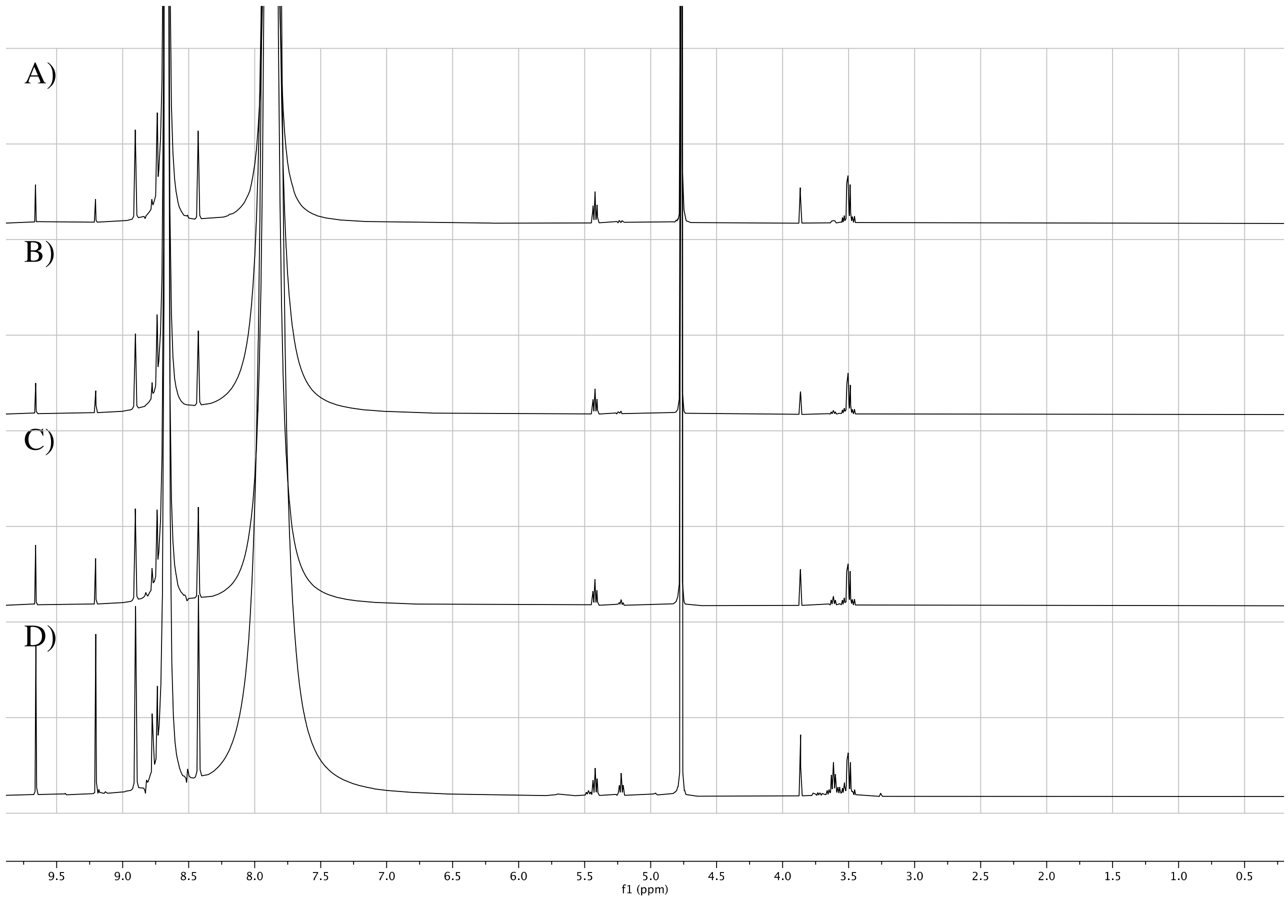

Fig S130. Stack of ^{1}H NMR spectra showing the reaction between FoDHA-CN and NaSH.H_2O (3.0 eq.) in formamide, room temperature A) 6h; B) 16h; C) 24h; D) 36h.

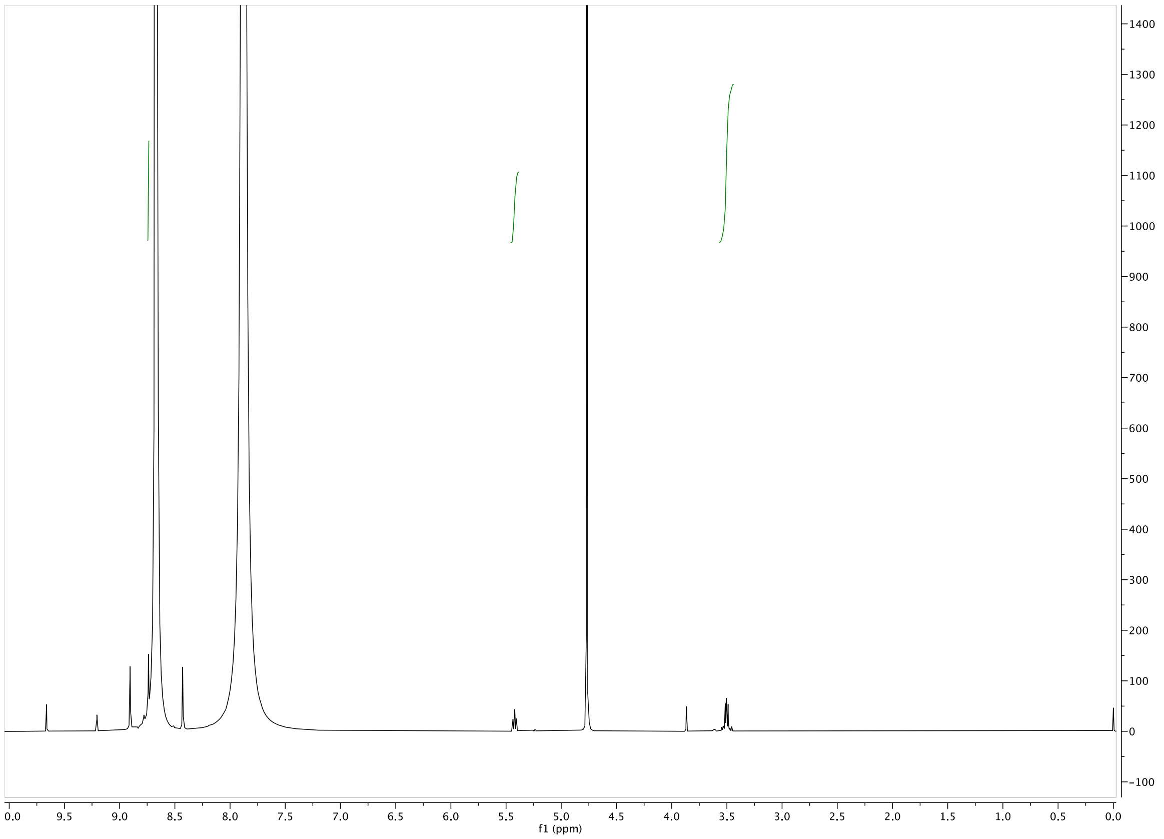

Fig S131. ^{1}H NMR spectrum of the reaction between FoDHA-CN and $NaSH.H_2O$ (3.0 eq.) in formamide, room temperature, 6h.

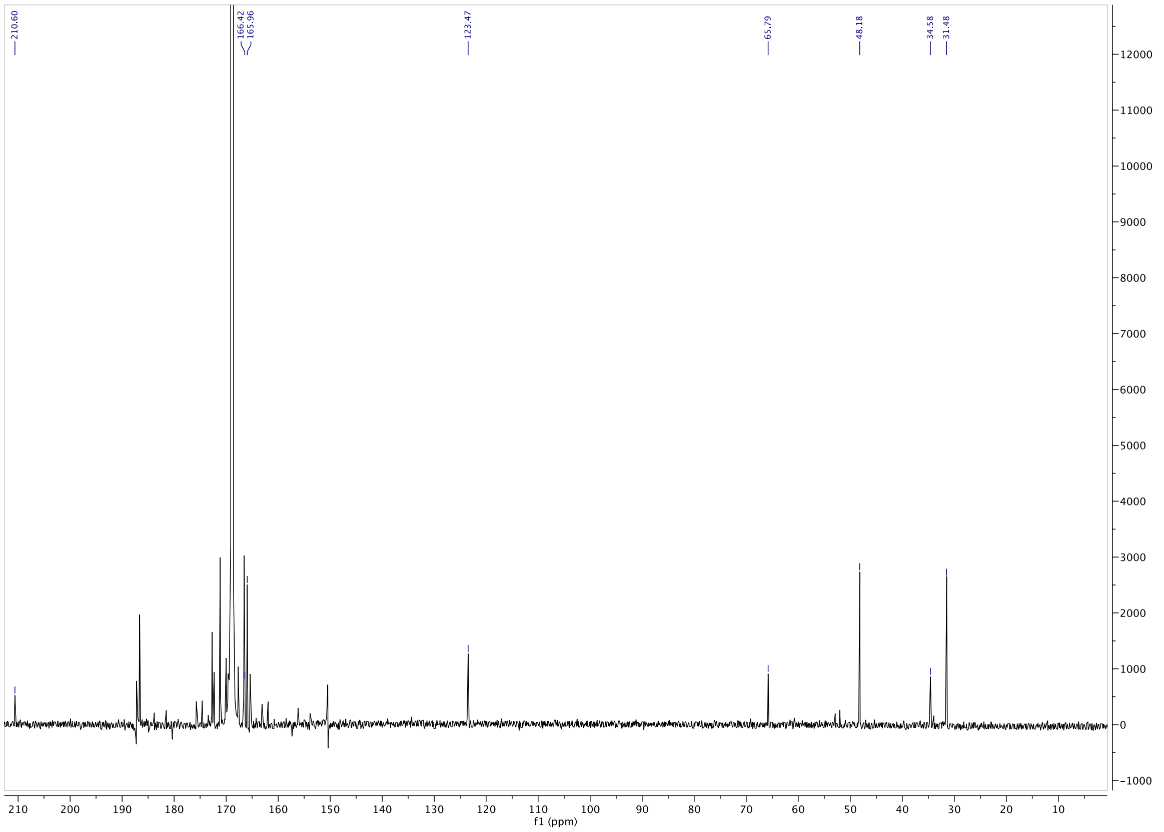

Fig S132. ^{13}C NMR spectrum of the reaction between FoDHA-CN and $NaSH.H_2O$ (3.0 eq.) in formamide, room temperature, 36h, showing FoCysCN and FoCysC(S)NH_2.

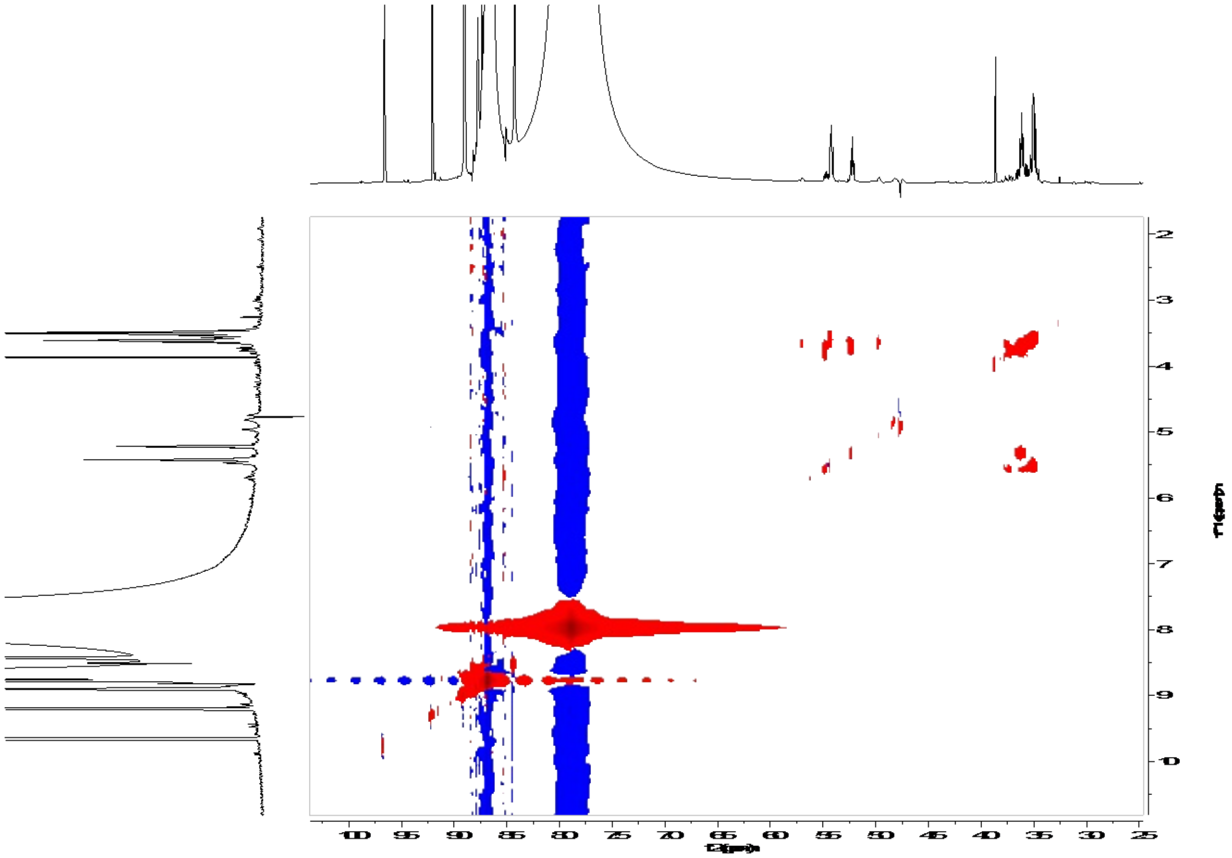

Fig S133. COSY spectrum of the reaction between FoDHA-CN and $NaSH.H_2O$ (3.0 eq.) in formamide, room temperature, 36h.

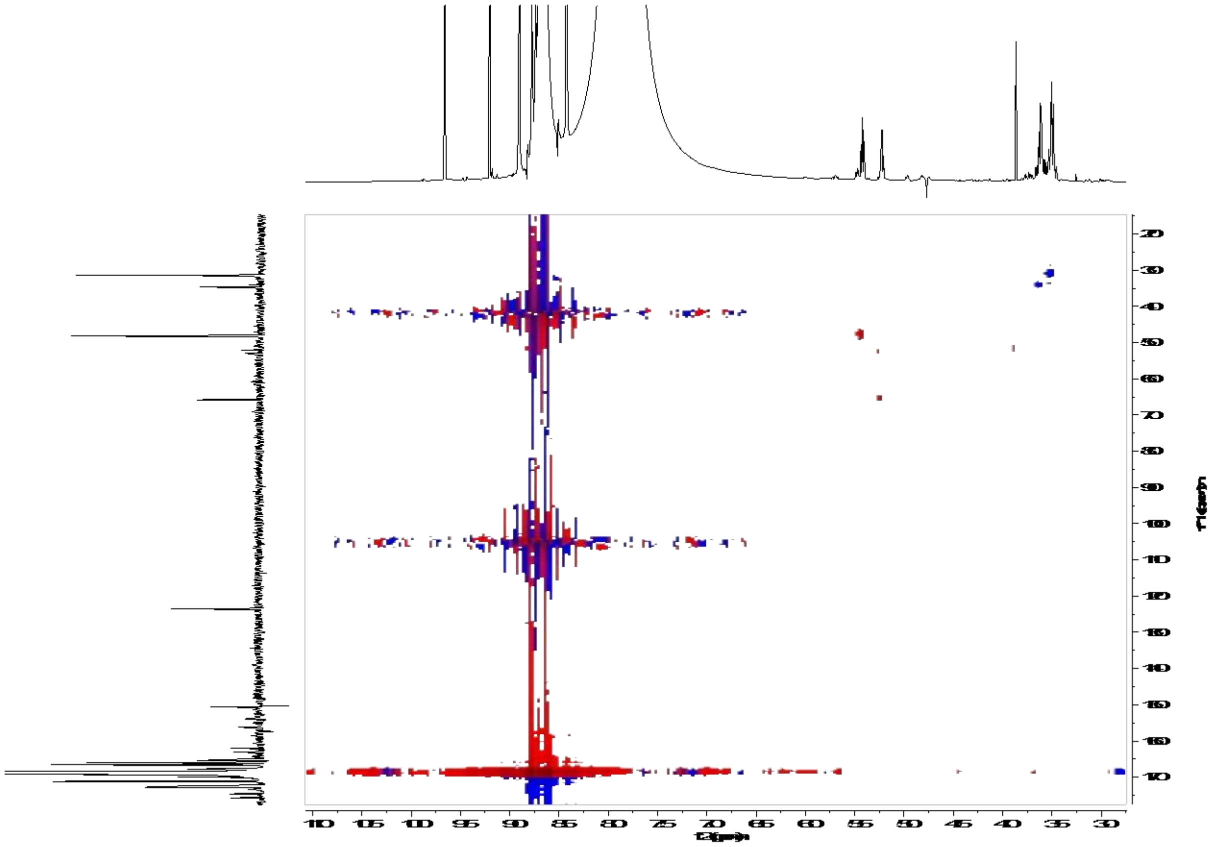

Fig S134. HSQC spectrum of the reaction between FoDHA-CN and $NaSH.H_2O$ (3.0 eq.) in formamide, room temperature, 36h.

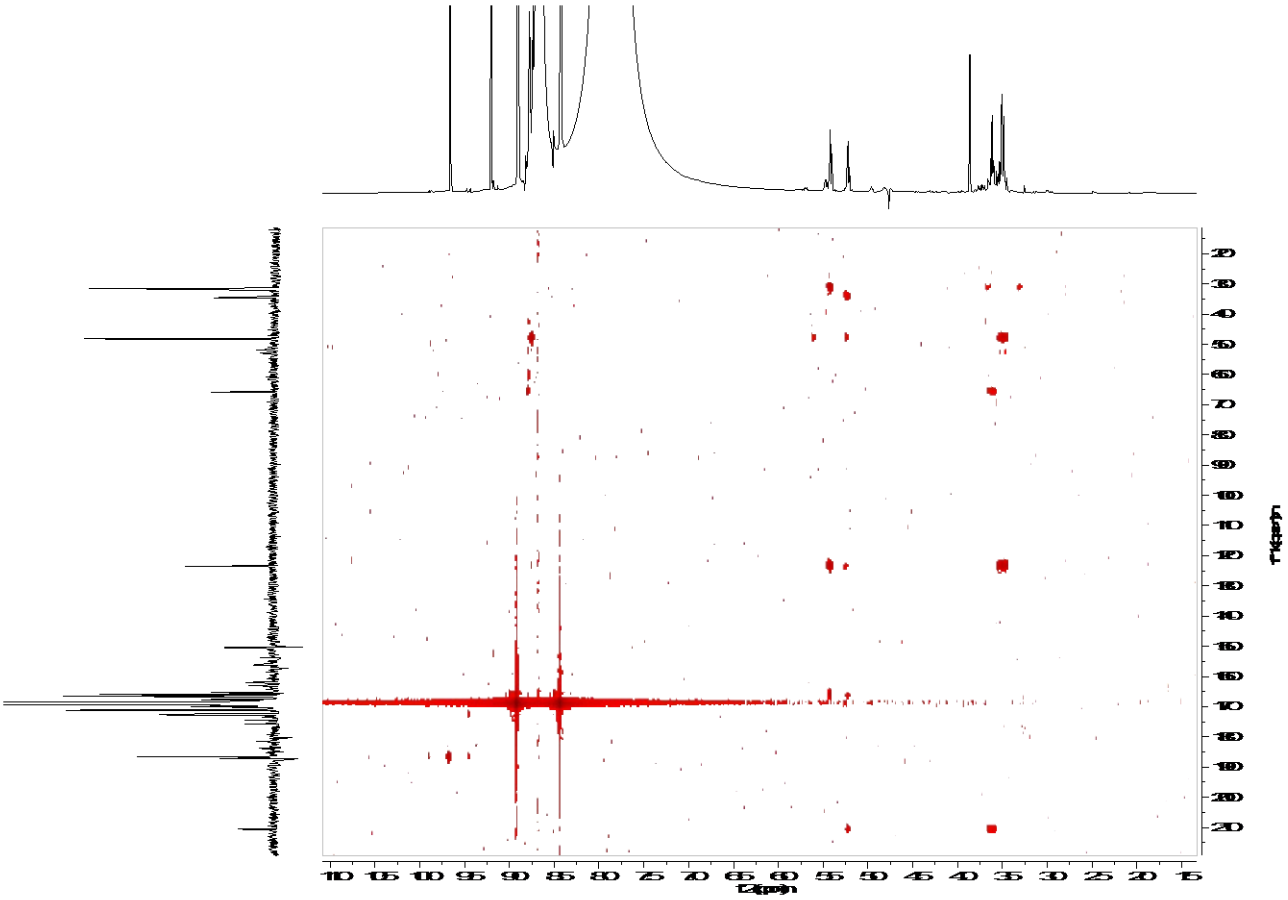

Fig S135. HMBC spectrum of the reaction between FoDHA-CN and NaSH.H_2O (3.0 eq.) in formamide, room temperature, 36h.

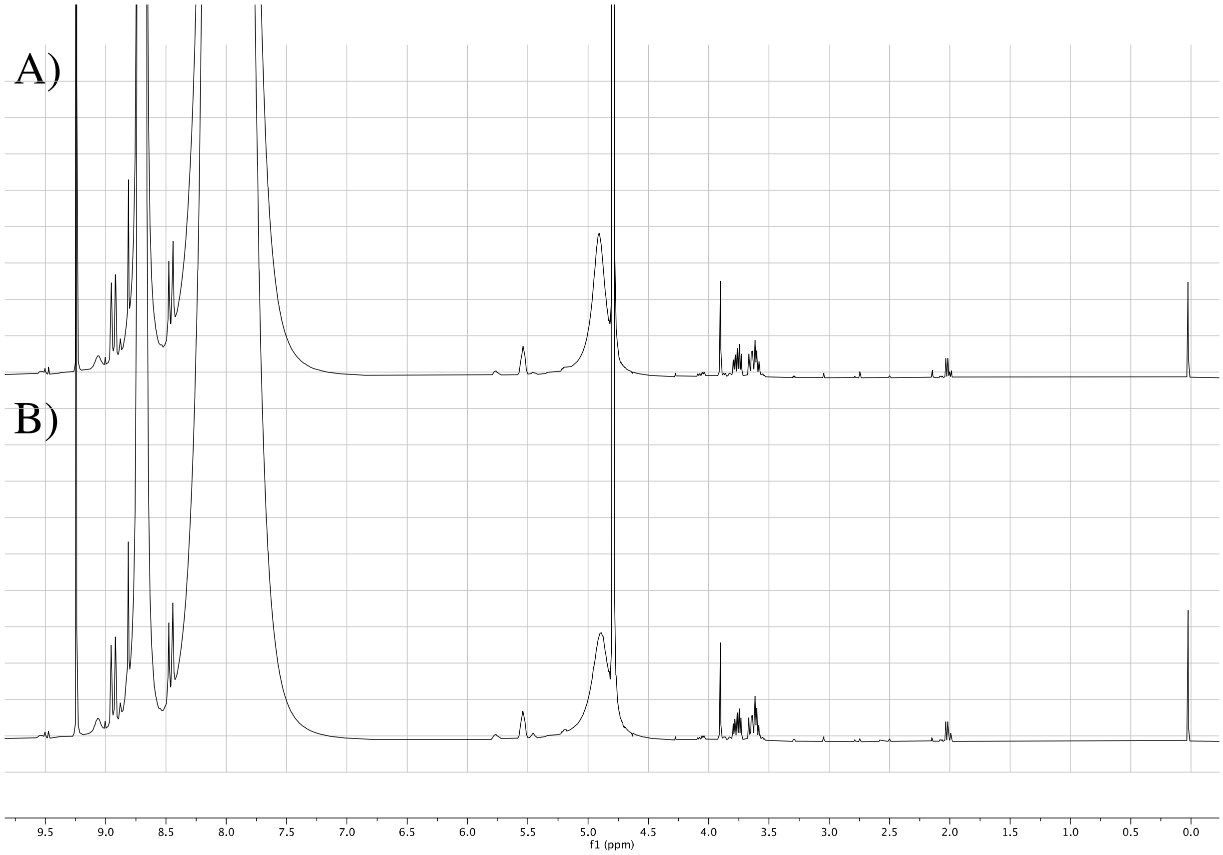

Fig S136. Stack of ^{1}H NMR spectra showing the reaction between FoDHA-CN, NaSH.H_2O, and formic acid (3.0 eq. each) in formamide, room temperature A) 18h; B) 36 h.

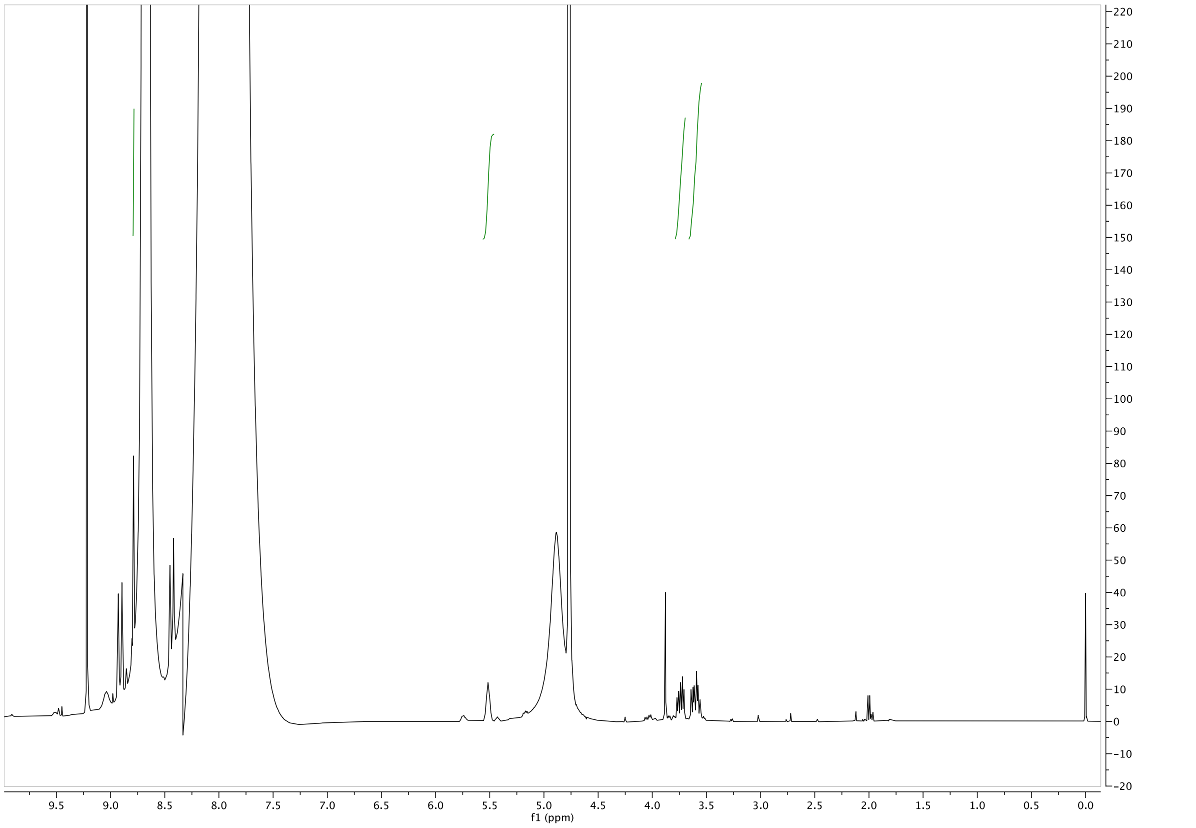

Fig S137. ^{1}H NMR spectrum of the reaction between FoDHA-CN, NaSH.H_2O, and formic acid (3.0 eq. each) in formamide, room temperature, 36h.

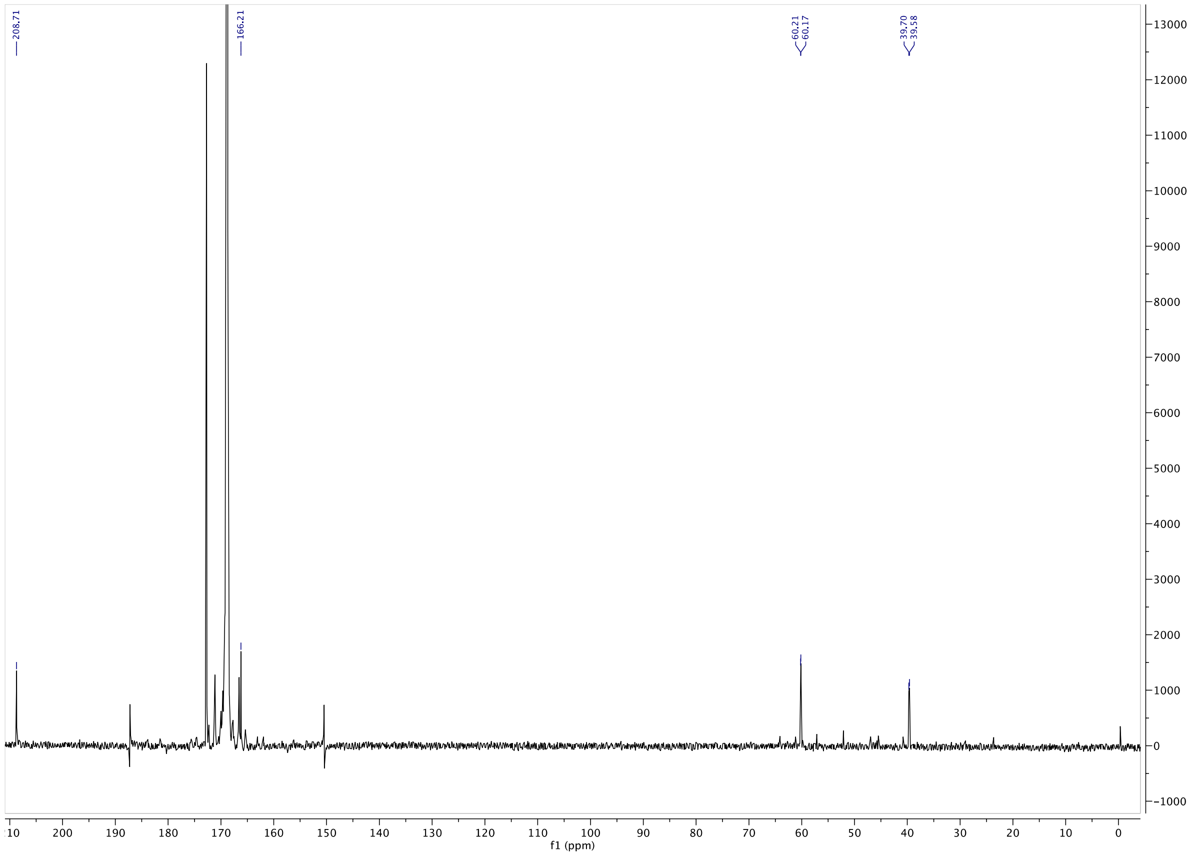

Fig S138. ^{13}C NMR spectrum of the reaction between FoDHA-CN, $NaSH.H_2O$, and formic acid (3.0 eq. each) in formamide, room temperature, 36h.

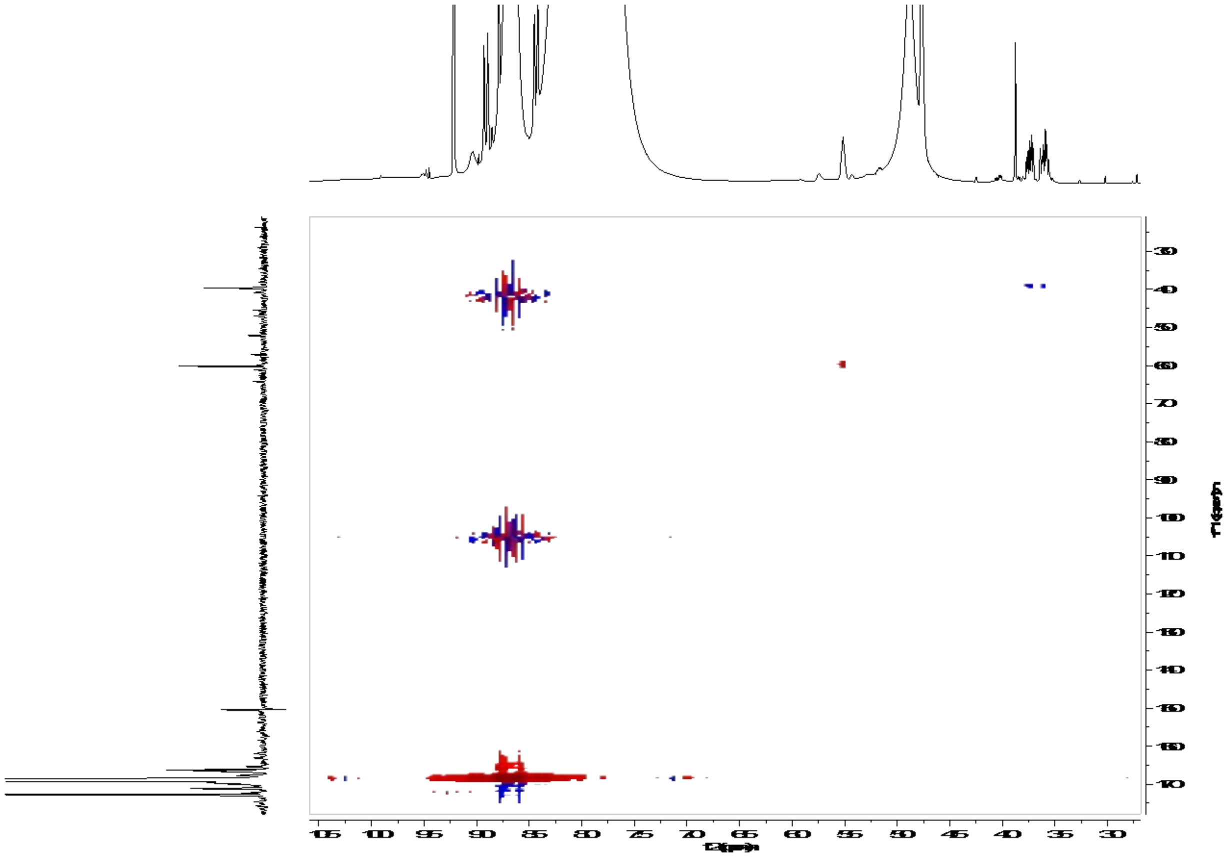

Fig S139. HSQC spectrum of the reaction between FoDHA-CN, $NaSH.H_2O$, and formic acid (3.0 eq. each) in formamide, room temperature, 36h.

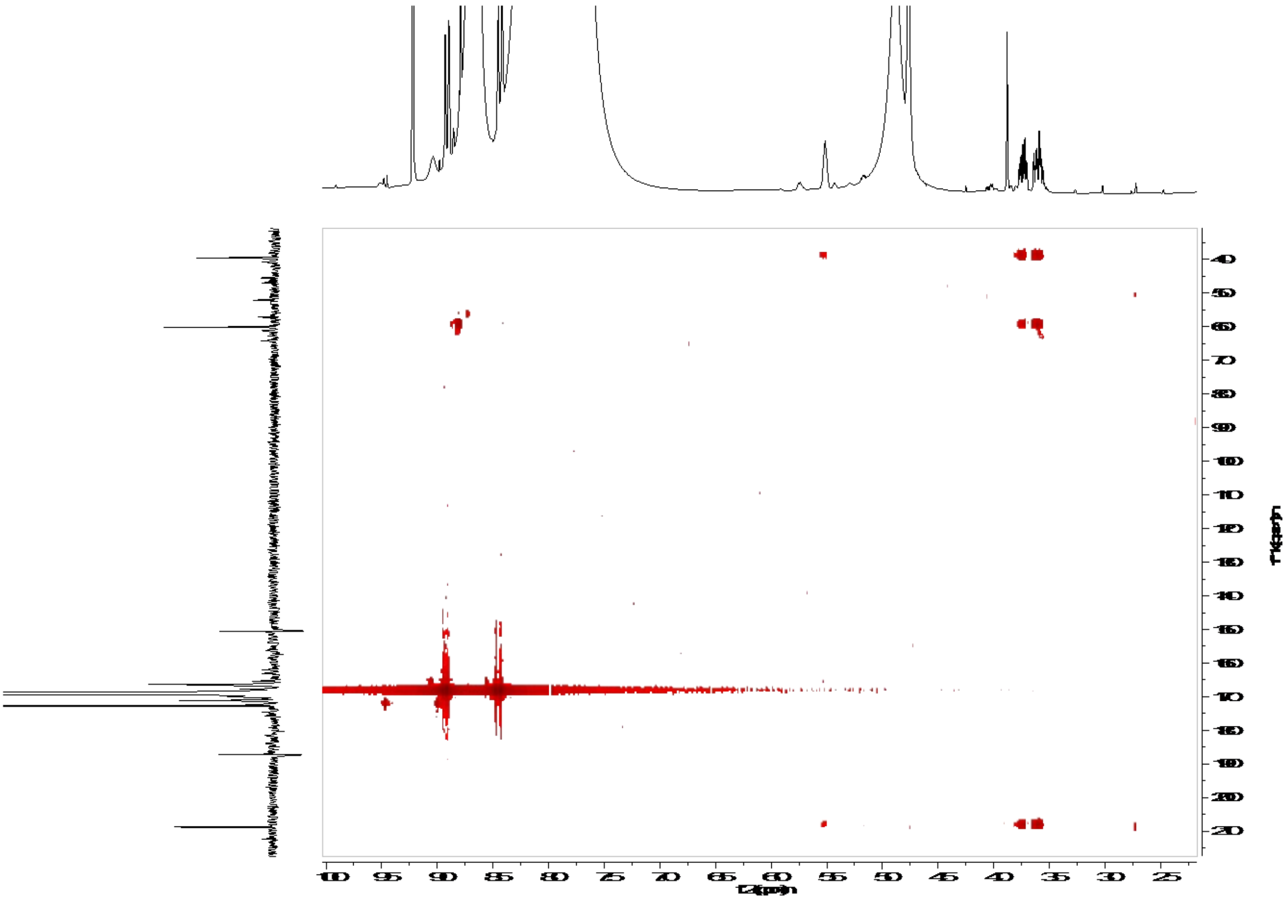

Fig S140. HMBC spectrum of the reaction between FoDHA-CN, $NaSH.H_2O$, and formic acid (3.0 eq. each) in formamide, room temperature, 36h.

HYDROLYSIS

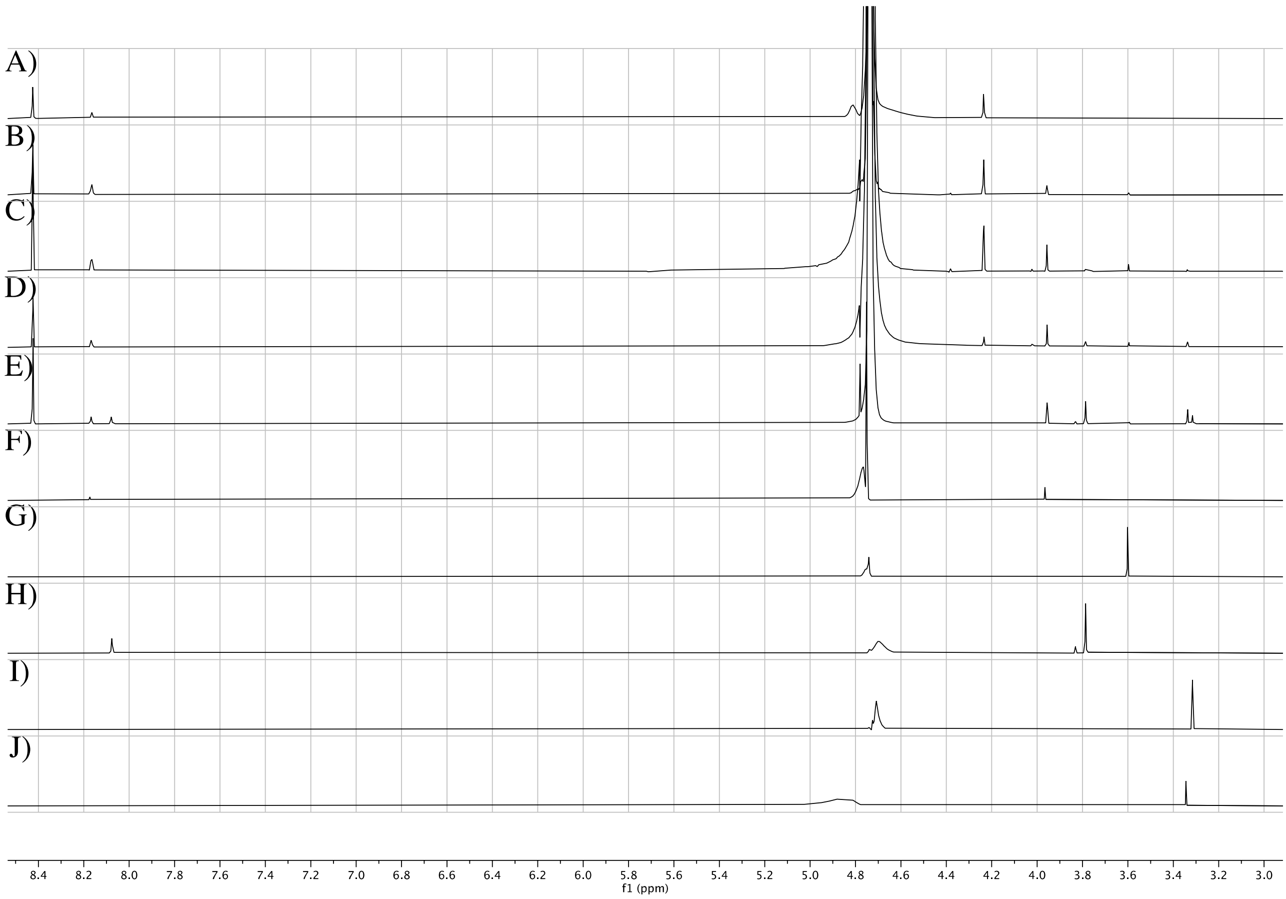

Fig S141. Stack of 1H NMR Spectra for hydrolysis of FoGlyCN, pH 10, 40 °C. A) Time zero. B) 18 h C) 36 h D) 105 h E) 345 h F) FoGlyNH$_2$ G) GlyCN H) FoGly I) Gly J) GlyNH2

Table S3. % hydrolysis of FoGlyCN

	Yields					
Time (hours)	GlyOH	GlyNH$_2$	GlyCN	FoGlyOH	FoGlyNH$_2$	SM
0						1.00
18		0.01	0.04	0.01	0.20	0.73
36		0.01	0.06	0.02	0.30	0.57
105	0.01	0.08	0.06	0.01	0.51	0.24
345	0.12	0.16	0.02	0.32	0.30	0.01

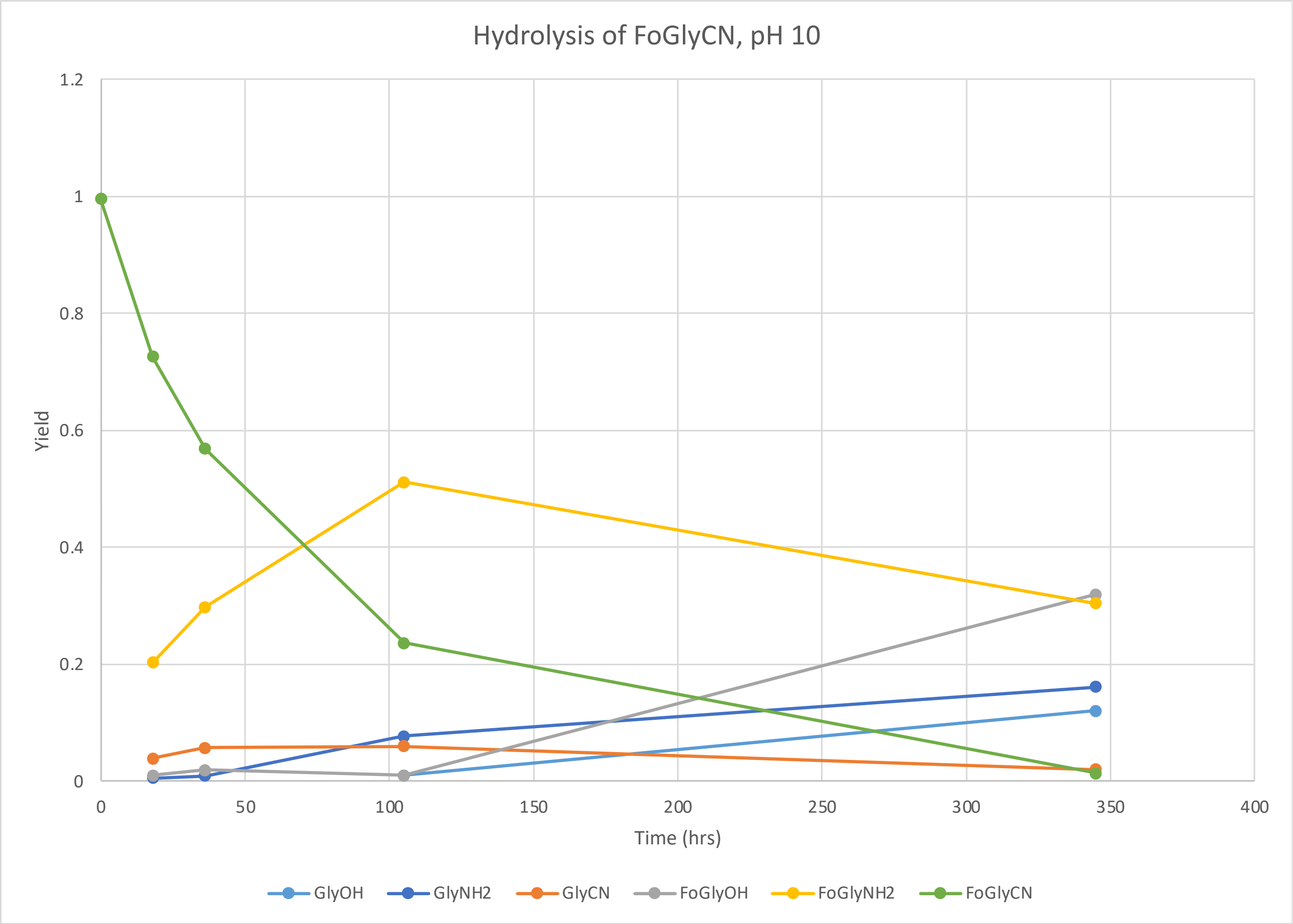

Graph S7. Hydrolysis of FoGlyCN at pH 10, 40 °C.

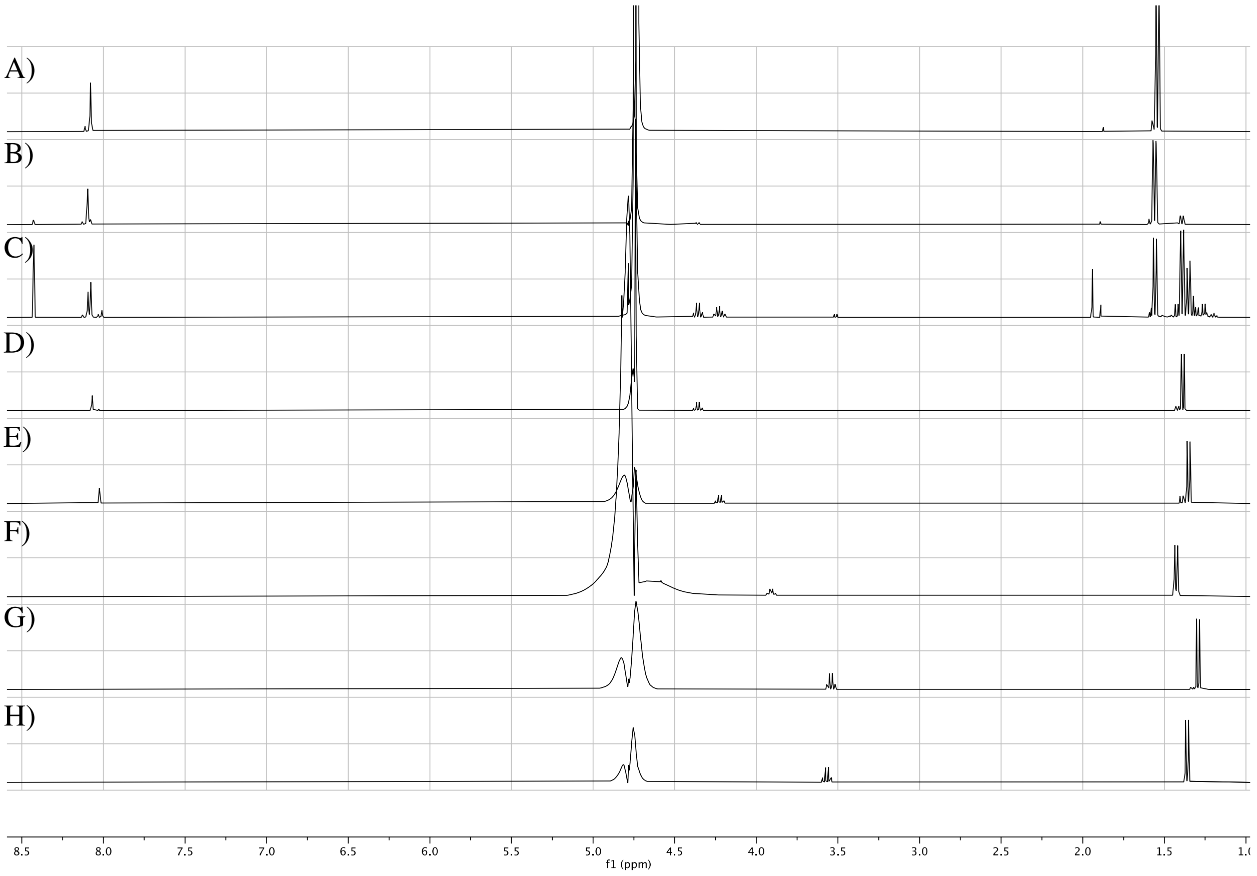

Fig S142. Stack of ^{1}H NMR Spectra for hydrolysis of FoAla-CN, pH 10, 40 °C. A) Time zero. B) 24 h C) 266 h D) FoAlaNH$_2$ E) FoAlaOH F) AlaCN G) AlaNH$_2$ H) AlaOH

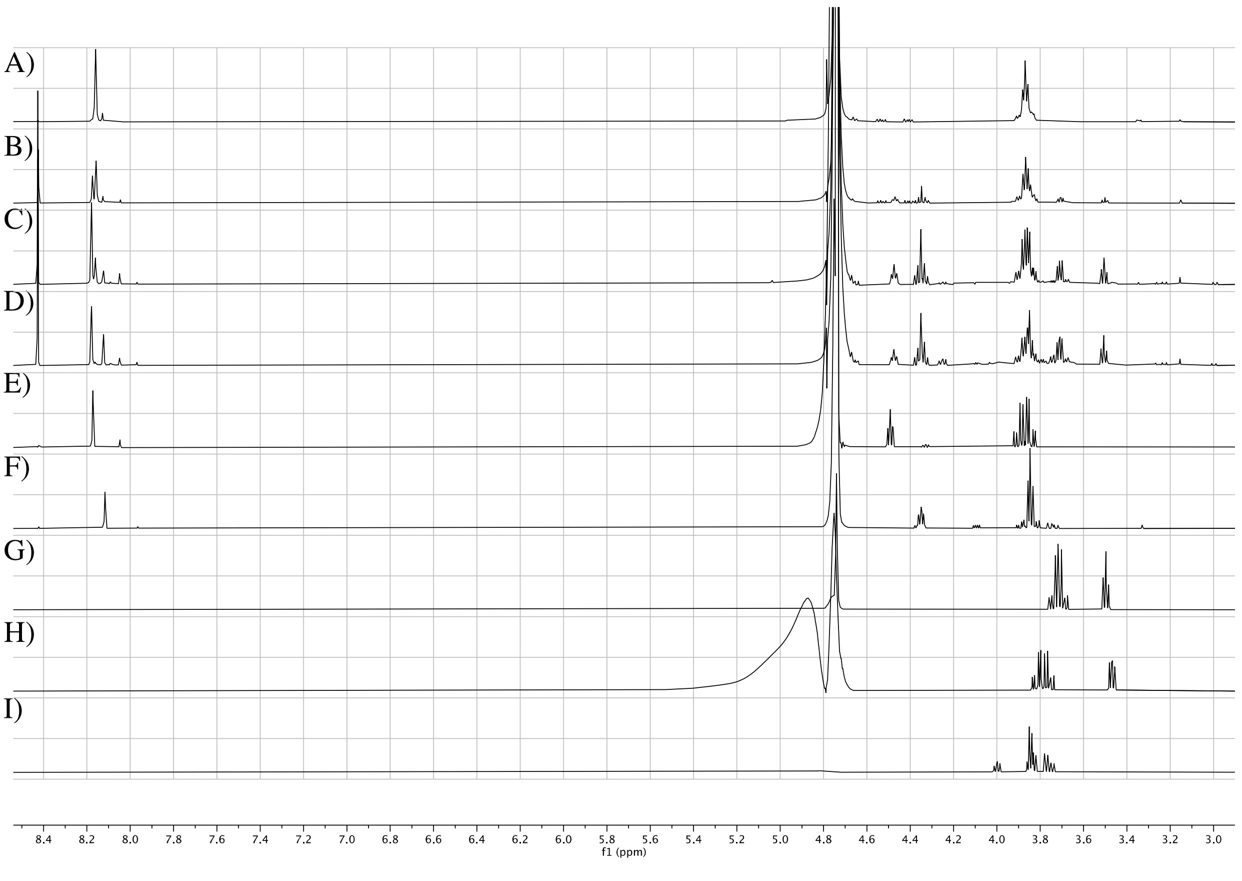

Fig S143. Stack of ^{1}H NMR Spectra for hydrolysis of FoSerCN, pH 10, 40 °C. A) Time zero. B) 24 h C) 77 h D) 237 h E) FoSerNH$_2$ F) FoSerOH G) SerNH$_2$ H) SerOH I) SerCN (+ glycolaldehyde impurity)

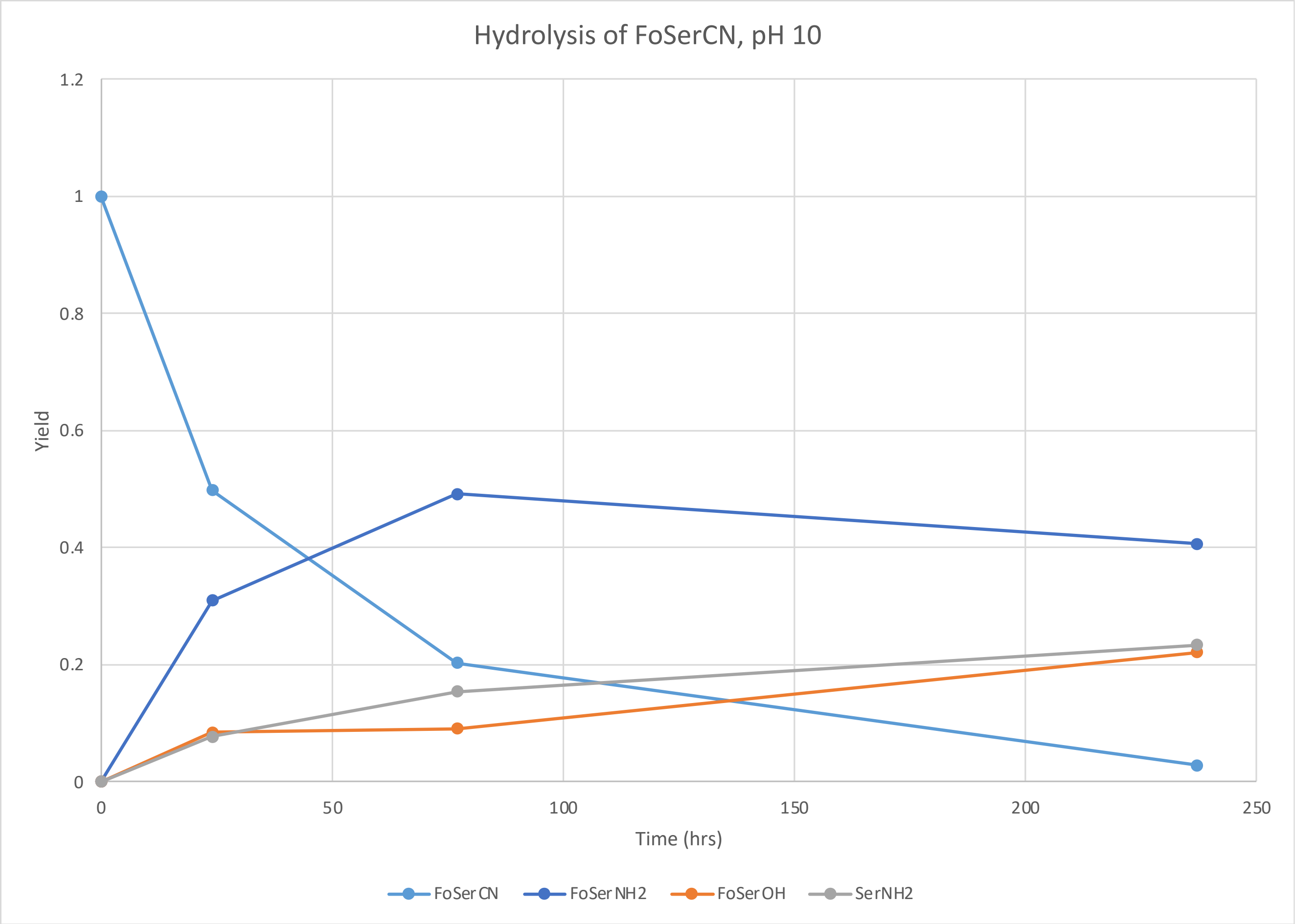

Graph S8. Hydrolysis of FoSerCN at pH 10, 40 °C.

Table S4. % hydrolysis of FoSerCN

Time (h)	FoSerCN	ForSerNH$_2$	FoSerOH	SerNH$_2$
0	1	0	0	0
24	0.50	0.31	0.08	0.08
77	0.20	0.49	0.09	0.15
237	0.03	0.41	0.22	0.23

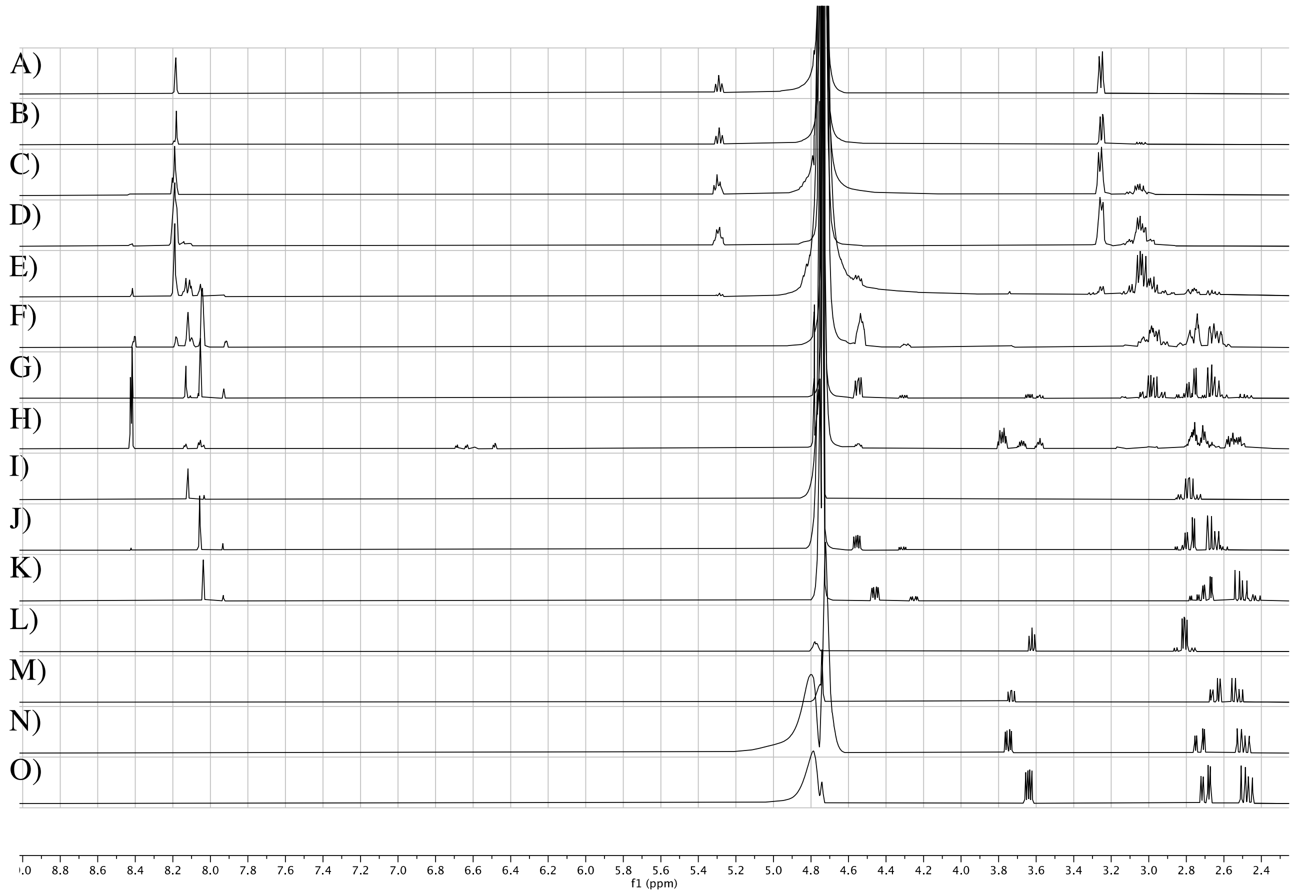

Fig S144. Stack of ^{1}H NMR spectra for hydrolysis of Fo(β-CN)Ala-CN, pH 10, 40 °C. A) Time zero. B) 1 h C) 3 h D) 6 h E) 23 h F) 47 h G) 275 h H) + 14 days 70 °C I) FoAsnNH2 J) FoAsn-OH K) FoAsp-OH L) (β-CN)Ala-OH M) $AsnNH_2$ N) Asp-OH O) Asn-OH

15N labelled studies

Formylation reaction in formamide in Table 1, entry 3a, was conducted as above, but in parallel using labelled (^{15}N)ValCN.HCl and unlabelled ValCN.HCl as starting material. The reaction was momitored by ^{1}H NMR, ^{15}N NMR, and LCMS (Atlantis T3 column, H_2O/MeCN solvent, ESI-). A sample of labelled (^{15}N)FoValCN prepared as above by formylation of (^{15}N)ValCN.HCl with EDCI and formic acid was used as a standard to determine the retention time.

LCMS indicated majority retention of (^{15}N) label (MW +0.68, see Table S5 below, Fig S146). ^{15}N NMR was in agreement, with a ^{15}N peak visible for the product (see Fig S147). Additional heating for 20h at 80 °C resulted in a slight erosion of label retention (MW +0.57). From these data we conclude that the major mechanism of formylation in Table 1 is via formylation of the nitrogen of the aminonitrile, occuing via acyl substitution on formamide. Reversion of Strecker condensation would result in unlabelled product (Fig S145).

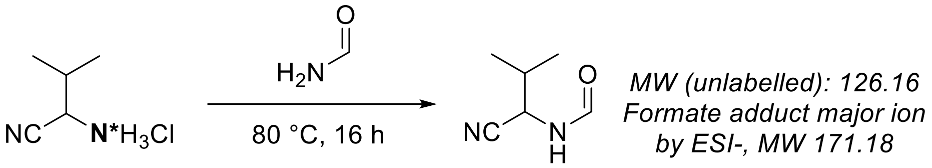

	LCMS MS peak intensity @ 10.9min (%)			MW
**Mass**	**171**	**172**	**173**	
**Unlabelled reaction**	90.9	8.0	1.1	171.15
**Labelled reaction**	27.8	66.0	6.2	171.83
**Labelled standard**	0.4	91.1	8.4	172.18

Table S5. LCMS data for formylation in formamide of unlabelled and (^{15}N)-labelled starting materials, and a synthesised labelled standard.

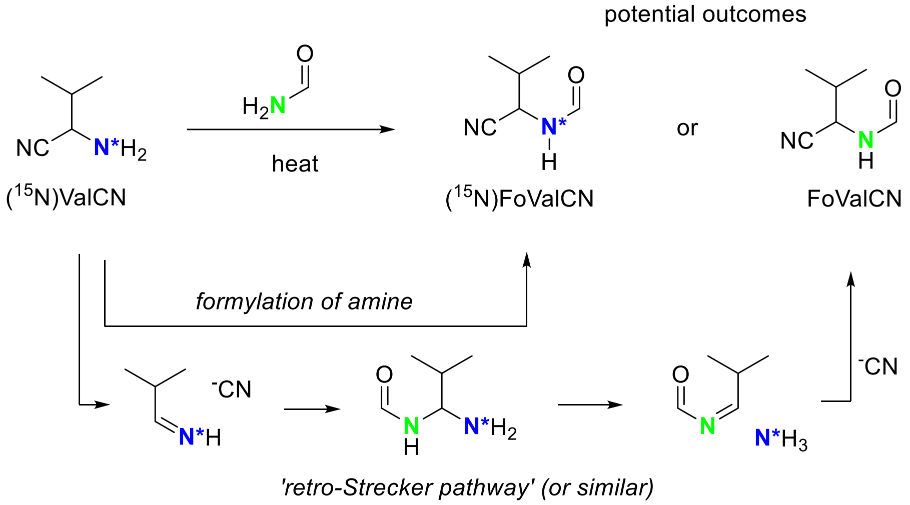

Fig S145. Potential mechanisms of formylation for the reaction in Table 1. The ‘retro-Strecker’ pathway could proceed via many possible intermediates, but the key point is that the source of nitrogen is formamide, not the starting material amine group, in this pathway.

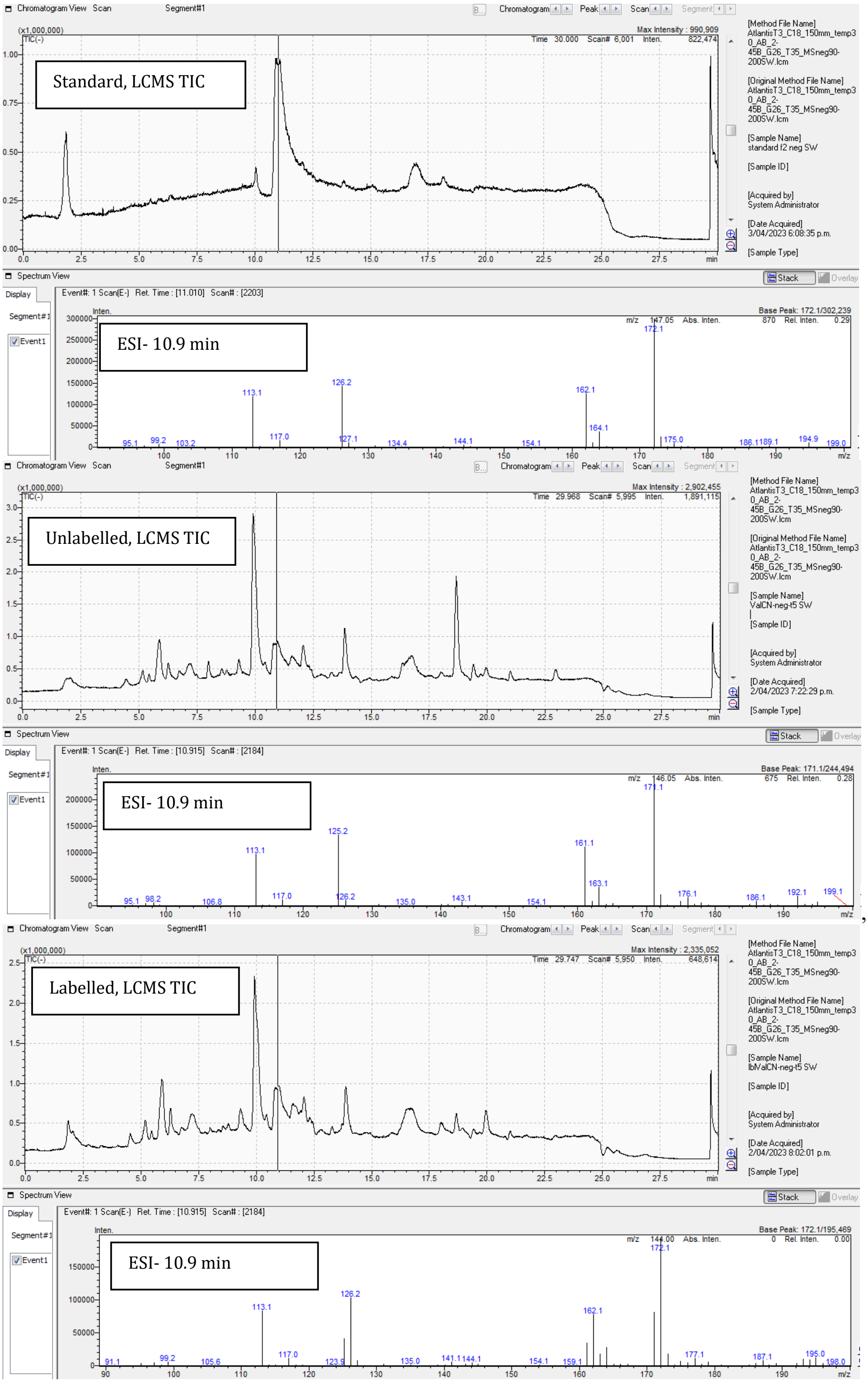

Fig S146. LCMS stack showing Total Ion Count (TIC) and ESI- Mass spec at 10.9 min retention time, for synthesised standard, unlabelled formylation reaction and (^{15}N)-labelled formylation reaction. The compound does not ionise particularly well, hence the relatively small peak in the TIC for the formylation reactions.

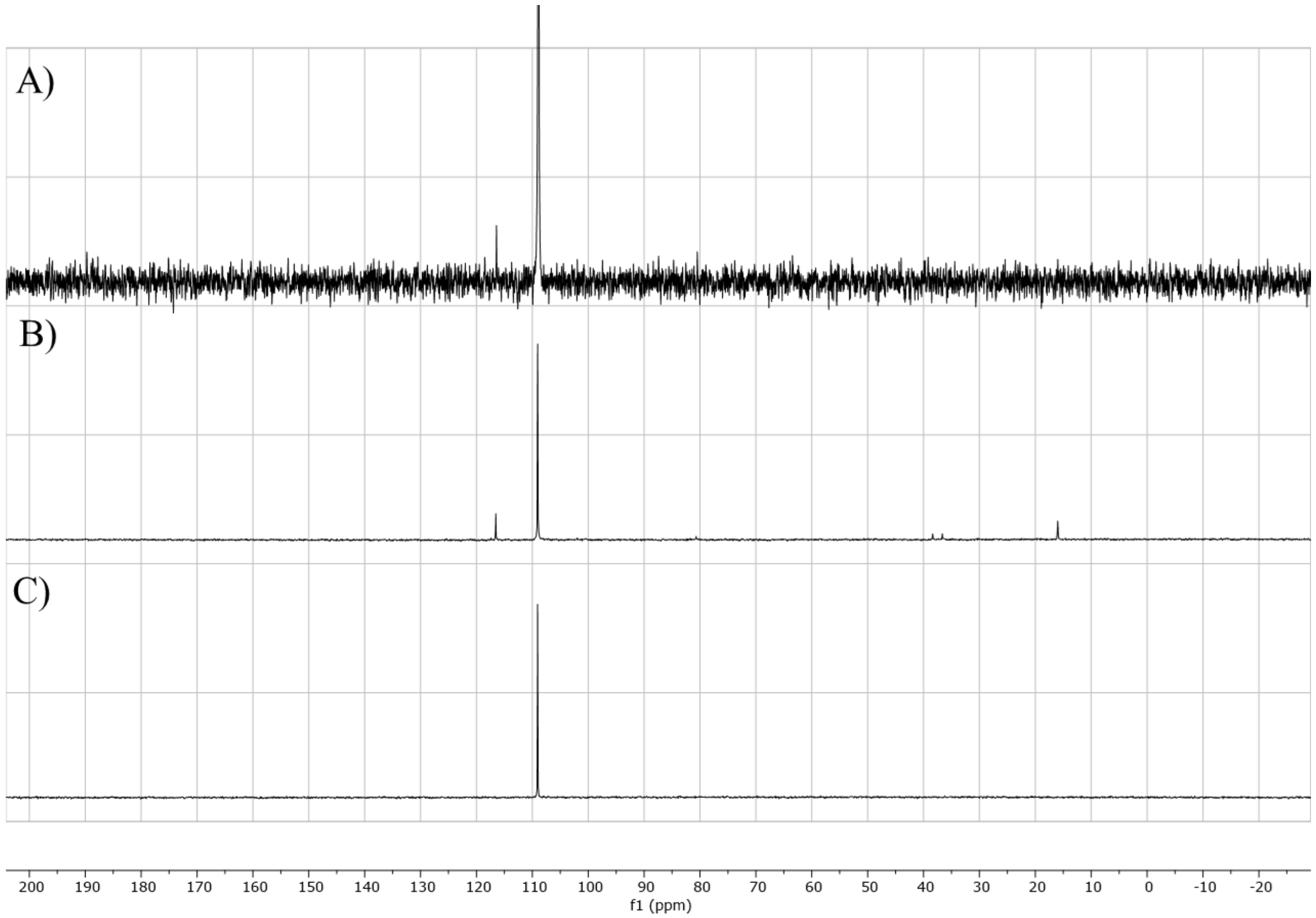

Fig S147. ^{15}N NMR stack showing a) Synthetic standard of (^{15}N)FoValCN in formamide; b) Formylation in formamide of (^{15}N)ValCN.HCl after 16 h at 80 °C; c) Formylation in formamide of ValCN.HCl after 16 h at 80 °C. Large peaks are residual (^{15}N)formamide.
